# Structured RNAs and synteny regions in the pig genome

**DOI:** 10.1186/1471-2164-15-459

**Published:** 2014-06-10

**Authors:** Christian Anthon, Hakim Tafer, Jakob H Havgaard, Bo Thomsen, Jakob Hedegaard, Stefan E Seemann, Sachin Pundhir, Stephanie Kehr, Sebastian Bartschat, Mathilde Nielsen, Rasmus O Nielsen, Merete Fredholm, Peter F Stadler, Jan Gorodkin

**Affiliations:** Center for non-coding RNA in Technology and Health, University of Copenhagen, DK-1870 Frederiksberg, Denmark; Department of Clinical and Animal Sciences, University of Copenhagen, DK-1870 Frederiksberg, Denmark; Bioinformatics Group, Department of Computer Science, Interdisciplinary Center for Bioinformatics, Universität Leipzig, Härtelstr. 16 - 18, D-04107 Leipzig, Germany; Transcriptome Bioinformatics group, LIFE, Leipzig Research Center for Civilization Diseases, Universität Leipzig, Philipp-Rosenthal-Strasse 27, D-04107 Leipzig, Germany; Department of Molecular Biology and Genetics, Aarhus University, Blichers Allé 20, DK-8830 Tjele, Denmark; Department of Molecular Medicine (MOMA), Molecular Diagnostic Laboratory, Aarhus University Hospital, Skejby, Brendstrupgaardsvej 100, DK-8200 Aarhus N, Denmark; GenoSkan A/S, Niels Pedersens Allé 2, DK-8830 Tjele, Denmark

## Abstract

**Background:**

Annotating mammalian genomes for noncoding RNAs (ncRNAs) is nontrivial since far from all ncRNAs are known and the computational models are resource demanding. Currently, the human genome holds the best mammalian ncRNA annotation, a result of numerous efforts by several groups. However, a more direct strategy is desired for the increasing number of sequenced mammalian genomes of which some, such as the pig, are relevant as disease models and production animals.

**Results:**

We present a comprehensive annotation of structured RNAs in the pig genome. Combining sequence and structure similarity search as well as class specific methods, we obtained a conservative set with a total of 3,391 structured RNA loci of which 1,011 and 2,314, respectively, hold strong sequence and structure similarity to structured RNAs in existing databases. The RNA loci cover 139 cis-regulatory element loci, 58 lncRNA loci, 11 conflicts of annotation, and 3,183 ncRNA genes. The ncRNA genes comprise 359 miRNAs, 8 ribozymes, 185 rRNAs, 638 snoRNAs, 1,030 snRNAs, 810 tRNAs and 153 ncRNA genes not belonging to the here fore mentioned classes. When running the pipeline on a local shuffled version of the genome, we obtained no matches at the highest confidence level. Additional analysis of RNA-seq data from a pooled library from 10 different pig tissues added another 165 miRNA loci, yielding an overall annotation of 3,556 structured RNA loci. This annotation represents our best effort at making an automated annotation. To further enhance the reliability, 571 of the 3,556 structured RNAs were manually curated by methods depending on the RNA class while 1,581 were declared as pseudogenes. We further created a multiple alignment of pig against 20 representative vertebrates, from which RNAz predicted 83,859 *de novo* RNA loci with conserved RNA structures. 528 of the RNAz predictions overlapped with the homology based annotation or novel miRNAs. We further present a substantial synteny analysis which includes 1,004 lineage specific *de novo* RNA loci and 4 ncRNA loci in the known annotation specific for Laurasiatheria (pig, cow, dolphin, horse, cat, dog, hedgehog).

**Conclusions:**

We have obtained one of the most comprehensive annotations for structured ncRNAs of a mammalian genome, which is likely to play central roles in both health modelling and production. The core annotation is available in Ensembl 70 and the complete annotation is available at http://rth.dk/resources/rnannotator/susscr102/version1.02.

**Electronic supplementary material:**

The online version of this article (doi:10.1186/1471-2164-15-459) contains supplementary material, which is available to authorized users.

## Background

With the sequencing of the human genome it became evident that protein coding sequences only make up about ∼1.2% of a mammalian genome [1]. It has since then been a main challenge to analyze the remaining part of the genome. A non-negligible fraction of the genome consist of noncoding RNAs (ncRNAs), an abundant class of genes which are not translated to proteins and instead function directly in the RNA form. In spite of the progress in both computational RNA biology and experimental techniques, annotating ncRNAs in mammalian genomes remains a major challenge. The challenge is to carefully pin point only *functional* ncRNAs, which can be done through similarity to existing ncRNAs or *de novo* through a combination of RNA structure prediction and RNA-seq data. The latter may with subsequent effort reveal full length transcripts, yet with unidentified function [2] and functional assignment will require tedious lab work on each instance.

The human genome is the most comprehensively annotated mammalian genome. Yet, the annotation of ncRNAs is still an ongoing effort, which was initiated with the release of the human genome more than a decade ago based on similarity search [3] and have expanded several times most noteworthy in the recent ENCODE and GENCODE projects [4–6]. Within the most recent GENCODE annotation ∼9300 long ncRNAs (lncRNAs) have been identified [6] by manual intervention, though their functionality in general is yet to be uncovered.

More and more mammalian genomes are being sequenced (the NCBI website as of May 2013 lists assemblies for 119 mammalian genomes). Thus it becomes important to annotate ncRNAs in mammalian genomes at least in a version containing the most well established RNAs and RNA families. The annotation of new mammalian genomes will in general build upon the existing annotation of the human genome; however, the corresponding number of years of effort is not feasible and a more direct strategy is needed.

The most comprehensive collection of structured RNAs is found in Rfam [7, 8], which has been constructed in a large collaborate effort by the (noncoding) RNA community. However, even in Rfam the annotations lack explicit functional annotation and the models for structured RNAs do not always hold a strong discrimination power to pseudogenes, as these may be incorporated into the Rfam seed sequences on which the structure models are based [9]. It is in general not possible to distinguish between real genes and pseudogenes, except for a few cases, exemplified by tRNAs [10].

Analysis on early and incomplete pig sequencing data for structured RNAs have been carried out previously both on genomic as well as on EST data [11–13]. Whereas, the annotation of EST sequences was based on *de novo* assembly comparison of the resulting contigs to related organisms, the annotation of genomic DNA as fragmented due to low coverage and annotation was carried out for all of the contiguous pieces in a similar way. Interestingly the EST annotation provided additional expression profiles as well. These studies on the incomplete genome included discovery of homologues as well as *de novo* predicted ncRNAs. As it was shown for the full transcriptomic analysis on the pig ESTs, the most diverse expression is in brain and testes tissues [12] and particular brain and developmental tissues hold a relatively higher expression of putative ncRNAs [13]. The search for lncRNAs in the pig genome has just begun and includes expression analysis of an mRNA-like ncRNA [14], suggestion of an mRNA-like ncRNA [15] and two other cases in the lncRNA database [16]. Other studies indicate a larger number of putative lncRNAs in pig [17]. Also, recent work has indicated a further potential for ncRNAs in *Sus scrofa*, for example, by the use of the genomic sequence to map small RNAs in the cumulus-oocyte complex and in early embryos in the pig [18]. Likewise for the discovery of miRNAs in the pig intestine [19] and in pig skeletal muscles [20]. While these provide initial information, the sequencing of the full genome for the first time opens for a systematic analysis and annotation of ncRNAs in pig. By annotating the pig genome for ncRNAs a range of related production animals *e.g.*, cow are being annotated as well using comparative genomics in this study.

When annotating genomes of model animals such as the pig it is also of interest to narrow down organisms or lineage specific ncRNAs, as one would like to avoid these in studying basic genetic mechanisms which can be used as a template in for example human diseases. Conversely, the lineage specific ncRNAs can potentially be highly relevant when studying the underlying genetic mechanisms of not only animal health, but also production.

To address the problem of ncRNA annotation of a mammalian genome using a reasonable amount of man-power resources, we here focus on structured RNAs, a main characteristic of many ncRNAs and regulatory elements in UTRs of mRNAs. We introduce a pipeline using the complementing resources of sequence or structure homology search, and small RNA-seq data analyzed for the potential of miRNAs. On top of this we address *de novo* annotation by employing RNAz [21]. The pipeline exploits that a range of related organisms exists where the genomes match up in regions extending beyond the individual structured RNAs. This enables manual curation of the structured RNAs and allows for synteny analysis of the annotation produced in this paper. The recent sequencing of the pig genome has provided insight into a range of medical, production and evolutionary aspects [22]. The full genome sequence, furthermore, makes it possible to obtain a good overview of the structured RNAs, genes as well as well as regulatory RNA structure, all with relevance for the mentioned topics.

## Results

### The modules of the pipeline

In the following section we present a pipeline for annotation of structured RNAs. The pipeline is targeted towards annotation of complete vertebrate genomes, but could in principle be used to annotate other types of genomes or even a collection of individual sequences. The pipeline also adds additional information to annotation in the form of synteny and contextual information such as close-lying protein coding genes.

**Figure 1:**
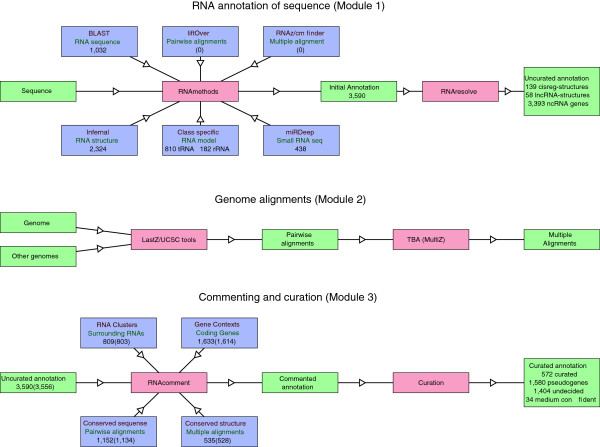
**Pipeline.** The modules of the RNA pipeline. Module 1 (annotation): The annotation pipeline takes as input any number of sequences and runs a number of external annotation tools on it (see text for details). This leads to the initial annotation of the RNA loci in the sequence. A naming and resolving tool decides on the final annotation of the locus. The 3,393 ncRNA genes cover 11 conflicts of annotation, 34 loci moved to the medium confident annotation during the curation step, 165 novel miRNA loci found exclusively by miRDeep, and 3,183 ncRNA genes found by homology. LncRNA loci and cis-regulatory elements are annotated separately. Module 2 (multiple alignments): The multiple alignment pipeline runs on a genomic scale and aligns the genomic sequence of the input genome against any number of other genomes, finally forming multiple alignments in MAF blocks. Module 3(post processing): The post-processing part of the pipeline adds context to the RNAs, which in many cases will allow for a curation of the structured RNA loci. The numbers in parenthesis are those obtained after the removal of 34 annotations as part of the curation procedure: 12 tRNAs, 15 homology based miRNAs, and 7 *de novo* miRNAs.

The pipeline for annotation of structured RNAs presented in Figure 1 is based on three main modules, (*i*) annotation, (*ii*) pairwise and multiple alignments, and (*iii*) tagging of the annotation. The last module adds additional information to the annotation.

*Module (i):* the annotation pipeline. Here structured RNA *loci* in a given input sequence(s) are annotated (an RNA *locus* is in this work defined as a set of overlapping RNA structure or sequence annotations). The annotation is based on a number of methods, classified as either sequence based homology search, structure based homology search, RNA class specific methods, or *de novo* structured RNA prediction. The sequence based homology search is presently based on BLAST [23] and a number of databases of known ncRNA sequences (see Section RNAs of the individual annotation tools for details). Structure based homology search is performed with Infernal [24] and covariance models (CM) from Rfam [7, 8]. Class specific methods are typically based on sequence or structure homology search but with some additional knowledge about the RNA, at present tRNAscan-SE [10], and RNAmmer [25] are built into the pipeline. snoStrip [26] is another class specific tool used in the present study, but is not yet fully integrated into the pipeline. The prediction of structured *de novo* ncRNAs requires information in addition to the sequence to be reliable, either in the form of multiple alignment of (part of) the input sequence used with tools like RNAz [21] or CMfinder [27], or in the form of RNA-seq data used with tools like miRDeep [28]. This submodule of the pipeline thus requires additional user supplied input data, which sets this submodule aside from the other three classification methods, which work on the input sequence alone. Levels of certainty of the RNA predictions are provided, as high, medium, low corresponding to the different cutoff levels for the individual tools, and the input to the annotator module will depend on the input from the tools as well as the confidence level. An overview of the cutoff levels are given in Table 1. The next step is to mark and resolve the conflicts that are introduced by the individual tools, or introduced by the merging of the results of the tools. Some tools are inherently conflict free like RNAmmer, tRNAscan-SE and snoStrip. Some tools, however, are supposed to be conflict free, like the Rfam models investigated with Infernal. Also when employing a tool like BLAST on a number of databases it is expected that a number of conflicts are introduced. The conflicts introduced by running BLAST were partly resolved by choosing the hit with the lowest E-value within each database and class of RNAs, *e.g.*, miRNAs or snoRNAs. Conflicts between Rfam families introduced by running Infernal were marked. Conflicts of annotation introduced by different databases and conflicts introduced by different tools between databases are only marked if they represent a conflict in classification, *e.g.*, a genomic locus is marked as a conflict if annotated as both snoRNA and miRNA. Different tools may indicate different names for the same RNA. We assumed that if two tools both classified a loci as belonging to the same class, but that the name used by the two tools were different, this did not indicate a real conflict, but only a conflict in naming. Therefore a name of the RNA is chosen based on a specific ranking of the tools annotating the loci. In general, the class specific methods add extra information to the RNAs that they annotate (*e.g.*, tRNAscan-SE tests for the codon) or they annotate full length RNAs, *e.g.*, RNAmmer, where BLAST or Infernal may be unable to. The predictions made by high confident BLAST runs will normally be more confident than the ones obtained from Infernal on the same model. Therefore, in the naming procedure class specific methods are preferred over BLAST, which in turn is preferred over Infernal/Rfam. For a full overview see Additional file 1: Table S1. *De novo* methods like miRDeep and RNAz are only used as a last resort in the naming and classification procedure.

**Table 1:** **Cutoffs for individual tools at different global confidence levels**

Comp. strategy	High	Medium	Low
BLAST	95% id 95% length	92.5% id 92.5% length	90% id 90% length
Infernal	BLAST E=1e-3	BLAST E=0.1	No BLAST filter
	Infernal E=1e-3	Infernal E=1e-3	Infernal E=1e-3
Infernal miRNA	Not applied	BLAST E=1e-3	BLAST E=0.1
		Infernal E=1e-9	Infernal E=1e-6
tRNAscan-SE	High	High	As is
RNAmmer	As is	As is	As is
snoStrip	As is	As is	As is
miRDeep	Hand cleaned	As is	As is

The results from the individual tools are merged at 3 different cutoff levels: high, medium and low. This table shows the correspondence between the cutoff levels of the merged annotation and those of the tools. For each computational (comp) strategy, we define these levels. Note that Infernal screen have been divided into Infernal (without miRNAs) and Infernal miRNA which is *only* for miRNA families. The Infernal results were filtered by the family specific gathering scores as well. For *As is*, we refer to the programs default values for the respective versions (see the Methods section for version numbers) without subsequent cleaning.

The result of the two steps of module (*i*) is an uncurated annotation which is to be analysed and curated in module (*ii*) and (*iii*).

*Module (ii):* Pairwise and multiple alignments. This module works on a full genome and aligns the genome against any number of related genomes. A user supplied phylogenetic tree is needed as well as a decision on the parameters to use for the pairwise alignments. Three sets of parameters for the pairwise alignments are provided: 1) highly related genomes, 2) related genomes, 3) distantly related genomes. These parameter choices are based on those used for the UCSC 46-way alignment of the human genome, where the three sets approximate the respective choices made for primates, placental mammals, and non-placental mammals as well as other vertebrates. The module is a framework built over pairwise alignments with LASTZ [29] and subsequent chaining and filtering with the UCSC [30] tool chain.

*Module (iii):* This module provides genomic context to the RNA annotation, or additional knowledge about the annotations. At present the following submodules are implemented: 1) Genic context, requires the Ensembl (protein coding) gene annotation. 2) Clustering of RNA loci, checks for other RNA loci annotated in the vicinity of the one in question. 3) Conservation of the sequence in other organisms derived from the pairwise alignments. 4) Conservation of structure derived from the multiple alignments using RNAz. Based on this information a curation of particular RNA loci or RNA classes is possible, which leads to the curated annotation.

The pipeline for RNA annotation presented above in module (*i*) is a flexible framework for RNA annotation, which may be extended by any number of future tools.

### RNAs of the individual annotation tools

The pipeline presented above was used to annotate the pig genome version 10.2. In this section we present and discuss the results of the RNA annotations obtained from the individual tools ordered by their classifications as sequence based homology search tools, structure based homology search tools, and class specific search tools. Details about the cutoffs used for the tools at high, medium and low confident levels are given in Table 1, while the medium and low confident results are mostly confined to Additional file 1.

*Sequence based homology search* using BLAST*w*as performed against a number of databases (see Methods section for details; an overview of the databases is also given in Additional file 1: Table S2). The databases have not been redundancy reduced and the sequences are used without modifications. The only exception is the miRNAs from the Rfam seeds, which had special problems and they were therefore excluded from the high confident results. The reason is that at least some Rfam miRNA families are inconsistent with miRBase (an example is mir-28, mir-708 where high confident BLAST Rfam hits are observed on the same locations on opposite strands, however miRBase only match one of the two families to a given location and the families are only weakly related. In the Rfam version 11 the two families are allowed some overlap by declaring them part of the same clan).

We found 1,032 high confident (95% id over 95% of the query length) RNA loci belonging to a total of 507 different RNA families see Additional file 1: Table S3 for the high confident sequence homology search results; the medium and low confident results are included in the table for completeness. Special care must be taken when using miRBase for annotation, since it contains both experimentally determined miRNAs and miRNAs determined by homology. In a few cases even purely *in silico* discovered miRNAs have entered the database (*e.g.*, [31]). This resulted in removal of 15 miRNA loci, which were found by homology to sequences without experimental evidence in miRBase, and which have no experimental support in this study (see Section Small RNA sequencing and novel miRNA predictions). In total we obtained 6 annotation conflicts, within the sequence similarity search results. The conflicts were always found to be between a miRNA from miRBase and a snoRNA (scaRNA or HACA box), or a miRNA from miRBase and SRP RNA from Rfam. For an overview of misannotation of miRNAs from small RNA-seq data see Langenberger *et al.* [32].

*Structure based homology search* was performed with Infernal [24] on the covariance models of the RNA families from Rfam (version 10.1) [7, 8]. Here, we only used the 695 families with seed sequences in vertebrates. Each Rfam family comes with a family dependent gathering score cutoff, however this alone proved insufficient for high confidence annotation since without additional filtering the scan of the Rfam families in the pig genome with Infernal yielded 709,026 loci, where 8% of them had conflicting Rfam annotation.

In light of the high number of matches presented by the unfiltered Infernal results we decided to impose additional filters on the structure based annotation. The details are found in the Methods section. In the current section, we touch upon the methodology and present the results of imposing these additional filters. For an overview see also Table 1.

The miRNA families come with special problems and must therefore be treated separately. These problems include families specifying a very broad range of miRNAs, which makes these families unsuitable for specific annotation and the Rfam miRNA families also come with a very high number of conflict loci without very strict filtering (see Methods section for details). For the non-miRNA families we in most cases use the Infernal E-value cutoffs corresponding to the family specific gathering score cutoff level. However, for families where this Infernal E-value cutoff would be higher than 1e-3 we keep it at 1e-3. For a sequence to be matched with high confidence to a particular family, we require that the sequence shows overlapping Infernal and BLAST matches to the family. For the high confident annotation we set the BLAST E-value cutoff to 1e-3. For the medium confidence annotation we lower the BLAST overlap requirement to a BLAST E-value cutoff of 0.1 and for the low confidence annotation we need no overlap in the BLAST comparison. Even a loose BLAST overlap requirement has the effect that it anchors the hit to the right family, thereby greatly reducing the number of conflicts and number of false family assignments at these levels.

Just as the Rfam precursor miRNA families were removed from the high confident sequence based annotation, we also removed them from the high confident structure based annotation (high confident homology based annotation of miRNAs is thus only performed for miRNA sequences from miRBase and only with sequence based homology search). For the medium and low confident annotations of Rfam miRNA families, we impose Infernal E-value cutoffs of 1e-9, and 1e-6, respectively. The miRNA precursor families are further required to have an overlapping BLAST hit, in both medium and low confidence annotations.

It is worth noticing some pitfalls of using Infernal with the Rfam families. First of all, we root out most problems by imposing a BLAST filter, by discarding the non-miRNA families, and by only looking at families with seeds in vertebrates. This causes the loss of a few families that otherwise would have been detected at lower cutoffs. For example, we find an extra 6 non-miRNA families at the lowest cutoff level. Two of them, VA and ACAT, are likely false positives without synteny to their origins in the original organisms. The other four, GAIT, CoTC_ribozyme, SNORD126 and ACA59, however, are syntenic to their human counterparts and could well be true matches that we miss because of the filtering. On the other hand, some families will still have false positives, pseudogenes or remote homologs, and we are left with no clear way to distinguish these by automatic means alone. We therefore proceed to curate the annotation in the coming sections. Hence, the high confident annotation represent a conservative automated structured RNA annotation, without curation of individual RNAs or RNA families.

We found 2,324 high confident loci with structure based homology distributed on 317 families (see Additional file 1: Table S4 for a summary of the structure homology based results). In contrast to the sequence similarity based results, conflicts are marked between overlapping Rfam families, rather than overlapping RNA classes. However, with the applied cutoffs we found conflicts only in the medium and low confident results, while the high confident results were free of conflicts. If we compare the 533 high confident BLAST results based on the Rfam database (see Additional file 1: Table S5) with the high confidence results of the Infernal run in Additional file 1: Table S4 we found that an additional 194 Rfam families and an additional 1,791 loci may be annotated with high confidence using structure based homology search.

*Class specific methods* were used to further enhance and extend the annotations of tRNAs (tRNAscan-SE), rRNAs (RNAmmer), and snoRNAs (snoStrip).

We scanned for tRNAs with tRNAscan-SE [10] in addition to the pure homology-search methods presented above. In the human genome tRNAscan-SE predicts a reasonable number of tRNAs (<1,000) but for other mammalian genomes number of tRNAs predicted by tRNAscan-SE is often much higher (See, *e.g.*, cow in the tRNAscan-SE database on the tRNAscan-SE homepage). Here we adopt the cutoffs used for cow in the tRNAscan-SE database to gain a set of high confident tRNA candidates (See Methods section for details). The unfiltered (or low confident) scan generated 32,303 tRNA loci compared to the 810 loci of the filtered results (high confident). 21 different tRNA types were found within the filtered results; tRNA.SeC were found in addition among the loci of the unfiltered results. The division of the tRNAscan-SE results on types are found in Additional file 1: Table S6.

The combination of tRNAs found by BLAST, Infernal and tRNAscan-SE produced 822 high confident results. However, as part of the cleanup of the annotation we changed the 12 tRNAs not detected by tRNAscan-SE from high to medium confident annotation. Thus reducing the number of tRNAs to 810. It is noteworthy that 389 of the 810 (48%) high confident tRNAs were found to be conserved in at least 5 other organisms (see Section *Human and pig synteny analysis* for details), while less than 2% of the 32,320 low confident tRNA candidates are conserved in at least 5 other organisms.

The RNAmmer [25] scan generated 182 rRNA loci subdivided on two 28S fragments, four 18S fragments and 176 8S fragments. The RNAmmer hits were considered to be high confident. The results of the RNAmmer scan were not directly comparable to the sequence similarity search since RNAmmer detects the larger ribosomal fragments, which BLAST have difficulties with, unless the hits are subsequently clustered. However, the 4 large ribosomal RNAs found by medium confident sequence similarity search are all included in the RNAmmer results.

snoStrip [26] generated a total of 173 snoRNA families in 513 loci, these are considered high confident results. In comparison, Infernal identified 508 Rfam based snoRNA loci. However, out of the 638 combined Infernal and snoStrip loci, 130 are unique to Infernal and 137 are unique to snoStrip. The annotation created by snoStrip will like the Infernal/Rfam snoRNAs include pseudogenes and possibly false positives as well (see the Methods section for false positives on shuffled sequence).

The annotation obtained in this section represents the raw annotation, which in the next section will be refined.

### Merged annotations of the homology based RNA loci

The annotations obtained from the individual tools are merged in an automated fashion, which will on the one hand ignore simple naming differences between the tools, but will on the other hand highlight real annotational conflicts.

**Figure 2:**
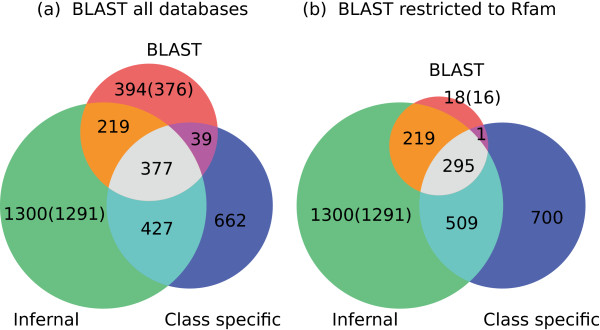
**Homology based annotation in overview.** The Venn diagrams for counting the high confident structured RNA loci found with sequence homology search (BLAST), structure homology search (Infernal) and class specific tools (tRNAscan-SE, snoStrip, and RNAmmer). **(a)** The diagram includes all 3,418 high confident structured RNA loci obtained by homology. **(b)** The similarity search is confined to the Rfam seed sequences (excluding the miRNA families in Rfam). 19 loci found by high confident BLAST against the Rfam sequences is missed by high confident structure homology search (18 + 1 in the red and purple areas of the right hand side of the figure). The reason is our additional Infernal E-value cutoff of 1e-3 imposed on all families. See text for detailed discussion. The numbers in parenthesis are after removal of 12 tRNAs and 15 miRNAs loci removed in the cleanup procedure. A total of 3,391 RNA loci were found. Of these 1,011 loci were found by sequence similarity, 2,314 were found by structure similarity, and 1,505 were detected by class specific methods.

We merged the high confident results of the individual tools to obtain the high confident annotation of structured RNAs (see Table 1 for the applied cutoffs). We found a total of 3,391 homology based high confident structured RNA loci and structural RNA elements after moving 12 tRNAs and 15 miRNAs to the medium confident annotation (Table 2). Of these 139 are cis-regulatory elements, 58 lncRNA loci (these are typically sub structures of the full length lncRNA), 3,183 (full length) ncRNA genes, and 11 conflicts of annotation. The distribution of the loci on those found by sequence similarity search, structure homology search and class specific tools is shown in Figure 2(a). A similar table with the homology based tools merged at the medium confident level and low confident level, respectively, is found in Additional file 1: Table S7.

**Table 2:** **Results of the homology based pipeline**

RNA class	High	
	Families	Loci
cisreg-elements	31	139
lncRNA-loci	58	58
miRNA	321	359
ribozyme	3	8
rRNA	5	185
snoRNA	211	638
snRNA	10	1,030
tRNA	51	810
Other	7	153
Conflict	9	11
Sum	706	3,391

The combined results of the sequence similarity search, structure homology search and class specific tools at the high confident cutoff level (See Table 1). The column *RNA class* contains cisreg-elements: cis-regulatory elements from Rfam/Infernal; lncRNA-loci: Infernal lncRNA structure loci; the next 7 rows contain (full length) ncRNA genes, miRNA: BLAST from miRBase and miRDeep predictions; ribozyme: ribozymes from Rfam/Infernal; rRNA: ribosomoal RNAs primarily from RNAmmer; snRNA and snoRNA: BLAST results and results from Infernal/Rfam; tRNA: tRNAs tRNAs from BLAST; tRNAscan-SE and Infernal/Rfam; lncRNA-loci: structural loci from larger genes(lncRNAs); other: RNA families from Rfam not belonging to one of the other classes; conflict: conflicts of annotation. Loci are the number of RNA loci of a given class; Families are a subdivision of classes into RNAs with the same name. 12 tRNAs and 15 miRNAs were moved to the medium confident annotation as part of the curation procedure. See text for details. Note that for the final high confident annotation we add 165 RNA-seq based miRNA candidates, reaching the total of 3,556 high confident RNA loci.

The high number of loci found by sequence similarity alone (red area in the figure) are primarily miRNAs found by similarity to miRNAs in miRBase, but miRNA families were excluded from the high confident structure similarity search. However, 19 loci found by sequence homology to sequences from Rfam were unconfirmed by structure homology. These are indicated by the red and purple areas in Figure 2(b). 15 of these 19 loci belong to two cis-regulatory families (SECIS_1 and IRE), 3 are tRNAs and 1 is a snoRNA belonging to the U3 family. In particular a cluster of 10 IRE loci located on chromosome 13 appear to be valid. 8 of these were confirmed by sequence similarity alone, however our imposed Infernal E-value cutoff of 1e-3 filter them out, while 2 were confirmed by both sequence and structure homology. The three tRNAs all fail the E-value filter of 1e-3, however, two of the tRNAs appear to be valid family members, both confirmed by low confident tRNAscan-SE. The third sequence might be incorrectly entered into the Rfam seeds with an Infernal E-value of 3.04e-02. The U3 locus may be an incorrectly curated sequence in Rfam with a score only half of the gathering score cutoff.

The substantial change between the two Venn diagrams (Figure 2) in the counts of the loci found by class specific tools and/or sequence and structure homology is caused by tRNAs found by BLAST against tRNAdb, but not found by BLAST against the tRNA seeds from the Rfam family. Amongst the 701 loci found by class specific tools alone were 115 rRNAs (16%), 135 snoRNAs (19%), and 451 tRNAs (64%). All but 21 rRNAs found by the class specific RNAmmer were recaptured by Infernal in the 5S rRNA family in the complete Infernal scan without any BLAST filtering and using the family specific gathering score as cutoff. However, the gathering score cutoff imply an Infernal E-value cutoff of ∼1000 for the 5S rRNA family. Similarly, 220 of the 451 tRNA candidates found by tRNAscan-SE but missed by high confident structure homology search were also found in the tRNA Rfam family in the complete Infernal scan. Here the gathering score cutoff implied an Infernal E-value cutoff of ∼30. Finally, 37 of the 135 snoRNAs found by snoStrip were also found in the complete Infernal scan. For the 5S ribosomal RNAs, tRNAs and for the snoRNAs detected by snoStrip, the difference in the loci detected by Infernal and by the class specific tools, indicates that sequence based homology search and additional test for class specific methods is a more optimal way of searching for these classes and genes. As a final remark, 1,127 of the 1,519 (74%) loci found by Infernal, but missed by the class specific tools, were marked as pseudogenes in the curation procedure (see the following sections).

Merging the results of the tools at the high confidence level introduced two kinds of inconsistencies. Firstly, naming differences due to inconsistent naming of the RNAs in different databases and by different tools. This type of inconsistencies were resolved by consistently preferring one tool over the others within each class of RNAs (See Additional file 1: Table S1). Secondly, the remaining inconsistencies will be genuine conflicts that needs to be resolved by hand, if possible. The conflicts within the high confident results are all between a miRNA and a non-miRNA. That is, some miRNA and one of the following snoRNAs: SNORA36, SNORA81, SCARNA4, SNORD59, SNORA53, SCARNA15, and SNORA18; Mir-1285 and Metazoa _SRP; and finally 28S rRNA and 4 different miRNAs.

This concludes the homology part of the annotation. Overall the homology part of the pipeline contributed 3,391 high confident structured RNAs (see Figure 2), after removal of 12 tRNAs and 15 miRNA loci in the cleanup procedure. The next section will deal with the small RNA-seq data, which will not only add confidence to the high confident miRNAs annotated above, but will also move a few medium or low confident miRNAs annotated by homology into the high confident annotation.

### Small RNA sequencing and novel miRNA predictions

Ten combined small RNA libraries were sequenced and scanned for miRNAs using miRDeep (See Methods section for details). The read profiles are available for download and online visualisation from http://rth.dk/resources/rnannotator/susscr102/version1.02.

The raw output from miRDeep indicated 467 putative miRNA loci with at least 10 reads present. However, due to duplication of genomic sequence in assembled chromosomes as well as unplaced scaffolds only 421 unique miRNA precursor sequences and 381 mature sequences were found. (Note: The 421 miRNA precursors were given provisional names, pre-1 …pre-421, pending correct names upon addition to miRBase). The cleanup procedure described in the Methods section reduced the number of sequences to 388 spanning 431 loci.

Of the 388 precursor sequences 174 are already known for pig in miRBase version 18, a further 69 could be identified by high confident sequence identity to miRNAs from other organisms in miRBase version 18. Finally 10 precursors without high confident annotations were identified using a combination of low-confident sequence identity (90% id), and synteny to other organisms.

The RNA annotations from the pipeline were checked for overlap with the small RNA library. We required a block of at least 10 reads overlapping with at least 20 nucleotides of the annotation and found overlap with 780 of the high confident annotations and 819 of the low confident annotations (308 and 329 of those were miRNAs at the respective annotation levels). See Additional file 1: Table S8 for a complete list of read supported high confident annotation.

The small RNA-seq data may have reads from ncRNAs yet to be discovered, beyond the miRNAs found by miRDeep. In the next section we present analysis with a recently developed tool, which may aid in finding new ncRNAs based on reads from small RNA-seq data.

### Annotation of de novo RNA transcripts using read profiles and deepBlockAlign

Small RNA sequencing data may contain transcripts, beyond miRNAs. To detect these *de novo* RNA transcripts, the closely spaced (<30 nt) set of mapped reads were grouped into distinct so-called *block groups* using blockbuster [33] and the resulting block group structures were compared by their read profiles using deepBlockAlign [34] to obtain putative RNA annotations of the unknown transcripts. See Methods section for details.

From blockbuster we found 1,127 block groups with a length of at least 50 nt, of which 541 overlapped with homology based annotations. Out of the 586 unannotated block groups 40 overlapped with miRDeep predicted novel miRNAs. By comparing (aligning) the read profiles of the annotated and unannotated RNA transcripts using deepBlockAlign we were able to give putative annotations to 165 block groups having no high confident homology annotation. The results are summarized in Table 3.

**Table 3:** **Annotation of unannotated block groups**

	miRDeep-unannotated	miRDeep-miRNA	Sum
dba-unannotated	417	4	421
dba-miRNA	46	31	77
dba-rRNA	1	0	1
dba-snoRNA	37	5	42
dba-snRNA	6	0	6
dba-tRNA	39	0	39
dba-annotated	129	36	165
Sum	546	40	586

The transcripts from the small RNA study without annotation by the homology based pipeline were analysed with blockbuster resulting in 586 transcripts (block-groups). The table displays a comparison of the *de novo* annotation of these block-groups by deepBlockAlign and with miRDeep. The table shows a comparison of the deepBlockAlign and miRDeep annotation of the 586 unannotated block groups. The second column contains the 546 block groups not annotated by miRDeep, the third column the 40 block groups annotated by miRDeep and the fourth is the sum of the two previous columns, *i.e.* all 586 block groups. The rows contain the deepBlockAlign annotations: the second row contains the block groups without deepBlockAlign(dba) annotation, rows 3–7 contains the deepBlockAlign classifications, row 8 is the sum of rows 3–7, and finally row 9 is the sum of rows 2 and 8, that is all 586 block groups and depending on the column, their miRDeep annotation.

deepBlockAlign and miRDeep share 36 predictions and in most (31) of the cases deepBlockAlign agrees with the miRNA assignments of the loci, however, 5 predictions made by miRDeep had read profiles that according to deepBlockAlign were most similar to snoRNAs and 4 had no matching profiles at the deepBlockAlign score cutoff of 0.6 [35]. These nine profiles are shown in Additional file 1: Figure S1 and Figure S2. In general, the miRDeep predictions with read profiles that align poorly with read profiles of known miRNAs, either have unusually long or short loop regions or have read profiles different from the two peaks normally observed for miRNAs.

Five interesting examples among the unannotated read profiles not overlapping with miRDeep predictions can be found by requiring both a deepBlockAlign classification as well as an overlap with a structurally conserved region as predicted by RNAz. These five examples are displayed in Additional file 1: Figure S3–S5. And amongst these examples we find 2 miRNAs that are missed by both high confident BLAST and miRDeep, but deepBlockAlign identifies the read profile as miRNA like, and low confident BLAST identifies the miRNAs as mir-223 and mir-431. One further miRNA, mir-1388, is identified by low confident BLAST and by deepBlockAlign, but is not overlapping with an RNAz loci (Additional file 1: Figure S6).

The examples presented above show how deepBlockAlign present an experimentalist with extra information on top of conventional analysis of small RNA-seq data. Both the 10 putative miRNAs with uncertain miRNA profiles, and the 3 miRNAs missed by both BLAST and miRDeep require additional experimental scrutiny, which, however, is outside the scope of this work.

### A multiple alignment of pig and 20 other vertebrate genomes

This and the following sections are concerned with adding context to the annotation provided above. We begin with a synteny analysis of the pig genome and of the structured RNA annotation.

**Figure 3:**
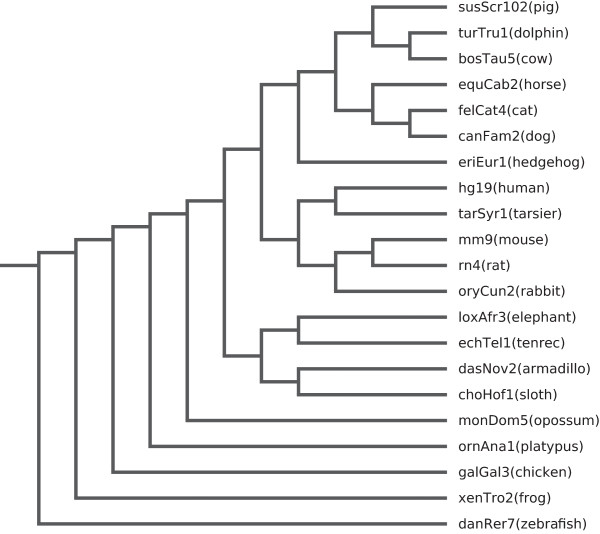
**Phyologentic tree.** Phylogenetic tree for the pig genome multiple alignments. This phylogenetic tree is derived from the human phylogenetic tree from UCSC based on the 46-way alignment. The organisms has been reordered to put pig on top and the branch lengths have been ignored. A tree in this form is needed as parameter for the TBA/MultiZ program. The tree is without branch lengths since these have not been recalculated for the pig genome.

**Figure 4:**
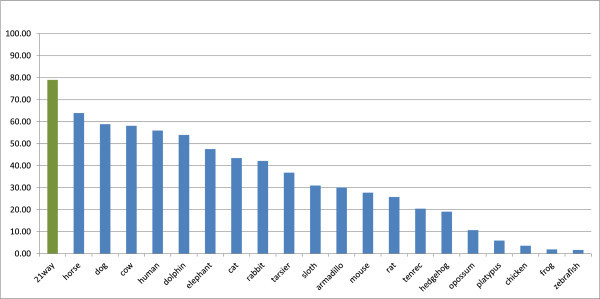
**Coverage of the pig genome.** Absolute coverage of the pig genome by the genomes used for the multiple alignment. The coverage is based on the cleaned alignments with single best coverage of the pig genome. However, depending on the method of cleaning the coverage of the target genomes (x-axis) may be multiple in some locations. Far left, in green, is the result of the coverage of the pig genome by any genome in the multiple alignments.

To investigate synteny between pig and 20 other vertebrate genomes, we formed LASTZ/UCSC [29, 30] pairwise alignments, which were subsequently cleaned for non-syntenic alignments. The resulting coverage of the pig genome ranged from around 1.7% for zebrafish to 64% for horse measured on the coverage of the non-Ns of the pig genome (the N’s currently account for 10% of the 2.8 Giga bases in the assembly). A multiple alignment was formed from the pairwise alignments with the Threaded-Blockset-Aligner (TBA/MultiZ) [36] using the phylogenetic tree presented in Figure 3 as guide. This pig-centered multiple alignment featured a coverage of 79% of the pig genome (Table 4 and Figure 4). Note that the combined pipeline of TBA/MultiZ and LASTZ is also known as multiZ.

**Table 4:** **Pairwise alignments**

Genome	Species	LASTZ/chaining options	Coverage	Alignment type
danRer7	Zebrafish	Distant	1.69	rbest
xenTro2	Frog	Distant	1.94	rbest
galGal3	Chicken	Distant	3.61	rbest
ornAna1	Platypus	Distant	5.97	rbest
monDom5	Opossum	Distant	10.70	syntenic
eriEur1	Hedgehog	Close	19.08	rbest
echTel1	Tenrec	Close	20.43	rbest
rn4	Rat	Close	25.76	syntenic
mm9	Mouse	Close	27.71	syntenic
dasNov2	Armadillo	Close	30.10	rbest
choHof1	Sloth	Close	30.94	rbest
tarSyr1	Tarsier	Close	36.80	rbest
oryCun2	Rabbit	Close	42.11	syntenic
felCat4	Cat	Close	43.42	rbest
loxAfr3	Elephant	Close	47.52	syntenic
turTru1	Dolphin	Close	53.93	rbest
hg19	Human	Close	55.93	syntenic
bosTau5	Cow	Close	58.13	syntenic
canFam2	Dog	Close	58.83	syntenic
equCab2	Horse	Close	63.90	syntenic
21way	Multiple alignment			78.98

Pairwise and multiple alignments of the pig genome. The other genomes were obtained from the UCSC genome browser website in their lower-case masked form. Masking was performed by the UCSC with RepeatMasker and Tandem repeat masker. The UCSC genome designation is given in the first column. The options for the pairwise alignments are given in the third column as either *closely* or *distantly* related to the pig in accordance with the choices made by UCSC for the human genome. The distance to pig has implications for the LASTZ and axtChain options as listed in Additional file 1: Table S19. The coverage of the pig genome is given in % in the fourth column based on the number of non-Ns covered by the pairwise alignment after cleaning of the alignments as specified. The pairwise alignments are cleaned either by synteny (small alignment chains are deleted when they would otherwise break synteny) or by deleting all but the best alignments where the target genome is multiply-covered. Both methods reduces the coverage of the pig genome by the alignment. The choice of cleanup method is given in the last column. A graphical representation of the coverage is given in Figure 4.

The coverage of the pig genome featured in Table 4 and Figure 4 is a less than ideal measure of the phylogenetic distance between the pig (target) and the other species (query). Ideally the distance is measured on the sequence change within a set of known and carefully selected genes. The coverage is influenced not only by varying rate of sequence change depending on the genomic loci but also on the different alignment parameters used for different genomes (that is, the more sensitive parameters used for the more distant genomes will result in a artificially high coverage), on the size of the completed genomes, and also on the degree of completion of the genomes.

The multiple alignment obtained in this section will be further used to find structurally conserved RNAs using RNAz. An application of the pairwise alignments is the synteny analysis of the structured RNA annotation in pig. However, to fully utilize the alignments we need a good annotation of structured RNAs in the query organism. The best annotated organism among the vertebrates is human and we therefore proceed with a more detailed analysis of the synteny between human and pig.

### Human and pig synteny analysis

We performed a detailed synteny analysis of the relationship between the pig and human genomes based on the pairwise alignment between the two. The analysis lead to blocks at least 5,000 nt long in which the synteny and strand information of the pairwise alignments was unbroken. These blocks, referred to as *synteny blocks*, were broken at gaps larger than 5,000nt. We identified 40,781 such synteny blocks between human and pig, which covered 79% of the non-Ns of the assembled pig chromosomes. The syntenic blocks contain gaps not covered by alignments, and the coverage is therefore larger than the raw coverage of 56% of the pig genome by the human genome as reported in Table 4. 3,242(97%) of the 3,556 high confident annotations were found on the assembled chromosomes and of these 78% are in the human syntenic blocks (See Table 5).

**Table 5:** **Synteny of the RNAs of the homology based pipeline**

RNA class	# loci	hg19			RNAs conserved in N other organisms		
		Syntenic	Conserved	Both	1	5	15
cisreg-elements	139	80	84	65	116	86	31
lncRNA-loci	58	57	53	53	58	57	7
miRNA	369	303	349	292	360	349	102
putative-miRNA	155	121	25	20	65	25	1
ribozyme	8	8	3	3	3	3	0
rRNA	185	143	0	0	6	1	0
snoRNA	638	473	266	221	400	282	43
snRNA	1,030	674	24	13	119	26	4
tRNA	810	549	274	199	389	284	14
other	153	111	10	10	17	11	0
Conflict	11	10	10	10	10	10	4
Sum	3,556	2,529	1,098	886	1,543	1,134	206

The columns are, *RNA class*, *# RNA loci*. *# loci in human syntenic blocks*, *# loci conserved in human* by 80% sequence identity. *# loci both syntenic and conserved* that is the number of ncRNAs in syntenic blocks where the ncRNA is actually conserved in human. *# loci RNAs conserved in N other organisms*, grouped by number of loci conserved in at least 1, 5, or 15 other organisms. Conservation is determined by the sequence identity in the pairwise alignments. The RNA loci are located in the pairwise alignments and the sequence identity is calculated when at least 80% of an RNA locus is covered. The RNA locus is counted as conserved in that organism if the locus has a sequence identity of at least 80%. In the table the number of RNAs conserved in at least N (N=1; N=5 or N=15) of the other genomes: bosTau5, canFam2, choHof1, danRer7, dasNov2, echTel1, equCab2, eriEur1, felCat4, galGal3, hg19, loxAfr3, mm9, monDom5, ornAna1, rn4, oryCun2, tarSyr1, turTru1, xenTro2.

When we merged neighbouring synteny blocks with identical chromosome and strand information, we found that the number of blocks could only be reduced to 13,302. If we instead ignored the strand information and merged neighbouring synteny blocks based only on a requirement of identical chromosome information, the number of blocks could be reduced to 218 synteny regions between human and pig, which cover 99% of the pig assembly (note that we allow large gaps, inversions and even to some extend rearrangements within these regions). The number of synteny regions found here, may be compared to the 51 synteny groups and 173 conserved segments found in the experimentally based human pig comparative map in reference [37].

When we did a similar analysis for the horse/human pairwise alignment we found the initial 27,219 initial synteny blocks could be reduced to 1,786 synteny regions when we ignored the gaps but respected the strand, and finally to 203 synteny blocks when gaps and strand were both ignored. These cover 99% of the horse genome. The difference lies in the assembly of the two genomes. The horse contigs are ordered and oriented correctly using a combination of probe sequences and positional information from the comprehensive radiation hybrid and FISH maps [38]. Notice that the 218 regions represent a pig-centered view of the pig-human synteny regions. The corresponding regions in human may contain (large) gaps and in certain cases overlap. A cleaned up version consisting of 377 smaller regions is found on the webpage ( http://rth.dk/resources/rnannotator/susscr102/version1.02/regions.html). In this version large gaps in the human regions are ignored, but in some cases small regions will overlap with large regions when viewed in the human coordinates.

The analysis of the synteny between human and pig highlights some issues in the current pig assembly, which will be touched further upon in the following sections. These issues however, only makes a detailed synteny analysis more desirable, since it enables the identification of some of these issues.

### Annotations having duplicated sequence

A particular issue in the current assembly is duplicated sequence, both within the assembled chromosomes, and between sequence on the assembled and unplaced scaffolds.

We found that annotations expected only once in a mammalian genome were found in more than one place in the pig genome with the exact same sequence. Sometimes these duplicated annotations were found twice on the same chromosome in close proximity (for example mir-196b twice on the same strand, and both mir-615 and mir-194 twice, once on each strand) and sometimes they were found both on the assembled chromosomes and within the unplaced contigs (for example mir-127, mir-155). Furthermore, sequence from the unplaced scaffolds are in some cases largely identical to sequence placed on the assembled chromosomes. To investigate this problem we formed a pairwise alignment of the pig genome with itself using Blat [30] requiring high sequence identity (>= 98%) and allowing no introns in the alignments. The result of the alignment of the assembled chromosomes against unplaced scaffolds were that 63% of the sequence of the unplaced scaffolds were aligned to sequence on the chromosomes. The self alignment also allowed the identification of a number of annotations that were duplicated in the pig genome. Some of these are known to have many copies in mammalian genomes, like 7SK, 8S rRNA, GP_knot1, Histone-3-prime-UTR, SNORA70, SNORD116, tRNAs, U1, and U6. However, others like the miRNAs listed above were not expected to be found in multiple copies with high sequence identity. A module was therefore added to the pipeline which identifies annotations with identical sequences. We found that 10% high confident miRNA genes had exact duplicates in the assembled chromosome or unplaced scaffolds. A full list of duplicated annotations is found in Additional file 1: Table S9. Note that, all copies of duplicated genes are used in the remaining analysis as it raises problems with the statistics when the genomic context is different between two copies. For example one copy may be conserved in another organism, while the other is not.

### General synteny analysis of the pig annotation

To further enhance the annotation we performed a general analysis of the conservation of the structured RNA loci. The full benefit of this analysis is only obtained when we have a good annotation of structured RNAs in the query organism. Special emphasis is therefore put on the pig/human conservation of the annotation.

We analyzed the synteny of the structured RNA annotation using the pairwise alignments is to check the coverage of a given annotation by transferring the coordinates to the target species using the pairwise alignment (liftOver). However, since the alignments are single best coverage of pig, but may be multiple coverage of the target genome in some region, the annotation will in some cases be matched with a paralogous sequence in the target genome. This problem is to some degree mitigated by using the cleaned-up pairwise alignments described above, which should reduce the coverage of paralogous alignments.

To annotate the RNAs in the non-pig organisms, each annotation is located in the pairwise alignment and the per-base identity is calculated. In Table 5 we list the loci that are conserved in human (left side of the table) and in depth in the phylogenetic tree (right side of the table). We have chosen 80% sequence identity as a cutoff for conservation between pig and other organisms. This choice is based on our experiences with the conservation of miRNAs between pig and human (see Section miRNAs in the pig genome for details). In general, deciding on such a cutoff is difficult if not impossible, and should depend on the class of the RNA annotation as well as the phylogenetic distance between the two organisms.

### Genic context of the annotations

Another important characteristic of the ncRNA annotations is their contexts of protein coding genes. We therefore marked the annotations with genic context as either UTR, coding-exonic or intronic using the coding genes from Ensembl version 68. Annotations overlapping a coding gene were marked as having a) coding-exon context if at least 50% of the annotation overlapped with a coding exon or b) 5’ or 3’ UTR context if the annotation overlapped with a non-coding exon. If an exon was marked as both coding and non-coding for different transcripts, the coding-exonic context was preferred. When an annotation is close to more than one protein coding gene all genic context designations are kept. We also look for proteins coding genes 10,000 nucleotides up or down stream of the annotation (similar cutoff as the miRNA genic contexts used by miRBase). A summary of the genic contexts for the different RNA classes are shown in Additional file 1: Table S10.

Note that the protein annotation of the pig genome is at an early stage and is further made difficult by incorrect ordering and strand assignment of the contigs of the pig genome hinted at in the section about the human/pig pairwise alignment. Also the UTRs of are not always well-defined or even known. Therefore the assignment of context as coding-exonic, UTR, or intronic will in some cases be immature or even incorrect.

We will touch upon the genic context again when analyzing conservation of contexts for particular classes of structured RNAs, *e.g.* cis-regulatory elements and snoRNAs.

### Clustering of the annotations

Certain structured RNAs are known to form clusters based on genomic position. Examples include, miRNA clusters, snoRNAs spliced from itrons, clusters of tRNAs and clusters of cis-regulatory elements, *e.g.* iron response elements (IRE).

All pairs of high confident annotations less than 10,000 nt apart on the same strand, were used to form clusters by single linkage. The distribution of the cluster size is shown in Additional file 1: Figure S7. The cutoff of 10,000 was chosen in accordance with the cutoff used by miRBase for the clustering of miRNAs. We found 261 clusters of which 10 contained at least three miRNAs. A number of large snoRNA clusters were also observed. Detailed examples of the clusters are given in later sections for miRNAs, and snoRNAs. In Additional file 1: Table S11 the number of RNAs in different classes and different clustering distances is shown.

### *De novo*prediction of structurally conserved RNAs

When novel structural RNAs in *e.g.*, UTRs are predicted by computational methods or by experiments, structural conservation may help the experimentalist in asserting their validity and phylogenetic domain. We therefore predicted *de novo* structured RNAs in the pig genome using RNAz and the MAF blocks of the multiple alignment of pig against 20 other vertebrate genomes. Details are in the methods section, including a discussion of false positive rates of RNAz.

We found 95,106 strand specific structurally conserved loci with a p-score cutoff of 0.9, which could be merged to 83,869 conserved loci when the strand prediction of RNAz was ignored. Additional file 1: Table S12 shows the overall statistics of the RNAz hits. The RNAz predictions were assigned genic contexts according to the same criteria as for the homology based annotations. Most of the RNAz predictions were found in intergenic regions (59%) or were intronic (23%); however a small number are found to be overlapping with exons of protein coding genes (1%) or near the beginning or the end of protein coding transcripts (5%) and (6%) respectively, 7% lie in the context of multiple genes, and their gene context is undecided.

The annotations of the pipeline were marked as structurally conserved, if there was an overlap of at least 20 nt to an RNAz loci irrespective of strand. The results are show in Table 6. 538 of the high confident annotations were found to overlap with RNAz loci and as expected the highly structured miRNA has the highest recovery rate in the RNAz results. It is, however, somewhat lower than one might have expected when Table 6 is compared to Table 5, which shows the statistics of RNAs conserved in genomic location. For example, based on the pairwise alignments 90% of the high confident miRNA candidates obtained by sequence homology are conserved in genomic position in at least 6 organisms including pig, which is included in the pig-centered alignments by default. However, RNAz only predicts 62% to be structurally conserved. This is not necessarily a flaw in RNAz, but more likely in the multiple alignment MAF blocks. For example, mir-30e is found to be conserved in 17 of the 21 species but is not confirmed with RNAz. Examination of the sequences shows that mir-30e is partially sequenced in the tenrec genome, which breaks the multiple alignment MAF block and thereby prohibits the RNAz prediction. Repairing the MAF blocks on a genome wide scale would in principle be possible. It is however, not straightforward and is outside of the scope of the present study.

**Table 6:** **Overlap of the** **RNAz** **predicted** ***de novo*** **with the high confident annotation**

RNA class	Annotation		RNAz overlap	
	Families	Loci	Families	Loci
cisreg-elements	31	139	4	10
lncRNA-loci	58	58	3	3
miRNA	330	369	222	241
putative-miRNA	135	155	14	16
ribozyme	3	8	0	0
rRNA	5	185	2	2
snoRNA	211	638	57	72
snRNA	10	1,030	9	20
tRNA	51	810	36	154
other	7	153	3	4
conflict	9	11	4	6
sum	850	3,556	354	528

Comparison of the strand specific RNAz results with the result of the automatic annotation pipeline. The columns are, *RNA class*, *# RNA families* in the high confident annotation, *# RNA loci* in the high confident annotation, *# RNA families* that overlap with the RNAz predictions, *# RNA loci* that overlap with the RNAz predictions.

As a final remark, it is noteworthy that while around 65% of the miRNAs obtained by similarity search are overlapping with RNAz loci, the same is only true for around 10% of the novel miRNAs. 16% are conserved in 6 organisms and 10 or 6% appear to be conserved in human and may thus represent miRNAs in human yet to be discovered.

### Lineage specific structured RNA loci

The lineage specific structured RNAs are important for the pig, both for the understanding of pig specific traits and when considering the pig as model organism as these can help pointing towards potential concerns in some pathway modelling.. We therefore investigated the lineage specificity of structured RNAs inside and outside of the Laurasiatheria (which contains pig, cow, dolphin, horse, cat, dog, hedgehog amongst the genomes investigated in the pairwise alignments).

The bulk part of the structured RNAs obtained here were found by homology search, and will therefore not be pig specific. However, the *de novo* RNAs may be lineage specific and both homology and *de novo* based annotations may be found in lineage specific loci, that is loci preserved only in a branch of the phylogenetic tree. Our search will therefore initially focus upon lineage specific regions in the pig genome.

In general, determining lineage specific RNAs is non-trivial since it require clear annotation in one or several closely related organisms, however, at some cutoff the annotation is not to be found in organisms outside the organism or the closely related organisms. Furthermore, determining the lineage specific annotations might be impacted by the lack of genomic coverage and the quality of the genomic sequences, as it is the case between human and Neanderthal [39]. The search for lineage specific structured RNA is further complicated by the fact that many structured RNAs can hold the same structure while being highly divergent in sequence. Structural (re-)alignment of genomic sequence is in these cases required to elucidate the structural conservation *e.g.*, [40–44]. Hence, we employ a conservative strategy where the sequences within the Laurasiatherian branch should exhibit relatively high sequence identity (>60%) between all pairs of organisms considered while at the same time have relatively low sequence identity (<30%) to all organisms outside the branch.

To find lineage specific structured RNAs, we calculated the sequence identity of the obtained structured RNAs between pig and the organism for which we have pairwise alignments, preferably, using the synteny cleaned pairwise alignments where available. The RNAz results are particularly well suited for this analysis, since structural conservation in at least 3 organisms including pig is required to yield a result in the first place. In concordance with our strategy, we need to find a lower cutoff in sequence identity for the loci to be not considered with the organisms in question.

In Additional file 1: Figure S8 we plotted the number of RNAz loci grouped by a given identity cutoff outside the Laurasiatherian branch (these are the lineage specific candidates). Up until 30% sequence identity cutoff the number of grouped loci remains fairly constant (and relatively small), but from around 50% quickly increase. We therefore choose a conservative sequence identity cutoff of 30% outside the lineage, which corresponds to around 1.5% of the RNAz loci.

In Additional file 1: Figure S9 we plot the number of RNAz loci that have at most 30% sequence identity to pig outside the lineage versus the minimum pairwise sequence identity between pig and the other species found in the pairwise alignment. The number quickly drops above 60% sequence identity and we therefore decide on a cutoff of 60% and reach a final number of 1,004 lineage specific loci containing RNAz predictions.

The high confident annotation was investigated for lineage specificity by the same criteria, however, only the annotations with at most 5 occurrences in the genome were investigated, as highly repetitive sequences are often skipped in the alignments. Furthermore, annotations with exact duplicates in the genome and annotations found in the unplaced scaffolds were excluded as well. With these criteria, we found 23 lineage or pig specific structured RNA loci on the assembled chromosomes within the high confident annotation shown in Additional file 1: Table S13. These are miRNAs mir-1949 and mir-2320, four *de novo* miRNAs, 16 snoRNAs and one vault RNA. Only four of these are conserved in other species within the lineage, three HACA box snoRNAs (SNORA22, SNORA23, SNORA81) and one known miRNA (mir-2320), specific for the cow/pig/dolphin part of the tree. The vault RNA found on chromosome X is likely a pseudogene since it lacks a polII promoter sequence. It does, however, score very high according to Infernal (69 compared to 82 for the real gene on chromosome 2). For further discussion about the vault RNAs see the Section Curation based on the detection of PolII and PolIII promoter sequences. One miRNA, mir-1949, is previously known only for rodents, and could also be a mis-annotation induced by the otherwise strict filtering of the BLAST results.

Lineage specific ncRNAs is still a relatively unexplored area, which is expected to grow as more analysis is performed on new genomes and organisms. MiRNAs are one class of ncRNAs where lineage specific ncRNAs have been discovered in pig, but more classes are expected to follow when the tools for novel ncRNA detection in small RNA-seq data are developed further.

### miRNAs in the pig genome

In this and the following sections we discuss the curation of the structured RNA annotation utilizing the different types of contexts added to the annotation in the preceding sections. We begin with the miRNAs in pig and the synteny between pig and human miRNAs, which in conclusion will allow us to curate the 125 miRNA genes that were found to be part of miRNA clusters common to human and pig.

The high confident homology pipeline provided 359 miRNA loci and the miRDeep pipeline adds an additional 165 *de novo* miRNA loci for a total of 524 miRNA loci. 10 of the miRDeep loci where renamed and reclassified by homology to human or by low confident (90% id) sequence identity to sequences from miRBase. See also Additional file 2.

MiRNA clusters are known for a number of organisms. In the analysis of the pig genome we observed 58 miRNA clusters, *i.e.* multiple miRNA loci within 10,000 nucleotides of each other, all on the same strand. In certain cases, miRNAs in clusters are found like *pearls on a string*. In the pig genome we found 10 miRNA clusters with at least three miRNAs. 6 had a cluster size of up to around 300 nucleotides per miRNA, while the last 4 had sizes of approximately 1,000-2,000 nucleotides per miRNA.

In comparison to the 58 pig miRNA clusters, a total of 89 of strand specific miRNA clusters are known in the human genome according to miRBase version 19. In the following, we have analyzed how many of these miRNA clusters were conserved in human using a combination of BLAST, and the genome scale pairwise alignments between the two organisms.

MiRNAs are often well conserved between mammalian genomes, however, a simple BLAST of the miRNAs will either include too many false positives or occlude the more distant homologs. In Additional file 1: Figure S10 we have plotted the conservation in pig of the 1,595 human miRNA loci from miRBase 19 using the genome scale human-pig pairwise alignment. From the figure we conclude that there is no clear indication of a identity cutoff where a miRNA may be said to be conserved between human and pig. However, if we restrict the plot to the miRNAs from human miRNA clusters (Additional file 1: Figure S11) a steep drop-off in the number of conserved miRNAs is observed at around 80–90% identity. Throughout the paper, we will use a cutoff of 80% sequence identity in the pairwise alignments to accept conservation between pig and human. This results in a total of 168 miRNAs from the human miRNA clusters that are considered conserved in pig. These form 51 clusters in pig, which again contains 151 miRNAs, *i.e.*, 17 miRNAs are conserved, but no longer a part of a miRNA cluster in pig at this cutoff level.

The 13 miRNA clusters from human with at least 4 miRNAs are shown in Additional file 1: Table S14. In the following, we discuss the conservation of these clusters in pig.

The mir-512 cluster is known to be primate specific according to miRBase, and we see no evidence to the contrary. The mir-379 cluster is incomplete in pig (Additional file 1: Figure S12), where it is found in an unplaced scaffold in pig missing part of the sequence. A similar issue is seen with the mir-450 cluster. The mir-493 cluster appear to be broken in pig. Most of the miRNAs are found in a cluster on chromosome 7, however they are located on both strands (Additional file 1: Figure S13). The cow, dolphin and horse genomes, which are phylogentically close to the pig genome, all harbors near complete mir-493 clusters, so this is likely an assembly issue in pig.

A more difficult case is the mir-532 cluster located on chrX in human (Additional file 1: Figure S14), which contains 8 known miRNAs from miRBase: mir-532, mir-188, mir-500a, mir-362, mir-501, mir-500b, mir-660, mir-502. In pig we found a cluster on chrX of mir-532, mir-188, mir-500, mir-362, mir-500, mir-660, and a mature unknown miRNA picked up by miRDeep. Investigation of the human-pig alignment revealed that mir-501 is poorly conserved with a pairwise identity of 57%, while the unknown miRNA picked up by miRDeep is mir-502 with a pairwise identity to human of 87%. Mir-501 is also poorly conserved in cow (65%), but somewhat better in horse (78%).

The mir-17 and mir-363 clusters are well conserved and supported by reads (Additional file 1: Figure S15 and S16). In the mir-367 cluster only 3 of the 5 miRNAs are identified by high confident BLAST, however, the cluster appears complete in the pairwise alignment (Additional file 1: Figure S17).

The mir-513a and mir-514b clusters are incomplete in the pig genome (See Additional file 1: Figure S18), leading to several problems in the pairwise alignments. Three miRNAs are observed in the miRDeep analysis of these clusters, which may be assigned as mir-506, mir-508 and likely mir-509.

The mir-450b cluster is incomplete in the assembly, but the miRNAs that we find are supported by BLAST and reads (Additional file 1: Figure S19). mir-3601 found on the opposite strand of the cluster is obtained by sequence similarity from cow.

The cluster containing mir-892c appear at first glance to be missing in pig. However, the six miRNAs in the mir-892 cluster on chrX in human, are conserved in a cluster on chrX in pig (See Additional file 1: Figure S20). The pairwise identities between the human and pig miRNAs in this cluster are in the 55–70% range and are thus under the cutoff chosen above. The mir-891a/b are two different loci in human but only one of them is observed in the pairwise alignments in the pig. The cluster is supported in the pig by 6 *de novo* structured RNA predictions by RNAz.

In conclusion, we include the 125 miRNA loci in the curation annotation, for which we have high confident annotation and which are are part of miRNA clusters in both pig and human.

### snoRNAs in the pig genome

The conservation and curation of snoRNAs in pig are discussed in this section. Using synteny between pig and human we curated 268 or 42% of the snoRNAs loci in pig.

We annotated 645 snoRNA loci (including 7 conflicting loci) in the pig genome using a combination of BLAST, snoStrip and Infernal on snoRNA families from Rfam.

Like miRNAs, snoRNAs are often found in clusters and we identified 73 clusters containing multiple snoRNAs within a distance 10,000 nt. In two particular cases large snoRNA clusters identify snoRNA host genes in pig, which are at present missing from the Ensembl annotation. In human these two transcripts, called SNHG1 and GAS5, are known to harbour a number of snoRNA. The human version of SNHG1 harbours SNORD25, SNORD26, SNORD27, SNORD28, SNORD22, SNORD29, SNORD30, SNORD31, and the same genes in the same order are observed in pig. GAS5, similarly displays exactly the same genes in human and pig. The 23 snoRNAs contained in these two host genes were curated based on this information.

The snoRNA database contains a set of curated human snoRNAs. Using the syntenic pairwise alignments between human and pig, we manually inspected the auto generated snoRNA annotations. We obtained the human coordinates for the pig snoRNAs and calculated the coverage using the methods explained in the synteny section above. We filtered the results by 60% sequence identity between human and pig and considered the coordinates of the curated human snoRNAs from the snoRNA database. In total we curated 268 loci, corresponding to 189 snoRNA families. The 278 non-curated members of these families were marked as pseudogenes. While the cutoff for sequence identity is 60%, all curated members were found to have a sequence identity above 70% between pig and human. It is noteworthy that only 64 (23%) of the curated snoRNAs are found with high confident BLAST.

This kind of synteny analysis limit ourselves to known genes in other organisms, here human. As an example we find 8 copies of SNORD14 in pig. A closer inspection, however, reveals that 3 of them have exact copies close to each other. These copies are likely to be the result of incorrect assembly. We thus find two SNORD14 clusters in pig, one with two members and one with three members, all scoring about equally well with Infernal. In human we find only two curated copies in the snoRNA database, which we are able to match and curate in pig using synteny. However, a full Infernal search reveals a total of 5 copies in human all scoring about equally well. And these form two clusters with 2 and 3 members, respectively, each cluster syntenic to those in pig.

In summary, we curated 42% of the snoRNAs in pig. However, it is expected that more snoRNA genes may be curated as the snoRNA discovery methods improve.

### Ribosomal RNA clusters in the pig genome

The ribosomal RNAs (rRNAs) are known to have a number of pseudogenes in the mammalian genomes, often only as part of the full length rRNA genes. For the rRNAs we only found one full length copy of the rRNA cluster. On chromosome 6 we found 28S, 18S, and 5.8S clustered closely together, and the finding is further confirmed by the existence of a pRNA locus upstream of the 28S locus on chromosome 6. Ribosomal RNAs part of this cluster are curated, all other rRNAs are marked as pseudogenes.

### Cis-regulatory elements in the pig genome

The cis-regulatory elements are regions of the RNA (and DNA) that regulate genes on the same chromosome, often on the same transcript or located relatively close up- or down-stream. Here, we only annotate those cis-regulatory elements that have RNA structure as extracted from Rfam. The genic context of the annotated cis-regulatory elements allowed us to curate 66% of them. For the remaining 34% the genic context could not be ascertained and the cis regulatory elements were marked as pseudogenes.

Normally close proximity between the cis-regulatory element and the gene is observed. Accordingly, the genic context of the cis-regulatory elements are of particular interest. As expected, we found that most of the annotated cis-regulatory elements are close to or overlapping with protein coding genes. Only 31 of the total 139 cis-regulatory elements are more than 10,000 nucleotides away from a protein coding gene, while 12 are between 1,000 and 10,000 nucleotides away. Of these 12 cis-regulatory elements 9 belong to the ubiquitous GP_Knot1 family. 1,000 nucleotides is therefore chosen as the cutoff for the curation procedure of the cis-regulatory elements. Of the 139 annotated cis-regulatory elements, 20 cis-regulatory elements are found to be antisense to protein coding genes. However closer inspection reveal all but two to be sense to a protein coding gene as well. Many of the intergenic cis-regulatory elements are found to be from highly repetitive families like GP_Knot1 and IRE, while others are very confident and highly specific like *e.g.*, SECIS-1 and IRES-c-sis. Only detailed analysis can reveal if these are true positives where the corresponding protein coding gene is undetected by for what ever reason.

In all 93 (63%) cis-regulatory-element loci were curated based on having sense genic context within 1,000 nucleotides. 31 cis-regulatory elements more than 10,00 nucleotides away from a protein were marked as pseudogenes.

As a final remark, the annotation of protein coding genes in the pig genome is at an early stage and is further complicated by the incorrect ordering and strand of the pig genome contigs. Therefore, we are unable to curate the cis-regulatory elements according to their exact genic contexts (coding-exonic, UTR or intronic), and just note in passing that certain elements are expected to have certain contexts, *e.g.*, IREs are supposed to be part of the UTRs, and we were not always able to confirm this.

### Curation based on the detection of PolII and PolIII promoter sequences

In the preceding sections we presented the curation of specific classes of structured RNAs. We now present a more general class of ncRNAs, namely those where active genes are expected to have PolII or PolIII promoter sequences. This class includes ncRNAs such as snRNAs, vault RNA, RNaseMRP, and Y RNAs.

A number of ncRNAs may be curated based on the presence of PolII/PolIII promoter sequences. For this we used Position Weight Matrices (PWM) extracted from human sequences to detect PolII- and PolIII-specific sequence elements derived from known human PolII and PolIII transcripts (see Additional file 1: Table S21–S26 and Methods section for details). Based on the PolIII-PWM, a total of 19 out of the 800 U6 candidates returned by the Infernal/Rfam search, were selected. Among these 5 are found to be well conserved in the same genomic location in other species, and 5 are located in introns. Single instances of U6atac, RNaseMRP, RNaseP_nuc were similarly found. Finally even though two 7SK were found to have satisfactory TATA-boxes and Proximal Sequence Elements (PSE), only one sequence was highly similar to other mammalian 7SK sequences.

For snRNAs, 17 U1, 14 U2, 3 U3, 6 U4, 7 U5, 2 U7, 2 U8, 1 U11, 1 U12 and 2 U13 were found to have PolII promoters sequences. In case of U4atac, the PSEB and PSEA segments were spatially inverted, i.e. PSEB was located upstream of PSEA. Still, due to the high conservation of U4atac in all other mammals, U4atac was kept in the annotation.

The structure based homology search picks up 3 vault RNAs. Two copies on chromosome 2 where one is an active copy with a degraded copy nearby conserved in pig, cattle, and dolphin (in pig it is not even part of the low confident annotation, but found in the complete set of BLAST supported Infernal/Rfam results with an Infernal E-value of around 0.2). It appears to be a common thread amongst the species within the Laurasiatherian lineage that only one good copy of the vault RNA gene exists within the vault RNA cluster, unlike the one in human which feature 4 good copies (chr2 in pig, chr14 in horse, chr7 in cow, scaffold111177 in dolphin, chr11 in dog, chrA1 in cat, and chr5 in human). A high scoring pseudogene is found on chromosome X, in pig, as well as human. A lower scoring vault RNA is found on chromosome X in cow, and possibly in dolphin, however in the latter the genome is not assembled on chromosomes. Note that these pseudogenic copies on chromosome X cannot be related through the genome wise pairwise alignments.

Y RNAs are Pol-III transcripts with the canonical copies transcribed from a single gene cluster, see [45]. We find 8 Y RNAs in the pig genome using structure based homology search. 4 are low-scoring and are scattered over the genome. In most mammalian genomes they are accompanied by a large number of retro-pseudogenes [46]. In the pig these numbers are relatively small (8 Y RNA candidates detected in pig in total with Infernal and the cutoffs applied for the high confident annotation, compared to 690 in human detected with the same cutoffs).

The pig genome harbours a complete Y RNA cluster: The arrangement on pig chromosome 9 Y1(+ strand), Y3(- strand), Y4(- strand), Y5(- strand) conforms to the ancestral eutherian prototype (with Y1 inverted relative to the ancestral mammalian arrangement). It is unclear, however, whether the Y5 locus is still functional in the pig genome or already a pseudogenized remnant. The divergence from the human sequence is much higher than that of the other three pig Y RNAs, indicating a drastic reduction of stabilizing selection: Y1 1.8%, Y3 3.0% Y4 4.3% Y5 13.6% based on UCSC browser repeat annotation. This is to be compared with the situation in cow and dolphin were Y5 is deteriorated to a degree were it is almost certainly non-functional. To further test this we tested the Y RNAs with cmalign for all seven organisms including pig where the pairwise alignment points to complete Y RNA clusters. The seven organisms are pig, cow, dolphin, horse, dog, human and rabbit. Alignment to the covariance model (CM) clearly shows that Y1 and Y3 fits the model consistently in all organisms, while the Y4 and Y5 RNAs are all somewhat divergent in the pig/cow/dolphin part of the tree. However, only the cow and dolphin Y5 RNAs scores badly in the Rfam Y RNA model. The Y RNA alignments in Stockholm format are available on the website of this paper.

This section concludes the discussion about specific classes of structured RNAs and the curation of these RNAs. We proceed with a summary of the complete curated annotation

### Curated annotation

The high confident annotation is a combination of the results of the high confident homology pipeline and the miRDeep detected miRNA candidates. We obtained a total of 3,556 high confident loci (Table 7). Using a variety of methods presented above we were able to curate 13% of the annotated RNA loci while 37% of the annotations are marked as pseudogenes. Pseudogenes are either ncRNAs expected, but failing, to be PolII or PolIII transcripts, cis-regulatory elements not in the vicinity of annotated protein coding genes, or ribosomal RNAs not part of the cluster on chromosome 6.

**Table 7:** **Curated annotation**

	# high confident loci	# curated loci	# pseudogenes	# loci,#pseudogenes subtracted
cisreg-elements	139	93	31	108
lncRNA-loci	58	0	0	58
miRNA	369	125	0	369
putative-miRNA	155	0	0	155
ribozyme	8	1	5	3
rRNA	185	3	182	3
snoRNA	638	269	278	360
snRNA	1,030	69	960	70
tRNA	810	0	0	810
Other	153	3	125	28
Conflict	11	8	0	11
Sum	3,556	571	1,581	1,975

The high confident annotation is a combination of the results of the high confident homology pipeline and the miRDeep results. See Table 2 for row labels. Column labels: high confident is the high confident annotation prior to curation; curated are the number of loci curated by methods explained in the text; pseudogenes are the loci expected to be PolII/PolIII transcript, but failing to be so, ribosomal RNAs not part of the cluster on chromosome 6, and cis-regulatory elements without gene context. In the column overlaps to structured RNA loci annotated by homology as well as putative miRNAs are given. Curated annotation contains loci that are a) curated or b) loci not tested in the curation procedure, *e.g.*, miRNA loci. High confident: is the complete high confident annotation (homology + miRDeep). 8 miRNAs detected by miRDeep, but not by high confident BLAST where re-annotated in the section *miRNAs in the pig genome*. A table with the 3,877 medium confident loci and 36,647 low confident loci are found in Additional file 1: Table S20.

The curated annotation presented in Table 7 contains the 571 genes that passed the curation procedures presented in the preceding sections. The same curation procedures marked 1,581 ncRNA genes as pseudogenes. The curated annotation represents a substantial manual annotation effort, which considered several aspects of the different classes of structured RNAs discussed in the previous sections, including the context of protein coding genes, clustering of neighbouring RNA loci, synteny, as well as sequence and structural conservation of the annotation in other organisms. The curated structured RNAs are clearly those that comes with the highest certainty, however, fields such as small RNA-seq, will benefit from the high confident annotation, and in some cases even from the medium or low confident annotations.

## Discussion

Annotating genomes for functional elements is in general a non-trivial problem, in particular, if this is considered to include full functional annotation. A recent conservative effort to characterize proteins, CharProtDB, contains only 213 human entries [47], approximately one percent of the total number of protein coding genes. Clearly the use of sequence homology to screen the genome for known sequences is useful. However, in contrast to protein coding genes, search for structured RNAs is a much harder task, partly due to the long ranging interactions in RNA (secondary) structure.

Other protocols for annotating ncRNA exists, such as RNAspace, but it is not feasible for full genome-wide analysis of a mammalian genome [48].

Our pipeline utilized sequence and structure homology search, small RNA-seq and *de novo* RNA structure prediction in genomic sequence. We obtained high confident annotation of 3,556 structured RNAs distributed over 3,348 (full length) ncRNA genes, 11 conflict loci, 139 cis-regulatory elements, as well as 58 lncRNA loci. The 11 conflict loci are all identified as very good matches to either snoRNAs, to SRP RNAs or to the rRNA subunit 28S. However, part of these RNA sequences have been identified as miRNAs in other studies and added to miRBase. Deciding if the 11 conflict RNAs can also serve as miRNAs is outside the scope of this study. We chose to keep these annotations as high confident as we have no doubt about their annotation, only their possible function as miRNAs are yet to be determined. Note, that 5 of the conflicts are also annotated as conflicts between snoRNAs and miRNAs in human or mouse; 3 conflicts are found by high confident BLAST to be between miRNAs and seed members of the SRP RNA family; and the annotation of 28S is beyond dispute as it is part of a full rRNA cluster. This leaves 2 conflicts unaccounted for, which are the results of more recent additions to miRBase from organisms besides mouse and human.

We found 524 miRNA loci from 465 different (putative) miRNAs precursor sequences, of which 174 were annotated for pig already. 151 are so far annotated only in pig, however, only 5 appear to be confined to the Laurasiatheria, 4 found only in pig and one specific to the cow/pig/dolphin part of the tree.

The sequence based part of the pipeline complete the highly conservative part of the pipeline, by employing BLAST cutoffs of 0.1 in E-value, 95% identity with a coverage of at least 95% coverage of the RNA query sequence. Correspondingly, for the Infernal screen we employed only the family specific gathering score cutoffs and ran Infernal directly on the genomic sequence, which is in contrast to the official Rfam annotation where BLAST pre-filtering is performed. Our Infernal screened yield 709,026 loci matches, and we found that BLAST filtering as well as additional Infernal E-value filtering were needed to obtain confident results. Our results are not directly comparable to those of Rfam, because the exact filtering procedure used by Rfam is unpublished and thereby not directly available for assessment.

The high confident annotation is the result of an automated pipeline, while the manual annotation is presented in the form of the curated genes. In particular the results of the Infernal and snoStrip results will contain pseudogenes and the Infernal results may contain false positives as well. We therefore ran all tools on a local (120 nt window) shuffled version (see Methods) of the pig genome to obtain an estimation of the false positives on random data. We found that most tools, even at their lowest confidence levels produced no results on the shuffled sequence. This includes, RNAmmer, tRNAscan-SE, snoStrip, and BLAST (with the exception of 6 very short CRISPR sequences not expected in vertebrate genomes). The exception is Infernal, which produces a high number of hits on the shuffled sequence without filtering in addition to what is offered by the family specific gathering score cutoffs. However, by introducing a global Infernal E-value cutoff of 1e-3 in addition to the gathering score cutoff, we were able to cut the number of false positives in the shuffled data down to just one low confident hit and no high confident hits. A second potential problem using Infernal is the possibility for incorrect family assignment (*e.g.*, a snoRNA or miRNA assigned with a wrong family name). However, with zero conflicts in the high confident Infernal results on the unshuffled data and only 7 within the low confident results, this appears not to be a problem with Infernal used as we do here.

This work represents a substantial computational effort. While most of the annotation tools, and the post processing could be run for the whole genome on a fast stand-alone workstation, two major parts of the pipeline precludes this. Firstly, running Infernal for all Rfam models takes around 25 CPU years. Secondly, the pairwise alignments take in the order of 1 CPU year per genome we aligned against the pig genome. Infernal has recently achieved a substantial speed up by taking full advantage of the recent developments in the HMM’er code [49]. The improved HMM’er filtering will hopefully replace the need for BLAST filtering of the Infernal results since a much better false discovery rate is reported in the cited paper. However, we will have to wait for a later version of the Rfam database as even the latest version (11.0) is constructed with Infernal version 1.0.2. With the improved Infernal code the annotation part of the pipeline could be run on a normal workstation within a few weeks. However, the pairwise and multiple alignments, and thereby the synteny analysis will still require access to a high-performance computing cluster.

To further ensure confidence, we manually or semi-automatically inspected a selected subset of the 3,556 annotated RNA loci to reach a total of 571 curated structured RNA and 1,581 pseudogenes. One class of genes inspected was Y RNAs for which the inspection gave rise to the observation that the Y5 loci, in contrast to the others, already might have mutated into a non-functional state.

Ensembl version 68 provides ncRNA annotation for the pig genome as part of their annotation pipeline. Ensembl version 68 contains 2,965 structured RNA loci for the pig genome divided on rRNAs, miRNAs, misc-RNAs, snoRNAs and snRNAs. Of the 3,418 high confident annotations obtained by our homology search only 1,955 are present in the Ensembl annotation. In particular all tRNAs are missing entirely or are annotated as miRNAs, though the basis for annotation is unclear to us. Likewise the cis-regulatory elements are missing completely from the Ensembl annotation. In the other end of the spectrum, we find that 522 of the 2,965 Ensembl annotations are not found in the low confident annotation. The ncRNAs found extra in the Ensembl annotation are in most cases miRNAs without an assigned gene name, indicating that they are probably low confident miRNAs from Rfam. A comparison of the two annotations is found in see Additional file 1: Table S15.

To further search for *de novo* RNA structures we employed RNAz [21]. RNAz uses a sliding window based approach which chop out sections (typically of 120 nt) of multiple (sequence) alignments and measure the structure potential over a background in that window. A main concern is the input MultiZ MAF blocks, which are typically used as outset. The more organisms the MAF blocks contain, the more fragmented they become and the input space is not covered optimally. Another concern is the heterogeneous distribution of the evolutionary distances between the organisms in the alignments. Ideally RNAz should be fed with equally distanced organisms. The RNAz search provided 83,869 structurally conserved RNA loci of which 528 overlapped with the high confident homology based annotation or novel miRNAs. The class best covered by RNAz loci were the miRNAs, where 65% of the loci were recovered. Most RNAz loci were found in the intergenic or intronic regions, however, around 10% were found near the beginning or end of protein coding transcripts.

A second source of novel candidate RNAs in pig comes from the small RNA-seq data presented in this work. Here, the main analysis of the sequencing data is performed with miRDeep and we therefore only annotate novel miRNA candidates. However, with deepBlockAlign (DBA) we also found novel RNA candidates in other classes of RNAs, but without more computational support or experimental evidence these are not annotated with high confidence and are therefore only presented as a special track. For miRNAs, the RNA-seq profiles are particularly specific and the mirDBA tool [35] for aligning miRNA RNA-seq profiles directly against a compiled database of RNA-seq profiles was developed in parallel with this work. Future updates will use the full potential of mirDBA.

Early versions of the pig genome have been investigated for ncRNAs, these studies investigated a low (0.66x) coverage version of the genome [11] in which 51 miRNAs were identified and a later study investigated the pig ESTs [13] in which 8 additional miRNAs not found in the previous study were identified. We confirm all these 59 miRNAs in this paper, except for 3 where the sequence is missing in the current assembly of the pig genome. The study of the low coverage version of the genome did not include a search for other types of ncRNAs, while the ESTs from the second study were selected with a bias against small ncRNAs and a comparison of the obtained full ncRNA annotations with the present is therefore not entirely meaningful.

Here, we focused on annotating structured RNAs (ncRNAs and RNA structural regulatory elements in UTRs of mRNAs) and we did not include lncRNAs with the exception of partial lncRNAs included in Rfam. LncRNA in contrast to the many structured RNAs are just emerging and systematically characterized by genomic coordinates, *e.g.*, [50]. These lncRNAs have been obtained through comprehensive transcription studies. Given that lncRNAs in general are poorly conserved and similar studies have not been carried out in pig, we in this study focused on structured RNA (of which some *de novo* RNA structure candidates may overlap lncRNAs).

Overall our ncRNA annotation is of further interest in the light of pig as production and model animal. The known ncRNAs holds potential to be studied in specific problems, where for example mapped RNAs can be useful in subsequent studies, such as differential expression of ncRNAs, *e.g.*, in necrotic lung tissue [51] and skeletal muscle tissue [52]. Furthermore, as next generation sequencing will make it possible to increase the quality of Quantitative Trait Loci (QTL) and genome-wide association studies, these regions can be analyzed for ncRNA content. For example we considered all QTL contributing regions less than 500,000 nt listed in the pigQTLdb [53] and by Fischer Exact test we found 55 QTL regions to be enriched (p <0.01) with RNAz candidates (RNAz p-score >0.9) (data not shown). A similar, but weaker trend is observed for a few cases when testing the enrichment for only the annotated families and classes of structured RNAs.

As an example of what one can do with an ncRNA annotation in relation to a larger set of genes, we analyzed the 46 regions of the pig genome containing olfactory receptor genes [54]. The regions overlapped with 249 of our high confident ncRNA candidates, including, 170 tRNAs, 8 known miRNAs, 1 miRNA predicted by miRDeep, 25 snoRNAs and 7 cis-regulatory elements. In particular the miRNAs and cis-regulatory elements could be involved in regulation of the olfactory receptor genes. The genome structure can be relevant for further studies aiming at mutually to elucidate the functionality as well as the mechanisms underlying these traits.

## Conclusions

We have conducted the, to date, probably most comprehensive ncRNA annotation of a mammalian genome in a single analysis, and we have obtained an extensive annotation of structured RNAs in *Sus scrofa*. We constructed a semi-automated pipeline enabling manual curation at reasonable level while also incorporating RNA-seq data and including a layer of *de novo* RNA structures. Overall we annotated 3,556 high confident structured RNAs of which a subset was inspected leading to 571 curated annotations. In addition we found 83,869 structurally conserved loci using RNAz, of which 528 overlapped with the homology based annotation or novel miRNA candidates. These structured RNAs constitute an essential resource for studying and generating hypothesis of genetic mechanisms underlying the various production traits. All the data resources are available at http://rth.dk/resources/rnannotator/susscr102/version1.02.

## Methods

Example command lines and program options for calling of the different tools are given in Additional file 1: Table S16 for the annotation pipeline in general, Additional file 1: Table S17 for the Infernal runs, and Additional file 1: Table S18 for the alignment pipeline.

### Sequence based homology search

The sequence based homology search was performed using blastall (blastn) from the BLAST [23] tool version 2.2.23 and a number of databases (miRNA hairpins from miRBase (version 18.0) [55–58], the vertebrate rRNAs from Silva (version 102) [59], snoRNAs from snoRNAbase (version 3) [60], tRNAs from tRNAdb (version 2009) [61], and seed sequences from Rfam (version 10.1) [7, 8]). The databases were not redundancy reduced prior to the search and there may therefore be overlapping hits on a given genomic position. Redundant overlaps were resolved as follows: Within each database the hits were classified as miRNAs, tRNAs, snoRNAs, snRNAs, rRNAs, cis- regulatory elements, or as *other* for those without a clear classification while still keeping track of the origin. Within the classified hits of each database the hit having the lowest BLAST E-value was chosen. For example, SNORA81 resides on chromosome 13 in pig. Two overlapping high confident BLAST hits from the Rfam version 10.1 seed sequences were found. One from small brown bat, and one as it turns out from pig. Not surprisingly, the hit having the lowest E-value is the sequence from pig. At the same genomic position and strand, a high confident hit from miRBase is found, eca-mir-1248 is found. The sequence was thus annotated twice, once as a snoRNA and once as a miRNA. Therefore this region will be marked as a conflict. For the high confident results, we required that a minimum of 95% align length and 95% identity of the BLAST hit, For the medium confident results, we required 92.5% length and 92.5% identity, and for the low confident results 90% length and 90% identity. All BLAST searches were performed with an E-value cutoff of 0.1.

### Structure based homology

The structure based homology search was based on the Infernal 1.02 [24] models from Rfam 10.1 [8]. A scan of the complete pig genome without any BLAST pre-filtering and with all models was performed. The Infernal options for a default search is given in the CM model files provided by Rfam, and depends on the model with the following parameters as variants: -g (global/local(default) alignment), -T (score cut off, set at the so-called gathering score cutoff for the given model), and –fil-no-hmm (turns of HMM pre-filtering, speeding up the search at the cost of sensitivity). The Infernal options were used as specified in the CM model file, except for three models Intron_gpI, RNase_MRP, and SSU_rRNA_bacteria where HMM pre-filtering was enforced because of the excessive time consumption posed by these models.

The Rfam families are generally constructed with sensitivity in mind. Each family comes with a gathering score cutoff, which is the recommended cutoff for scanning for new members of the family. This cutoff will by the very construction be too sensitive, but it is the only alternative that Rfam provides.

Initially the scan was performed with the models of Rfam version 10.0 against a pre-release of the pig genome version 10. Models added or updated in Rfam version 10.1 were later applied to the full genome (pig genome version 10.2). The unchanged models from Rfam version 10.0 were applied to new or altered sequence between the pre-release of the pig genome and the final version of the genome otherwise used in this paper. Because of this, the exact run time for the pig genome is unknown, however, a corresponding scan of the human genome took 25 CPU years. The latest version of Infernal is claimed [49] to be a 100 times faster than the version used in this study, which would put the entire annotation pipeline within reach of a small computational cluster.

The raw output of the Infernal scan of the Rfam families on pig yielded a total of 709,026 loci, where 8% of these had conflicting Rfam annotation. Additional filtering is obviously necessary even at the lowest of confidence levels. The first thing one should notice is that the construction of the families and setting of the cutoffs by the Rfam consortium includes a BLAST filtering step, and the exact procedure of which is unpublished. This vastly enhances the selectivity of the families and allows much lower Infernal score cutoffs than may be reasonable if the families are used without BLAST pre-filtering, which for the pig genome results in the 709,026 matches reported above.

We therefore chose to apply 4 additional filters as explained below, 1. using only families for which we expect a hit (*i.e.* only families with seeds in vertebrates), 2. Infernal E-value filtering for families with very loose E-value cutoffs implied by the gathering score, 3. a BLAST filter, 4. special filtering for the miRNA families.

*Filter 1.* Not all families are known for vertebrate genomes. We limit the analysis to the 695 families with seed sequences in vertebrates.

*Filter 2.* We normally used the family’s gathering score as cutoff. However, in some cases this cutoff was very loose when translated to Infernal E-values. The score cutoff on an Infernal CM model implies an equivalent Infernal E-value cutoff. The relationship between the two is found by calibration of each family. In some cases we find the model gathering score cutoff to imply Infernal E-value cutoffs up to around 1e6 (the extreme case is S_pombe_snR97). For non-miRNA families we therefore impose an additional Infernal E-value cutoff of 1e-3 in the cases where the E-value cutoff implied by the gathering score cutoff is above 1e-3. The cutoff was chosen because with stricter E-value cutoffs we quickly start to remove *valid* candidates from tRNA and snoRNA families.

To further justify the global E-value cutoff, we ran the 514 Infernal models with (low) confident hits in pig on shuffled sequence (see the Subsection False positive rate of the homology search pipeline for details on the shuffling procedure). 208 or about 40% of the families produced hits in the shuffled sequence at their family specific score cutoffs. The worst being mir-684 with 3,212 hits and 5S_rRNA with 719 hits. When we reduce the global Infernal E-value cutoff, we gradually reduce the number of families that have matches in the shuffled sequence to 161 at an Infernal E-value cutoff of 1 where the worst family is Histone-3-prime-UTR with 47 hits. Down to only two hits in two different families at a cutoff of 1e-3 (See the graph in the Additional file 1: Figure S21). This means that we only observe two false positives in the shuffled sequence at the low confident cutoff prior to the BLAST filtering. At our the cutoffs used for the low confidence annotation a single false positive is found, while none are found at the more strict cutoff levels.

*Filter 3.* The specificity and sensitivity of a family is highly dependent on the BLAST filtering, and using the models score cutoff alone will often be far too loose. However, even a very loose BLAST filter was sufficient to greatly enhance specificity without affecting the sensitivity of most models. That is the sequence based homology filter anchored the families to the right genomic locations and greatly reduce the number of hits. For each family we applied a BLAST filter based the family’s seed sequences. We tested the implications of a common BLAST E-value cutoff for all non-miRNA families. By gradually relaxing (increasing) the cutoff, we found that conflict of annotation occurred at an E-value of 1e-1 (two different Rfam families annotated the same locus). By gradually decreasing the cutoff we found that with a BLAST cutoff value of 1e-5 we loose valid annotations. The worst case is the Histone-3-prime-UTR family, where 17 of 21 matches are lost and since these 17 matches are part of three clusters of Histone-3-prime-UTR annotations, we believe them to be valid family members. We therefore settled on an intermediate BLAST E-value cutoff of 1e-3 for all non-miRNA families, which by observation corresponds to an exact sequence match of length 24. A 100% identical sequence match of this length was considered sufficient to filter the structure matches down to those matching the correct Rfam family by being in the order of the shorter sequence motifs, *e.g.*, the length of a mature miRNA. The BLAST cutoff reduced the number of loci with conflicting families to zero. For the medium confident results we filtered with an E-value of 1e-1, which is the limit of the BLAST run we did above. And for the low confident results, the BLAST filter is turned of completely. Note that, the HMM filtering that some families provide is in a sense a refinement of the BLAST filtering.

*Filter 4.* We found the miRNA families to be particularly troublesome Rfam families. This may not be too surprising given their simple hairpin loop structure, which is ubiquitous in the mammalian genomes. In any case, we exempt the Rfam miRNA families from the high confident results, both for the structure similarity search and for sequence similarity search. We do, however, include the Rfam miRNA families at the medium and low confident annotation levels. See also the subsection *RNAs of the individual annotation tools* for a specific example of a problematic mir-family.

While, we apply a maximum Infernal E-value cutoff of 1e-3 for most families, we can use more strict cutoffs of 1e-6 or 1e-9, for low and medium confident annotation of Rfam miRNA families since we observed that the seed sequences of the miRNA families in general have much lower Infernal E-values. However, with Infernal E-value cutoffs lower than 1e-9 we loose valid miRNA family members observed with high confident BLAST against miRBase. We furthermore require a BLAST cutoff at all confidence levels. Without this requirement, we found that about 40% of the miRNA loci would contain conflicts even after imposing the extra Infernal E-value filters.

In summary, annotating full vertebrate genomes with Rfam and Infernal is a difficult task, albeit without Infernal it is hard to annotate structured RNAs at all (a specific example is discussed in the Results section under snoRNAs). The automatic annotation that we present in here is, even with the extra filters, is likely to contain a few pseudogenes or in worst case uncertain annotations. Thus, the curated genes always contain the annotation with the highest certainty.

### Class specific tools

RNAmmer [25] version 1.2 tool were used for rRNA detection and the results were used as is. In this mode the tool detects a number of 8S, 18S and 28S fragments which will need hand curation.

tRNAscan-SE version 1.23 [10] was used to search for tRNAs. For mammalian genomes it is often necessary to apply additional filtering of the tRNAscan-SE results. For the high and medium confident results we apply the filtering used for the cow genome in the database on the tRNAscan-SE website: The high confident results we report are filtered by HMM Score 10.0, 2’Str Score 5.0, and COVE Score 20 when the tRNA is found by both first time parsers and by COVE Score above 55 when the tRNA is only found by one. The low confident results are the unfiltered results of the tRNAscan-SE.

snoStrip [26] in a pre-release version was used as a specialized tool for identifying snoRNAs from incomplete BLAST hits. The BLAST search for snoRNAs was done using all snoRNAs from an internally curated snoRNA database. BLAST-hits were accepted as homologous snoRNAs with a sequence identity higher than 85% and a minimal length of at least 90% of the given query, the *E*-value cutoff was 1e-10. Iteratively, we searched with this method as long as we were able to find new snoRNA homologs. In order to further remove pseudogenes, snoRNA-boxes were scored based on PWM generated with the curated snoRNA sequences contained in the database. The false positive rate of the snoStrip tool was gauged by running *the release version* on a shuffled version of the pig genome. No results were found in the shuffled sequence when running snoStrip at the same cutoffs as those applied on the real pig genome.

### Detection of active PolIII and PolII transcribed RNAs

PolIII transcribed RNAs like U6 may behave like repetitive elements, making it difficult to know which annotated element undertakes the expected cellular function.

Based on the assumption that only transcribed elements should actually be functional, we devised a method based on the detection of the Proximal Sequence Element (PSE) and TATA-Box upstream of the annotated transcript, two sequence elements that are essential for the successful transcription of PolIII elements (see Additional file 1: Figure S22). A slice of 100 nts directly upstream of the annotated element of curated human PolIII transcripts (7SK, U6, U6atac, Y RNA, RNaseP, RNaseMRP), was taken and searched for PSE and TATA (See Additional file 1: Table S21)

A strong TATA box signal was observed at the 3’end of the upstream region of the full set of high confident U6 snRNAs for the preliminary annotation when compared to a set of 1,000 random sequences(see Additional file 1: Table S23, Table S22 and Figure S23).

Similar to PolIII transcripts, PolII transcripts also possess promoter sequences upstream of the transcript. Those are called Proximal Sequence Element A, PSEA, (-50 nts upstream) and Proximal Sequence Element B, PSEB, (-25 nts upstream) (see Additional file 1: Table S24 and Figure S24).

It should be noted that PSEA with high scores on the set of raw PolII transcripts is significantly different from the PSEA distribution of the random sequences (see Additional file 1: Table S25, Table S26 and Figure S23). In the case of the putative PolII transcripts, the high-scoring PSEA elements preferentially locate 50 nts upstream of the transcript start.

Based on these PWMs the reported PolIII (U6, U6atac, 7SK, RNaseP_nuc, RNaseMRP) transcripts were filtered. Finally, alignments of the selected sequences were generated either with clustalw [62] and/or cmalign [24] in order to better spot sequences that might not be functional.

### Genome alignments

The pairwise alignments were performed using LASTZ version 1.02, and the alignments were subsequently chained using Kent’s axtChain, which is part of the UCSC tool chain with code base dated September 2011 [30].

The alignment is performed with LASTZ [29] on smaller chunks of the genomes involved. The target genome(pig) is cut down to fragments of at most 80,010,000 with an overlap between chunks of 10,000 nucleotides. The source genome (see Table 4 for at list of source genomes) is cut down to fragments of at most 80,000,000. The parameters used for the LASTZ alignments are divided into *closely* and *distantly* related genomes. See Table 4 for which genomes implies which parameters and Additional file 1: Table S19 for the exact options used.

The LASTZ alignments are chained with the axtChain tool and merged into one big chain file containing all the chained alignments(chains). These chains may overlap due to duplication of sequence in either target or source. The chained alignments were converted to single coverage alignments(nets) of the pig genome using chainNet. The netted alignments, which are only single coverage of the pig genome not the source genomes, were cleaned for non-syntenic alignments with netFilter or by forming reciprocal best nets, in which only the best alignment is kept in case of multiple coverage of the source genome. (axtChain, chainNet and netFilter are all part of the UCSC tool chain [30]).

The pairwise alignments were joined to a multiple alignment with the roast program from the Threaded-Blockset-Aligner (TBA/MultiZ) [36]. Default parameters were used for the program, *i.e.*, a dynamic programming range of 30 and a minimum MAF block size of 1. The pairwise alignments were cleaned for synteny prior to running roast; either by using the syntenic nets or by using the reciprocal best nets in the cases of many unplaced contigs in the source genome, a scaffold based genome, or low coverage of the pig genome. See Table 4 for the nets used. A phylogenetic tree without branch lengths is required as part of the input to roast, see Figure 3.

The UCSC tool chain allows an automatic cleanup of the best single coverage alignments of the pig against other genomes in which alignments of gap-filling character has been deleted. These so-called syntenic nets are ideal for identification of synteny. The synteny blocks from the syntenic nets were identified by merging neighbouring alignments with identical source chromosome, target chromosome and strand information. The alignments were only merged when the gap between them were smaller than 5,000 nt. Only merged alignments at least 5,000 nt long were used in the further analysis.

### RNAz

RNAz version 2.1 [21] was used for *de novo* prediction. RNAz identifies loci of conserved structure based upon a multiple alignment. RNAz ran on windows of the MAF blocks from the multiple alignment. Windows were of size 120, step size 40. A maximum of 6 sequences were allowed in each window. In case of more than 6 sequences in a window, the alignments were optimized for an average sequence identity of 80%. RNAz was used with the di-nucleotide option and with strand identification. At least three sequences were required to make a prediction. The results are filtered according to a RNAz p-score 0.9. The p-score is a reliability score for the RNA prediction and *not* a p-value. RNAz is known to have a high false positive rate [21] and we therefore ran RNAz on MAF windows shuffled using multiperm [63]. We found false positive rates of 61% and 52% with p-score cutoffs of 0.5 and 0.9, respectively (with the false positive rates defined as the number of shuffled windows with RNA structures divided by the corresponding number on the unshuffled windows). In accordance with the original paper where the respective false positive rates where 59% and 54% [21].

### False positive rate of the homology search pipeline

To gauge the false positive rate of the pipeline, we created a shuffled version of the pig genome and ran the prediction tools with the original settings on the pig genome.

The approximate size of the typical structured RNAs that we find is around 120 nt. Accordingly, the contigs of the pig genome were shuffled in 120 nt windows with uShuffle [64] set to preserve the di-nucleotide contents. The shuffled windows were then put together in the corresponding order in the contigs to create a shuffled pig-genome with the exact same (di-)nucleotide contents, local GC content bias and contig size as the unshuffled one. The false positive rate of the tools were then found as the number of hits obtained in the shuffled genome.

### Preparation of small RNA libraries

Total RNA was isolated from liver, lung, kidney, colon, small intestine, spleen, lymph node, cerebellum, frontal lobe, and placenta of Pinky, a genetically identical clone of TJ Tabasco, using mirVana miRNA™ Isolation Kit (Ambion). Ten micrograms of RNA from each tissue was separated on 15% polyacrylamide Tris-Borate-EDTA-Urea gels (Bio-Rad), stained with SYBR®; Safe DNA gel stain (Invitrogen) and visualized using a Dark Reader Transilluminator (Clare Chemical Research). Small RNA in the 15–30 nt range was excised, eluted from the gel slice in 0.3 M NaCl, precipitated in ethanol, and finally dissolved in DEPC-treated water. Next, small RNA libraries were prepared for next-generation sequencing using the AIR™ Small RNA Sequencing Kit (Bioo Scientific Corporation) in combination with AIR™ Barcoded Adenylated Adaptors (Bioo Scientific Corporation) to enable multiplex sequencing. The 5’ adaptor and 3’ adaptors were sequentially ligated to the small RNA and the ligation products were gel purified between each ligation step on 10% Tris-Borate-EDTA-Urea polyacrylamide gels. The gel-purified ligation products were reverse transcribed and amplified by 20 cycles of PCR. The resulting 100 bp PCR fragments were separated on 4% high resolution agarose gels (Metaphor Agarose, Lonza), purified using a QIAquick gel extraction kit (QIAGEN), eluted in 30 *μ*l 10mM Tris-HCl pH 8.5 (EB buffer) and dried down to a volume of 15 *μ*L using a SpeedVac. The size distributions, qualities and quantities of the libraries were measured using a NanoDrop ND-1000 Spectrophotometer and an Agilent 2100 Bioanalyzer. Finally, the concentrations were adjusted to 10 nM using EB buffer.

### Illumina Genome Analyzer Sequencing and primary sequence analysis

The ten barcoded libraries were cluster amplified using an Illumina Cluster Station followed by 50 cycles of sequencing on an Illumina Genome Analyzer IIx Sequencer and primary data analysis using the Illumina Pipeline software (CASAVA 1.7). The sequence reads were first processed by demultiplexing and trimming of adaptor sequences, using only reads with full length 3’ adaptor sequences. Sequence reads shorter than 15 nt were discarded, leaving approximately 40M reads for further analysis. Identical reads were collapsed into unique reads that were quantified (558K), and subsequently aligned to build 10.2 of the *Sus scrofa* reference genome using Megablast (-W 12, -p 100). Sequences mapping to more than five genomic loci were discarded, and only sequences with perfect, full-length alignment (100% identity) to the genome were retained for evaluation, yielding 210K collapsed reads that represent 27.7M reads.

### miRDeepanalysis of the small RNA data

Annotation of the reference pig genome with known and candidate miRNA genes was accomplished with miRDeep version 1 [28, 65–68]. The sequences of the miRNAs will be submitted to miRBase for annotation.

The raw output from miRDeep indicated 467 putative miRNA loci with at least 10 reads present. However, due to duplication of genomic sequence in assembled chromosomes as well as unplaced scaffolds only 421 different miRNA precursor sequences, and 381 mature sequences were found. (Note: The 421 miRNA precursors were given provisional names, pre-1 …pre-421, pending correct names upon addition to miRBase).

Exact mapping of these 421 sequences resulted in 459 loci, 7 of them ambiguous because of overlapping precursor sequences. In 5 of the seven cases the ambiguity could be resolved by noting that both star and mature sequences were identical between the conflicting precursor sequences. In the last 2 cases the star reads were missing and both precursors discarded. In one particular instance a precursor was reported with to different mature sequences, one two nucleotides longer than the other. The shortest mature sequence was discarded in this instance.

The 459 loci were compared to the high confident annotation obtained in Table 2 and were found to overlap with snoRNAs, U5 or 28S in a total of 9 instances and the corresponding precursor sequences were discarded. Loci overlapping with conflicts already marked in the homology search based results were kept.

In 7 instances of novel miRNAs, we found that the precursor sequences overlapped with protein coding annotation, which led to the move of 6 precursor sequences to the medium confident annotation.

All mature sequences were mapped against all precursor sequences to identify *misplaced* reads. That is, reads mapping to multiple places in the genome or reads incorrectly placed because of mapping issues. Careful examination of the mapping results lead to the deletion of a further 10 precursor sequences.

9 sequences were deleted because they would introduce conflicts of annotation, and 8 sequences were deleted due to overlapping pre-miRNA coordinates. 10 sequences were deleted because they originated from misplaced reads. Finally 6 sequences were moved to the medium confident annotation due to overlap with protein coding annotation. Resulting in a final 388 precursor sequences, of which 232 where found to have both star and mature reads. The 388 precursor sequences mapped to a total of 431 loci. All details are found in Additional file 2.

### deepBlockAlign

The mapped reads from the small RNA library were grouped into block groups using blockbuster version 1.0 (with parameters: -distance 30, -minBlockHeight 2, -minClusterHeight 10, -scale 0.5 -blockHeight abs) [33]. The block groups were aligned using deepBlockAlign version 1.0, which aligns the relative read expressions and arrangements of reads of the two block groups. The alignment score from deepBlockAlign lies between 0 that suggests perfect dissimilarity and 1 that suggests perfect similarity between the two block groups. We require that the length of block groups were at least 50 to make sure that structure could be obtained from the read profile and for example singular peaks of stacked reads could be filtered out). deepBlockAlign scores are only considered meaning when >=0.6. This cutoff is based on an empirical study of two biological replicates, where 95% of the read profiles had an alignment score >= 0.6 between the two samples.

## Note added in proof

During completion of this paper a release version of the program snoStrip was published. We have employed this updated version, which resulted in minor changes for the annotated snoRNAs. Thus, the number of high confident annotated snoRNAs is now changed from 638 to 621, in which the subset of curated snoRNAs now consist of 289 compared to the previous 269. Overall the total number of high confident annotated RNAs has correspondingly changed from 3,556 to 3,539. These changes are now included in a further release, 1.03 (see http://rth.dk/resources/rnannotator/susscr102/version1.03/ where we describe the changes to the previous version (1.02) presented in the paper).

## Electronic supplementary material

Additional file 1: **Comprises the Supplementary Tables S1–S26 and Supplementary Figures S1–S24.** Additional data and tracks for genome browser visualisation also available on http://rth.dk/resources/rnannotator/susscr102/version1.02. (PDF 648 KB)

Additional file 2: **Spreadsheet containing data for the** **miRDeep** **detection of miRNAs.** (XLS 117 KB)
