# Supplementary material for: Structured RNAs and synteny regions in the pig genome
Source: BMC Genomics. 2014 Jun 10;15(1):459. doi: 10.1186/1471-2164-15-459 (PMC4124155; doi:10.1186/1471-2164-15-459)
Supplement: Supplementary file 1 — Additional file 1: Comprises the Supplementary Tables S1–S26 and Supplementary Figures S1–S24. Additional data and tracks for genome browser visualisation also available on http://rth.dk/resources/rnannotator/susscr102/version1.02. (PDF 648 KB) [file 12864_2013_6316_MOESM1_ESM.pdf]

Additional file 1:
Structured RNAs and synteny regions in the pig genome

Christian Anthon[1,2], Hakim Tafer[3,4], Jakob H. Havgaard[1,2], Bo Thomsen[5], Jakob Hedegaard[5,6], Stefan E. Seemann[1,2], Sachin Pundhir[1,2], Stephanie Kehr[3,4], Sebastian Bartschat[3,4], Mathilde Nielsen[5], Rasmus O. Nielsen[5,7], Merete Fredholm[1,2], Peter F. Stadler[1,3,4] and Jan Gorodkin[1,2]*

[1]Center for non-coding RNA in Technology and Health, IBHV, University of Copenhagen, DK-1870 Frederiksberg, Denmark
[2]Department of Basic Animal and Veterinary Sciences, University of Copenhagen, DK-1870 Frederiksberg, Denmark
[3]Bioinformatics Group, Department of Computer Science, Interdisciplinary Center for Bioinformatics
[4]Transcriptome Bioinformatics group, LIFE, Leipzig Research Center for Civilization Diseases, Universität Leipzig, Philipp-Rosenthal-Strasse 27, D-04107 Leipzig, Germany
[5]Dept. of Molecular Biology and Genetics, Aarhus University, Blichers Allé 20, DK-8830 Tjele, Denmark
[6]Department of Molecular Medicine (MOMA), Molecular Diagnostic Laboratory, Aarhus University Hospital, Skejby, Brendstrupgaardsvej 100, DK-8200 Aarhus N, Denmark
[7]Present address: GenoSkan A/S, Niels Pedersens Allé 2, DK-8830 Tjele, Denmark

Email: Jan Gorodkin*- gorodkin@rth.dk;

*Corresponding author

Additional material also available on http://rth.dk/resources/rnannotator/susscr102/version1.01

Contents

Table S1 - Priority of ncRNA tools . . . 3
Table S2 - Databases used for the sequence based homology search . . . 4
Table S3 - Sequence based homology search results . . . 5
Table S4 - Structure based homology search results . . . 6
Table S5 - Sequence based homology search Rfam . . . 7
Table S6 - `tRNAscan-SE` types . . . 8
Table S7 - High, medium and low confident results of the homology based pipeline . . . 10
Table S8 - Read supported annotation . . . 11
Table S9 - Duplicate ncRNA genes . . . 31
Table S10 - Genic context of the high confident annotations . . . 33
Table S11 - Clustering at different distances . . . 34
Table S12a - `RNAz` genic contexts and conservation in other organisms . . . 35
Table S12b - Sequence conservation in other organisms `RNAz` . . . 35
Table S13 - Lineage specific structured RNA loci within the high confident annotation . . . 36
Table S14 - miRNA clusters in human and conservation of clustered miRNAs in pig . . . 37
Table S15 - Comparison of presented annotation and Ensembl version 68 . . . 39
Table S16 - Command lines used for the annotation pipeline . . . 40
Table S17 - `Infernal` command lines . . . 41
Table S18 - Command lines used for the alignment pipeline . . . 82
Table S19 - `LASTZ` and `axtChain` options . . . 83
Table S20 - Final high, medium and low confident annotation . . . 84

Table S21 - 100nt upstream of human PolIII sequences 85
Table S22 - PWM for the PSE derived from PolIII sequences 86
Table S23 - PWM for the TATA box derived from PolIII sequences 87
Table S24 - 100nt upstream of human PolII sequences 88
Table S25 - PWM for the PSEA derived from PolII sequences 91
Table S26 - PWM for the PSEB derived from PolII sequences 92
Figure S1a - miRDeep predictions with read profile best aligning with snoRNAs 93
Figure S1b - miRDeep predictions with read profiles best aligning with snoRNAs 94
Figure S2 - miRDeep prediction with read profile not aligning to those of known ncRNAs 95
Figure S3 - Read profile annotated by deepBlockAlign with overlapping RNAz loci 96
Figure S4 - Read profile annotated by deepBlockAlign and overlapping with mir-431 97
Figure S5 - Read profile annotated by deepBlockAlign and overlapping with mir-223 97
Figure S6 - Read profile annotated by deepBlockAlign and overlapping with mir-1388 98
Figure S7 - Cluster size of ncRNA cluster found in the high confident annotation of the pig genome 99
Figure S8 - Number of RNAz loci versus maximum sequence identity cutoff outside the Laurasiatherian lineage 100
Figure S9 - Number of RNAz loci versus minimum sequence identity cutoff inside the Laurasiatherian lineage 101
Figure S10 - Conservation of the 1,595 human miRNA loci in the pig genome 102
Figure S11 - Conservation in the pig genome of the human miRNA loci in miRNA clusters 103
Figure S12 - mir-379 cluster in pig 104
Figure S13 - mir-493 cluster in pig 105
Figure S14 - mir-532 cluster in pig 106
Figure S15 - mir-17 cluster in pig 107
Figure S16 - mir-363 cluster in pig 108
Figure S17 - mir-367 cluster in pig 109
Figure S18 - mir-513a cluster in pig 110
Figure S19 - mir-450b cluster in pig 111
Figure S20 - mir-892c cluster in pig 112
Figure S21 - False positives in the Infernal results 113
Figure S22 - PolIII: Logo PSE element and TATA-box 114
Figure S23 - Density plot of the position distribution of the PSEA elements 115
Figure S24 - PolII: Logo of PSE element and TATA-box 116

Table S1 - Priority of ncRNA tools

Priority of tools in the naming of the RNAs. The general order is class specific tools, `BLAST`, `Infernal`/Rfam, *de novo* tools.

`RNAmmer`
`tRNAscan`
`BLAST` silva
`BLAST` mirbase
`BLAST` trnadb
`BLAST` rfam
`snoStrip`
`Infernal`/Rfam
`BLAST` snoRNAdb
`miRDeep`
`RNAz`

Table S2 - Databases used for the sequence based homology search
Databases and version used for the sequence based homology search.

miRBase	version 18.0 ssc hairpins[1, 2, 3, 4]
miRBase	version 18.0 all hairpins[1, 2, 3, 4]
Rfam	version 10.1, seed sequences only[5, 6]
Silva rRNAdb	Version 102, vertebrates only[7]
snoRNAbase	Version 3[8]
tRNAdb	Version 2009[9]

Table S3 - Sequence based homology search results

Results of the BLAST scan of the databases against the pig genome using high or medium confident cutoffs. See the main paper Table 2 for an explanation of row labels. The number of RNA (sub) families and the number of annotated loci at high and medium confident BLAST cutoffs. Conflicts are the result of overlapping families of different RNA classes. In practice conflicts always arise between a miRNA and a longer non-miRNA family. Cutoffs, High: BLAST E-value 0.1, 95% identity, 95% of query covered, Medium: BLAST E-value 0.1, 92.5% identity, 92.5% of query covered, Low: BLAST E-value 0.1, 90% identity, 90% of query covered.

confidence levels	high		medium		low	
RNA class	families	loci	families	loci	families	loci
cisreg-elements	15	76	18	94	21	104
lncRNA-loci	32	32	42	42	47	47
miRNA	340	382	393	492	432	1,611
ribozyme	0	0	1	1	1	1
rRNA	2	3	2	3	3	4
snoRNA	58	68	119	153	155	229
snRNA	8	101	8	176	9	292
tRNA	43	355	44	397	44	434
other	5	9	6	10	6	12
conflicts	4	6	9	11	10	14
sum	507	1,032	642	1,379	728	2,748

Table S4 - Structure based homology search results

Results of the `Infernal`/Rfam 10.1 scan of the pig genome using different cutoffs. The number of RNA (sub) families and the number of annotated loci at different cutoff levels. Conflicts are the result of overlapping Rfam families, typically from similar RNA families. Cutoffs, All families are filtered with their family specific `Infernal` gathering score cutoff. Only families with seeds members from vertebrates were used. Additional filters: High: `BLAST` E=0.001, `Infernal` E=0.001 (no miRNA families). Medium: `BLAST` E=0.1, `Infernal`=0.001 (miRNAs `BLAST` E=0.001, `Infernal` E=1e-9). Low: no `BLAST` filter, `Infernal` E=0.001 (miRNAs `BLAST` E=0.1, `Infernal` E=1e-6).

confidence levels	high		medium		low	
RNA class	families	loci	families	loci	families	loci
cisreg-elements	31	125	31	145	33	156
lncRNA-loci	58	58	58	58	58	58
miRNA	0	0	188	397	191	431
ribozyme	3	8	3	8	4	9
rRNA	2	71	2	72	2	72
snRNA	10	1,030	10	1,066	10	1,147
snoRNA	205	508	205	540	205	590
tRNA	1	368	1	382	1	434
other	7	156	7	161	8	203
conflicts	0	0	1	1	3	7
sum	317	2,324	506	2,830	515	3,107

Table S5 - Sequence based homology search Rfam

Results of the BLAST scan of Rfam against the pig genome using high or medium confident cutoffs. Conflicts are found to be between hits from tRNA and tRNA-Sec. These conflicts are not detected during the normal BLAST pipeline since conflicts there were only marked between different RNA classes. Further more, these BLAST hits score insufficiently with Infernal to be true tRNA-Sec family members. For details also see Table S3

confidence levels	BLAST high		BLAST medium		BLAST low	
RNA class	families	loci	families	loci	families	loci
cisreg-elements	15	76	18	94	21	104
lncRNA-loci	32	32	42	42	47	47
miRNA	0	0	161	313	174	461
ribozyme	0	0	1	1	1	1
rRNA	1	1	1	1	2	2
snRNA	8	101	8	176	9	292
snoRNA	59	69	123	158	157	233
tRNA	1	230	1	265	1	305
other	6	12	6	13	6	16
conflicts	1	12	1	13	1	13
sum	123	533	362	1,076	419	1,474

Table S6 - `tRNAscan-SE` **types**

Types of tRNA identified with `tRNAscan-SE` filtered as explained in the main text section (`tRNAscan-SE`) as well as the raw results.

type	filtered	unfiltered
tRNA.Ala.AGC	23	32
tRNA.Ala.CGC	7	8
tRNA.Ala.GGC	0	17
tRNA.Ala.TGC	14	45
tRNA.Arg.ACG	7	7
tRNA.Arg.CCG	3	3
tRNA.Arg.CCT	4	5
tRNA.Arg.TCG	5	5
tRNA.Arg.TCT	7	12
tRNA.Asn.ATT	2	14
tRNA.Asn.GTT	18	128
tRNA.Asp.ATC	10	157
tRNA.Asp.GTC	97	1,513
tRNA.Cys.ACA	1	2
tRNA.Cys.GCA	23	28
tRNA.Gln.CTG	12	13
tRNA.Gln.TTG	8	33
tRNA.Glu.CTC	13	58
tRNA.Glu.TTC	235	1,376
tRNA.Gly.ACC	0	7
tRNA.Gly.CCC	6	9
tRNA.Gly.GCC	19	94
tRNA.Gly.TCC	10	39
tRNA.His.ATG	0	2
tRNA.His.GTG	10	36
tRNA.Ile.AAT	13	15
tRNA.Ile.GAT	0	2
tRNA.Ile.TAT	5	7
tRNA.Leu.AAG	7	7
tRNA.Leu.CAA	6	11
tRNA.Leu.CAG	5	7
tRNA.Leu.TAA	4	4
tRNA.Leu.TAG	5	6
tRNA.Lys.CTT	16	21
tRNA.Lys.TTT	38	175
tRNA.Met.CAT	23	26
tRNA.Phe.GAA	21	21
tRNA.Pro.AGG	9	48
tRNA.Pro.CGG	3	3
tRNA.Pro.GGG	0	3
tRNA.Pro.TGG	4	13
tRNA.SeC.TCA	0	64
tRNA.Ser.ACT	0	4
tRNA.Ser.AGA	10	13
tRNA.Ser.CGA	4	9
tRNA.Ser.GCT	10	29

Table S6 – continued from previous page

type	filtered	unfiltered
tRNA.Ser.GGA	0	1
tRNA.Ser.TGA	5	8
tRNA.Sup.CTA	0	2
tRNA.Sup.TTA	1	17
tRNA.Thr.AGT	13	46
tRNA.Thr.CGT	4	7
tRNA.Thr.GGT	0	3
tRNA.Thr.TGT	8	13
tRNA.Trp.CCA	7	9
tRNA.Tyr.ATA	0	6
tRNA.Tyr.GTA	17	30
tRNA.Val.AAC	10	13
tRNA.Val.CAC	12	15
tRNA.Val.GAC	1	10
tRNA.Val.TAC	11	16
Unkown/Pseudo	4	27,893
Total	810	32,303

Table S7 - High, medium and low confident results of the homology based pipeline

The combined results of the sequence similarity search, structure homology search and class specific tools at the different cutoff levels (Compare with main text Table 1). Each confidence level contains the RNAs of the previous one, that is high is contained in medium, which again is contained in low. The column *RNA class* contains cisreg-elements: cis-regulatory elements from Rfam/`Infernal`; lncRNA-loci: `Infernal` lncRNA structure loci; the next 7 rows contain (full length) ncRNA genes, miRNA: `BLAST` from miRBase and `miRDeep` predictions; ribozyme: ribozymes from Rfam/`Infernal`; rRNA: ribosomoal RNAs primarily from `RNAmmer`; snRNA and snoRNA: `BLAST` results and results from `Infernal`/Rfam; tRNA: tRNAs tRNAs from `BLAST`; `tRNAscan-SE` and `Infernal`/Rfam; lncRNA-loci: structural loci from larger genes(lncRNAs); other: RNA families from Rfam not belonging to one of the other classes; conflict: conflicts of annotation. Loci are the number of RNA loci of a given class; Families are a subdivision of classes into RNAs with the same name. 12 tRNAs and 15 miRNAs were moved to the medium confident annotation as part of the curation procedure. See text for details. Note that for the final annotation we add the RNA-seq based miRNA candidates, reaching the final total of 3,556 high confident RNA loci, 3,877 medium confident RNA loci and 36,647 low confident RNA loci.

confidence levels	high		medium		low	
RNA class	families	loci	families	loci	families	loci
cisreg	31	139	31	163	33	176
lncRNA	58	58	58	58	58	58
miRNA	321	359	408	566	435	1,670
ribozyme	3	8	3	8	4	9
rRNA	5	185	5	186	5	186
snoRNA	211	638	214	635	217	667
snRNA	10	1,030	10	1,066	10	1,147
tRNA	51	810	51	837	126	32,320
other	7	153	7	158	8	198
conflict	9	11	13	19	22	38
sum	706	3,391	800	3,696	918	36,469

Table S8 - Read supported annotation

High confident annotation supported by reads from the small RNA library. The annotation is given in the first column, the locus in the second, indication of miRDeep support in the third, and the # of reads overlapping with the annotation in the fourth and last column. The # of reads is given as multiple numbers separated by commas in the cases where gaps are observed in the read coverage of the annotation.

annotation	locus	miRDeep	# of reads
SNORA29	1:9392133-9392273:+		23
SNORA20	1:9397984-9398115:+		14
pre-396	1:13208028-13208138:+	miRDeep	13
pre-72	1:16414454-16414564:+	miRDeep	11
tRNA.Leu_TAA	1:23464934-23465017:-		349
pre-412	1:28743959-28744085:-	miRDeep	35
SNORD100	1:34439762-34439839:-		76
SNORD101	1:34440857-34440941:-		46
tRNA.Glu_CTC	1:40973351-40973423:+		223
7SK	1:41992343-41992658:+		17
mir-30c-2	1:57721673-57721804:-	miRDeep	218389
mir-30a	1:57750735-57750842:-		2034837
SNORD50	1:61061227-61061297:-		520
SNORD50	1:61061506-61061580:-		206
8S	1:76147917-76148035:+		33
tRNA.Gln_TTG	1:100519049-100519122:+		121
tRNA.Ala_AGC	1:108245319-108245392:+		151
SNORD58	1:108448181-108448248:-		31
SNORD58	1:108449421-108449490:-		16
SNORD58	1:108450284-108450356:-		49
SCARNA17	1:108870764-108870847:+		74
mir-190a	1:120448003-120448124:-	miRDeep	110
7SK	1:127843475-127843799:+		94
7SK	1:127993024-127993348:+		94
mir-628	1:129013443-129013569:+	miRDeep	3643
8S	1:138677526-138677632:-		41
mir-147	1:140918974-140919081:+	miRDeep	183
tRNA.His_GTG	1:141140248-141140320:-		33
tRNA.His_GTG	1:141140980-141141052:+		10
tRNA.His_GTG	1:141142533-141142605:+		41
pre-420	1:141443593-141443697:+	miRDeep	10
pre-420	1:141539541-141539645:+	miRDeep	10
mir-2366	1:142775303-142775372:-		43
mir-2366	1:142822093-142822162:+		43
tRNA.Ser_GCT	1:145840100-145840182:+		17
tRNA.Glu_TTC	1:157402787-157402859:+		460
SNORD116	1:158000979-158001084:-		1178
SNORD116	1:158003058-158003163:-		1178
SNORD116	1:158006971-158007076:+		1178
SNORD116	1:158009605-158009710:+		1178
mir-122	1:179916326-179916428:-	miRDeep	424083
mir-3591	1:179916341-179916414:+		26
U5	1:180900947-180901063:+		35
U5	1:180909302-180909419:+		70

Table S8 – continued from previous page

annotation	locus	miRDeep	# of reads
tRNA.Gln_CTG	1:181493190-181493262:+		75
SCARNA14	1:182119630-182119767:-		62
SNORD18	1:182228374-182228447:-		11
SNORD18	1:182229574-182229647:-		22
pre-38	1:182942449-182942554:-	miRDeep	11
SNORD127	1:195181257-195181343:+		88
(mir-1285,Metazoa_SRP)	1:199694415-199694718:-		1439
(mir-1285,Metazoa_SRP)	1:200006505-200006808:-		908
(mir-1285,Metazoa_SRP)	1:200101913-200102216:+		1439
7SK	1:203221421-203221745:-		34
tRNA.Lys_CTT	1:208243360-208243433:-		1479
mir-7-1	1:212651377-212651482:-	miRDeep	3792
mir-31	1:224397143-224397251:+	miRDeep	1007
mir-491	1:225810894-225811016:-	miRDeep	177
SCARNA8	1:227405644-227405775:+		80
tRNA.His_GTG	1:231846912-231846984:+		93
mir-101-1	1:242988409-242988514:-	miRDeep	905631
Metazoa_SRP	1:244532194-244532474:-		451
mir-204	1:250527012-250527136:-	miRDeep	25035
pre-189	1:250901263-250901370:-	miRDeep	188
tRNA.Val_TAC	1:261465667-261465740:-		41
tRNA.Val_TAC	1:261617485-261617558:-		41
RNase_MRP	1:264105053-264105311:-		10,136
mir-32	1:280645117-280645245:-	miRDeep	1590
U4	1:285896503-285896584:-		38
mir-455	1:286017366-286017472:+	miRDeep	3608
U4	1:286171495-286171575:-		38
SNORD90	1:297261845-297261956:-		186
mir-181a-2	1:299322119-299322244:+	miRDeep	605747
mir-181b-2	1:299324001-299324107:+	miRDeep	134765
SNORA21	1:299756994-299757130:-		22
SNORA65	1:301872753-301872889:-		17
mir-199b	1:302681802-302681914:-	miRDeep	85103
mir-219-2	1:302806837-302806947:-	miRDeep	5044
mir-2964	1:302806847-302806944:+		17
SNORD62	1:305426339-305426427:+		151
SNORD62	1:305430344-305430432:+		161
U6atac	1:307553517-307553643:-		16
pre-256	1:308789958-308790068:+	miRDeep	33
pre-52	1:311742594-311742704:+	miRDeep	317
mir-126	1:313734668-313734741:+		776939
SNORA17	1:313849903-313850035:+		17
Y_RNA	10:6017459-6017557:+		1743
8S	10:7400166-7400283:+		34
mir-215	10:11786560-11786666:-	miRDeep	404427
mir-194	10:11786858-11786961:-	miRDeep	10876
(mir-664,SNORA36)	10:11890455-11890592:+		1565
mir-194	10:11962849-11962952:+	miRDeep	10876
mir-215	10:11963144-11963250:+	miRDeep	404427

Table S8 – continued from previous page

annotation	locus	miRDeep	# of reads
tRNA.Thr_TGT	10:13422441-13422514:+		29
U6atac	10:13627637-13627763:-		14
mir-181b-1	10:26426842-26426950:-	miRDeep	134693
mir-181a-1	10:26427015-26427122:-	miRDeep	605199
pre-261	10:27752718-27752828:+	miRDeep	69
pre-335	10:27752721-27752831:-	miRDeep	69
mir-24-2	10:31340316-31340445:-	miRDeep	118602
mir-27b	10:31340863-31340995:-	miRDeep	498314
mir-23b	10:31341097-31341224:-	miRDeep	45158
tRNA.Asp_GTC	10:32428442-32428514:+		12
mir-7	10:35113318-35113449:+	miRDeep	6139
pre-203	10:38055761-38055864:+	miRDeep	17
mir-873	10:42020452-42020555:+	miRDeep	121
mir-876	10:42052689-42052770:+		33
mir-876	10:42170916-42170997:-		33
pre-247	10:52762860-52762970:-	miRDeep	42
8S	10:61215653-61215791:+		109
pre-133	11:4056368-4056471:-	miRDeep	118
SNORD102	11:4200342-4200423:+		29
pre-66	11:18917615-18917725:+	miRDeep	16
tRNA.Glu_TTC	11:22627776-22627848:+		473
mir-17	11:66610045-66610152:+	miRDeep	6942
mir-18a	11:66610185-66610293:+	miRDeep	466
mir-19a	11:66610315-66610435:+	miRDeep	204
mir-20	11:66610475-66610600:+	miRDeep	8223
mir-19b-1	11:66610610-66610737:+	miRDeep	1460
mir-92a-1	11:66610749-66610855:+	miRDeep	137767
U3	11:75593883-75594099:+		5350
pre-418	11:75694866-75694976:-	miRDeep	10
pre-37	11:81643713-81643823:+	miRDeep	163
pre-322	11:84694639-84694749:+	miRDeep	11
mir-338	12:1507053-1507178:+	miRDeep	6739
pre-82	12:1513526-1513636:+	miRDeep	10
pre-84	12:1918946-1919056:-	miRDeep	41
SCARNA16	12:4273848-4274036:+		284
SNORD1	12:4809431-4809516:-		43
pre-401	12:9111174-9111284:+	miRDeep	50
pre-401	12:9188722-9188832:-	miRDeep	50
SNORA76	12:14862198-14862331:-		191
SNORND104	12:14862518-14862598:-		607
tRNA.Ala_CGC	12:16466377-16466449:+		10
pre-177	12:18750320-18750430:+	miRDeep	131
pre-228	12:18820612-18820716:-	miRDeep	37
pre-228	12:18959796-18959900:+	miRDeep	37
U2	12:20156277-20156468:+		1650
U2	12:20158962-20159153:+		653
pre-374	12:20165351-20165455:-	miRDeep	12
U2	12:20179351-20179542:-		1654
pre-105	12:23440121-23440225:-	miRDeep	26

Table S8 – continued from previous page

annotation	locus	miRDeep	# of reads
pre-106	12:23443837-23443947:-	miRDeep	16
mir-152	12:24289526-24289631:-	miRDeep	19543
mir-10a	12:24791251-24791382:-	miRDeep	96758
mir-196a-1	12:24836740-24836866:+	miRDeep	4676
tRNA.Gln_TTG	12:25299748-25299820:-		108
pre-116	12:26484651-26484761:-	miRDeep	21
U1	12:35716299-35716463:+		188
U3	12:35722001-35722217:-		118
U3	12:35725693-35725909:-		90
U1	12:35743060-35743224:-		27
U1	12:35836711-35836875:-		26
U3	12:35845116-35845332:+		95
U3	12:35847556-35847772:+		5350
U1	12:35888694-35888858:+		138
U3	12:35915626-35915842:+		106
mir-142	12:36203901-36204031:+	miRDeep	23022
mir-301	12:37079075-37079180:+	miRDeep	199
mir-454	12:37088069-37088181:+	miRDeep	419
mir-21	12:37340355-37340484:+	miRDeep	3710170
pre-263	12:38333492-38333602:-	miRDeep	15
mir-378-2	12:38396699-38396767:+		648978
SNORA17	12:39216248-39216382:-		12
SNORD7	12:41449487-41449584:-		19
tRNA.Gly_GCC	12:41618661-41618732:-		16
mir-365-2	12:45152780-45152887:-	miRDeep	1211
mir-193a	12:45165024-45165144:-	miRDeep	22388
tRNA.Thr_CGT	12:45176015-45176087:-		40
SNORD42	12:46942592-46942659:+		71
SNORD4	12:46945424-46945501:+		27
mir-144	12:47176938-47177042:+	miRDeep	5213
mir-451	12:47177113-47177178:+		342252
mir-423	12:48287007-48287132:-	miRDeep	19142
mir-132	12:50015707-50015829:-	miRDeep	78181
mir-212	12:50016137-50016252:-	miRDeep	1363
mir-497	12:54717009-54717115:+	miRDeep	3757
mir-195	12:54717307-54717412:+	miRDeep	7773
mir-324	12:54860430-54860552:+	miRDeep	745
SNORD10	12:55302065-55302208:-		681
SNORA48	12:55303752-55303887:-		14
tRNA.Gln_CTG	12:55802711-55802783:+		70
tRNA.Leu_TAG	12:55803238-55803320:-		36
tRNA.Arg_TCT	12:55803827-55803914:+		33
tRNA.Ser_CGA	12:55835990-55836072:-		83
tRNA.Thr_AGT	12:55836468-55836542:-		12
U8	12:55869747-55869883:-		170
tRNA.Trp_CCA	12:55877469-55877541:+		21
tRNA.Thr_AGT	12:55878296-55878370:+		16
tRNA.Trp_CCA	12:55896174-55896246:-		16
tRNA.Gly_TCC	12:55896825-55896897:+		97

Table S8 – continued from previous page

annotation	locus	miRDeep	# of reads
tRNA.Pro_CGG	12:55898246-55898318:-		32
tRNA.Thr_AGT	12:55902001-55902075:-		29
tRNA.Ser_AGA	12:55902372-55902454:-		20
mir-744	12:59358202-59358282:+		18948
SNORD49	12:62260434-62260514:-		130
SNORD49	12:62260898-62260981:-		277
pre-27	12:62507588-62507698:-	miRDeep	15
pre-130	13:19940659-19940769:-	miRDeep	13
mir-128-2	13:22859158-22859283:+	miRDeep	21425
mir-26a-1	13:24885233-24885338:-	miRDeep	13767
pre-287	13:26080877-26080987:+	miRDeep	24
SNORA6	13:26315916-26316067:-		45
mir-138a	13:30329388-30329495:+	miRDeep	17626
tRNA.Arg_ACG	13:31794454-31794527:+		18
pre-402	13:33474495-33474605:+	miRDeep	230
mir-425	13:34898091-34898201:-	miRDeep	15153
mir-191	13:34898585-34898714:-	miRDeep	276416
SNORD22	13:36738117-36738230:+		114
Metazoa_SRP	13:37548434-37548734:-		103
mir-135-1	13:37564044-37564148:+	miRDeep	715
let-7g	13:37599003-37599083:+		291780,57
SNORD19	13:38024237-38024316:+		12
SNORD69	13:38026568-38026645:+		27
pre-148	13:44671144-44671254:-	miRDeep	12
pre-376	13:50185395-50185505:-	miRDeep	12
U2	13:66187538-66187729:+		38
SNORA7	13:76061641-76061781:-		99,10
pre-53	13:80688641-80688748:-	miRDeep	89
Metazoa_SRP	13:101964270-101964572:-		26
pre-352	13:108096407-108096517:+	miRDeep	11
mir-15b	13:108388108-108388235:+	miRDeep	3789
mir-16-1	13:108388254-108388383:+	miRDeep	40348
mir-551b	13:115915946-115916036:+		182
Telomerase-vert	13:117009657-117010100:-		48
mir-1224	13:131580810-131580918:+		11369
SNORD66	13:131653411-131653489:+		78
snR39B	13:133974702-133974771:+		172
(mir-1248,SNORA81)	13:133976591-133976769:+		18
SNORA63	13:133977045-133977176:+		79
mir-28	13:135461278-135461387:+	miRDeep	66471
pre-120	13:141240629-141240739:-	miRDeep	12
pre-336	13:149441277-149441383:+	miRDeep	5909
pre-94	13:149704276-149704383:+	miRDeep	11
pre-8	13:152984931-152985034:-	miRDeep	78
pre-110	13:158962868-158962974:-	miRDeep	12
8S	13:167058034-167058159:-		460
pre-419	13:190079476-190079586:-	miRDeep	90
mir-99a	13:191558597-191558703:+	miRDeep	162196
let-7c	13:191559326-191559440:+	miRDeep	575693

Table S8 – continued from previous page

annotation	locus	miRDeep	# of reads
SNORA29	13:199016091-199016230:-		20
mir-155	13:199063326-199063436:-	miRDeep	2415
pre-388	13:199987637-199987747:+	miRDeep	13,12
pre-32	13:202679318-202679417:+	miRDeep	23
mir-802	13:209487882-209487987:+	miRDeep	416
pre-246	13:217449293-217449404:+	miRDeep	13
mir-320	14:6954923-6955025:-	miRDeep	20033
mir-124a-2	14:14442991-14443119:-	miRDeep	248506
tRNA.Ala_TGC	14:29953251-29953323:+		15
pre-171	14:36090834-36090944:-	miRDeep	11
U4	14:42856401-42856542:+		106
SNORD125	14:49440017-49440120:-		49
U3	14:50473396-50473608:-		17
mir-130b	14:53695548-53695654:-	miRDeep	5153
mir-301b	14:53695884-53695986:-	miRDeep	29
mir-185	14:55086510-55086611:+	miRDeep	5093
mir-1306	14:55110942-55111047:+	miRDeep	437
mir-1296	14:72055623-72055724:-	miRDeep	58
tRNA.Ser_TGA	14:76937732-76937814:+		51
mir-346	14:94051819-94051929:-	miRDeep	51
mir-107	14:110407084-110407211:-	miRDeep	8464
8S	14:114893823-114893929:-		23
mir-146b	14:123301748-123301853:+	miRDeep	35006
U8	14:126472413-126472506:-		20
pre-153	14:133619316-133619419:+	miRDeep	1067
pre-237	14:138325820-138325921:-	miRDeep	42
SNORA19	14:140518199-140518333:-		41
tRNA.Ile_TAT	14:143674828-143674921:+		38
mir-4331	14:144183467-144183577:+	miRDeep	929
8S	14:145513928-145514039:+		23
mir-128-1	15:18661601-18661723:-	miRDeep	23589
U4atac	15:34820148-34820274:-		148
tRNA.Glu_TTC	15:35901799-35901871:+		344
pre-58	15:39570811-39570921:-	miRDeep	39
snoU13	15:59535635-59535742:-		243,30
tRNA.Ala_CGC	15:70738453-70738525:+		55
pre-270	15:85376699-85376803:+	miRDeep	50
mir-10b	15:91418988-91419114:+	miRDeep	240821
SNORD70	15:117358105-117358194:+		11
SNORD70	15:117360171-117360258:+		16
SNORD11	15:117372460-117372544:+		11
SNORD51	15:121003093-121003172:+		20
SNORA41	15:121003459-121003592:+		18
mir-375	15:133957529-133957639:+	miRDeep	10523
mir-153-1	15:134369136-134369242:-	miRDeep	934
mir-4334	15:134432978-134433088:+	miRDeep	12
SNORD20	15:145943453-145943534:+		449
GGgCD78_GGgCD79	15:147698071-147698351:-		11,61
pre-2	15:151429178-151429288:+	miRDeep	15

Table S8 – continued from previous page

annotation	locus	miRDeep	# of reads
mir-149	15:154446988-154447116:-	miRDeep	3665
pre-285	16:18362870-18362980:+	miRDeep	23
mir-449	16:36468107-36468232:-	miRDeep	3679
mir-449b	16:36468228-36468334:-	miRDeep	327
mir-582	16:41335739-41335844:+	miRDeep	665
pre-294	16:48458252-48458362:+	miRDeep	27
8S	16:51742263-51742382:-		9170
pre-80	16:57174562-57174672:-	miRDeep	12
pre-209	16:59466880-59466987:+	miRDeep	17
mir-218b	16:59686613-59686725:+	miRDeep	21944
mir-103-1	16:59912587-59912711:+	miRDeep	270663
SNORD13	16:64718747-64718854:-		30
8S	16:65080068-65080177:+		995
mir-146a	16:68394500-68394610:-	miRDeep	16227
mir-146a	16:68488115-68488225:+	miRDeep	16227
tRNA.Lys_TTT	16:81577559-81577632:+		14
mir-383	17:2603729-2603857:-	miRDeep	2363
mir-486-2	17:12191215-12191342:-	miRDeep	15298
SNORD17	17:29798988-29799225:-		41
mir-103-2	17:36149752-36149879:-	miRDeep	270691
SNORD110	17:37442163-37442231:+		38
SNORA51	17:37443010-37443141:+		29
SNORD56	17:37444514-37444596:+		10
SNORD57	17:37444846-37444921:+		326
SNORD119	17:37510785-37510867:+		3603
pre-202	17:39852381-39852485:+	miRDeep	13
pre-202	17:40148163-40148267:+	miRDeep	13
mir-499	17:43571137-43571240:+	miRDeep	597
SNORA39	17:46915809-46915947:-		36
mir-296	17:66288689-66288799:-	miRDeep	1070
pre-207	17:69264140-69264242:-	miRDeep	18
mir-133a-1	17:69274649-69274755:-	miRDeep	1826
mir-1	17:69285391-69285501:-	miRDeep	42097
18S	17:69465617-69467475:+		81,553,69,215,111,1203,229
18S	17:69655363-69657444:+		81,553,69,215,23
pre-346	18:2856055-2856165:+	miRDeep	313
mir-671	18:6286672-6286787:-	miRDeep	245
tRNA.Arg_CCT	18:10741887-10741960:+		303
pre-75	18:11728598-11728708:-	miRDeep	13
mir-490	18:13361965-13362093:-	miRDeep	1781
mir-29b	18:19034808-19034916:+	miRDeep	22614
mir-29a	18:19035213-19035322:+	miRDeep	204964
mir-335	18:19337983-19338104:-	miRDeep	8050
mir-183	18:20030631-20030739:+	miRDeep	21792
mir-96	18:20030875-20030985:+	miRDeep	607
mir-182	18:20035198-20035307:+	miRDeep	23548
mir-129b	18:21181540-21181650:+	miRDeep	21514
mir-592	18:22280525-22280635:-	miRDeep	21
mir-592	18:22467309-22467419:+	miRDeep	21

Table S8 – continued from previous page

annotation	locus	miRDeep	# of reads
Metazoa_SRP	18:23522469-23522694:-		25
8S	18:40957726-40957845:-		34
pre-300	18:46443985-46444095:+	miRDeep	46
mir-196b	18:49836513-49836635:+	miRDeep	11981
mir-196b	18:50037474-50037596:+	miRDeep	11981
mir-148a	18:51216016-51216143:+	miRDeep	117202
SNORA21	18:55201882-55202019:+		26
SNORA5	18:55204668-55204803:+		24
mir-4657	18:55338727-55338830:-	miRDeep	22
SNORA21	18:55424383-55424520:-		25
SNORA52	2:165214-165347:-		18
mir-210	2:339060-339165:+	miRDeep	1814
SNORA54	2:454685-454813:-		71,11
SNORA54	2:455608-455738:-		71,11
pre-345	2:1283992-1284102:+	miRDeep	12
tRNA.Ala_AGC	2:3158776-3158849:+		40
tRNA.Ser_GCT	2:5119555-5119637:-		46
mascRNA-menRNA	2:5813656-5813713:+		95
pre-29	2:5835471-5835576:+	miRDeep	11
mir-194	2:6416746-6416869:+	miRDeep	202346
mir-192	2:6416966-6417076:+	miRDeep	1018006
pre-117	2:7444688-7444792:-	miRDeep	455
U2	2:8235179-8235370:-		931
SNORD22	2:8242098-8242224:-		147
SNORD31	2:8242428-8242499:-		67
SNORD30	2:8242742-8242818:-		229
SNORD29	2:8242979-8243050:-		281
SNORD22	2:8243269-8243397:-		11,10
SNORD28	2:8243747-8243828:-		24
SNORD27	2:8244121-8244208:-		79
SNORD26	2:8244488-8244562:-		50
SNORD25	2:8244780-8244845:-		67
pre-231	2:8531461-8531571:+	miRDeep	14
IRE	2:8983284-8983314:+		63
pre-238	2:9139835-9139937:+	miRDeep	23
pre-164	2:9662219-9662322:+	miRDeep	11
tRNA.Leu_TAA	2:11403671-11403754:-		18
tRNA.Val_TAC	2:11404440-11404513:+		239
tRNA.Val_TAC	2:12562086-12562159:+		153
mir-130a	2:12929867-12929975:-	miRDeep	4528
U2	2:15921985-15922176:+		97
mir-129a	2:20324612-20324740:-	miRDeep	23622
pre-234	2:20325914-20326022:-	miRDeep	26
mir-670	2:20399924-20400024:-		67
mir-1343	2:28433181-28433260:-		3810
SNORA18	2:29018925-29019061:+		63
SNORD14	2:45086051-45086150:+		34
SNORD14	2:45087271-45087368:+		26
pre-416	2:45901729-45901839:+	miRDeep	17

Table S8 – continued from previous page

annotation	locus	miRDeep	# of reads
pre-64	2:45901738-45901848:-	miRDeep	17
pre-212	2:50496457-50496562:-	miRDeep	100
snoU97	2:52020792-52020933:+		13
tRNA.Glu_CTC	2:55171214-55171286:-		224
tRNA.Leu_CAA	2:55171572-55171678:-		20
7SK	2:55842859-55843198:-		19
mir-181d	2:65553470-65553580:-	miRDeep	10575
mir-181c	2:65553652-65553759:-	miRDeep	30244
mir-23a	2:65581837-65581907:+		29591
mir-27a	2:65581985-65582111:+	miRDeep	40952
mir-24-1	2:65582140-65582268:+	miRDeep	119834
8S	2:66027753-66027855:+		38
SNORD41	2:66661515-66661590:+		21
SNORD105	2:69288112-69288199:+		43
mir-199a-2	2:69991757-69991871:-	miRDeep	104100
SNORD37	2:75293902-75293976:+		14
U2	2:79545079-79545270:+		97
mir-340	2:80118818-80118948:+	miRDeep	6773
mir-340	2:80137459-80137589:+	miRDeep	6773
8S	2:81464805-81464907:-		515
pre-129	2:82423653-82423757:-	miRDeep	11
mir-1271	2:83154225-83154303:-		16311
SNORA21	2:83796914-83797051:+		28
tRNA.Arg_TCG	2:84899518-84899591:+		14
pre-218	2:88902969-88903077:+	miRDeep	186
pre-78	2:92577480-92577590:-	miRDeep	10
snoZ39	2:96866368-96866431:+		31
mir-9-2	2:98744537-98744666:-	miRDeep	2116866
mir-3660	2:100272625-100272729:+	miRDeep	13
mir-874	2:145381084-145381189:-	miRDeep	837
SNORD63	2:146235152-146235223:-		84
SNORA74	2:147014069-147014272:+		58
Vault	2:148364673-148364768:+		62
pre-331	2:156771689-156771799:-	miRDeep	55
pre-331	2:156907053-156907163:+	miRDeep	55
pre-262	2:157162716-157162825:-	miRDeep	24
mir-143	2:157344724-157344834:+	miRDeep	3036233
mir-145	2:157346096-157346226:+	miRDeep	26862
mir-378-1	2:157640321-157640450:+	miRDeep	649583
mir-339	3:726697-726800:+	miRDeep	5600
pre-288	3:2177085-2177195:+	miRDeep	19
tRNA.Trp_CCA	3:6645044-6645116:-		25
pre-306	3:9681084-9681186:+	miRDeep	14
SCARNA20	3:10668074-10668205:+		11
mir-590	3:11024977-11025082:+	miRDeep	703
SNORA22	3:17184818-17184953:+		13
pre-312	3:17756702-17756807:+	miRDeep	17
tRNA.Leu_TAG	3:24417883-24417965:+		636
8S	3:28371747-28371861:-		21

Table S8 – continued from previous page

annotation	locus	miRDeep	# of reads
pre-190	3:29052243-29052346:+	miRDeep	106
pre-193	3:29285071-29285181:+	miRDeep	32
mir-365-1	3:29976217-29976326:-	miRDeep	1229
tRNA.Arg_CCT	3:40415535-40415608:-		1006
tRNA.Pro_AGG	3:40423046-40423118:-		80
tRNA.Pro_TGG	3:40424336-40424408:-		55
tRNA.Arg_CCT	3:40427777-40427850:+		209
tRNA.Pro_CGG	3:40429898-40429970:-		30
tRNA.Pro_TGG	3:40444315-40444387:-		59
tRNA.Arg_CCT	3:40451776-40451849:-		280
tRNA.Arg_CCG	3:40453835-40453908:-		16
tRNA.Gly_CCC	3:41343172-41343243:-		276
pre-149	3:42287614-42287729:-	miRDeep	10
SNORD60	3:42352443-42352527:-		54
mir-1842	3:42401464-42401569:-	miRDeep	131
SNORD60	3:42478882-42478966:-		54
let-7a-2	3:44864432-44864524:+		1718483
let-7f-1	3:44864803-44864890:+		847969
let-7d	3:44867261-44867364:+		37040,2511
tRNA.Ser_AGA	3:51160997-51161079:-		17
SNORD89	3:55607594-55607704:+		111
SNORD94	3:61495489-61495624:-		19,55
8S	3:71917648-71917753:-		121
tRNA.Gly_CCC	3:75511798-75511869:+		278
mir-216b	3:90047302-90047408:+	miRDeep	1420
mir-216	3:90058948-90059079:+	miRDeep	56
mir-217	3:90065706-90065824:+	miRDeep	404
Metazoa_SRP	3:106666009-106666299:+		22
U2	3:112777452-112777644:+		58
SNORD53_SNORD92	3:117181361-117181447:-		19
SNORD53_SNORD92	3:117182221-117182305:-		23
SNORD53_SNORD92	3:117191986-117192075:-		31
tRNA.Ala_AGC	3:119064630-119064703:-		194
tRNA.Tyr_GTA	3:119065019-119065108:-		45
mir-1329	3:134560356-134560481:+	miRDeep	241
mir-324	3:139855116-139855238:-	miRDeep	745
pre-140	3:141748056-141748157:+	miRDeep	20
pre-140	3:142177108-142177209:-	miRDeep	20
pre-216	4:1220219-1220329:-	miRDeep	487
mir-30d	4:6713481-6713608:+	miRDeep	793757
mir-30b	4:6717607-6717735:+	miRDeep	25690
8S	4:14502236-14502354:-		742
tRNA.Met_CAT	4:16940604-16940677:-		102
tRNA.Met_CAT	4:17080008-17080081:+		102
mir-599	4:40328414-40328518:+	miRDeep	15
tRNA.Ser_AGA	4:44728560-44728642:-		17
pre-173	4:56174488-56174598:+	miRDeep	25
SNORD87	4:73907576-73907665:+		34
tRNA.Ala_AGC	4:74708280-74708353:-		195

Table S8 – continued from previous page

annotation	locus	miRDeep	# of reads
tRNA.Tyr_GTA	4:74709040-74709134:-		16
mir-124a-1	4:76136452-76136580:-	miRDeep	248508
snoU54	4:82680412-82680477:+		69
tRNA.Pro_CGG	4:90991619-90991691:-		36
tRNA.Gly_GCC	4:96929311-96929382:+		62
tRNA.Glu_CTC	4:96939475-96939547:-		223
tRNA.Gly_TCC	4:96939875-96939947:-		98
tRNA.Leu_CAG	4:96941154-96941237:+		83
SNORA19	4:97338991-97339120:+		40
tRNA.Arg_TCT	4:99284478-99284552:+		259
mir-9-1	4:102543197-102543306:-	miRDeep	2116867
(mir-1940,SCARNA4)	4:102848299-102848430:+		58
SNORA42	4:102853982-102854119:+		16
mir-92b	4:103407086-103407194:-	miRDeep	38218
SNORA58	4:104279837-104279973:-		11
SNORA58	4:104398192-104398328:-		11
SNORA58	4:104403867-104404002:-		420
mir-190	4:104457809-104457892:+		147
tRNA.Gly_CCC	4:108723136-108723207:-		11
tRNA.Val_AAC	4:108736396-108736469:-		20
tRNA.Gly_CCC	4:108899725-108899796:-		10
tRNA.Gly_CCC	4:108901285-108901356:+		10
tRNA.Glu_TTC	4:108904797-108904869:-		98
U1	4:108941896-108942060:+		123
tRNA.Gly_TCC	4:108959166-108959238:-		100
tRNA.Glu_CTC	4:108961246-108961318:-		223
pre-350	4:108993433-108993568:+	miRDeep	69
pre-323	4:117785404-117785514:-	miRDeep	13
SCARNA2	4:121458134-121458546:-		62
pre-317	4:129758551-129758661:-	miRDeep	35
mir-137	4:131567064-131567168:-	miRDeep	2949
pre-227	4:131749035-131749145:+	miRDeep	83
tRNA.Arg_TCT	4:135066900-135066985:-		35
mir-760	4:135067623-135067736:-	miRDeep	738
SNORD21	4:135984518-135984617:-		101
U12	5:3631945-3632095:-		120
pre-266	5:4083319-4083429:-	miRDeep	49
pre-267	5:5388478-5388606:+	miRDeep	28014
SNORD43	5:6217443-6217508:+		3177
SNORD83	5:6221777-6221871:+		120
pre-143	5:9677597-9677723:+	miRDeep	245
pre-344	5:11266526-11266624:-	miRDeep	24
mir-196a	5:19654955-19655063:-	miRDeep	4818
mir-615	5:19690645-19690755:-	miRDeep	13
mir-615	5:20147909-20148019:+	miRDeep	13
mir-148b	5:20388175-20388284:+	miRDeep	4799
tRNA.Ser_CGA	5:23226335-23226417:-		12
(mir-677,SNORD59)	5:23664723-23664798:-		11
pre-213	5:24730557-24730660:-	miRDeep	12

Table S8 – continued from previous page

annotation	locus	miRDeep	# of reads
let-7i	5:30021223-30021308:+		168997,669
pre-154	5:30185285-30185395:-	miRDeep	95
snoU89	5:66190819-66191074:+		21,16
mir-141	5:66193694-66193827:-	miRDeep	17875
mir-200c	5:66194107-66194216:-	miRDeep	60513
U7	5:66218013-66218074:-		88
SCARNA11	5:66482022-66482168:-		62
pre-83	5:77831944-77832054:-	miRDeep	26
8S	5:79210270-79210379:+		109
SNORA2	5:82778922-82779061:+		66
tRNA.Trp_CCA	5:89679121-89679193:-		51
tRNA.Asp_GTC	5:89679830-89679902:-		57
mir-135-2	5:90552041-90552146:-	miRDeep	785
Metazoa_SRP	5:90996693-90997000:+		72,27
mir-331	5:92224977-92225057:-		758
mir-3059	5:100312243-100312353:+	miRDeep	122
SNORD68	6:519451-519540:-		1303
(28S,mir-2904-1,2887-1,4332,5105)	6:863854-871277:-		223,87,51,40283,314213
5_8S_rRNA	6:872545-872698:-		37506
18S	6:874064-876366:-		71100,39
pre-167	6:4245057-4245167:-	miRDeep	137
pre-36	6:4257655-4257765:-	miRDeep	11
pre-14	6:12803963-12804073:+	miRDeep	11
pre-14	6:12953016-12953126:-	miRDeep	11
SNORD71	6:14363473-14363559:-		93
tRNA.Leu_CAG	6:17242879-17242962:+		55
tRNA.Leu_CAG	6:17243236-17243319:-		58
SNORA50	6:18437299-18437435:+		35
SNORA46	6:18447846-18447983:+		244
mir-328	6:25134065-25134175:-	miRDeep	3187,30
pre-54	6:36007855-36007965:-	miRDeep	10
tRNA.Thr_AGT	6:38547148-38547222:+		413
tRNA.Ile_TAT	6:43890847-43890940:+		38
mir-769	6:47579793-47579920:+	miRDeep	3185
mir-769	6:47708729-47708856:+	miRDeep	3185
SNORD23	6:48980942-48981061:+		20
pre-5	6:49993466-49993576:-	miRDeep	15
IRE	6:50097441-50097471:-		57
SNORD33	6:50295419-50295503:+		259
SNORD33	6:50296128-50296209:+		102
SNORD34	6:50296417-50296488:+		26
mir-150	6:50309849-50309969:-	miRDeep	7085
SNORD88	6:51443505-51443596:-		41
SNORD88	6:51445811-51445907:-		18,12
SNORD88	6:51446744-51446839:-		54
pre-411	6:51787897-51788007:-	miRDeep	12
mir-99b	6:51858199-51858306:+	miRDeep	147577
let-7e	6:51858342-51858473:+	miRDeep	49041
mir-125a	6:51858815-51858943:+	miRDeep	84563

Table S8 – continued from previous page

annotation	locus	miRDeep	# of reads
mir-935	6:53057485-53057595:+	miRDeep	364
mir-429	6:58044160-58044284:-	miRDeep	43551
mir-200b	6:58064286-58064392:+	miRDeep	211827
pre-377	6:58626647-58626757:+	miRDeep	12
mir-551a	6:59927705-59927834:+	miRDeep	18
mir-4331	6:60130080-60130190:-	miRDeep	929
mir-2320	6:60147822-60147903:-		933
U5	6:65880855-65880972:+		46
pre-364	6:68330291-68330401:-	miRDeep	16
8S	6:77384713-77384823:-		1268
SNORA73	6:79349748-79349954:+		13,12
SNORD99	6:79408209-79408283:-		482
SNORA61	6:79408843-79408976:-		52
SNORA44	6:79409979-79410110:-		14
SNORD85	6:81192904-81192981:+		17
SNORD103	6:81217634-81217721:+		14
SNORD103	6:81230908-81230995:+		14
SNORD22	6:85243656-85243781:+		147
SNORA55	6:88475890-88476026:-		20
U2	6:98175519-98175710:+		56
mir-1-2	6:99481920-99482030:+	miRDeep	42097
mir-133a-2	6:99485192-99485299:+	miRDeep	1827
mir-187	6:112191783-112191885:-	miRDeep	561
SNORD45	6:127504072-127504144:-		29
SNORD45	6:127504653-127504737:-		72
SNORD45	6:127505298-127505381:-		101
mir-186	6:131558956-131559063:+	miRDeep	39011
mir-101-2	6:135736104-135736209:+	miRDeep	874247
U5	6:153989526-153989642:-		61
SNORD55	6:154026840-154026924:+		207
SNORD46	6:154027354-154027466:+		17,28
SNORD38	6:154028387-154028470:+		55
SNORD38	6:154028882-154028957:+		102
pre-290	6:154077901-154078011:+	miRDeep	26
pre-174	6:155232003-155232113:+	miRDeep	20
mir-30c-1	6:157473594-157473674:-		207877
mir-30e	6:157476637-157476749:-	miRDeep	351618
pre-232	7:4037088-4037201:-	miRDeep	44
pre-370	7:8701416-8701520:+	miRDeep	57
pre-399	7:15776619-15776729:+	miRDeep	21
tRNA.Leu_CAG	7:22101877-22101960:+		85
tRNA.Arg_ACG	7:22160423-22160496:+		33
tRNA.Ala_CGC	7:22174545-22174617:+		22
tRNA.Ala_AGC	7:22182018-22182091:-		59
tRNA.Tyr_GTA	7:22184029-22184119:+		10
tRNA.Gln_TTG	7:22241643-22241715:-		85
tRNA.Gln_TTG	7:22242263-22242335:-		86
tRNA.Ser_TGA	7:22243389-22243471:-		12
tRNA.Trp_CCA	7:22250172-22250244:-		22

Table S8 – continued from previous page

annotation	locus	miRDeep	# of reads
tRNA.Arg_TCG	7:22252163-22252237:+		14
tRNA.Arg_ACG	7:22256613-22256686:+		20
tRNA.Trp_CCA	7:22259203-22259275:-		26
tRNA.Leu_CAG	7:22311394-22311477:+		82
tRNA.Ile_TAT	7:22410948-22411042:-		34
tRNA.Ala_AGC	7:22503237-22503310:-		36
tRNA.Met_CAT	7:22506125-22506198:-		50
tRNA.Ala_AGC	7:22507652-22507725:-		36
tRNA.Tyr_GTA	7:22550696-22550786:-		10
tRNA.Ala_AGC	7:22552727-22552800:+		59
tRNA.Met_CAT	7:22693181-22693253:+		13
tRNA.Val_TAC	7:22724467-22724540:-		69
tRNA.Val_TAC	7:22815548-22815621:-		69
tRNA.Met_CAT	7:22847017-22847089:-		13
tRNA.Gln_CTG	7:22958915-22958987:+		72
pre-403	7:22962927-22963041:+	miRDeep	204
tRNA.Ser_AGA	7:22984473-22984555:-		15
tRNA.Leu_CAA	7:23017562-23017668:+		10
tRNA.Leu_CAA	7:23020204-23020310:-		38
tRNA.Ile_TAT	7:23030090-23030184:+		176
tRNA.Leu_TAA	7:23070887-23070970:-		13
tRNA.Val_AAC	7:23074800-23074873:+		15
tRNA.Gln_TTG	7:23081614-23081686:-		90
tRNA.Ser_GCT	7:24015526-24015608:+		124
tRNA.Met_CAT	7:24191691-24191764:+		47
tRNA.Ile_TAT	7:24248279-24248373:+		28
tRNA.Arg_TCG	7:24251231-24251304:-		14
tRNA.Gln_TTG	7:24278593-24278665:+		50
tRNA.Ala_CGC	7:24313308-24313380:-		16
tRNA.Lys_TTT	7:24319394-24319467:-		14
tRNA.Arg_CCG	7:24372935-24373008:-		12
tRNA.Arg_CCG	7:24412383-24412456:+		12
tRNA.Leu_CAA	7:24425956-24426064:-		164
tRNA.Met_CAT	7:24455767-24455840:-		51
tRNA.Leu_CAA	7:24460066-24460175:+		41
tRNA.Gln_CTG	7:24460587-24460659:-		68
tRNA.Met_CAT	7:24465451-24465524:+		55
tRNA.Met_CAT	7:24470422-24470495:-		51
Metazoa_SRP	7:24476037-24476335:+		11,57
tRNA.Glu_CTC	7:24477379-24477451:+		226
SNORD83	7:27583801-27583884:+		72
SNORD117	7:27587930-27588012:+		64
mir-219	7:29752688-29752795:-	miRDeep	216
7SK	7:31997222-31997523:-		34
pre-326	7:37088391-37088501:+	miRDeep	48
pre-383	7:39611512-39611622:+	miRDeep	10
pre-217	7:41158279-41158382:-	miRDeep	19
mir-206	7:52426428-52426538:+	miRDeep	12856
mir-133b	7:52430276-52430380:+	miRDeep	164

Table S8 – continued from previous page

annotation	locus	miRDeep	# of reads
tRNA.Lys_CTT	7:53580956-53581029:+		1478
mir-184	7:53883615-53883722:+	miRDeep	2678
(mir-1839,SCARNA15,pre-186)	7:57683188-57683337:-	miRDeep	372
tRNA.Arg_TCG	7:59882665-59882738:+		25
mir-9-3	7:59924178-59924309:+	miRDeep	2116579
pre-188	7:69920223-69920333:+	miRDeep	45
tRNA.Tyr_GTA	7:70020294-70020384:+		15
U1	7:70022592-70022756:+		12
SNORD8	7:83112641-83112756:+		48
tRNA.Pro_TGG	7:83338607-83338679:-		138
tRNA.Thr_TGT	7:83340373-83340446:+		40
tRNA.Leu_TAG	7:83343855-83343937:-		39
tRNA.Thr_TGT	7:83350085-83350158:+		41
RNaseP_nuc	7:83579872-83580200:+		101
pre-25	7:98873377-98873487:-	miRDeep	13
SNORA21	7:102131353-102131490:-		145
pre-389	7:106158334-106158444:-	miRDeep	23
SCARNA13	7:124000306-124000582:-		11,57
mir-345	7:128658276-128658390:+	miRDeep	53
SNORD113	7:132020059-132020134:+		16
mir-493	7:132069386-132069497:-	miRDeep	2028
mir-432	7:132078986-132079099:+	miRDeep	5096
mir-136	7:132079193-132079275:+		1734
mir-431	7:132097565-132097656:+		420
mir-127	7:132099546-132099651:+	miRDeep	39170
SNORD113	7:132107066-132107131:+		44
SNORD114	7:132112041-132112118:-		49
SNORD114	7:132118155-132118227:-		60
SNORD113	7:132120108-132120181:-		36
SNORD113	7:132124867-132124942:-		19
7SK	7:134400748-134401079:+		41,222
mir-95	8:4277219-4277329:+	miRDeep	2074
mir-218	8:15443751-15443857:-	miRDeep	21883
pre-369	8:16329028-16329113:-	miRDeep	14
mir-574	8:31672442-31672552:+	miRDeep	1477
pre-340	8:40584084-40584194:-	miRDeep	11
Metazoa_SRP	8:43400952-43401251:+		29
28S	8:57211651-57216538:-		51,194,132,475,39
U1	8:75621457-75621621:+		80
SNORD73	8:82595078-82595154:-		18
pre-17	8:87897276-87897386:+	miRDeep	19
mir-302a	8:115200384-115200453:+		26
U11	8:121660637-121660771:-		63
SNORA76	8:133590840-133590973:+		189
pre-413	8:139758089-139758199:+	miRDeep	45
U2	8:147376160-147376350:+		46
SNORA3	9:778833-778963:-		26
SNORA3	9:951345-951475:-		26
mir-139	9:7935614-7935718:-	miRDeep	6115

Table S8 – continued from previous page

annotation	locus	miRDeep	# of reads
pre-176	9:7940493-7940594:-	miRDeep	14
SNORD15	9:10632575-10632730:-		26
mir-326	9:10800183-10800310:-	miRDeep	213
mir-708	9:15120459-15120593:-	miRDeep	13823
U2	9:18490302-18490490:+		28
SNORA40	9:29684154-29684282:+		10,10
(mir-4336,SNORA18,pre-410)	9:29685340-29685493:+	miRDeep	63
SNORD5	9:29685613-29685688:+		33
snoZ40	9:29687501-29687574:+		89
SNORA32	9:29688454-29688576:+		162
snoU2-30	9:29696263-29696327:-		23
snoU2_19	9:29696476-29696556:-		14
pre-343	9:31199328-31199432:+	miRDeep	12
mir-34c	9:44166861-44166964:-	miRDeep	162636
mir-34b	9:44167416-44167521:-	miRDeep	1179
mir-125b-1	9:54334895-54335021:-	miRDeep	436309
let-7a-1	9:54378041-54378177:-	miRDeep	1718168
mir-100	9:54383366-54383489:-	miRDeep	235012
SNORD14	9:55306103-55306190:+		631
SNORD14	9:55306521-55306607:+		454
SNORD14	9:55307407-55307491:+		47
SNORD14	9:55407917-55408004:+		631
SNORD14	9:55408335-55408421:+		454
SNORD14	9:55409221-55409305:+		47
7SK	9:68662049-68662359:-		54
mir-135b	9:72339082-72339188:-	miRDeep	621
mir-135b	9:72461631-72461737:+	miRDeep	621
mir-653	9:80161133-80161239:-	miRDeep	330
U2	9:82020503-82020694:-		1335
8S	9:87804693-87804800:+		84
SNORD93	9:101043623-101043697:+		28
SNORD29	9:106203651-106203715:-		261
Y_RNA	9:120225377-120225490:+		3360
Y_RNA	9:120228798-120228900:-		1210
Y_RNA	9:120251507-120251604:-		2358
Y_RNA	9:120265589-120265670:-		12774
Metazoa_SRP	9:124189423-124189697:+		11,11
mir-3120	9:126099449-126099562:+		13
mir-214	9:126099456-126099581:-	miRDeep	1054
mir-199a-1	9:126105233-126105340:-	miRDeep	104119
SNORD81	9:127779998-127780077:-		87
SNORD47	9:127780220-127780296:-		84
SNORD80	9:127780916-127781004:-		78
SNORD79	9:127781171-127781255:-		34
SNORD78	9:127781580-127781660:-		27
SNORD44	9:127781886-127781955:-		18
SNORD76	9:127782592-127782676:-		89
SNORD75	9:127782829-127782893:-		16
SNORD74	9:127783400-127783479:-		141

Table S8 – continued from previous page

annotation	locus	miRDeep	# of reads
mir-488	9:130508339-130508422:-		81
U5	9:145656544-145656660:+		20
mir-205	9:146808270-146808376:-	miRDeep	1564
mir-29b-2	9:148552424-148552534:+	miRDeep	23300
mir-29c	9:148552967-148553096:+	miRDeep	31949
SNORD52	GL892492.1:6247-6333:+		10
mir-7-3	GL892815.1:39649-39773:+	miRDeep	3832
mir-140	GL892841.1:107249-107381:+	miRDeep	174757
mir-26b	GL892871.2:55997-56081:+	miRDeep	112187
tRNA.Thr_TGT	GL892933.2:85434-85507:-		41
tRNA.Leu_TAG	GL892933.2:88262-88344:+		39
tRNA.Thr_TGT	GL892933.2:91892-91965:-		40
tRNA.Leu_TAG	GL892933.2:125684-125766:-		34
tRNA.Thr_TGT	GL892933.2:138540-138613:+		42
tRNA.Tyr_GTA	GL892933.2:139830-139920:+		13
pre-251	GL892983.1:13322-13410:-	miRDeep	94
SNORA65	GL893103.2:5211-5347:-		17
SNORD96	GL893304.1:60595-60682:+		93
pre-345	GL893360.2:28349-28459:+	miRDeep	12
mir-218	GL893382.1:26741-26847:-	miRDeep	21883
tRNA.Glu_TTC	GL893609.2:24144-24216:+		344
SNORD72	GL893678.1:24710-24790:-		14
mir-455	GL893885.2:75353-75459:+	miRDeep	3608
SNORA48	GL893964.1:8572-8707:+		14
SNORD10	GL893964.1:10253-10396:+		681
mir-151	GL893970.1:7086-7166:+		169617
SNORD4	GL894032.2:19146-19223:-		27
SNORD42	GL894032.2:21820-21887:-		71
mir-302a	GL894213.2:15691-15760:-		26
mir-412	GL894231.1:6707-6831:-	miRDeep	447
mir-377	GL894231.1:10058-10127:-		238
mir-154b	GL894231.1:12239-12349:-	miRDeep	113
mir-323b	GL894231.1:15847-15967:-	miRDeep	1999
mir-485	GL894231.1:16592-16716:-	miRDeep	2711
mir-487a	GL894231.1:20428-20538:-	miRDeep	15
mir-3959	GL894231.1:20884-20986:-	miRDeep	187
mir-655	GL894231.1:23048-23153:-	miRDeep	649
mir-539	GL894231.1:25676-25807:-	miRDeep	151
mir-381	GL894231.1:27123-27232:-	miRDeep	368183
mir-376a	GL894231.1:30842-30947:-	miRDeep	78
mir-376b	GL894231.1:31290-31395:-	miRDeep	246
mir-376a-2	GL894231.1:31662-31765:-	miRDeep	992
mir-376c	GL894231.1:32020-32125:-	miRDeep	2534
mir-184	GL894254.2:214188-214295:-	miRDeep	2678
pre-299	GL894329.1:53735-53845:+	miRDeep	17
mir-138-2	GL894339.1:3058-3194:-	miRDeep	20466
SNORA42	GL894404.1:13996-14130:-		31
pre-402	GL894542.2:9079-9189:-	miRDeep	230
pre-388	GL894613.1:37133-37243:+	miRDeep	13,12

Table S8 – continued from previous page

annotation	locus	miRDeep	# of reads
mir-486-2	GL894726.2:3452-3579:-	miRDeep	15298
SNORD57	GL894920.1:21072-21147:-		326
SNORD56	GL894920.1:21397-21479:-		10
SNORA51	GL894920.1:22852-22983:-		29
SNORD110	GL894920.1:23762-23830:-		38
U3	GL894932.2:179534-179746:+		17
mir-652	GL894953.1:52739-52847:-	miRDeep	1008
SNORD90	GL894970.2:21667-21778:+		186
mir-190	GL894998.2:17130-17213:-		147
SNORA58	GL894998.2:64887-65023:+		11
pre-179	GL895128.2:107902-108012:+	miRDeep	11
mir-127	GL895236.1:17227-17332:-	miRDeep	39170
tRNA.Ala_TGC	GL895275.1:7928-8000:-		18
tRNA.Pro_TGG	GL895275.1:25604-25676:+		59
tRNA.Leu_AAG	GL895275.1:26440-26522:-		12
mir-30a	GL895276.2:200545-200652:+		2034837
mir-30c-2	GL895276.2:226479-226610:+	miRDeep	218389
mir-542	GL895293.1:3335-3441:+	miRDeep	20486
mir-450c	GL895293.1:4042-4144:+	miRDeep	6804
mir-450a	GL895293.1:4172-4278:+	miRDeep	5382
mir-450b	GL895293.1:4342-4446:+	miRDeep	9205
8S	GL895387.1:30627-30746:-		15
SNORD20	GL895502.2:19253-19334:-		449
SNORD82	GL895502.2:23980-24050:-		294
pre-9	GL895563.1:52448-52548:-	miRDeep	11
SNORD22	GL895583.1:53128-53253:+		147
18S	GL895807.1:895-2821:-		123,76,80,12
mir-1307	GL895853.2:46345-46454:-	miRDeep	4765
mir-30b	GL895891.2:156248-156376:-	miRDeep	25690
mir-30d	GL895891.2:160375-160502:-	miRDeep	793214
mir-26a	GL895994.1:40611-40734:+	miRDeep	360151
mir-4338	GL896017.1:115005-115107:-	miRDeep	18
pre-406	GL896148.1:24082-24192:+	miRDeep	12
SNORD111	GL896221.1:9869-9948:+		71
SNORD111	GL896221.1:17283-17376:+		46
pre-300	GL896241.2:16225-16335:+	miRDeep	46
SNORD12	GL896253.1:2711-2801:+		169
SNORA21	GL896261.1:10011-10148:+		26
tRNA.Arg_ACG	GL896267.1:4106-4179:-		64
pre-254	GL896292.1:11850-11951:-	miRDeep	45
mir-330	GL896302.1:17704-17812:-	miRDeep	4092
pre-329	GL896425.1:1726-1817:-	miRDeep	9234
SNORA21	GL896426.1:64213-64350:-		680
tRNA.Cys_GCA	GL896426.1:74312-74384:+		14
SNORD33	GL896454.1:11145-11229:+		259
SNORD33	GL896454.1:11915-11996:+		102
snoU90	GL896502.1:1579-1672:+		19
SNORA64	GL896557.1:11836-11971:+		48
pre-396	JH118469.1:91787-91897:-	miRDeep	13

Table S8 – continued from previous page

annotation	locus	miRDeep	# of reads
pre-379	JH118486.1:3042-3147:+	miRDeep	25
pre-154	JH118494.1:36713-36823:-	miRDeep	95
mir-885	JH118519.1:42281-42364:-	miRDeep	3789
mir-28	JH118523.1:169138-169247:+	miRDeep	66471
tRNA.Arg_TCG	JH118533.1:10060-10133:+		14
mir-148a	JH118567.1:80904-81031:+	miRDeep	117202
pre-331	JH118582.1:99935-100045:+	miRDeep	55
pre-370	JH118585.1:53392-53496:+	miRDeep	57
pre-202	JH118620.1:126723-126827:+	miRDeep	13
8S	JH118636.1:424214-424328:-		21
pre-343	JH118644.1:36242-36346:-	miRDeep	12
mir-449	JH118654.1:238120-238245:-	miRDeep	3679
mir-15a	JH118724.1:70400-70505:+	miRDeep	4961
mir-16-2	JH118724.1:70556-70635:+		40351
pre-218	JH118729.1:80962-81070:+	miRDeep	186
SNORA29	JH118804.1:110036-110175:+		20
mir-155	JH118804.1:133165-133275:-	miRDeep	2415
pre-297	JH118806.1:23331-23441:-	miRDeep	23
pre-213	JH118928.1:45769-45872:-	miRDeep	12
mir-124a-1	JH118951.1:125787-125915:-	miRDeep	248508
pre-373	X:8905439-8905549:-	miRDeep	128
pre-373	X:9012901-9013011:-	miRDeep	128
tRNA.Val_TAC	X:16319629-16319702:-		194
pre-39	X:21570186-21570293:-	miRDeep	12
mir-221	X:45274853-45274983:-	miRDeep	39493
mir-222	X:45275592-45275724:-	miRDeep	5535
mir-532	X:48632015-48632120:+	miRDeep	21305
mir-188	X:48632372-48632474:+	miRDeep	88
mir-500	X:48637979-48638086:+	miRDeep	4232
mir-362	X:48638452-48638558:+	miRDeep	2302
mir-500	X:48639757-48639841:+		1015
mir-660	X:48640811-48640891:+		3611
mir-502	X:48643194-48643304:+	miRDeep	140
pre-31	X:51475429-51475547:-	miRDeep	21
mir-98	X:51800863-51800943:-		31227
let-7f	X:51801727-51801810:-		846424
pre-276	X:53138293-53138402:+	miRDeep	356
mir-1468	X:56757034-56757139:-	miRDeep	32329
mir-676	X:63280896-63281016:+	miRDeep	1507
mir-421	X:67425031-67425109:-		538
mir-374b	X:67425182-67425294:-	miRDeep	5019
mir-374c	X:67425197-67425267:+		12
mir-545	X:67530180-67530288:-	miRDeep	161
mir-374a	X:67530356-67530472:-	miRDeep	12646
mir-325	X:69436715-69436820:+	miRDeep	261
mir-384	X:69515818-69515925:+	miRDeep	1121
mir-325	X:69673007-69673112:-	miRDeep	261
mir-361	X:78804769-78804895:-	miRDeep	4327
pre-198	X:94852384-94852489:+	miRDeep	156

Table S8 – continued from previous page

annotation	locus	miRDeep	# of reads
mir-1912	X:108629454-108629564:+	miRDeep	11
mir-1264	X:108630535-108630645:+	miRDeep	26
mir-1298	X:108691594-108691712:+	miRDeep	219
mir-448	X:108806657-108806773:+	miRDeep	19
SNORA69	X:113433466-113433598:+		42
8S	X:114189164-114189281:-		34
U2	X:117257285-117257476:-		28
mir-2483	X:117608185-117608290:-	miRDeep	2508
mir-363	X:126199329-126199460:-	miRDeep	17794
mir-92a-2	X:126199495-126199602:-	miRDeep	127873
mir-19b-2	X:126199633-126199743:-	miRDeep	1454
mir-20b	X:126199761-126199866:-	miRDeep	1491
mir-18b	X:126200006-126200113:-	miRDeep	38
mir-106a	X:126200171-126200273:-	miRDeep	542
SNORD61	X:127897892-127897966:+		106
mir-504	X:129654205-129654285:+		605
mir-505	X:130835628-130835749:-	miRDeep	810
mir-506	X:137160417-137160520:-	miRDeep	30
mir-508	X:137168898-137169008:-	miRDeep	16
mir-509	X:137225782-137225884:-	miRDeep	18
mir-224	X:140860045-140860155:-	miRDeep	118
mir-452	X:140861099-140861204:-	miRDeep	451
mir-105-1	X:141091488-141091591:-	miRDeep	689
mir-105-2	X:141093604-141093708:-	miRDeep	45
pre-35	X:143497300-143497410:-	miRDeep	108

Table S9 - Duplicate ncRNA genes

Number of high confident ncRNA annotations marked as duplicates, that is, an annotation with 100% identical sequence is found somewhere in the genome. The number of duplicated annotations are grouped by name. For example, the 169 duplicated U6 annotations will not all have the same sequence, but rather be identical in pairs of two in most cases. (The table is designed in two columns.)

# duplicate loci	name	# duplicate loci	name
169	U6	58	tRNA.Glu_TTC
23	GP_knot1	23	tRNA.Asp_GTC
21	7SK	21	tRNA.Lys_TTT
20	8s_rRNA	19	U1
18	tRNA.Cys_GCA	16	tRNA.Met_CAT
14	tRNA.Phe_GAA	13	tRNA.Ile_AAT
12	IRE	12	SNORA31
12	SNORA70	12	tRNA.Asn_GTT
12	tRNA.Gly_GCC	11	tRNA.Val_CAC
10	tRNA.Lys_CTT	10	tRNA.Thr_AGT
9	tRNA.His_GTG	9	tRNA.Pro_AGG
8	Histone-3-prime-UTR	8	K_chan_RES
8	tRNA.Ala_AGC	8	tRNA.Ser_AGA
8	tRNA.Ser_GCT	8	U2
7	tRNA.Arg_ACG	7	tRNA.Glu_CTC
6	SNORD14	6	tRNA.Gln_CTG
6	tRNA.Leu_AAG	6	tRNA.Tyr_GTA
6	U4	5	tRNA.Leu_CAG
5	tRNA.Val_AAC	4	SNORA11
4	SNORA19	4	SNORA3
4	SNORD116	4	SNORD33
4	tRNA.Ala_TGC	4	tRNA.Gly_CCC
4	tRNA.Leu_TAG	4	tRNA.Thr_TGT
4	tRNA.Trp_CCA	4	tRNA.Val_TAC
4	U3	3	pre-202
3	pre-331	3	SNORA21
3	SNORA58	3	tRNA.Arg_CCG
3	tRNA.Gln_TTG	3	tRNA.Gly_TCC
3	tRNA.Pro_CGG	3	tRNA.Pro_TGG
2	GABA3	2	GGoACA7
2	mir-1249	2	mir-124a-1
2	mir-127	2	mir-135b
2	mir-146a	2	mir-148a
2	mir-155	2	mir-184
2	mir-1895	2	mir-190
2	mir-194	2	mir-196b
2	mir-2053	2	mir-2113
2	mir-215	2	mir-218
2	mir-2366	2	mir-28
2	mir-302a	2	mir-302c
2	mir-30a	2	mir-30b
2	mir-30c-2	2	mir-324
2	mir-325	2	mir-340
2	mir-3666	2	mir-449

Table S9 – continued from previous page

# duplicate loci	name	# duplicate loci	name
2	mir-455	2	mir-486-2
2	mir-568	2	mir-592
2	mir-615	2	mir-769
2	mir-876	2	pre-14
2	pre-140	2	pre-154
2	pre-213	2	pre-218
2	pre-228	2	pre-300
2	pre-343	2	pre-345
2	pre-370	2	pre-373
2	pre-388	2	pre-396
2	pre-401	2	pre-402
2	pre-420	2	SCARNA18
2	SNORA15	2	SNORA25
2	SNORA29	2	SNORA40
2	SNORA42	2	SNORA48
2	SNORA5	2	SNORA51
2	SNORA61	2	SNORA65
2	SNORA67	2	SNORA7
2	SNORA72	2	SNORA75
2	SNORA84	2	SNORD10
2	SNORD103	2	SNORD110
2	SNORD112	2	SNORD123
2	SNORD124	2	SNORD20
2	SNORD22	2	SNORD4
2	SNORD42	2	SNORD56
2	SNORD57	2	SNORD60
2	SNORD77	2	SNORD86
2	SNORD90	2	tRNA.Ala_CGC
2	tRNA.Arg_TCG	2	tRNA.Arg_TCT
2	tRNA.Asp_ATC	2	tRNA.Ile_TAT
2	tRNA.Leu_CAA	2	tRNA.Undet_???
2	U11	2	U5
2	U6atac	2	U8
2	mir-1285,Metazoa_SRP		

Table S10 - Genic context of the high confident annotations

Each annotated locus within 10,000 nucleotides of a protein coding gene has been marked a gene context (otherwise the annotation is marked as *intergenic*). In some cases an annotated locus is marked contexts of multiple protein genes, in which case the annotation counts as having a *multi gene* context in the table. Annotations overlapping protein coding genes, but not covered over 50% of the annotation length by any exon (coding or not coding) are marked as intronic. Annotation are marked as *coding* when at least 50% of the annotation is covered by a protein coding exon. Annotations are marked as 3' UTR or 5' UTR when found within 10,000 nt of the start or end of the protein coding gene, but outside the coding sequence. All non-intergenic annotations are marked as sense or anti-sense to the protein coding gene with which it overlaps. See the main paper Table 2 for an explanation of row labels.

RNA class	# loci	intergenic	multi gene	sense				antisense			
				coding	intronic	5'UTR	3'UTR	coding	intronic	5'UTR	3'UTR
cisreg-elements	139	31	37	25	10	22	11	0	0	3	0
lncRNA-loci	58	46	7	0	0	0	1	0	0	4	0
miRNA	369	172	53	1	62	21	14	3	21	4	18
putative-miRNA	155	55	26	0	40	6	3	3	11	9	2
ribozyme	8	4	1	0	1	0	0	0	0	2	0
rRNA	185	122	9	0	16	2	3	0	29	2	2
snoRNA	638	218	154	0	138	21	12	0	46	19	30
snRNA	1030	621	49	0	120	29	31	0	114	32	34
tRNA	810	426	73	0	73	45	49	0	72	41	31
other	153	101	6	0	15	3	6	0	17	2	3
conflicts	11	2	3	0	3	1	0	0	0	1	1
sum	3556	1798	418	26	478	150	130	6	310	119	121

Table S11 - Clustering at different distances

Clustered RNAs of select classes at different clustering distances. All pairs of high confident annotations within the clustering distance of each other on the same strand, were used to form clusters by single linkage. In the last row we show the total number of RNAs in clusters at a given cutoff and the number of clusters in parenthesis. Only ncRNAs annotated as miRNAs, tRNAs, snoRNAs or "conflicts" are considered. Interclass clusters are allowed, e.g. clusters containing both miRNA and tRNAs.

RNA class/Clustering distance	50	100	200	500	1,000	10,000
miRNA	21	41	55	85	101	153
tRNA	1	1	3	44	100	278
snoRNA	0	2	23	76	125	226
conflicts	0	0	1	2	3	4
all	22(8)	44(18)	82(36)	207(86)	329(133)	661(208)

Table S12a - `RNAz` genic contexts and conservation in other organisms

Results of the search for *de novo* structured RNA loci with `RNAz` version 2.1, based on the 21 way multiZ alignment of pig versus 20 other vertebrate genomes. Cutoff is set at a p-score of 0.9 which results in 83,859 strand independent loci. Shown in the table is the genic context of the `RNAz` annotations. This table should be compared with Table S10 above.

	# loci	intergenic	multi gene	coding	intronic	5'UTR	3'UTR
all	83,859	49,117	5,543	1,009	18,999	4,259	4,932

Table S12b - Sequence conservation in other organisms `RNAz`

Shown in the table is the number of loci conserved by 80% sequence id in at least N other organisms, (N=1, 5, 15). This table should be compared to Table 5 of the main text. Note, that about 20,000 RNAz loci are unconserved in sequence in even 1 organism at the chosen cutoff of 80% sequence identity. In fact we observe RNAz loci with an average pair wise sequence identity of down to 50%, which is the lower limit imposed by the `RNAz` windowing treatment of the multiple alignments. There is, in theory, no lower limit to the observed sequence identity in a structurally conserved locus; only a practical limit, which in the present case is imposed by the sequence based multiple alignments and the `RNAz` window procedure.

all	83,859
conserved by sequence (80%id) in 1 or more organisms	64,442
conserved by sequence (80%id) in 5 or more organisms	24,818
conserved by sequence (80%id) in 15 or more organisms	2,625

Table S13 - Lineage specific structured RNA loci within the high confident annotation

Loci within the high confident annotation found to be lineage specific (at least 60% sequence identity to pig when found within the Laurasiatherian branch/lineage, at most 30% sequence identity when found outside the lineage. The third column contains the sequence identity in % between pig and other species within the lineage.

locus	annotation	sequence identity to pig within the lineage
chr1:311742594-311742704	putative-miRNA|pre-52	
chr3:115373181-115373254	snoRNA|SNORD99	
chr3:115566580-115566725	snoRNA|SNORA6	
chr5:60015709-60015901	snoRNA|SNORA23	turTru1:86.9%,bosTau5:84.3%
chr6:60147822-60147903	miRNA|mir-2320	turTru1:87.6%,bosTau5:96.2%
chr6:64160185-64160312	snoRNA|SNORA2	
chr7:94873024-94873118	snoRNA|SNORA22	turTru1:75.5%,bosTau5:65.9%
chr9:106203651-106203715	snoRNA|SNORD29	
chr9:118790552-118790624	snoRNA|SNORD29	
chr9:123184151-123184216	snoRNA|SNORD25	
chr11:4056368-4056471	putative-miRNA|pre-133	
chr12:23440121-23440225	putative-miRNA|pre-105	
chr12:23443837-23443947	putative-miRNA|pre-106	
chr13:126830485-126830607	snoRNA|SNORA81	canFam2:77.8%,turTru1:85.2%,felCat4:72.1%
chr13:133950936-133951059	snoRNA|SNORA27	
chr13:153238001-153238055	snoRNA|SNORD12	
chr14:128944611-128944760	snoRNA|SNORA6	
chr14:1787080-1787166	snoRNA|SNORD12	
chr15:153983923-153983973	snoRNA|SNORD49	
chr16:52043171-52043255	snoRNA|SNORD55	
chrX:26820119-26820215	Vault|Vault	
chrX:54752507-54752576	miRNA|mir-1949	
chrX:72393306-72393377	snoRNA|SNORD77	

Table S14 - miRNA clusters in human and conservation of clustered miRNAs in pig

miRNA clusters in human with at least 4 miRNAs. First column, the chromosome in human. Second column, a list of the human miRNAs in the cluster. Third column, the chromosome or scaffold in the pig genome containing the cluster. Fourth column, the miRNAs conserved in the human-pig pairwise alignment with at least 80% sequence identity. Fifth column, the miRNAs in the cluster found by high confident BLAST against miRBase.

Table on next page

hg19	miRNA in human miRNA cluster	susScr102	miRNAs in human pig pairwise alignment	miRNAs identified by high confident `BLAST`
19	512−1 512−2 1323 498 520e 515−1 519e 520f 515−2 519c 1283−1 520a 526b 519b 525 523 518f 520b 518b 526a−1 520c 518c 524 517a 519d 521−2 520d 517b 520g 516b−2 526a−2 518e 518a−1 518d 516b−1 518a−2 517c 520h 521−1 522 519a−1 527 516a−1 1283−2 516a−2 519a−2			
14	379 411 299 380 1197 323a 758 329−1 329−2 494 1193 543 495 376c 376a−2 654 376b 376a−1 300 1185−1 1185−2 381 487b 539 889 544a 655 487a 382 134 668 485 323b 154 496 377 541 409 412 369 410 656	GL894231.1	376c 376a−2 654 376b 376a−1 1185−1 1185−2 381 539 544a 655 487a 382 134 485 323b 154 496 377 541 412	412 377 154b 323b 485 487a 544a 539 381 376a 376b 376a−2 376c
14	493 337 665 431 433 127 432 136	7	493 665	493 432 136 3071 431
X	532 188 500a 362 501 500b 660 502	X	532 188 500a 362 500b 660 502	532 188 500 362 500 660
9	3689c 3689a 3689d−1 3689b 3689d−2 3689e 3689f			
13	17 18a 19a 20a 19b−1 92a−1	11	17 18a 19a 20a 19b−1 92a−1	17 18a 19a 20a 19b−1 92a−1
X	363 92a−2 19b−2 20b 18b 106a	X	363 92a−2 19b−2 20b 18b 106a	363 92a−2 19b−2 20b 18b 106a
X	450b 450a−1 450a−2 542 503 424	GL895293.1	450b 450a−1 450a−2 542	542 3601 450c 450a 450b
X	892c 890 888 892a 892b 891b			
4	367 302d 302a 302c 302b	8	367 302d 302a 302c 302b	302c 302a 367
X	513a−2 506 507 508	X	507	
X	514b 509−2 509−3 509−1	X	514b 509−2 509−3 509−1	
X	510 514a−1 514a−2 514a−3	X	514a−1 514a−2 514a−3	

Table S15 - Comparison of presented annotation and Ensembl version 68

Comparison of the ensembl annotation with our homology based annotation. Classification of RNAs according to Ensembl. Overlaps of all non-protein coding transcripts from Ensembl with our high confident annotation in column 2. Notice that our annotation may differ from that of Ensembl, e.g. 7 miRNAs from Ensembl are in fact tRNAs. The third column contains the number of not protein coding transcripts from Ensembl, that are not found even in our low confident annotation. A direct comparison of our annotations with those from Ensembl is difficult because gene names are often missing from the ncRNAs of Ensembl version 68.

	Ensembl	Shared with high confident	Missing from low confident
miRNA	877	357	413
misc-RNA	185	146	7
rRNA	171	130	40
snoRNA	640	549	60
snRNA	1,092	978	2
total	2,965	2,160	522

Table S16 - Command lines used for the annotation pipeline

cmdline	comments
BLAST and formatdb	
formatdb -p F -n susScr10200.fa -i susScr10200.fa -o T	blast database split in multiple files
blastall -p blastn -F F -e 0.1 -z 2700000000 -d susScr10200.fa -i db.fa -o blastout	
Infernal	See Table S17
tRNAscan-SE -H -y -o out -f sec -m stat fasta	
rnammer -S euk -m lsu,ssu,tsu -xml xml -gff gff -h hmmreport < fasta	
snoStrip	
RNAzpreprocessing and RNAz	
rnazWindow.pl -window=120 -slide=40 -max-masked=1.0 -min-seq=3 -max-seq=6 > window.maf <maf<br>RNAz -dinucleotide -predict-strand -both-strands -cutoff=0.3 window.maf > rnaz	

Table S17 - `Infernal` **command lines**

command line
cmsearch -Z 5400 -T 13.60 RF00001.cm fasta
cmsearch -Z 5400 -T 34.00 -g –fil-no-hmm RF00002.cm fasta
cmsearch -Z 5400 -T 23.80 RF00003.cm fasta
cmsearch -Z 5400 -T 39.27 RF00004.cm fasta
cmsearch -Z 5400 -T 20.40 -g –fil-no-hmm RF00005.cm fasta
cmsearch -Z 5400 -T 24.65 RF00006.cm fasta
cmsearch -Z 5400 -T 30.60 RF00007.cm fasta
cmsearch -Z 5400 -T 18.70 –fil-no-hmm RF00008.cm fasta
cmsearch -Z 5400 -T 17.00 RF00009.cm fasta
cmsearch -Z 5400 -T 32.30 -g RF00010.cm fasta
cmsearch -Z 5400 -T 76.50 RF00011.cm fasta
cmsearch -Z 5400 -T 56.10 RF00012.cm fasta
cmsearch -Z 5400 -T 17.00 RF00013.cm fasta
cmsearch -Z 5400 -T 29.75 RF00014.cm fasta
cmsearch -Z 5400 -T 34.00 RF00015.cm fasta
cmsearch -Z 5400 -T 19.55 RF00016.cm fasta
cmsearch -Z 5400 -T 45.05 -g RF00017.cm fasta
cmsearch -Z 5400 -T 29.75 -g RF00018.cm fasta
cmsearch -Z 5400 -T 17.85 RF00019.cm fasta
cmsearch -Z 5400 -T 33.15 RF00020.cm fasta
cmsearch -Z 5400 -T 24.65 RF00021.cm fasta
cmsearch -Z 5400 -T 34.00 RF00022.cm fasta
cmsearch -Z 5400 -T 24.65 RF00023.cm fasta
cmsearch -Z 5400 -T 22.10 RF00024.cm fasta
cmsearch -Z 5400 -T 14.45 RF00025.cm fasta
cmsearch -Z 5400 -T 32.30 RF00026.cm fasta
cmsearch -Z 5400 -T 26.35 RF00027.cm fasta
cmsearch -Z 5400 -T 34.00 -g RF00028.cm fasta
cmsearch -Z 5400 -T 21.25 -g –fil-no-hmm RF00029.cm fasta
cmsearch -Z 5400 -T 16.15 RF00030.cm fasta
cmsearch -Z 5400 -T 17.00 RF00031.cm fasta
cmsearch -Z 5400 -T 17.00 RF00032.cm fasta
cmsearch -Z 5400 -T 17.00 RF00033.cm fasta
cmsearch -Z 5400 -T 23.80 RF00034.cm fasta
cmsearch -Z 5400 -T 46.75 RF00035.cm fasta
cmsearch -Z 5400 -T 37.40 RF00036.cm fasta
cmsearch -Z 5400 -T 21.25 RF00037.cm fasta
cmsearch -Z 5400 -T 74.80 RF00038.cm fasta
cmsearch -Z 5400 -T 20.40 RF00039.cm fasta
cmsearch -Z 5400 -T 19.55 RF00040.cm fasta
cmsearch -Z 5400 -T 34.00 RF00041.cm fasta
cmsearch -Z 5400 -T 42.50 RF00042.cm fasta
cmsearch -Z 5400 -T 17.00 RF00043.cm fasta
cmsearch -Z 5400 -T 26.35 RF00044.cm fasta
cmsearch -Z 5400 -T 17.85 RF00045.cm fasta
cmsearch -Z 5400 -T 17.00 RF00046.cm fasta
cmsearch -Z 5400 -T 25.50 RF00047.cm fasta
cmsearch -Z 5400 -T 25.50 RF00048.cm fasta

Table S17 – continued from previous page

command line
cmsearch -Z 5400 -T 22.10 RF00049.cm fasta
cmsearch -Z 5400 -T 34.00 RF00050.cm fasta
cmsearch -Z 5400 -T 36.55 RF00051.cm fasta
cmsearch -Z 5400 -T 28.05 RF00052.cm fasta
cmsearch -Z 5400 -T 29.75 RF00053.cm fasta
cmsearch -Z 5400 -T 17.85 RF00054.cm fasta
cmsearch -Z 5400 -T 22.10 RF00055.cm fasta
cmsearch -Z 5400 -T 27.20 RF00056.cm fasta
cmsearch -Z 5400 -T 29.75 RF00057.cm fasta
cmsearch -Z 5400 -T 59.50 RF00058.cm fasta
cmsearch -Z 5400 -T 25.50 RF00059.cm fasta
cmsearch -Z 5400 -T 42.50 RF00060.cm fasta
cmsearch -Z 5400 -T 43.35 RF00061.cm fasta
cmsearch -Z 5400 -T 17.34 RF00062.cm fasta
cmsearch -Z 5400 -T 51.00 RF00063.cm fasta
cmsearch -Z 5400 -T 44.20 RF00064.cm fasta
cmsearch -Z 5400 -T 42.50 RF00065.cm fasta
cmsearch -Z 5400 -T 17.00 RF00066.cm fasta
cmsearch -Z 5400 -T 22.10 RF00067.cm fasta
cmsearch -Z 5400 -T 17.85 RF00068.cm fasta
cmsearch -Z 5400 -T 26.35 RF00069.cm fasta
cmsearch -Z 5400 -T 20.40 RF00070.cm fasta
cmsearch -Z 5400 -T 25.50 RF00071.cm fasta
cmsearch -Z 5400 -T 28.05 RF00072.cm fasta
cmsearch -Z 5400 -T 22.95 RF00073.cm fasta
cmsearch -Z 5400 -T 30.60 RF00074.cm fasta
cmsearch -Z 5400 -T 21.25 –fil-no-hmm RF00075.cm fasta
cmsearch -Z 5400 -T 26.35 RF00076.cm fasta
cmsearch -Z 5400 -T 27.20 RF00077.cm fasta
cmsearch -Z 5400 -T 25.50 RF00078.cm fasta
cmsearch -Z 5400 -T 32.30 RF00079.cm fasta
cmsearch -Z 5400 -T 27.20 RF00080.cm fasta
cmsearch -Z 5400 -T 23.80 RF00081.cm fasta
cmsearch -Z 5400 -T 25.50 -g RF00082.cm fasta
cmsearch -Z 5400 -T 33.15 RF00083.cm fasta
cmsearch -Z 5400 -T 29.75 RF00084.cm fasta
cmsearch -Z 5400 -T 27.20 RF00085.cm fasta
cmsearch -Z 5400 -T 18.70 RF00086.cm fasta
cmsearch -Z 5400 -T 18.70 RF00087.cm fasta
cmsearch -Z 5400 -T 20.40 RF00088.cm fasta
cmsearch -Z 5400 -T 22.95 RF00089.cm fasta
cmsearch -Z 5400 -T 16.15 RF00090.cm fasta
cmsearch -Z 5400 -T 18.70 RF00091.cm fasta
cmsearch -Z 5400 -T 18.70 RF00092.cm fasta
cmsearch -Z 5400 -T 20.40 RF00093.cm fasta
cmsearch -Z 5400 -T 19.55 RF00094.cm fasta
cmsearch -Z 5400 -T 17.00 RF00095.cm fasta
cmsearch -Z 5400 -T 29.75 RF00096.cm fasta
cmsearch -Z 5400 -T 21.25 RF00097.cm fasta

Table S17 – continued from previous page

command line
cmsearch -Z 5400 -T 22.95 RF00099.cm fasta
cmsearch -Z 5400 -T 23.80 RF00100.cm fasta
cmsearch -Z 5400 -T 26.35 RF00101.cm fasta
cmsearch -Z 5400 -T 25.50 RF00102.cm fasta
cmsearch -Z 5400 -T 25.50 RF00103.cm fasta
cmsearch -Z 5400 -T 21.25 RF00104.cm fasta
cmsearch -Z 5400 -T 22.10 RF00105.cm fasta
cmsearch -Z 5400 -T 26.35 RF00106.cm fasta
cmsearch -Z 5400 -T 17.00 RF00107.cm fasta
cmsearch -Z 5400 -T 20.40 RF00108.cm fasta
cmsearch -Z 5400 -T 26.35 RF00109.cm fasta
cmsearch -Z 5400 -T 34.00 RF00110.cm fasta
cmsearch -Z 5400 -T 28.90 RF00111.cm fasta
cmsearch -Z 5400 -T 25.50 RF00112.cm fasta
cmsearch -Z 5400 -T 46.75 RF00113.cm fasta
cmsearch -Z 5400 -T 17.85 RF00114.cm fasta
cmsearch -Z 5400 -T 25.50 RF00115.cm fasta
cmsearch -Z 5400 -T 59.50 RF00116.cm fasta
cmsearch -Z 5400 -T 42.50 RF00117.cm fasta
cmsearch -Z 5400 -T 40.80 RF00118.cm fasta
cmsearch -Z 5400 -T 34.00 RF00119.cm fasta
cmsearch -Z 5400 -T 29.75 RF00121.cm fasta
cmsearch -Z 5400 -T 38.25 RF00122.cm fasta
cmsearch -Z 5400 -T 24.65 RF00124.cm fasta
cmsearch -Z 5400 -T 68.00 RF00125.cm fasta
cmsearch -Z 5400 -T 51.00 RF00126.cm fasta
cmsearch -Z 5400 -T 27.20 RF00127.cm fasta
cmsearch -Z 5400 -T 28.05 RF00128.cm fasta
cmsearch -Z 5400 -T 43.35 RF00129.cm fasta
cmsearch -Z 5400 -T 18.70 RF00130.cm fasta
cmsearch -Z 5400 -T 37.40 RF00131.cm fasta
cmsearch -Z 5400 -T 19.55 RF00132.cm fasta
cmsearch -Z 5400 -T 18.70 RF00133.cm fasta
cmsearch -Z 5400 -T 17.00 RF00134.cm fasta
cmsearch -Z 5400 -T 14.101500 RF00135.cm fasta
cmsearch -Z 5400 -T 22.10 RF00136.cm fasta
cmsearch -Z 5400 -T 26.35 RF00137.cm fasta
cmsearch -Z 5400 -T 17.00 RF00138.cm fasta
cmsearch -Z 5400 -T 26.35 RF00139.cm fasta
cmsearch -Z 5400 -T 17.85 RF00140.cm fasta
cmsearch -Z 5400 -T 17.00 RF00142.cm fasta
cmsearch -Z 5400 -T 17.00 RF00143.cm fasta
cmsearch -Z 5400 -T 39.95 RF00144.cm fasta
cmsearch -Z 5400 -T 27.20 RF00145.cm fasta
cmsearch -Z 5400 -T 17.00 RF00147.cm fasta
cmsearch -Z 5400 -T 33.15 RF00149.cm fasta
cmsearch -Z 5400 -T 23.80 RF00150.cm fasta
cmsearch -Z 5400 -T 27.20 RF00151.cm fasta
cmsearch -Z 5400 -T 21.25 RF00152.cm fasta

Table S17 – continued from previous page

command line
cmsearch -Z 5400 -T 28.05 RF00153.cm fasta
cmsearch -Z 5400 -T 16.49 RF00154.cm fasta
cmsearch -Z 5400 -T 22.95 RF00155.cm fasta
cmsearch -Z 5400 -T 34.00 RF00156.cm fasta
cmsearch -Z 5400 -T 25.50 RF00157.cm fasta
cmsearch -Z 5400 -T 22.10 RF00158.cm fasta
cmsearch -Z 5400 -T 17.00 RF00159.cm fasta
cmsearch -Z 5400 -T 17.00 RF00160.cm fasta
cmsearch -Z 5400 -T 17.00 RF00161.cm fasta
cmsearch -Z 5400 -T 37.40 RF00162.cm fasta
cmsearch -Z 5400 -T 22.10 –fil-no-hmm RF00163.cm fasta
cmsearch -Z 5400 -T 26.35 RF00164.cm fasta
cmsearch -Z 5400 -T 28.90 RF00165.cm fasta
cmsearch -Z 5400 -T 24.65 RF00166.cm fasta
cmsearch -Z 5400 -T 29.75 RF00167.cm fasta
cmsearch -Z 5400 -T 40.80 RF00168.cm fasta
cmsearch -Z 5400 -T 34.00 RF00169.cm fasta
cmsearch -Z 5400 -T 17.00 RF00170.cm fasta
cmsearch -Z 5400 -T 29.75 RF00171.cm fasta
cmsearch -Z 5400 -T 17.00 RF00172.cm fasta
cmsearch -Z 5400 -T 17.00 RF00173.cm fasta
cmsearch -Z 5400 -T 33.15 RF00174.cm fasta
cmsearch -Z 5400 -T 25.50 RF00175.cm fasta
cmsearch -Z 5400 -T 20.40 RF00176.cm fasta
cmsearch -Z 5400 -T 510.00 -g RF00177.cm fasta
cmsearch -Z 5400 -T 30.60 RF00178.cm fasta
cmsearch -Z 5400 -T 17.00 RF00179.cm fasta
cmsearch -Z 5400 -T 17.00 RF00180.cm fasta
cmsearch -Z 5400 -T 22.10 RF00181.cm fasta
cmsearch -Z 5400 -T 17.00 RF00182.cm fasta
cmsearch -Z 5400 -T 27.20 RF00183.cm fasta
cmsearch -Z 5400 -T 17.00 RF00184.cm fasta
cmsearch -Z 5400 -T 22.10 RF00185.cm fasta
cmsearch -Z 5400 -T 18.70 RF00186.cm fasta
cmsearch -Z 5400 -T 29.75 RF00187.cm fasta
cmsearch -Z 5400 -T 24.65 RF00188.cm fasta
cmsearch -Z 5400 -T 18.70 RF00189.cm fasta
cmsearch -Z 5400 -T 28.90 RF00190.cm fasta
cmsearch -Z 5400 -T 29.75 RF00191.cm fasta
cmsearch -Z 5400 -T 17.00 RF00192.cm fasta
cmsearch -Z 5400 -T 32.30 RF00193.cm fasta
cmsearch -Z 5400 -T 24.65 RF00194.cm fasta
cmsearch -Z 5400 -T 23.80 RF00195.cm fasta
cmsearch -Z 5400 -T 17.00 RF00196.cm fasta
cmsearch -Z 5400 -T 22.95 RF00198.cm fasta
cmsearch -Z 5400 -T 27.20 RF00199.cm fasta
cmsearch -Z 5400 -T 17.00 RF00200.cm fasta
cmsearch -Z 5400 -T 17.00 RF00201.cm fasta
cmsearch -Z 5400 -T 25.50 RF00202.cm fasta

Table S17 – continued from previous page

command line
cmsearch -Z 5400 -T 17.00 RF00203.cm fasta
cmsearch -Z 5400 -T 18.70 RF00204.cm fasta
cmsearch -Z 5400 -T 23.80 RF00205.cm fasta
cmsearch -Z 5400 -T 17.00 RF00206.cm fasta
cmsearch -Z 5400 -T 17.00 RF00207.cm fasta
cmsearch -Z 5400 -T 17.00 RF00208.cm fasta
cmsearch -Z 5400 -T 51.85 RF00209.cm fasta
cmsearch -Z 5400 -T 18.70 RF00210.cm fasta
cmsearch -Z 5400 -T 28.05 RF00211.cm fasta
cmsearch -Z 5400 -T 28.90 RF00212.cm fasta
cmsearch -Z 5400 -T 23.80 RF00213.cm fasta
cmsearch -Z 5400 -T 18.70 RF00214.cm fasta
cmsearch -Z 5400 -T 20.06 RF00215.cm fasta
cmsearch -Z 5400 -T 17.85 RF00216.cm fasta
cmsearch -Z 5400 -T 22.95 RF00217.cm fasta
cmsearch -Z 5400 -T 20.40 RF00218.cm fasta
cmsearch -Z 5400 -T 34.00 RF00220.cm fasta
cmsearch -Z 5400 -T 17.00 –fil-no-hmm RF00221.cm fasta
cmsearch -Z 5400 -T 46.75 -g RF00222.cm fasta
cmsearch -Z 5400 -T 30.60 RF00223.cm fasta
cmsearch -Z 5400 -T 25.50 -g RF00224.cm fasta
cmsearch -Z 5400 -T 17.85 RF00225.cm fasta
cmsearch -Z 5400 -T 26.35 RF00226.cm fasta
cmsearch -Z 5400 -T 17.00 RF00227.cm fasta
cmsearch -Z 5400 -T 92.65 RF00228.cm fasta
cmsearch -Z 5400 -T 40.80 RF00229.cm fasta
cmsearch -Z 5400 -T 17.425000 RF00230.cm fasta
cmsearch -Z 5400 -T 41.65 RF00231.cm fasta
cmsearch -Z 5400 -T 22.95 RF00232.cm fasta
cmsearch -Z 5400 -T 19.55 RF00233.cm fasta
cmsearch -Z 5400 -T 19.55 RF00234.cm fasta
cmsearch -Z 5400 -T 21.25 RF00235.cm fasta
cmsearch -Z 5400 -T 17.00 RF00236.cm fasta
cmsearch -Z 5400 -T 18.70 RF00237.cm fasta
cmsearch -Z 5400 -T 17.00 RF00238.cm fasta
cmsearch -Z 5400 -T 20.40 RF00239.cm fasta
cmsearch -Z 5400 -T 29.75 RF00240.cm fasta
cmsearch -Z 5400 -T 27.20 RF00241.cm fasta
cmsearch -Z 5400 -T 17.195500 RF00242.cm fasta
cmsearch -Z 5400 -T 25.50 RF00243.cm fasta
cmsearch -Z 5400 -T 23.80 RF00244.cm fasta
cmsearch -Z 5400 -T 24.65 RF00245.cm fasta
cmsearch -Z 5400 -T 34.85 RF00246.cm fasta
cmsearch -Z 5400 -T 17.00 RF00247.cm fasta
cmsearch -Z 5400 -T 26.35 RF00248.cm fasta
cmsearch -Z 5400 -T 34.00 RF00249.cm fasta
cmsearch -Z 5400 -T 19.55 RF00250.cm fasta
cmsearch -Z 5400 -T 26.35 RF00251.cm fasta
cmsearch -Z 5400 -T 23.80 RF00252.cm fasta

Table S17 – continued from previous page

command line
cmsearch -Z 5400 -T 42.50 RF00253.cm fasta
cmsearch -Z 5400 -T 29.75 RF00254.cm fasta
cmsearch -Z 5400 -T 25.50 RF00255.cm fasta
cmsearch -Z 5400 -T 27.20 RF00256.cm fasta
cmsearch -Z 5400 -T 28.90 RF00257.cm fasta
cmsearch -Z 5400 -T 25.50 RF00258.cm fasta
cmsearch -Z 5400 -T 18.70 RF00259.cm fasta
cmsearch -Z 5400 -T 32.30 RF00260.cm fasta
cmsearch -Z 5400 -T 23.80 RF00261.cm fasta
cmsearch -Z 5400 -T 21.25 RF00262.cm fasta
cmsearch -Z 5400 -T 17.00 RF00263.cm fasta
cmsearch -Z 5400 -T 18.70 RF00264.cm fasta
cmsearch -Z 5400 -T 21.25 RF00265.cm fasta
cmsearch -Z 5400 -T 19.55 RF00266.cm fasta
cmsearch -Z 5400 -T 25.50 RF00267.cm fasta
cmsearch -Z 5400 -T 17.00 RF00268.cm fasta
cmsearch -Z 5400 -T 20.40 RF00270.cm fasta
cmsearch -Z 5400 -T 28.05 RF00271.cm fasta
cmsearch -Z 5400 -T 21.25 RF00272.cm fasta
cmsearch -Z 5400 -T 17.85 RF00273.cm fasta
cmsearch -Z 5400 -T 20.40 RF00274.cm fasta
cmsearch -Z 5400 -T 28.05 RF00275.cm fasta
cmsearch -Z 5400 -T 21.25 RF00276.cm fasta
cmsearch -Z 5400 -T 26.35 RF00277.cm fasta
cmsearch -Z 5400 -T 23.80 RF00278.cm fasta
cmsearch -Z 5400 -T 22.95 RF00279.cm fasta
cmsearch -Z 5400 -T 26.35 RF00280.cm fasta
cmsearch -Z 5400 -T 27.20 RF00281.cm fasta
cmsearch -Z 5400 -T 30.60 RF00282.cm fasta
cmsearch -Z 5400 -T 18.70 RF00283.cm fasta
cmsearch -Z 5400 -T 23.80 RF00284.cm fasta
cmsearch -Z 5400 -T 17.00 RF00285.cm fasta
cmsearch -Z 5400 -T 19.55 RF00286.cm fasta
cmsearch -Z 5400 -T 21.25 RF00287.cm fasta
cmsearch -Z 5400 -T 26.35 RF00288.cm fasta
cmsearch -Z 5400 -T 34.00 RF00289.cm fasta
cmsearch -Z 5400 -T 17.00 RF00290.cm fasta
cmsearch -Z 5400 -T 19.55 RF00291.cm fasta
cmsearch -Z 5400 -T 25.50 RF00292.cm fasta
cmsearch -Z 5400 -T 17.00 RF00293.cm fasta
cmsearch -Z 5400 -T 34.85 RF00294.cm fasta
cmsearch -Z 5400 -T 25.50 RF00295.cm fasta
cmsearch -Z 5400 -T 27.20 RF00296.cm fasta
cmsearch -Z 5400 -T 25.50 RF00300.cm fasta
cmsearch -Z 5400 -T 17.00 RF00301.cm fasta
cmsearch -Z 5400 -T 19.55 RF00302.cm fasta
cmsearch -Z 5400 -T 28.90 RF00303.cm fasta
cmsearch -Z 5400 -T 27.20 RF00304.cm fasta
cmsearch -Z 5400 -T 17.00 RF00305.cm fasta

Table S17 – continued from previous page

command line
cmsearch -Z 5400 -T 17.85 RF00306.cm fasta
cmsearch -Z 5400 -T 17.00 RF00307.cm fasta
cmsearch -Z 5400 -T 17.00 RF00309.cm fasta
cmsearch -Z 5400 -T 17.00 RF00310.cm fasta
cmsearch -Z 5400 -T 17.00 RF00311.cm fasta
cmsearch -Z 5400 -T 19.55 RF00312.cm fasta
cmsearch -Z 5400 -T 17.00 RF00313.cm fasta
cmsearch -Z 5400 -T 17.00 RF00314.cm fasta
cmsearch -Z 5400 -T 17.00 RF00315.cm fasta
cmsearch -Z 5400 -T 17.00 RF00316.cm fasta
cmsearch -Z 5400 -T 17.136000 RF00317.cm fasta
cmsearch -Z 5400 -T 17.00 RF00318.cm fasta
cmsearch -Z 5400 -T 24.65 RF00319.cm fasta
cmsearch -Z 5400 -T 17.00 RF00320.cm fasta
cmsearch -Z 5400 -T 15.589000 RF00321.cm fasta
cmsearch -Z 5400 -T 18.70 RF00322.cm fasta
cmsearch -Z 5400 -T 17.00 RF00323.cm fasta
cmsearch -Z 5400 -T 20.40 RF00324.cm fasta
cmsearch -Z 5400 -T 22.10 RF00325.cm fasta
cmsearch -Z 5400 -T 17.00 RF00326.cm fasta
cmsearch -Z 5400 -T 17.00 RF00327.cm fasta
cmsearch -Z 5400 -T 21.25 RF00328.cm fasta
cmsearch -Z 5400 -T 17.00 RF00329.cm fasta
cmsearch -Z 5400 -T 30.60 RF00330.cm fasta
cmsearch -Z 5400 -T 17.875500 RF00331.cm fasta
cmsearch -Z 5400 -T 29.75 RF00332.cm fasta
cmsearch -Z 5400 -T 17.00 RF00333.cm fasta
cmsearch -Z 5400 -T 21.25 RF00334.cm fasta
cmsearch -Z 5400 -T 17.136000 RF00335.cm fasta
cmsearch -Z 5400 -T 17.00 RF00336.cm fasta
cmsearch -Z 5400 -T 17.85 RF00337.cm fasta
cmsearch -Z 5400 -T 17.00 RF00338.cm fasta
cmsearch -Z 5400 -T 27.20 RF00339.cm fasta
cmsearch -Z 5400 -T 26.35 RF00340.cm fasta
cmsearch -Z 5400 -T 17.00 RF00341.cm fasta
cmsearch -Z 5400 -T 17.00 RF00342.cm fasta
cmsearch -Z 5400 -T 22.10 RF00343.cm fasta
cmsearch -Z 5400 -T 17.00 RF00344.cm fasta
cmsearch -Z 5400 -T 26.35 RF00345.cm fasta
cmsearch -Z 5400 -T 21.25 RF00348.cm fasta
cmsearch -Z 5400 -T 17.00 RF00349.cm fasta
cmsearch -Z 5400 -T 17.00 RF00350.cm fasta
cmsearch -Z 5400 -T 29.75 RF00351.cm fasta
cmsearch -Z 5400 -T 29.75 RF00352.cm fasta
cmsearch -Z 5400 -T 28.05 RF00353.cm fasta
cmsearch -Z 5400 -T 34.00 RF00355.cm fasta
cmsearch -Z 5400 -T 38.25 RF00356.cm fasta
cmsearch -Z 5400 -T 24.65 RF00357.cm fasta
cmsearch -Z 5400 -T 29.75 RF00358.cm fasta

Table S17 – continued from previous page

command line
cmsearch -Z 5400 -T 17.00 RF00359.cm fasta
cmsearch -Z 5400 -T 17.85 RF00360.cm fasta
cmsearch -Z 5400 -T 18.70 RF00361.cm fasta
cmsearch -Z 5400 -T 17.00 RF00362.cm fasta
cmsearch -Z 5400 -T 17.00 RF00363.cm fasta
cmsearch -Z 5400 -T 17.00 RF00364.cm fasta
cmsearch -Z 5400 -T 17.00 RF00365.cm fasta
cmsearch -Z 5400 -T 17.00 RF00366.cm fasta
cmsearch -Z 5400 -T 17.00 RF00367.cm fasta
cmsearch -Z 5400 -T 28.05 RF00368.cm fasta
cmsearch -Z 5400 -T 25.50 RF00369.cm fasta
cmsearch -Z 5400 -T 17.00 RF00370.cm fasta
cmsearch -Z 5400 -T 17.00 RF00371.cm fasta
cmsearch -Z 5400 -T 63.75 RF00372.cm fasta
cmsearch -Z 5400 -T 45.05 -g RF00373.cm fasta
cmsearch -Z 5400 -T 31.45 RF00374.cm fasta
cmsearch -Z 5400 -T 24.65 RF00375.cm fasta
cmsearch -Z 5400 -T 22.95 RF00376.cm fasta
cmsearch -Z 5400 -T 27.20 RF00377.cm fasta
cmsearch -Z 5400 -T 22.10 RF00378.cm fasta
cmsearch -Z 5400 -T 18.70 RF00379.cm fasta
cmsearch -Z 5400 -T 21.25 RF00380.cm fasta
cmsearch -Z 5400 -T 19.55 RF00381.cm fasta
cmsearch -Z 5400 -T 25.50 RF00382.cm fasta
cmsearch -Z 5400 -T 25.50 RF00383.cm fasta
cmsearch -Z 5400 -T 19.55 RF00384.cm fasta
cmsearch -Z 5400 -T 20.40 RF00385.cm fasta
cmsearch -Z 5400 -T 17.00 RF00386.cm fasta
cmsearch -Z 5400 -T 17.00 RF00387.cm fasta
cmsearch -Z 5400 -T 17.00 RF00388.cm fasta
cmsearch -Z 5400 -T 20.40 RF00389.cm fasta
cmsearch -Z 5400 -T 22.95 RF00390.cm fasta
cmsearch -Z 5400 -T 17.00 RF00391.cm fasta
cmsearch -Z 5400 -T 20.40 RF00392.cm fasta
cmsearch -Z 5400 -T 24.65 RF00393.cm fasta
cmsearch -Z 5400 -T 30.60 RF00394.cm fasta
cmsearch -Z 5400 -T 17.85 RF00396.cm fasta
cmsearch -Z 5400 -T 18.70 RF00397.cm fasta
cmsearch -Z 5400 -T 22.10 RF00398.cm fasta
cmsearch -Z 5400 -T 22.95 RF00399.cm fasta
cmsearch -Z 5400 -T 28.90 RF00400.cm fasta
cmsearch -Z 5400 -T 36.55 RF00401.cm fasta
cmsearch -Z 5400 -T 25.50 RF00402.cm fasta
cmsearch -Z 5400 -T 21.25 RF00403.cm fasta
cmsearch -Z 5400 -T 32.30 RF00404.cm fasta
cmsearch -Z 5400 -T 27.20 RF00405.cm fasta
cmsearch -Z 5400 -T 17.00 RF00406.cm fasta
cmsearch -Z 5400 -T 25.50 RF00407.cm fasta
cmsearch -Z 5400 -T 34.00 RF00408.cm fasta

Table S17 – continued from previous page

command line
cmsearch -Z 5400 -T 17.00 RF00409.cm fasta
cmsearch -Z 5400 -T 48.45 RF00410.cm fasta
cmsearch -Z 5400 -T 32.30 RF00411.cm fasta
cmsearch -Z 5400 -T 24.65 RF00412.cm fasta
cmsearch -Z 5400 -T 22.10 RF00413.cm fasta
cmsearch -Z 5400 -T 35.70 RF00414.cm fasta
cmsearch -Z 5400 -T 34.85 RF00415.cm fasta
cmsearch -Z 5400 -T 51.00 RF00416.cm fasta
cmsearch -Z 5400 -T 27.20 RF00417.cm fasta
cmsearch -Z 5400 -T 57.80 RF00418.cm fasta
cmsearch -Z 5400 -T 17.00 RF00419.cm fasta
cmsearch -Z 5400 -T 30.60 RF00420.cm fasta
cmsearch -Z 5400 -T 18.70 RF00421.cm fasta
cmsearch -Z 5400 -T 30.60 RF00422.cm fasta
cmsearch -Z 5400 -T 29.75 RF00423.cm fasta
cmsearch -Z 5400 -T 25.50 RF00424.cm fasta
cmsearch -Z 5400 -T 28.05 RF00425.cm fasta
cmsearch -Z 5400 -T 19.55 RF00426.cm fasta
cmsearch -Z 5400 -T 28.90 RF00427.cm fasta
cmsearch -Z 5400 -T 29.75 RF00428.cm fasta
cmsearch -Z 5400 -T 34.00 RF00429.cm fasta
cmsearch -Z 5400 -T 25.50 RF00430.cm fasta
cmsearch -Z 5400 -T 17.85 RF00431.cm fasta
cmsearch -Z 5400 -T 28.05 RF00432.cm fasta
cmsearch -Z 5400 -T 18.70 RF00433.cm fasta
cmsearch -Z 5400 -T 17.85 RF00434.cm fasta
cmsearch -Z 5400 -T 18.70 RF00435.cm fasta
cmsearch -Z 5400 -T 21.25 RF00436.cm fasta
cmsearch -Z 5400 -T 17.00 RF00437.cm fasta
cmsearch -Z 5400 -T 31.45 RF00438.cm fasta
cmsearch -Z 5400 -T 28.90 RF00439.cm fasta
cmsearch -Z 5400 -T 17.280500 RF00440.cm fasta
cmsearch -Z 5400 -T 29.75 RF00441.cm fasta
cmsearch -Z 5400 -T 20.40 RF00442.cm fasta
cmsearch -Z 5400 -T 21.25 RF00443.cm fasta
cmsearch -Z 5400 -T 27.20 RF00444.cm fasta
cmsearch -Z 5400 -T 25.50 RF00445.cm fasta
cmsearch -Z 5400 -T 17.85 RF00446.cm fasta
cmsearch -Z 5400 -T 28.05 RF00447.cm fasta
cmsearch -Z 5400 -T 17.00 RF00448.cm fasta
cmsearch -Z 5400 -T 16.15 RF00449.cm fasta
cmsearch -Z 5400 -T 28.90 RF00451.cm fasta
cmsearch -Z 5400 -T 25.50 –fil-no-hmm RF00452.cm fasta
cmsearch -Z 5400 -T 29.75 RF00453.cm fasta
cmsearch -Z 5400 -T 29.75 RF00454.cm fasta
cmsearch -Z 5400 -T 27.20 RF00455.cm fasta
cmsearch -Z 5400 -T 39.95 RF00456.cm fasta
cmsearch -Z 5400 -T 25.50 RF00457.cm fasta
cmsearch -Z 5400 -T 24.65 RF00458.cm fasta

Table S17 – continued from previous page

command line
cmsearch -Z 5400 -T 45.90 RF00459.cm fasta
cmsearch -Z 5400 -T 28.90 RF00460.cm fasta
cmsearch -Z 5400 -T 48.45 RF00461.cm fasta
cmsearch -Z 5400 -T 17.00 RF00462.cm fasta
cmsearch -Z 5400 -T 25.50 RF00463.cm fasta
cmsearch -Z 5400 -T 18.70 RF00464.cm fasta
cmsearch -Z 5400 -T 22.95 RF00465.cm fasta
cmsearch -Z 5400 -T 22.95 RF00467.cm fasta
cmsearch -Z 5400 -T 20.40 RF00468.cm fasta
cmsearch -Z 5400 -T 29.75 RF00469.cm fasta
cmsearch -Z 5400 -T 64.60 RF00470.cm fasta
cmsearch -Z 5400 -T 38.25 RF00471.cm fasta
cmsearch -Z 5400 -T 32.30 RF00472.cm fasta
cmsearch -Z 5400 -T 25.50 RF00473.cm fasta
cmsearch -Z 5400 -T 21.25 RF00474.cm fasta
cmsearch -Z 5400 -T 28.05 RF00475.cm fasta
cmsearch -Z 5400 -T 24.65 RF00476.cm fasta
cmsearch -Z 5400 -T 26.35 -g RF00477.cm fasta
cmsearch -Z 5400 -T 23.80 RF00478.cm fasta
cmsearch -Z 5400 -T 28.90 RF00479.cm fasta
cmsearch -Z 5400 -T 30.60 RF00480.cm fasta
cmsearch -Z 5400 -T 62.05 RF00481.cm fasta
cmsearch -Z 5400 -T 17.00 RF00482.cm fasta
cmsearch -Z 5400 -T 17.00 RF00483.cm fasta
cmsearch -Z 5400 -T 18.70 RF00484.cm fasta
cmsearch -Z 5400 -T 17.00 RF00485.cm fasta
cmsearch -Z 5400 -T 18.70 RF00486.cm fasta
cmsearch -Z 5400 -T 31.45 RF00487.cm fasta
cmsearch -Z 5400 -T 85.00 RF00488.cm fasta
cmsearch -Z 5400 -T 29.75 RF00489.cm fasta
cmsearch -Z 5400 -T 17.00 RF00490.cm fasta
cmsearch -Z 5400 -T 17.85 RF00491.cm fasta
cmsearch -Z 5400 -T 31.45 RF00492.cm fasta
cmsearch -Z 5400 -T 26.35 RF00493.cm fasta
cmsearch -Z 5400 -T 18.70 RF00494.cm fasta
cmsearch -Z 5400 -T 28.90 RF00495.cm fasta
cmsearch -Z 5400 -T 17.00 RF00496.cm fasta
cmsearch -Z 5400 -T 17.00 RF00498.cm fasta
cmsearch -Z 5400 -T 17.85 RF00499.cm fasta
cmsearch -Z 5400 -T 17.00 RF00500.cm fasta
cmsearch -Z 5400 -T 17.00 RF00501.cm fasta
cmsearch -Z 5400 -T 34.00 RF00502.cm fasta
cmsearch -Z 5400 -T 79.90 RF00503.cm fasta
cmsearch -Z 5400 -T 18.70 RF00504.cm fasta
cmsearch -Z 5400 -T 38.25 RF00505.cm fasta
cmsearch -Z 5400 -T 28.90 RF00506.cm fasta
cmsearch -Z 5400 -T 24.65 RF00507.cm fasta
cmsearch -Z 5400 -T 32.30 RF00509.cm fasta
cmsearch -Z 5400 -T 38.25 RF00510.cm fasta

Table S17 – continued from previous page

command line
cmsearch -Z 5400 -T 38.25 RF00511.cm fasta
cmsearch -Z 5400 -T 28.05 RF00512.cm fasta
cmsearch -Z 5400 -T 28.90 RF00513.cm fasta
cmsearch -Z 5400 -T 32.30 RF00514.cm fasta
cmsearch -Z 5400 -T 18.70 –fil-no-hmm RF00515.cm fasta
cmsearch -Z 5400 -T 23.80 RF00516.cm fasta
cmsearch -Z 5400 -T 26.35 RF00517.cm fasta
cmsearch -Z 5400 -T 22.95 -g –fil-no-hmm RF00518.cm fasta
cmsearch -Z 5400 -T 17.85 RF00519.cm fasta
cmsearch -Z 5400 -T 18.70 RF00520.cm fasta
cmsearch -Z 5400 -T 22.10 RF00521.cm fasta
cmsearch -Z 5400 -T 26.35 RF00522.cm fasta
cmsearch -Z 5400 -T 21.25 RF00523.cm fasta
cmsearch -Z 5400 -T 32.30 RF00524.cm fasta
cmsearch -Z 5400 -T 20.40 RF00525.cm fasta
cmsearch -Z 5400 -T 17.00 RF00526.cm fasta
cmsearch -Z 5400 -T 17.00 RF00527.cm fasta
cmsearch -Z 5400 -T 40.80 RF00528.cm fasta
cmsearch -Z 5400 -T 17.85 RF00529.cm fasta
cmsearch -Z 5400 -T 24.65 RF00530.cm fasta
cmsearch -Z 5400 -T 17.00 RF00531.cm fasta
cmsearch -Z 5400 -T 17.00 RF00532.cm fasta
cmsearch -Z 5400 -T 17.00 RF00533.cm fasta
cmsearch -Z 5400 -T 24.65 RF00534.cm fasta
cmsearch -Z 5400 -T 17.00 RF00535.cm fasta
cmsearch -Z 5400 -T 17.00 RF00536.cm fasta
cmsearch -Z 5400 -T 16.787500 RF00537.cm fasta
cmsearch -Z 5400 -T 17.00 RF00538.cm fasta
cmsearch -Z 5400 -T 28.90 RF00539.cm fasta
cmsearch -Z 5400 -T 21.25 RF00540.cm fasta
cmsearch -Z 5400 -T 21.25 RF00541.cm fasta
cmsearch -Z 5400 -T 23.80 RF00542.cm fasta
cmsearch -Z 5400 -T 32.30 RF00543.cm fasta
cmsearch -Z 5400 -T 17.85 RF00544.cm fasta
cmsearch -Z 5400 -T 18.70 RF00545.cm fasta
cmsearch -Z 5400 -T 22.10 RF00546.cm fasta
cmsearch -Z 5400 -T 34.00 RF00547.cm fasta
cmsearch -Z 5400 -T 33.15 –fil-no-hmm RF00548.cm fasta
cmsearch -Z 5400 -T 96.90 RF00549.cm fasta
cmsearch -Z 5400 -T 45.05 RF00550.cm fasta
cmsearch -Z 5400 -T 77.35 RF00551.cm fasta
cmsearch -Z 5400 -T 36.55 RF00552.cm fasta
cmsearch -Z 5400 -T 21.25 RF00553.cm fasta
cmsearch -Z 5400 -T 45.90 RF00554.cm fasta
cmsearch -Z 5400 -T 25.50 –fil-no-hmm RF00555.cm fasta
cmsearch -Z 5400 -T 18.70 RF00556.cm fasta
cmsearch -Z 5400 -T 25.50 RF00557.cm fasta
cmsearch -Z 5400 -T 25.50 RF00558.cm fasta
cmsearch -Z 5400 -T 17.00 RF00559.cm fasta

Table S17 – continued from previous page

command line
cmsearch -Z 5400 -T 23.80 RF00560.cm fasta
cmsearch -Z 5400 -T 43.35 RF00561.cm fasta
cmsearch -Z 5400 -T 20.40 RF00562.cm fasta
cmsearch -Z 5400 -T 32.30 RF00563.cm fasta
cmsearch -Z 5400 -T 17.85 RF00564.cm fasta
cmsearch -Z 5400 -T 20.40 RF00565.cm fasta
cmsearch -Z 5400 -T 17.85 RF00566.cm fasta
cmsearch -Z 5400 -T 38.25 RF00567.cm fasta
cmsearch -Z 5400 -T 31.45 RF00568.cm fasta
cmsearch -Z 5400 -T 19.55 RF00569.cm fasta
cmsearch -Z 5400 -T 19.295000 RF00570.cm fasta
cmsearch -Z 5400 -T 26.35 RF00571.cm fasta
cmsearch -Z 5400 -T 23.80 RF00572.cm fasta
cmsearch -Z 5400 -T 24.65 RF00573.cm fasta
cmsearch -Z 5400 -T 24.65 RF00574.cm fasta
cmsearch -Z 5400 -T 16.15 RF00575.cm fasta
cmsearch -Z 5400 -T 28.90 RF00576.cm fasta
cmsearch -Z 5400 -T 32.30 RF00577.cm fasta
cmsearch -Z 5400 -T 18.70 RF00578.cm fasta
cmsearch -Z 5400 -T 29.75 RF00579.cm fasta
cmsearch -Z 5400 -T 33.15 RF00580.cm fasta
cmsearch -Z 5400 -T 23.80 RF00581.cm fasta
cmsearch -Z 5400 -T 21.25 RF00582.cm fasta
cmsearch -Z 5400 -T 38.25 RF00584.cm fasta
cmsearch -Z 5400 -T 27.20 RF00586.cm fasta
cmsearch -Z 5400 -T 21.25 RF00588.cm fasta
cmsearch -Z 5400 -T 17.85 RF00591.cm fasta
cmsearch -Z 5400 -T 21.25 RF00592.cm fasta
cmsearch -Z 5400 -T 18.275000 RF00593.cm fasta
cmsearch -Z 5400 -T 17.00 RF00594.cm fasta
cmsearch -Z 5400 -T 19.55 RF00598.cm fasta
cmsearch -Z 5400 -T 24.65 RF00599.cm fasta
cmsearch -Z 5400 -T 28.05 RF00600.cm fasta
cmsearch -Z 5400 -T 22.10 RF00601.cm fasta
cmsearch -Z 5400 -T 27.20 RF00602.cm fasta
cmsearch -Z 5400 -T 27.20 RF00603.cm fasta
cmsearch -Z 5400 -T 22.95 RF00604.cm fasta
cmsearch -Z 5400 -T 25.50 RF00606.cm fasta
cmsearch -Z 5400 -T 17.00 RF00607.cm fasta
cmsearch -Z 5400 -T 29.75 RF00608.cm fasta
cmsearch -Z 5400 -T 22.10 RF00609.cm fasta
cmsearch -Z 5400 -T 24.65 RF00610.cm fasta
cmsearch -Z 5400 -T 22.10 RF00611.cm fasta
cmsearch -Z 5400 -T 18.70 RF00612.cm fasta
cmsearch -Z 5400 -T 21.25 RF00613.cm fasta
cmsearch -Z 5400 -T 21.25 RF00614.cm fasta
cmsearch -Z 5400 -T 85.00 RF00615.cm fasta
cmsearch -Z 5400 -T 30.60 RF00616.cm fasta
cmsearch -Z 5400 -T 26.35 RF00617.cm fasta

Table S17 – continued from previous page

command line
cmsearch -Z 5400 -T 24.65 RF00618.cm fasta
cmsearch -Z 5400 -T 28.05 RF00619.cm fasta
cmsearch -Z 5400 -T 37.40 RF00620.cm fasta
cmsearch -Z 5400 -T 27.20 RF00621.cm fasta
cmsearch -Z 5400 -T 17.00 RF00622.cm fasta
cmsearch -Z 5400 -T 17.00 RF00623.cm fasta
cmsearch -Z 5400 -T 29.75 RF00624.cm fasta
cmsearch -Z 5400 -T 22.10 RF00625.cm fasta
cmsearch -Z 5400 -T 17.00 RF00626.cm fasta
cmsearch -Z 5400 -T 32.30 RF00627.cm fasta
cmsearch -Z 5400 -T 34.00 -g RF00628.cm fasta
cmsearch -Z 5400 -T 39.95 RF00629.cm fasta
cmsearch -Z 5400 -T 30.60 RF00630.cm fasta
cmsearch -Z 5400 -T 17.00 RF00632.cm fasta
cmsearch -Z 5400 -T 25.50 RF00634.cm fasta
cmsearch -Z 5400 -T 21.25 RF00635.cm fasta
cmsearch -Z 5400 -T 93.50 RF00636.cm fasta
cmsearch -Z 5400 -T 17.00 RF00637.cm fasta
cmsearch -Z 5400 -T 34.00 RF00638.cm fasta
cmsearch -Z 5400 -T 43.35 RF00639.cm fasta
cmsearch -Z 5400 -T 25.50 –fil-no-hmm RF00640.cm fasta
cmsearch -Z 5400 -T 18.70 RF00641.cm fasta
cmsearch -Z 5400 -T 22.95 RF00642.cm fasta
cmsearch -Z 5400 -T 38.25 –fil-no-hmm RF00643.cm fasta
cmsearch -Z 5400 -T 37.40 RF00644.cm fasta
cmsearch -Z 5400 -T 27.20 –fil-no-hmm RF00645.cm fasta
cmsearch -Z 5400 -T 28.90 RF00646.cm fasta
cmsearch -Z 5400 -T 20.40 –fil-no-hmm RF00647.cm fasta
cmsearch -Z 5400 -T 34.00 –fil-no-hmm RF00648.cm fasta
cmsearch -Z 5400 -T 42.50 RF00649.cm fasta
cmsearch -Z 5400 -T 38.25 RF00650.cm fasta
cmsearch -Z 5400 -T 25.50 RF00651.cm fasta
cmsearch -Z 5400 -T 32.30 RF00652.cm fasta
cmsearch -Z 5400 -T 18.70 RF00653.cm fasta
cmsearch -Z 5400 -T 29.75 RF00654.cm fasta
cmsearch -Z 5400 -T 35.70 RF00655.cm fasta
cmsearch -Z 5400 -T 18.70 RF00656.cm fasta
cmsearch -Z 5400 -T 22.10 RF00657.cm fasta
cmsearch -Z 5400 -T 42.50 RF00658.cm fasta
cmsearch -Z 5400 -T 18.70 RF00659.cm fasta
cmsearch -Z 5400 -T 34.00 RF00660.cm fasta
cmsearch -Z 5400 -T 22.95 RF00661.cm fasta
cmsearch -Z 5400 -T 28.90 RF00662.cm fasta
cmsearch -Z 5400 -T 17.00 RF00663.cm fasta
cmsearch -Z 5400 -T 46.75 RF00664.cm fasta
cmsearch -Z 5400 -T 25.50 RF00665.cm fasta
cmsearch -Z 5400 -T 34.00 RF00666.cm fasta
cmsearch -Z 5400 -T 34.00 RF00667.cm fasta
cmsearch -Z 5400 -T 19.55 RF00668.cm fasta

Table S17 – continued from previous page

command line
cmsearch -Z 5400 -T 18.70 RF00669.cm fasta
cmsearch -Z 5400 -T 17.00 RF00670.cm fasta
cmsearch -Z 5400 -T 21.25 RF00671.cm fasta
cmsearch -Z 5400 -T 32.30 RF00672.cm fasta
cmsearch -Z 5400 -T 17.00 RF00673.cm fasta
cmsearch -Z 5400 -T 42.50 RF00674.cm fasta
cmsearch -Z 5400 -T 42.50 RF00675.cm fasta
cmsearch -Z 5400 -T 17.00 RF00676.cm fasta
cmsearch -Z 5400 -T 16.15 RF00677.cm fasta
cmsearch -Z 5400 -T 63.75 RF00678.cm fasta
cmsearch -Z 5400 -T 23.80 RF00679.cm fasta
cmsearch -Z 5400 -T 42.50 RF00680.cm fasta
cmsearch -Z 5400 -T 17.00 RF00681.cm fasta
cmsearch -Z 5400 -T 17.00 RF00682.cm fasta
cmsearch -Z 5400 -T 42.50 RF00683.cm fasta
cmsearch -Z 5400 -T 29.75 RF00684.cm fasta
cmsearch -Z 5400 -T 17.00 RF00685.cm fasta
cmsearch -Z 5400 -T 18.70 RF00686.cm fasta
cmsearch -Z 5400 -T 17.00 RF00687.cm fasta
cmsearch -Z 5400 -T 35.70 RF00688.cm fasta
cmsearch -Z 5400 -T 39.10 RF00689.cm fasta
cmsearch -Z 5400 -T 32.30 RF00690.cm fasta
cmsearch -Z 5400 -T 20.40 RF00691.cm fasta
cmsearch -Z 5400 -T 46.75 RF00692.cm fasta
cmsearch -Z 5400 -T 22.10 RF00693.cm fasta
cmsearch -Z 5400 -T 18.70 RF00694.cm fasta
cmsearch -Z 5400 -T 34.00 RF00695.cm fasta
cmsearch -Z 5400 -T 17.00 RF00696.cm fasta
cmsearch -Z 5400 -T 34.00 RF00697.cm fasta
cmsearch -Z 5400 -T 42.50 RF00698.cm fasta
cmsearch -Z 5400 -T 21.25 RF00699.cm fasta
cmsearch -Z 5400 -T 25.50 RF00700.cm fasta
cmsearch -Z 5400 -T 59.50 RF00701.cm fasta
cmsearch -Z 5400 -T 51.00 RF00702.cm fasta
cmsearch -Z 5400 -T 17.00 RF00703.cm fasta
cmsearch -Z 5400 -T 17.85 RF00704.cm fasta
cmsearch -Z 5400 -T 51.00 RF00705.cm fasta
cmsearch -Z 5400 -T 27.20 RF00706.cm fasta
cmsearch -Z 5400 -T 17.00 RF00707.cm fasta
cmsearch -Z 5400 -T 22.10 RF00708.cm fasta
cmsearch -Z 5400 -T 42.50 RF00709.cm fasta
cmsearch -Z 5400 -T 27.20 RF00710.cm fasta
cmsearch -Z 5400 -T 20.40 RF00711.cm fasta
cmsearch -Z 5400 -T 17.00 RF00712.cm fasta
cmsearch -Z 5400 -T 18.70 RF00713.cm fasta
cmsearch -Z 5400 -T 47.60 RF00714.cm fasta
cmsearch -Z 5400 -T 17.00 RF00715.cm fasta
cmsearch -Z 5400 -T 17.00 RF00716.cm fasta
cmsearch -Z 5400 -T 17.00 RF00717.cm fasta

Table S17 – continued from previous page

command line
cmsearch -Z 5400 -T 42.50 RF00718.cm fasta
cmsearch -Z 5400 -T 25.50 RF00719.cm fasta
cmsearch -Z 5400 -T 17.00 RF00720.cm fasta
cmsearch -Z 5400 -T 17.85 RF00721.cm fasta
cmsearch -Z 5400 -T 34.85 RF00722.cm fasta
cmsearch -Z 5400 -T 18.70 RF00723.cm fasta
cmsearch -Z 5400 -T 25.50 RF00724.cm fasta
cmsearch -Z 5400 -T 17.00 RF00725.cm fasta
cmsearch -Z 5400 -T 17.00 RF00726.cm fasta
cmsearch -Z 5400 -T 48.45 RF00727.cm fasta
cmsearch -Z 5400 -T 45.90 RF00728.cm fasta
cmsearch -Z 5400 -T 17.00 RF00729.cm fasta
cmsearch -Z 5400 -T 17.85 RF00730.cm fasta
cmsearch -Z 5400 -T 23.80 RF00731.cm fasta
cmsearch -Z 5400 -T 17.00 RF00732.cm fasta
cmsearch -Z 5400 -T 34.00 RF00733.cm fasta
cmsearch -Z 5400 -T 38.25 RF00734.cm fasta
cmsearch -Z 5400 -T 42.50 RF00735.cm fasta
cmsearch -Z 5400 -T 20.40 RF00736.cm fasta
cmsearch -Z 5400 -T 42.50 RF00737.cm fasta
cmsearch -Z 5400 -T 17.00 RF00739.cm fasta
cmsearch -Z 5400 -T 17.00 RF00740.cm fasta
cmsearch -Z 5400 -T 34.00 RF00741.cm fasta
cmsearch -Z 5400 -T 34.00 RF00742.cm fasta
cmsearch -Z 5400 -T 17.00 RF00743.cm fasta
cmsearch -Z 5400 -T 42.50 RF00744.cm fasta
cmsearch -Z 5400 -T 46.75 RF00745.cm fasta
cmsearch -Z 5400 -T 38.25 RF00746.cm fasta
cmsearch -Z 5400 -T 17.00 RF00747.cm fasta
cmsearch -Z 5400 -T 46.75 RF00748.cm fasta
cmsearch -Z 5400 -T 19.55 RF00749.cm fasta
cmsearch -Z 5400 -T 17.00 RF00750.cm fasta
cmsearch -Z 5400 -T 34.00 RF00751.cm fasta
cmsearch -Z 5400 -T 17.00 RF00752.cm fasta
cmsearch -Z 5400 -T 38.25 RF00753.cm fasta
cmsearch -Z 5400 -T 17.00 RF00754.cm fasta
cmsearch -Z 5400 -T 34.00 RF00755.cm fasta
cmsearch -Z 5400 -T 17.00 RF00756.cm fasta
cmsearch -Z 5400 -T 17.00 RF00757.cm fasta
cmsearch -Z 5400 -T 17.00 RF00758.cm fasta
cmsearch -Z 5400 -T 19.55 RF00760.cm fasta
cmsearch -Z 5400 -T 28.90 RF00761.cm fasta
cmsearch -Z 5400 -T 38.25 RF00762.cm fasta
cmsearch -Z 5400 -T 34.00 RF00763.cm fasta
cmsearch -Z 5400 -T 29.75 RF00764.cm fasta
cmsearch -Z 5400 -T 42.50 RF00765.cm fasta
cmsearch -Z 5400 -T 46.75 RF00766.cm fasta
cmsearch -Z 5400 -T 21.25 RF00767.cm fasta
cmsearch -Z 5400 -T 68.00 RF00768.cm fasta

Table S17 – continued from previous page

command line
cmsearch -Z 5400 -T 17.00 RF00769.cm fasta
cmsearch -Z 5400 -T 51.00 RF00770.cm fasta
cmsearch -Z 5400 -T 21.25 RF00771.cm fasta
cmsearch -Z 5400 -T 38.25 RF00772.cm fasta
cmsearch -Z 5400 -T 17.00 RF00773.cm fasta
cmsearch -Z 5400 -T 25.50 RF00774.cm fasta
cmsearch -Z 5400 -T 17.85 RF00775.cm fasta
cmsearch -Z 5400 -T 16.762000 RF00776.cm fasta
cmsearch -Z 5400 -T 38.25 RF00777.cm fasta
cmsearch -Z 5400 -T 22.10 RF00778.cm fasta
cmsearch -Z 5400 -T 18.70 RF00779.cm fasta
cmsearch -Z 5400 -T 36.55 RF00780.cm fasta
cmsearch -Z 5400 -T 17.00 RF00781.cm fasta
cmsearch -Z 5400 -T 22.10 RF00782.cm fasta
cmsearch -Z 5400 -T 23.80 RF00783.cm fasta
cmsearch -Z 5400 -T 42.50 RF00784.cm fasta
cmsearch -Z 5400 -T 42.50 RF00785.cm fasta
cmsearch -Z 5400 -T 17.00 RF00786.cm fasta
cmsearch -Z 5400 -T 17.00 RF00787.cm fasta
cmsearch -Z 5400 -T 17.00 RF00788.cm fasta
cmsearch -Z 5400 -T 17.00 RF00789.cm fasta
cmsearch -Z 5400 -T 42.50 RF00790.cm fasta
cmsearch -Z 5400 -T 42.50 RF00791.cm fasta
cmsearch -Z 5400 -T 46.75 RF00792.cm fasta
cmsearch -Z 5400 -T 59.50 RF00793.cm fasta
cmsearch -Z 5400 -T 42.50 RF00794.cm fasta
cmsearch -Z 5400 -T 25.50 RF00795.cm fasta
cmsearch -Z 5400 -T 25.50 RF00796.cm fasta
cmsearch -Z 5400 -T 42.50 RF00797.cm fasta
cmsearch -Z 5400 -T 25.50 RF00798.cm fasta
cmsearch -Z 5400 -T 34.00 RF00799.cm fasta
cmsearch -Z 5400 -T 42.50 RF00800.cm fasta
cmsearch -Z 5400 -T 17.00 RF00801.cm fasta
cmsearch -Z 5400 -T 17.00 RF00802.cm fasta
cmsearch -Z 5400 -T 26.35 RF00803.cm fasta
cmsearch -Z 5400 -T 42.50 RF00804.cm fasta
cmsearch -Z 5400 -T 68.00 RF00805.cm fasta
cmsearch -Z 5400 -T 42.50 RF00806.cm fasta
cmsearch -Z 5400 -T 17.00 RF00807.cm fasta
cmsearch -Z 5400 -T 22.10 RF00808.cm fasta
cmsearch -Z 5400 -T 34.00 RF00809.cm fasta
cmsearch -Z 5400 -T 42.50 RF00810.cm fasta
cmsearch -Z 5400 -T 25.50 RF00811.cm fasta
cmsearch -Z 5400 -T 42.50 RF00812.cm fasta
cmsearch -Z 5400 -T 17.00 RF00813.cm fasta
cmsearch -Z 5400 -T 17.00 RF00814.cm fasta
cmsearch -Z 5400 -T 42.50 RF00815.cm fasta
cmsearch -Z 5400 -T 17.00 RF00816.cm fasta
cmsearch -Z 5400 -T 42.50 RF00817.cm fasta

Table S17 – continued from previous page

command line
cmsearch -Z 5400 -T 17.00 RF00818.cm fasta
cmsearch -Z 5400 -T 42.50 RF00819.cm fasta
cmsearch -Z 5400 -T 42.50 RF00820.cm fasta
cmsearch -Z 5400 -T 34.00 RF00821.cm fasta
cmsearch -Z 5400 -T 17.00 RF00822.cm fasta
cmsearch -Z 5400 -T 29.75 RF00823.cm fasta
cmsearch -Z 5400 -T 25.50 RF00824.cm fasta
cmsearch -Z 5400 -T 28.90 RF00825.cm fasta
cmsearch -Z 5400 -T 29.75 RF00826.cm fasta
cmsearch -Z 5400 -T 38.25 RF00827.cm fasta
cmsearch -Z 5400 -T 34.00 RF00828.cm fasta
cmsearch -Z 5400 -T 17.00 RF00829.cm fasta
cmsearch -Z 5400 -T 34.00 RF00830.cm fasta
cmsearch -Z 5400 -T 29.75 RF00831.cm fasta
cmsearch -Z 5400 -T 22.10 RF00832.cm fasta
cmsearch -Z 5400 -T 59.50 RF00833.cm fasta
cmsearch -Z 5400 -T 17.00 RF00834.cm fasta
cmsearch -Z 5400 -T 34.00 RF00835.cm fasta
cmsearch -Z 5400 -T 38.25 RF00836.cm fasta
cmsearch -Z 5400 -T 42.50 RF00837.cm fasta
cmsearch -Z 5400 -T 32.30 RF00838.cm fasta
cmsearch -Z 5400 -T 17.00 RF00839.cm fasta
cmsearch -Z 5400 -T 34.00 RF00840.cm fasta
cmsearch -Z 5400 -T 22.10 RF00841.cm fasta
cmsearch -Z 5400 -T 42.50 RF00842.cm fasta
cmsearch -Z 5400 -T 25.50 RF00843.cm fasta
cmsearch -Z 5400 -T 34.00 RF00844.cm fasta
cmsearch -Z 5400 -T 17.00 RF00845.cm fasta
cmsearch -Z 5400 -T 29.75 RF00846.cm fasta
cmsearch -Z 5400 -T 42.50 RF00847.cm fasta
cmsearch -Z 5400 -T 25.50 RF00848.cm fasta
cmsearch -Z 5400 -T 17.00 RF00849.cm fasta
cmsearch -Z 5400 -T 17.00 RF00850.cm fasta
cmsearch -Z 5400 -T 25.50 RF00851.cm fasta
cmsearch -Z 5400 -T 21.25 RF00852.cm fasta
cmsearch -Z 5400 -T 17.00 RF00853.cm fasta
cmsearch -Z 5400 -T 17.00 RF00854.cm fasta
cmsearch -Z 5400 -T 34.00 RF00855.cm fasta
cmsearch -Z 5400 -T 42.50 RF00856.cm fasta
cmsearch -Z 5400 -T 25.50 RF00857.cm fasta
cmsearch -Z 5400 -T 17.00 RF00858.cm fasta
cmsearch -Z 5400 -T 42.50 RF00859.cm fasta
cmsearch -Z 5400 -T 17.00 RF00861.cm fasta
cmsearch -Z 5400 -T 17.00 RF00862.cm fasta
cmsearch -Z 5400 -T 18.70 RF00863.cm fasta
cmsearch -Z 5400 -T 17.00 RF00864.cm fasta
cmsearch -Z 5400 -T 20.40 RF00865.cm fasta
cmsearch -Z 5400 -T 17.00 RF00866.cm fasta
cmsearch -Z 5400 -T 17.00 RF00867.cm fasta

Table S17 – continued from previous page

command line
cmsearch -Z 5400 -T 17.00 RF00868.cm fasta
cmsearch -Z 5400 -T 17.00 RF00869.cm fasta
cmsearch -Z 5400 -T 34.00 RF00870.cm fasta
cmsearch -Z 5400 -T 17.00 RF00871.cm fasta
cmsearch -Z 5400 -T 42.50 RF00872.cm fasta
cmsearch -Z 5400 -T 17.00 RF00873.cm fasta
cmsearch -Z 5400 -T 17.85 RF00874.cm fasta
cmsearch -Z 5400 -T 17.85 RF00875.cm fasta
cmsearch -Z 5400 -T 13.60 RF00876.cm fasta
cmsearch -Z 5400 -T 17.00 RF00877.cm fasta
cmsearch -Z 5400 -T 17.00 RF00878.cm fasta
cmsearch -Z 5400 -T 29.75 RF00879.cm fasta
cmsearch -Z 5400 -T 73.95 RF00882.cm fasta
cmsearch -Z 5400 -T 21.25 RF00883.cm fasta
cmsearch -Z 5400 -T 17.00 RF00884.cm fasta
cmsearch -Z 5400 -T 82.45 RF00885.cm fasta
cmsearch -Z 5400 -T 41.65 RF00886.cm fasta
cmsearch -Z 5400 -T 34.00 RF00887.cm fasta
cmsearch -Z 5400 -T 25.50 RF00888.cm fasta
cmsearch -Z 5400 -T 27.20 RF00890.cm fasta
cmsearch -Z 5400 -T 29.75 RF00891.cm fasta
cmsearch -Z 5400 -T 20.40 RF00892.cm fasta
cmsearch -Z 5400 -T 42.50 RF00893.cm fasta
cmsearch -Z 5400 -T 29.75 RF00894.cm fasta
cmsearch -Z 5400 -T 42.50 RF00895.cm fasta
cmsearch -Z 5400 -T 42.50 RF00896.cm fasta
cmsearch -Z 5400 -T 21.25 RF00897.cm fasta
cmsearch -Z 5400 -T 25.50 RF00898.cm fasta
cmsearch -Z 5400 -T 21.25 RF00899.cm fasta
cmsearch -Z 5400 -T 17.85 RF00900.cm fasta
cmsearch -Z 5400 -T 17.00 RF00901.cm fasta
cmsearch -Z 5400 -T 29.75 RF00902.cm fasta
cmsearch -Z 5400 -T 25.50 RF00903.cm fasta
cmsearch -Z 5400 -T 42.50 RF00904.cm fasta
cmsearch -Z 5400 -T 17.00 RF00905.cm fasta
cmsearch -Z 5400 -T 42.50 RF00906.cm fasta
cmsearch -Z 5400 -T 17.85 RF00907.cm fasta
cmsearch -Z 5400 -T 18.70 RF00908.cm fasta
cmsearch -Z 5400 -T 25.50 RF00909.cm fasta
cmsearch -Z 5400 -T 51.00 RF00910.cm fasta
cmsearch -Z 5400 -T 59.50 RF00911.cm fasta
cmsearch -Z 5400 -T 38.25 RF00912.cm fasta
cmsearch -Z 5400 -T 68.00 RF00914.cm fasta
cmsearch -Z 5400 -T 46.75 RF00915.cm fasta
cmsearch -Z 5400 -T 31.45 RF00917.cm fasta
cmsearch -Z 5400 -T 39.10 RF00918.cm fasta
cmsearch -Z 5400 -T 29.75 RF00919.cm fasta
cmsearch -Z 5400 -T 18.70 RF00920.cm fasta
cmsearch -Z 5400 -T 23.80 -g RF00921.cm fasta

Table S17 – continued from previous page

command line
cmsearch -Z 5400 -T 17.00 RF00922.cm fasta
cmsearch -Z 5400 -T 85.00 RF00925.cm fasta
cmsearch -Z 5400 -T 18.70 RF00926.cm fasta
cmsearch -Z 5400 -T 68.00 RF00927.cm fasta
cmsearch -Z 5400 -T 18.70 RF00928.cm fasta
cmsearch -Z 5400 -T 25.50 RF00929.cm fasta
cmsearch -Z 5400 -T 17.85 RF00931.cm fasta
cmsearch -Z 5400 -T 59.50 RF00932.cm fasta
cmsearch -Z 5400 -T 17.00 RF00933.cm fasta
cmsearch -Z 5400 -T 17.00 RF00934.cm fasta
cmsearch -Z 5400 -T 17.00 RF00935.cm fasta
cmsearch -Z 5400 -T 38.25 RF00936.cm fasta
cmsearch -Z 5400 -T 33.15 RF00937.cm fasta
cmsearch -Z 5400 -T 38.25 RF00939.cm fasta
cmsearch -Z 5400 -T 17.00 RF00940.cm fasta
cmsearch -Z 5400 -T 15.30 RF00941.cm fasta
cmsearch -Z 5400 -T 25.50 RF00942.cm fasta
cmsearch -Z 5400 -T 170.00 RF00943.cm fasta
cmsearch -Z 5400 -T 17.00 RF00945.cm fasta
cmsearch -Z 5400 -T 29.75 RF00946.cm fasta
cmsearch -Z 5400 -T 17.00 RF00947.cm fasta
cmsearch -Z 5400 -T 17.00 RF00948.cm fasta
cmsearch -Z 5400 -T 17.00 RF00949.cm fasta
cmsearch -Z 5400 -T 17.00 RF00950.cm fasta
cmsearch -Z 5400 -T 57.80 RF00951.cm fasta
cmsearch -Z 5400 -T 38.25 RF00952.cm fasta
cmsearch -Z 5400 -T 17.00 RF00953.cm fasta
cmsearch -Z 5400 -T 17.00 RF00954.cm fasta
cmsearch -Z 5400 -T 17.00 RF00955.cm fasta
cmsearch -Z 5400 -T 17.00 RF00956.cm fasta
cmsearch -Z 5400 -T 18.70 RF00957.cm fasta
cmsearch -Z 5400 -T 29.75 -g RF00958.cm fasta
cmsearch -Z 5400 -T 25.50 RF00959.cm fasta
cmsearch -Z 5400 -T 17.00 RF00960.cm fasta
cmsearch -Z 5400 -T 18.70 RF00961.cm fasta
cmsearch -Z 5400 -T 17.00 RF00962.cm fasta
cmsearch -Z 5400 -T 17.00 RF00963.cm fasta
cmsearch -Z 5400 -T 17.00 RF00964.cm fasta
cmsearch -Z 5400 -T 17.00 RF00965.cm fasta
cmsearch -Z 5400 -T 28.90 RF00966.cm fasta
cmsearch -Z 5400 -T 17.00 RF00967.cm fasta
cmsearch -Z 5400 -T 23.80 RF00968.cm fasta
cmsearch -Z 5400 -T 17.00 RF00969.cm fasta
cmsearch -Z 5400 -T 17.00 RF00970.cm fasta
cmsearch -Z 5400 -T 17.00 RF00971.cm fasta
cmsearch -Z 5400 -T 26.35 RF00972.cm fasta
cmsearch -Z 5400 -T 30.60 RF00973.cm fasta
cmsearch -Z 5400 -T 45.05 RF00974.cm fasta
cmsearch -Z 5400 -T 46.75 RF00975.cm fasta

Table S17 – continued from previous page

command line
cmsearch -Z 5400 -T 20.40 RF00976.cm fasta
cmsearch -Z 5400 -T 29.75 RF00977.cm fasta
cmsearch -Z 5400 -T 17.00 RF00978.cm fasta
cmsearch -Z 5400 -T 17.00 RF00979.cm fasta
cmsearch -Z 5400 -T 17.85 RF00980.cm fasta
cmsearch -Z 5400 -T 25.50 RF00981.cm fasta
cmsearch -Z 5400 -T 51.00 RF00982.cm fasta
cmsearch -Z 5400 -T 22.10 RF00983.cm fasta
cmsearch -Z 5400 -T 17.00 RF00984.cm fasta
cmsearch -Z 5400 -T 28.90 RF00985.cm fasta
cmsearch -Z 5400 -T 17.00 RF00986.cm fasta
cmsearch -Z 5400 -T 17.00 RF00987.cm fasta
cmsearch -Z 5400 -T 17.00 RF00988.cm fasta
cmsearch -Z 5400 -T 23.80 RF00989.cm fasta
cmsearch -Z 5400 -T 42.50 RF00990.cm fasta
cmsearch -Z 5400 -T 19.55 RF00991.cm fasta
cmsearch -Z 5400 -T 17.00 RF00992.cm fasta
cmsearch -Z 5400 -T 17.00 RF00993.cm fasta
cmsearch -Z 5400 -T 67.15 RF00994.cm fasta
cmsearch -Z 5400 -T 17.00 RF00995.cm fasta
cmsearch -Z 5400 -T 23.80 RF00996.cm fasta
cmsearch -Z 5400 -T 18.70 RF00997.cm fasta
cmsearch -Z 5400 -T 57.80 RF00998.cm fasta
cmsearch -Z 5400 -T 17.00 RF00999.cm fasta
cmsearch -Z 5400 -T 17.00 RF01000.cm fasta
cmsearch -Z 5400 -T 17.85 RF01001.cm fasta
cmsearch -Z 5400 -T 19.55 RF01002.cm fasta
cmsearch -Z 5400 -T 17.00 RF01003.cm fasta
cmsearch -Z 5400 -T 17.00 RF01004.cm fasta
cmsearch -Z 5400 -T 19.55 RF01005.cm fasta
cmsearch -Z 5400 -T 35.70 RF01006.cm fasta
cmsearch -Z 5400 -T 17.85 RF01007.cm fasta
cmsearch -Z 5400 -T 17.00 RF01008.cm fasta
cmsearch -Z 5400 -T 17.00 RF01009.cm fasta
cmsearch -Z 5400 -T 28.90 RF01010.cm fasta
cmsearch -Z 5400 -T 19.55 RF01011.cm fasta
cmsearch -Z 5400 -T 34.00 RF01012.cm fasta
cmsearch -Z 5400 -T 17.00 RF01013.cm fasta
cmsearch -Z 5400 -T 29.75 RF01014.cm fasta
cmsearch -Z 5400 -T 42.50 RF01015.cm fasta
cmsearch -Z 5400 -T 42.50 RF01016.cm fasta
cmsearch -Z 5400 -T 17.00 RF01017.cm fasta
cmsearch -Z 5400 -T 27.20 RF01018.cm fasta
cmsearch -Z 5400 -T 17.00 RF01019.cm fasta
cmsearch -Z 5400 -T 17.00 RF01020.cm fasta
cmsearch -Z 5400 -T 25.50 RF01021.cm fasta
cmsearch -Z 5400 -T 19.55 RF01022.cm fasta
cmsearch -Z 5400 -T 28.90 RF01023.cm fasta
cmsearch -Z 5400 -T 17.00 RF01024.cm fasta

Table S17 – continued from previous page

command line
cmsearch -Z 5400 -T 15.30 RF01025.cm fasta
cmsearch -Z 5400 -T 15.30 RF01026.cm fasta
cmsearch -Z 5400 -T 17.00 RF01027.cm fasta
cmsearch -Z 5400 -T 17.00 RF01028.cm fasta
cmsearch -Z 5400 -T 51.00 RF01029.cm fasta
cmsearch -Z 5400 -T 17.00 RF01030.cm fasta
cmsearch -Z 5400 -T 30.60 RF01031.cm fasta
cmsearch -Z 5400 -T 17.00 RF01032.cm fasta
cmsearch -Z 5400 -T 46.75 RF01033.cm fasta
cmsearch -Z 5400 -T 19.55 RF01034.cm fasta
cmsearch -Z 5400 -T 17.00 RF01035.cm fasta
cmsearch -Z 5400 -T 20.40 RF01036.cm fasta
cmsearch -Z 5400 -T 22.10 RF01037.cm fasta
cmsearch -Z 5400 -T 29.75 RF01038.cm fasta
cmsearch -Z 5400 -T 21.25 RF01039.cm fasta
cmsearch -Z 5400 -T 17.00 RF01040.cm fasta
cmsearch -Z 5400 -T 17.00 RF01041.cm fasta
cmsearch -Z 5400 -T 19.55 RF01042.cm fasta
cmsearch -Z 5400 -T 38.25 RF01043.cm fasta
cmsearch -Z 5400 -T 30.60 RF01044.cm fasta
cmsearch -Z 5400 -T 56.95 RF01045.cm fasta
cmsearch -Z 5400 -T 17.85 RF01046.cm fasta
cmsearch -Z 5400 -T 28.05 RF01047.cm fasta
cmsearch -Z 5400 -T 27.20 -g RF01050.cm fasta
cmsearch -Z 5400 -T 27.20 RF01051.cm fasta
cmsearch -Z 5400 -T 28.05 RF01052.cm fasta
cmsearch -Z 5400 -T 17.00 RF01053.cm fasta
cmsearch -Z 5400 -T 17.00 RF01054.cm fasta
cmsearch -Z 5400 -T 23.80 RF01055.cm fasta
cmsearch -Z 5400 -T 17.00 RF01056.cm fasta
cmsearch -Z 5400 -T 19.55 –fil-no-hmm RF01057.cm fasta
cmsearch -Z 5400 -T 112.20 RF01058.cm fasta
cmsearch -Z 5400 -T 33.15 RF01059.cm fasta
cmsearch -Z 5400 -T 68.00 RF01061.cm fasta
cmsearch -Z 5400 -T 63.75 RF01062.cm fasta
cmsearch -Z 5400 -T 19.55 RF01063.cm fasta
cmsearch -Z 5400 -T 34.00 RF01064.cm fasta
cmsearch -Z 5400 -T 38.25 RF01065.cm fasta
cmsearch -Z 5400 -T 20.40 RF01066.cm fasta
cmsearch -Z 5400 -T 22.10 RF01067.cm fasta
cmsearch -Z 5400 -T 19.55 RF01068.cm fasta
cmsearch -Z 5400 -T 21.25 RF01069.cm fasta
cmsearch -Z 5400 -T 22.10 RF01070.cm fasta
cmsearch -Z 5400 -T 85.00 RF01071.cm fasta
cmsearch -Z 5400 -T 19.55 RF01072.cm fasta
cmsearch -Z 5400 -T 32.30 RF01073.cm fasta
cmsearch -Z 5400 -T 17.00 RF01074.cm fasta
cmsearch -Z 5400 -T 17.00 RF01075.cm fasta
cmsearch -Z 5400 -T 17.00 RF01076.cm fasta

Table S17 – continued from previous page

command line
cmsearch -Z 5400 -T 17.00 RF01077.cm fasta
cmsearch -Z 5400 -T 17.00 RF01078.cm fasta
cmsearch -Z 5400 -T 17.00 RF01079.cm fasta
cmsearch -Z 5400 -T 17.00 RF01080.cm fasta
cmsearch -Z 5400 -T 16.447500 RF01081.cm fasta
cmsearch -Z 5400 -T 17.00 RF01082.cm fasta
cmsearch -Z 5400 -T 25.50 RF01083.cm fasta
cmsearch -Z 5400 -T 38.25 RF01084.cm fasta
cmsearch -Z 5400 -T 17.00 RF01085.cm fasta
cmsearch -Z 5400 -T 52.70 RF01086.cm fasta
cmsearch -Z 5400 -T 27.20 RF01087.cm fasta
cmsearch -Z 5400 -T 17.00 RF01088.cm fasta
cmsearch -Z 5400 -T 17.00 RF01089.cm fasta
cmsearch -Z 5400 -T 35.70 RF01090.cm fasta
cmsearch -Z 5400 -T 17.00 RF01091.cm fasta
cmsearch -Z 5400 -T 17.00 RF01092.cm fasta
cmsearch -Z 5400 -T 28.90 RF01093.cm fasta
cmsearch -Z 5400 -T 17.00 RF01094.cm fasta
cmsearch -Z 5400 -T 17.00 RF01095.cm fasta
cmsearch -Z 5400 -T 17.00 RF01096.cm fasta
cmsearch -Z 5400 -T 36.55 RF01097.cm fasta
cmsearch -Z 5400 -T 24.65 RF01098.cm fasta
cmsearch -Z 5400 -T 39.95 RF01099.cm fasta
cmsearch -Z 5400 -T 17.00 RF01100.cm fasta
cmsearch -Z 5400 -T 17.00 RF01101.cm fasta
cmsearch -Z 5400 -T 17.00 RF01102.cm fasta
cmsearch -Z 5400 -T 29.75 RF01103.cm fasta
cmsearch -Z 5400 -T 17.00 RF01104.cm fasta
cmsearch -Z 5400 -T 16.745000 RF01105.cm fasta
cmsearch -Z 5400 -T 17.00 RF01106.cm fasta
cmsearch -Z 5400 -T 17.00 RF01107.cm fasta
cmsearch -Z 5400 -T 17.00 RF01108.cm fasta
cmsearch -Z 5400 -T 17.00 RF01109.cm fasta
cmsearch -Z 5400 -T 17.00 RF01110.cm fasta
cmsearch -Z 5400 -T 27.20 RF01111.cm fasta
cmsearch -Z 5400 -T 17.00 RF01112.cm fasta
cmsearch -Z 5400 -T 17.00 RF01113.cm fasta
cmsearch -Z 5400 -T 23.80 RF01114.cm fasta
cmsearch -Z 5400 -T 21.25 RF01115.cm fasta
cmsearch -Z 5400 -T 21.25 RF01116.cm fasta
cmsearch -Z 5400 -T 17.00 RF01117.cm fasta
cmsearch -Z 5400 -T 79.90 RF01118.cm fasta
cmsearch -Z 5400 -T 17.00 RF01119.cm fasta
cmsearch -Z 5400 -T 17.00 RF01120.cm fasta
cmsearch -Z 5400 -T 42.50 RF01121.cm fasta
cmsearch -Z 5400 -T 34.00 RF01122.cm fasta
cmsearch -Z 5400 -T 17.00 RF01123.cm fasta
cmsearch -Z 5400 -T 38.25 RF01124.cm fasta
cmsearch -Z 5400 -T 28.90 RF01125.cm fasta

Table S17 – continued from previous page

command line
cmsearch -Z 5400 -T 29.75 RF01126.cm fasta
cmsearch -Z 5400 -T 51.00 RF01127.cm fasta
cmsearch -Z 5400 -T 28.90 -g RF01128.cm fasta
cmsearch -Z 5400 -T 34.00 RF01129.cm fasta
cmsearch -Z 5400 -T 38.25 RF01130.cm fasta
cmsearch -Z 5400 -T 40.80 RF01131.cm fasta
cmsearch -Z 5400 -T 17.00 RF01132.cm fasta
cmsearch -Z 5400 -T 25.50 –fil-no-hmm RF01133.cm fasta
cmsearch -Z 5400 -T 42.50 RF01134.cm fasta
cmsearch -Z 5400 -T 42.50 -g RF01135.cm fasta
cmsearch -Z 5400 -T 40.80 RF01136.cm fasta
cmsearch -Z 5400 -T 17.00 RF01137.cm fasta
cmsearch -Z 5400 -T 17.00 RF01138.cm fasta
cmsearch -Z 5400 -T 17.00 RF01139.cm fasta
cmsearch -Z 5400 -T 25.50 RF01140.cm fasta
cmsearch -Z 5400 -T 25.50 RF01141.cm fasta
cmsearch -Z 5400 -T 34.00 RF01142.cm fasta
cmsearch -Z 5400 -T 17.85 RF01143.cm fasta
cmsearch -Z 5400 -T 42.50 RF01144.cm fasta
cmsearch -Z 5400 -T 18.70 RF01145.cm fasta
cmsearch -Z 5400 -T 17.00 RF01146.cm fasta
cmsearch -Z 5400 -T 19.55 RF01147.cm fasta
cmsearch -Z 5400 -T 25.50 RF01148.cm fasta
cmsearch -Z 5400 -T 38.25 RF01149.cm fasta
cmsearch -Z 5400 -T 13.60 RF01150.cm fasta
cmsearch -Z 5400 -T 22.10 RF01151.cm fasta
cmsearch -Z 5400 -T 17.85 RF01152.cm fasta
cmsearch -Z 5400 -T 51.00 RF01153.cm fasta
cmsearch -Z 5400 -T 17.00 RF01154.cm fasta
cmsearch -Z 5400 -T 17.00 RF01155.cm fasta
cmsearch -Z 5400 -T 22.95 RF01156.cm fasta
cmsearch -Z 5400 -T 46.75 RF01157.cm fasta
cmsearch -Z 5400 -T 42.50 RF01158.cm fasta
cmsearch -Z 5400 -T 17.00 RF01159.cm fasta
cmsearch -Z 5400 -T 16.677000 RF01160.cm fasta
cmsearch -Z 5400 -T 18.70 RF01161.cm fasta
cmsearch -Z 5400 -T 42.50 RF01162.cm fasta
cmsearch -Z 5400 -T 17.00 RF01163.cm fasta
cmsearch -Z 5400 -T 28.90 RF01164.cm fasta
cmsearch -Z 5400 -T 38.25 RF01165.cm fasta
cmsearch -Z 5400 -T 17.00 RF01166.cm fasta
cmsearch -Z 5400 -T 38.25 RF01167.cm fasta
cmsearch -Z 5400 -T 25.50 RF01168.cm fasta
cmsearch -Z 5400 -T 17.00 RF01169.cm fasta
cmsearch -Z 5400 -T 17.00 RF01170.cm fasta
cmsearch -Z 5400 -T 17.00 RF01171.cm fasta
cmsearch -Z 5400 -T 51.00 RF01172.cm fasta
cmsearch -Z 5400 -T 28.90 RF01173.cm fasta
cmsearch -Z 5400 -T 17.00 RF01174.cm fasta

Table S17 – continued from previous page

command line
cmsearch -Z 5400 -T 17.00 RF01175.cm fasta
cmsearch -Z 5400 -T 24.65 RF01176.cm fasta
cmsearch -Z 5400 -T 24.65 RF01177.cm fasta
cmsearch -Z 5400 -T 31.45 RF01178.cm fasta
cmsearch -Z 5400 -T 31.45 RF01179.cm fasta
cmsearch -Z 5400 -T 25.50 RF01180.cm fasta
cmsearch -Z 5400 -T 42.50 RF01181.cm fasta
cmsearch -Z 5400 -T 20.40 RF01182.cm fasta
cmsearch -Z 5400 -T 22.95 RF01183.cm fasta
cmsearch -Z 5400 -T 20.40 RF01184.cm fasta
cmsearch -Z 5400 -T 29.75 RF01185.cm fasta
cmsearch -Z 5400 -T 32.30 RF01186.cm fasta
cmsearch -Z 5400 -T 17.00 RF01187.cm fasta
cmsearch -Z 5400 -T 18.70 RF01188.cm fasta
cmsearch -Z 5400 -T 51.00 RF01189.cm fasta
cmsearch -Z 5400 -T 17.586500 RF01190.cm fasta
cmsearch -Z 5400 -T 23.80 RF01191.cm fasta
cmsearch -Z 5400 -T 22.95 RF01192.cm fasta
cmsearch -Z 5400 -T 25.50 RF01193.cm fasta
cmsearch -Z 5400 -T 59.50 RF01194.cm fasta
cmsearch -Z 5400 -T 26.35 RF01195.cm fasta
cmsearch -Z 5400 -T 17.00 RF01196.cm fasta
cmsearch -Z 5400 -T 22.10 RF01197.cm fasta
cmsearch -Z 5400 -T 17.00 RF01198.cm fasta
cmsearch -Z 5400 -T 17.00 RF01199.cm fasta
cmsearch -Z 5400 -T 42.50 RF01200.cm fasta
cmsearch -Z 5400 -T 37.40 RF01201.cm fasta
cmsearch -Z 5400 -T 51.00 RF01202.cm fasta
cmsearch -Z 5400 -T 21.25 RF01203.cm fasta
cmsearch -Z 5400 -T 17.00 RF01204.cm fasta
cmsearch -Z 5400 -T 17.00 RF01205.cm fasta
cmsearch -Z 5400 -T 17.00 RF01206.cm fasta
cmsearch -Z 5400 -T 21.25 RF01207.cm fasta
cmsearch -Z 5400 -T 17.00 RF01208.cm fasta
cmsearch -Z 5400 -T 28.90 RF01209.cm fasta
cmsearch -Z 5400 -T 93.50 RF01210.cm fasta
cmsearch -Z 5400 -T 28.90 RF01211.cm fasta
cmsearch -Z 5400 -T 19.55 RF01212.cm fasta
cmsearch -Z 5400 -T 29.75 RF01213.cm fasta
cmsearch -Z 5400 -T 25.415000 RF01214.cm fasta
cmsearch -Z 5400 -T 35.70 RF01215.cm fasta
cmsearch -Z 5400 -T 17.00 RF01216.cm fasta
cmsearch -Z 5400 -T 23.80 RF01217.cm fasta
cmsearch -Z 5400 -T 42.50 RF01218.cm fasta
cmsearch -Z 5400 -T 23.80 RF01219.cm fasta
cmsearch -Z 5400 -T 38.25 RF01220.cm fasta
cmsearch -Z 5400 -T 25.50 RF01221.cm fasta
cmsearch -Z 5400 -T 68.00 RF01222.cm fasta
cmsearch -Z 5400 -T 17.85 RF01223.cm fasta

Table S17 – continued from previous page

command line
cmsearch -Z 5400 -T 34.00 RF01224.cm fasta
cmsearch -Z 5400 -T 25.50 RF01225.cm fasta
cmsearch -Z 5400 -T 17.00 RF01226.cm fasta
cmsearch -Z 5400 -T 17.00 RF01227.cm fasta
cmsearch -Z 5400 -T 29.75 RF01228.cm fasta
cmsearch -Z 5400 -T 23.80 RF01229.cm fasta
cmsearch -Z 5400 -T 17.00 RF01230.cm fasta
cmsearch -Z 5400 -T 21.25 RF01231.cm fasta
cmsearch -Z 5400 -T 17.00 RF01232.cm fasta
cmsearch -Z 5400 -T 25.50 RF01233.cm fasta
cmsearch -Z 5400 -T 34.00 RF01234.cm fasta
cmsearch -Z 5400 -T 17.00 RF01235.cm fasta
cmsearch -Z 5400 -T 25.50 RF01236.cm fasta
cmsearch -Z 5400 -T 25.50 RF01237.cm fasta
cmsearch -Z 5400 -T 21.25 RF01238.cm fasta
cmsearch -Z 5400 -T 25.50 RF01239.cm fasta
cmsearch -Z 5400 -T 25.50 RF01240.cm fasta
cmsearch -Z 5400 -T 21.25 RF01241.cm fasta
cmsearch -Z 5400 -T 24.65 RF01242.cm fasta
cmsearch -Z 5400 -T 21.25 RF01243.cm fasta
cmsearch -Z 5400 -T 17.00 RF01244.cm fasta
cmsearch -Z 5400 -T 17.00 RF01245.cm fasta
cmsearch -Z 5400 -T 28.05 RF01246.cm fasta
cmsearch -Z 5400 -T 24.65 RF01247.cm fasta
cmsearch -Z 5400 -T 38.25 RF01248.cm fasta
cmsearch -Z 5400 -T 19.55 RF01249.cm fasta
cmsearch -Z 5400 -T 26.35 RF01250.cm fasta
cmsearch -Z 5400 -T 22.10 RF01251.cm fasta
cmsearch -Z 5400 -T 17.00 RF01252.cm fasta
cmsearch -Z 5400 -T 25.50 RF01253.cm fasta
cmsearch -Z 5400 -T 20.40 RF01254.cm fasta
cmsearch -Z 5400 -T 25.50 RF01255.cm fasta
cmsearch -Z 5400 -T 23.80 RF01256.cm fasta
cmsearch -Z 5400 -T 29.75 RF01257.cm fasta
cmsearch -Z 5400 -T 59.50 RF01258.cm fasta
cmsearch -Z 5400 -T 27.20 RF01259.cm fasta
cmsearch -Z 5400 -T 17.00 RF01260.cm fasta
cmsearch -Z 5400 -T 20.40 RF01261.cm fasta
cmsearch -Z 5400 -T 26.35 RF01262.cm fasta
cmsearch -Z 5400 -T 38.25 RF01263.cm fasta
cmsearch -Z 5400 -T 48.45 RF01264.cm fasta
cmsearch -Z 5400 -T 27.20 RF01265.cm fasta
cmsearch -Z 5400 -T 34.00 RF01266.cm fasta
cmsearch -Z 5400 -T 56.95 RF01267.cm fasta
cmsearch -Z 5400 -T 68.00 RF01268.cm fasta
cmsearch -Z 5400 -T 22.95 RF01269.cm fasta
cmsearch -Z 5400 -T 92.65 RF01270.cm fasta
cmsearch -Z 5400 -T 107.10 RF01271.cm fasta
cmsearch -Z 5400 -T 188.70 RF01272.cm fasta

Table S17 – continued from previous page

command line
cmsearch -Z 5400 -T 17.00 RF01273.cm fasta
cmsearch -Z 5400 -T 32.30 RF01274.cm fasta
cmsearch -Z 5400 -T 34.00 RF01275.cm fasta
cmsearch -Z 5400 -T 51.00 RF01276.cm fasta
cmsearch -Z 5400 -T 23.80 RF01277.cm fasta
cmsearch -Z 5400 -T 29.75 RF01278.cm fasta
cmsearch -Z 5400 -T 16.15 RF01279.cm fasta
cmsearch -Z 5400 -T 17.00 RF01280.cm fasta
cmsearch -Z 5400 -T 34.00 RF01281.cm fasta
cmsearch -Z 5400 -T 29.75 RF01283.cm fasta
cmsearch -Z 5400 -T 27.20 RF01284.cm fasta
cmsearch -Z 5400 -T 17.00 RF01285.cm fasta
cmsearch -Z 5400 -T 17.00 RF01286.cm fasta
cmsearch -Z 5400 -T 19.55 RF01287.cm fasta
cmsearch -Z 5400 -T 17.00 RF01288.cm fasta
cmsearch -Z 5400 -T 17.00 RF01289.cm fasta
cmsearch -Z 5400 -T 26.35 RF01290.cm fasta
cmsearch -Z 5400 -T 29.75 RF01291.cm fasta
cmsearch -Z 5400 -T 27.20 RF01292.cm fasta
cmsearch -Z 5400 -T 22.10 RF01293.cm fasta
cmsearch -Z 5400 -T 34.85 RF01294.cm fasta
cmsearch -Z 5400 -T 49.30 RF01295.cm fasta
cmsearch -Z 5400 -T 30.60 RF01296.cm fasta
cmsearch -Z 5400 -T 17.00 RF01297.cm fasta
cmsearch -Z 5400 -T 17.00 RF01298.cm fasta
cmsearch -Z 5400 -T 20.40 RF01299.cm fasta
cmsearch -Z 5400 -T 17.00 RF01300.cm fasta
cmsearch -Z 5400 -T 17.00 RF01301.cm fasta
cmsearch -Z 5400 -T 32.30 RF01302.cm fasta
cmsearch -Z 5400 -T 24.65 RF01303.cm fasta
cmsearch -Z 5400 -T 25.50 RF01304.cm fasta
cmsearch -Z 5400 -T 22.10 RF01305.cm fasta
cmsearch -Z 5400 -T 42.50 RF01306.cm fasta
cmsearch -Z 5400 -T 29.75 RF01307.cm fasta
cmsearch -Z 5400 -T 46.75 RF01308.cm fasta
cmsearch -Z 5400 -T 38.25 RF01309.cm fasta
cmsearch -Z 5400 -T 17.00 RF01310.cm fasta
cmsearch -Z 5400 -T 21.25 RF01311.cm fasta
cmsearch -Z 5400 -T 25.50 RF01312.cm fasta
cmsearch -Z 5400 -T 17.85 RF01313.cm fasta
cmsearch -Z 5400 -T 17.00 RF01314.cm fasta
cmsearch -Z 5400 -T 18.70 RF01315.cm fasta
cmsearch -Z 5400 -T 26.35 RF01316.cm fasta
cmsearch -Z 5400 -T 28.05 RF01317.cm fasta
cmsearch -Z 5400 -T 21.25 RF01318.cm fasta
cmsearch -Z 5400 -T 28.05 RF01319.cm fasta
cmsearch -Z 5400 -T 24.65 RF01320.cm fasta
cmsearch -Z 5400 -T 13.60 RF01321.cm fasta
cmsearch -Z 5400 -T 20.40 RF01322.cm fasta

Table S17 – continued from previous page

command line
cmsearch -Z 5400 -T 20.40 RF01323.cm fasta
cmsearch -Z 5400 -T 38.25 RF01324.cm fasta
cmsearch -Z 5400 -T 21.25 RF01325.cm fasta
cmsearch -Z 5400 -T 23.80 RF01326.cm fasta
cmsearch -Z 5400 -T 13.540500 RF01327.cm fasta
cmsearch -Z 5400 -T 26.35 RF01328.cm fasta
cmsearch -Z 5400 -T 38.25 RF01329.cm fasta
cmsearch -Z 5400 -T 17.00 RF01330.cm fasta
cmsearch -Z 5400 -T 22.10 RF01331.cm fasta
cmsearch -Z 5400 -T 22.10 RF01332.cm fasta
cmsearch -Z 5400 -T 17.00 RF01333.cm fasta
cmsearch -Z 5400 -T 34.00 RF01334.cm fasta
cmsearch -Z 5400 -T 22.10 RF01335.cm fasta
cmsearch -Z 5400 -T 20.40 RF01336.cm fasta
cmsearch -Z 5400 -T 22.95 RF01337.cm fasta
cmsearch -Z 5400 -T 26.35 RF01338.cm fasta
cmsearch -Z 5400 -T 23.80 RF01339.cm fasta
cmsearch -Z 5400 -T 17.00 RF01340.cm fasta
cmsearch -Z 5400 -T 21.08 RF01341.cm fasta
cmsearch -Z 5400 -T 15.30 RF01342.cm fasta
cmsearch -Z 5400 -T 17.00 RF01343.cm fasta
cmsearch -Z 5400 -T 20.40 RF01344.cm fasta
cmsearch -Z 5400 -T 17.00 RF01345.cm fasta
cmsearch -Z 5400 -T 28.90 RF01346.cm fasta
cmsearch -Z 5400 -T 17.00 RF01347.cm fasta
cmsearch -Z 5400 -T 23.80 RF01348.cm fasta
cmsearch -Z 5400 -T 16.83 RF01349.cm fasta
cmsearch -Z 5400 -T 24.65 RF01350.cm fasta
cmsearch -Z 5400 -T 16.286000 RF01351.cm fasta
cmsearch -Z 5400 -T 17.00 RF01352.cm fasta
cmsearch -Z 5400 -T 17.00 RF01353.cm fasta
cmsearch -Z 5400 -T 28.90 RF01354.cm fasta
cmsearch -Z 5400 -T 27.20 RF01355.cm fasta
cmsearch -Z 5400 -T 13.60 RF01356.cm fasta
cmsearch -Z 5400 -T 17.00 RF01357.cm fasta
cmsearch -Z 5400 -T 17.00 RF01358.cm fasta
cmsearch -Z 5400 -T 18.70 RF01359.cm fasta
cmsearch -Z 5400 -T 17.748000 RF01360.cm fasta
cmsearch -Z 5400 -T 17.00 RF01361.cm fasta
cmsearch -Z 5400 -T 17.00 RF01362.cm fasta
cmsearch -Z 5400 -T 17.00 RF01363.cm fasta
cmsearch -Z 5400 -T 17.00 RF01364.cm fasta
cmsearch -Z 5400 -T 17.00 RF01365.cm fasta
cmsearch -Z 5400 -T 17.00 RF01366.cm fasta
cmsearch -Z 5400 -T 17.00 RF01367.cm fasta
cmsearch -Z 5400 -T 16.294500 RF01368.cm fasta
cmsearch -Z 5400 -T 17.00 RF01369.cm fasta
cmsearch -Z 5400 -T 25.50 RF01370.cm fasta
cmsearch -Z 5400 -T 20.40 RF01371.cm fasta

Table S17 – continued from previous page

command line
cmsearch -Z 5400 -T 16.923500 RF01372.cm fasta
cmsearch -Z 5400 -T 22.95 RF01373.cm fasta
cmsearch -Z 5400 -T 29.75 RF01374.cm fasta
cmsearch -Z 5400 -T 34.00 RF01375.cm fasta
cmsearch -Z 5400 -T 29.75 RF01376.cm fasta
cmsearch -Z 5400 -T 34.00 RF01377.cm fasta
cmsearch -Z 5400 -T 36.55 RF01378.cm fasta
cmsearch -Z 5400 -T 32.30 RF01379.cm fasta
cmsearch -Z 5400 -T 17.00 RF01380.cm fasta
cmsearch -Z 5400 -T 19.55 RF01381.cm fasta
cmsearch -Z 5400 -T 18.70 RF01382.cm fasta
cmsearch -Z 5400 -T 10.20 RF01383.cm fasta
cmsearch -Z 5400 -T 51.00 RF01384.cm fasta
cmsearch -Z 5400 -T 16.15 RF01385.cm fasta
cmsearch -Z 5400 -T 25.50 RF01386.cm fasta
cmsearch -Z 5400 -T 19.55 RF01387.cm fasta
cmsearch -Z 5400 -T 29.75 RF01388.cm fasta
cmsearch -Z 5400 -T 127.50 RF01389.cm fasta
cmsearch -Z 5400 -T 85.00 RF01390.cm fasta
cmsearch -Z 5400 -T 42.50 RF01391.cm fasta
cmsearch -Z 5400 -T 38.25 RF01392.cm fasta
cmsearch -Z 5400 -T 23.80 RF01393.cm fasta
cmsearch -Z 5400 -T 18.70 RF01394.cm fasta
cmsearch -Z 5400 -T 29.75 -g RF01395.cm fasta
cmsearch -Z 5400 -T 37.40 RF01396.cm fasta
cmsearch -Z 5400 -T 29.75 RF01397.cm fasta
cmsearch -Z 5400 -T 38.25 RF01398.cm fasta
cmsearch -Z 5400 -T 51.00 RF01399.cm fasta
cmsearch -Z 5400 -T 38.25 RF01400.cm fasta
cmsearch -Z 5400 -T 21.25 RF01401.cm fasta
cmsearch -Z 5400 -T 30.60 RF01402.cm fasta
cmsearch -Z 5400 -T 51.00 RF01403.cm fasta
cmsearch -Z 5400 -T 51.00 RF01404.cm fasta
cmsearch -Z 5400 -T 29.75 RF01405.cm fasta
cmsearch -Z 5400 -T 67.15 RF01406.cm fasta
cmsearch -Z 5400 -T 102.00 RF01407.cm fasta
cmsearch -Z 5400 -T 22.10 RF01408.cm fasta
cmsearch -Z 5400 -T 30.60 RF01409.cm fasta
cmsearch -Z 5400 -T 29.75 RF01410.cm fasta
cmsearch -Z 5400 -T 42.50 RF01411.cm fasta
cmsearch -Z 5400 -T 34.00 RF01412.cm fasta
cmsearch -Z 5400 -T 17.255000 RF01413.cm fasta
cmsearch -Z 5400 -T 17.00 RF01414.cm fasta
cmsearch -Z 5400 -T 37.40 RF01415.cm fasta
cmsearch -Z 5400 -T 25.50 RF01416.cm fasta
cmsearch -Z 5400 -T 28.90 RF01417.cm fasta
cmsearch -Z 5400 -T 45.90 RF01418.cm fasta
cmsearch -Z 5400 -T 44.20 RF01419.cm fasta
cmsearch -Z 5400 -T 25.50 RF01420.cm fasta

Table S17 – continued from previous page

command line
cmsearch -Z 5400 -T 18.70 RF01421.cm fasta
cmsearch -Z 5400 -T 34.00 RF01422.cm fasta
cmsearch -Z 5400 -T 29.75 RF01423.cm fasta
cmsearch -Z 5400 -T 29.75 RF01424.cm fasta
cmsearch -Z 5400 -T 38.25 RF01425.cm fasta
cmsearch -Z 5400 -T 34.00 RF01426.cm fasta
cmsearch -Z 5400 -T 17.85 -g RF01427.cm fasta
cmsearch -Z 5400 -T 25.50 RF01428.cm fasta
cmsearch -Z 5400 -T 25.50 RF01429.cm fasta
cmsearch -Z 5400 -T 34.00 RF01430.cm fasta
cmsearch -Z 5400 -T 29.75 RF01431.cm fasta
cmsearch -Z 5400 -T 42.50 RF01432.cm fasta
cmsearch -Z 5400 -T 25.50 RF01433.cm fasta
cmsearch -Z 5400 -T 85.00 RF01434.cm fasta
cmsearch -Z 5400 -T 68.00 RF01435.cm fasta
cmsearch -Z 5400 -T 68.00 RF01436.cm fasta
cmsearch -Z 5400 -T 42.50 RF01437.cm fasta
cmsearch -Z 5400 -T 68.00 RF01438.cm fasta
cmsearch -Z 5400 -T 85.00 RF01439.cm fasta
cmsearch -Z 5400 -T 76.50 RF01440.cm fasta
cmsearch -Z 5400 -T 51.00 RF01441.cm fasta
cmsearch -Z 5400 -T 68.00 RF01442.cm fasta
cmsearch -Z 5400 -T 42.50 RF01443.cm fasta
cmsearch -Z 5400 -T 68.00 RF01444.cm fasta
cmsearch -Z 5400 -T 85.00 RF01445.cm fasta
cmsearch -Z 5400 -T 51.00 RF01446.cm fasta
cmsearch -Z 5400 -T 85.00 RF01447.cm fasta
cmsearch -Z 5400 -T 42.50 RF01448.cm fasta
cmsearch -Z 5400 -T 68.00 RF01449.cm fasta
cmsearch -Z 5400 -T 85.00 RF01450.cm fasta
cmsearch -Z 5400 -T 4.25 RF01451.cm fasta
cmsearch -Z 5400 -T 76.50 RF01452.cm fasta
cmsearch -Z 5400 -T 85.00 RF01453.cm fasta
cmsearch -Z 5400 -T 63.75 RF01454.cm fasta
cmsearch -Z 5400 -T 22.10 RF01455.cm fasta
cmsearch -Z 5400 -T 38.25 RF01456.cm fasta
cmsearch -Z 5400 -T 68.00 RF01457.cm fasta
cmsearch -Z 5400 -T 20.40 RF01458.cm fasta
cmsearch -Z 5400 -T 76.50 RF01459.cm fasta
cmsearch -Z 5400 -T 212.50 RF01460.cm fasta
cmsearch -Z 5400 -T 85.00 RF01461.cm fasta
cmsearch -Z 5400 -T 85.00 RF01462.cm fasta
cmsearch -Z 5400 -T 34.00 RF01463.cm fasta
cmsearch -Z 5400 -T 153.00 RF01464.cm fasta
cmsearch -Z 5400 -T 68.00 RF01465.cm fasta
cmsearch -Z 5400 -T 23.80 RF01466.cm fasta
cmsearch -Z 5400 -T 24.65 RF01467.cm fasta
cmsearch -Z 5400 -T 85.00 RF01468.cm fasta
cmsearch -Z 5400 -T 14.45 -g RF01469.cm fasta

Table S17 – continued from previous page

command line
cmsearch -Z 5400 -T 22.95 RF01470.cm fasta
cmsearch -Z 5400 -T 38.25 RF01471.cm fasta
cmsearch -Z 5400 -T 212.50 RF01472.cm fasta
cmsearch -Z 5400 -T 425.00 RF01473.cm fasta
cmsearch -Z 5400 -T 18.70 -g RF01474.cm fasta
cmsearch -Z 5400 -T 51.00 RF01475.cm fasta
cmsearch -Z 5400 -T 85.00 RF01476.cm fasta
cmsearch -Z 5400 -T 46.75 RF01477.cm fasta
cmsearch -Z 5400 -T 170.00 RF01478.cm fasta
cmsearch -Z 5400 -T 85.00 RF01479.cm fasta
cmsearch -Z 5400 -T 63.75 RF01480.cm fasta
cmsearch -Z 5400 -T 26.35 RF01481.cm fasta
cmsearch -Z 5400 -T 42.50 RF01482.cm fasta
cmsearch -Z 5400 -T 68.00 RF01483.cm fasta
cmsearch -Z 5400 -T 170.00 RF01484.cm fasta
cmsearch -Z 5400 -T 34.00 RF01485.cm fasta
cmsearch -Z 5400 -T 127.50 RF01486.cm fasta
cmsearch -Z 5400 -T 20.40 RF01487.cm fasta
cmsearch -Z 5400 -T 34.00 RF01488.cm fasta
cmsearch -Z 5400 -T 38.25 RF01489.cm fasta
cmsearch -Z 5400 -T 29.75 RF01490.cm fasta
cmsearch -Z 5400 -T 382.50 RF01491.cm fasta
cmsearch -Z 5400 -T 35.70 RF01492.cm fasta
cmsearch -Z 5400 -T 76.50 RF01493.cm fasta
cmsearch -Z 5400 -T 42.50 RF01494.cm fasta
cmsearch -Z 5400 -T 18.70 RF01495.cm fasta
cmsearch -Z 5400 -T 47.60 RF01496.cm fasta
cmsearch -Z 5400 -T 34.00 RF01497.cm fasta
cmsearch -Z 5400 -T 21.25 RF01498.cm fasta
cmsearch -Z 5400 -T 51.00 RF01499.cm fasta
cmsearch -Z 5400 -T 25.50 RF01500.cm fasta
cmsearch -Z 5400 -T 25.50 RF01501.cm fasta
cmsearch -Z 5400 -T 55.25 -g RF01502.cm fasta
cmsearch -Z 5400 -T 25.50 RF01503.cm fasta
cmsearch -Z 5400 -T 51.00 RF01504.cm fasta
cmsearch -Z 5400 -T 27.20 RF01505.cm fasta
cmsearch -Z 5400 -T 31.45 RF01506.cm fasta
cmsearch -Z 5400 -T 42.50 RF01507.cm fasta
cmsearch -Z 5400 -T 63.75 RF01508.cm fasta
cmsearch -Z 5400 -T 25.50 RF01509.cm fasta
cmsearch -Z 5400 -T 34.00 RF01510.cm fasta
cmsearch -Z 5400 -T 25.50 RF01511.cm fasta
cmsearch -Z 5400 -T 37.40 RF01512.cm fasta
cmsearch -Z 5400 -T 23.80 RF01513.cm fasta
cmsearch -Z 5400 -T 17.00 RF01514.cm fasta
cmsearch -Z 5400 -T 23.80 RF01515.cm fasta
cmsearch -Z 5400 -T 63.75 RF01516.cm fasta
cmsearch -Z 5400 -T 29.75 RF01517.cm fasta
cmsearch -Z 5400 -T 25.50 RF01518.cm fasta

Table S17 – continued from previous page

command line
cmsearch -Z 5400 -T 29.75 RF01519.cm fasta
cmsearch -Z 5400 -T 29.75 RF01520.cm fasta
cmsearch -Z 5400 -T 34.00 RF01521.cm fasta
cmsearch -Z 5400 -T 34.00 RF01522.cm fasta
cmsearch -Z 5400 -T 42.50 RF01523.cm fasta
cmsearch -Z 5400 -T 42.50 RF01524.cm fasta
cmsearch -Z 5400 -T 29.75 RF01525.cm fasta
cmsearch -Z 5400 -T 51.00 RF01526.cm fasta
cmsearch -Z 5400 -T 15.30 RF01527.cm fasta
cmsearch -Z 5400 -T 17.00 RF01528.cm fasta
cmsearch -Z 5400 -T 42.50 RF01529.cm fasta
cmsearch -Z 5400 -T 25.50 RF01530.cm fasta
cmsearch -Z 5400 -T 34.00 RF01531.cm fasta
cmsearch -Z 5400 -T 38.25 RF01532.cm fasta
cmsearch -Z 5400 -T 38.25 RF01533.cm fasta
cmsearch -Z 5400 -T 42.50 RF01534.cm fasta
cmsearch -Z 5400 -T 34.00 RF01535.cm fasta
cmsearch -Z 5400 -T 21.25 RF01536.cm fasta
cmsearch -Z 5400 -T 13.60 RF01537.cm fasta
cmsearch -Z 5400 -T 34.00 RF01538.cm fasta
cmsearch -Z 5400 -T 13.60 RF01539.cm fasta
cmsearch -Z 5400 -T 21.25 RF01540.cm fasta
cmsearch -Z 5400 -T 17.00 RF01541.cm fasta
cmsearch -Z 5400 -T 51.00 RF01542.cm fasta
cmsearch -Z 5400 -T 35.70 RF01543.cm fasta
cmsearch -Z 5400 -T 21.25 RF01544.cm fasta
cmsearch -Z 5400 -T 51.00 RF01545.cm fasta
cmsearch -Z 5400 -T 34.00 RF01546.cm fasta
cmsearch -Z 5400 -T 25.50 RF01547.cm fasta
cmsearch -Z 5400 -T 51.00 RF01548.cm fasta
cmsearch -Z 5400 -T 25.50 RF01549.cm fasta
cmsearch -Z 5400 -T 38.25 RF01550.cm fasta
cmsearch -Z 5400 -T 51.00 RF01551.cm fasta
cmsearch -Z 5400 -T 29.75 RF01552.cm fasta
cmsearch -Z 5400 -T 25.50 RF01553.cm fasta
cmsearch -Z 5400 -T 38.25 RF01554.cm fasta
cmsearch -Z 5400 -T 38.25 RF01555.cm fasta
cmsearch -Z 5400 -T 42.50 RF01556.cm fasta
cmsearch -Z 5400 -T 29.75 RF01557.cm fasta
cmsearch -Z 5400 -T 42.50 RF01558.cm fasta
cmsearch -Z 5400 -T 68.00 RF01559.cm fasta
cmsearch -Z 5400 -T 68.00 RF01560.cm fasta
cmsearch -Z 5400 -T 59.50 RF01561.cm fasta
cmsearch -Z 5400 -T 59.50 RF01562.cm fasta
cmsearch -Z 5400 -T 68.00 RF01563.cm fasta
cmsearch -Z 5400 -T 68.00 RF01564.cm fasta
cmsearch -Z 5400 -T 76.50 RF01565.cm fasta
cmsearch -Z 5400 -T 68.00 RF01566.cm fasta
cmsearch -Z 5400 -T 24.65 RF01567.cm fasta

Table S17 – continued from previous page

command line
cmsearch -Z 5400 -T 85.00 RF01568.cm fasta
cmsearch -Z 5400 -T 76.50 RF01569.cm fasta
cmsearch -Z 5400 -T 127.50 RF01570.cm fasta
cmsearch -Z 5400 -T 51.00 RF01571.cm fasta
cmsearch -Z 5400 -T 85.00 RF01572.cm fasta
cmsearch -Z 5400 -T 76.50 RF01573.cm fasta
cmsearch -Z 5400 -T 18.547000 RF01574.cm fasta
cmsearch -Z 5400 -T 85.00 RF01575.cm fasta
cmsearch -Z 5400 -T 68.00 RF01576.cm fasta
cmsearch -Z 5400 -T 46.75 RF01577.cm fasta
cmsearch -Z 5400 -T 19.55 RF01578.cm fasta
cmsearch -Z 5400 -T 76.50 RF01579.cm fasta
cmsearch -Z 5400 -T 68.00 RF01580.cm fasta
cmsearch -Z 5400 -T 29.75 RF01581.cm fasta
cmsearch -Z 5400 -T 42.50 RF01582.cm fasta
cmsearch -Z 5400 -T 38.25 RF01583.cm fasta
cmsearch -Z 5400 -T 30.60 RF01584.cm fasta
cmsearch -Z 5400 -T 25.50 RF01585.cm fasta
cmsearch -Z 5400 -T 42.50 RF01586.cm fasta
cmsearch -Z 5400 -T 42.50 RF01587.cm fasta
cmsearch -Z 5400 -T 51.00 RF01588.cm fasta
cmsearch -Z 5400 -T 17.00 RF01589.cm fasta
cmsearch -Z 5400 -T 20.40 RF01590.cm fasta
cmsearch -Z 5400 -T 27.20 RF01591.cm fasta
cmsearch -Z 5400 -T 34.00 RF01592.cm fasta
cmsearch -Z 5400 -T 30.60 RF01593.cm fasta
cmsearch -Z 5400 -T 34.00 RF01594.cm fasta
cmsearch -Z 5400 -T 51.00 RF01595.cm fasta
cmsearch -Z 5400 -T 21.25 RF01596.cm fasta
cmsearch -Z 5400 -T 24.65 RF01597.cm fasta
cmsearch -Z 5400 -T 34.00 RF01598.cm fasta
cmsearch -Z 5400 -T 25.50 RF01599.cm fasta
cmsearch -Z 5400 -T 42.50 RF01600.cm fasta
cmsearch -Z 5400 -T 29.75 RF01601.cm fasta
cmsearch -Z 5400 -T 34.00 RF01602.cm fasta
cmsearch -Z 5400 -T 32.30 RF01603.cm fasta
cmsearch -Z 5400 -T 53.55 RF01604.cm fasta
cmsearch -Z 5400 -T 34.00 RF01605.cm fasta
cmsearch -Z 5400 -T 34.00 RF01606.cm fasta
cmsearch -Z 5400 -T 68.00 RF01607.cm fasta
cmsearch -Z 5400 -T 59.50 RF01608.cm fasta
cmsearch -Z 5400 -T 34.00 RF01609.cm fasta
cmsearch -Z 5400 -T 55.25 RF01610.cm fasta
cmsearch -Z 5400 -T 34.00 RF01611.cm fasta
cmsearch -Z 5400 -T 21.25 RF01612.cm fasta
cmsearch -Z 5400 -T 34.00 RF01613.cm fasta
cmsearch -Z 5400 -T 42.50 RF01614.cm fasta
cmsearch -Z 5400 -T 35.70 RF01615.cm fasta
cmsearch -Z 5400 -T 29.75 RF01617.cm fasta

Table S17 – continued from previous page

command line
cmsearch -Z 5400 -T 29.75 RF01618.cm fasta
cmsearch -Z 5400 -T 29.75 RF01619.cm fasta
cmsearch -Z 5400 -T 68.00 RF01620.cm fasta
cmsearch -Z 5400 -T 85.00 RF01621.cm fasta
cmsearch -Z 5400 -T 55.25 RF01622.cm fasta
cmsearch -Z 5400 -T 34.00 RF01623.cm fasta
cmsearch -Z 5400 -T 21.25 RF01624.cm fasta
cmsearch -Z 5400 -T 59.50 RF01625.cm fasta
cmsearch -Z 5400 -T 55.25 RF01626.cm fasta
cmsearch -Z 5400 -T 29.75 RF01627.cm fasta
cmsearch -Z 5400 -T 20.40 RF01628.cm fasta
cmsearch -Z 5400 -T 76.50 RF01629.cm fasta
cmsearch -Z 5400 -T 38.25 RF01630.cm fasta
cmsearch -Z 5400 -T 25.50 RF01631.cm fasta
cmsearch -Z 5400 -T 29.75 RF01632.cm fasta
cmsearch -Z 5400 -T 42.50 RF01633.cm fasta
cmsearch -Z 5400 -T 59.50 RF01634.cm fasta
cmsearch -Z 5400 -T 51.00 RF01635.cm fasta
cmsearch -Z 5400 -T 42.50 RF01636.cm fasta
cmsearch -Z 5400 -T 17.00 RF01637.cm fasta
cmsearch -Z 5400 -T 59.50 RF01638.cm fasta
cmsearch -Z 5400 -T 55.25 RF01639.cm fasta
cmsearch -Z 5400 -T 63.75 RF01640.cm fasta
cmsearch -Z 5400 -T 51.00 RF01641.cm fasta
cmsearch -Z 5400 -T 63.75 RF01642.cm fasta
cmsearch -Z 5400 -T 21.25 RF01643.cm fasta
cmsearch -Z 5400 -T 42.50 RF01644.cm fasta
cmsearch -Z 5400 -T 68.00 RF01645.cm fasta
cmsearch -Z 5400 -T 55.25 RF01646.cm fasta
cmsearch -Z 5400 -T 34.00 RF01647.cm fasta
cmsearch -Z 5400 -T 51.00 RF01648.cm fasta
cmsearch -Z 5400 -T 34.00 RF01649.cm fasta
cmsearch -Z 5400 -T 17.00 RF01650.cm fasta
cmsearch -Z 5400 -T 17.00 RF01651.cm fasta
cmsearch -Z 5400 -T 42.50 RF01652.cm fasta
cmsearch -Z 5400 -T 68.00 RF01653.cm fasta
cmsearch -Z 5400 -T 68.00 RF01654.cm fasta
cmsearch -Z 5400 -T 25.50 RF01655.cm fasta
cmsearch -Z 5400 -T 17.00 RF01656.cm fasta
cmsearch -Z 5400 -T 18.70 RF01657.cm fasta
cmsearch -Z 5400 -T 59.50 RF01658.cm fasta
cmsearch -Z 5400 -T 21.25 RF01659.cm fasta
cmsearch -Z 5400 -T 42.50 RF01660.cm fasta
cmsearch -Z 5400 -T 25.50 RF01661.cm fasta
cmsearch -Z 5400 -T 51.00 RF01662.cm fasta
cmsearch -Z 5400 -T 17.00 RF01663.cm fasta
cmsearch -Z 5400 -T 59.50 RF01664.cm fasta
cmsearch -Z 5400 -T 59.50 RF01665.cm fasta
cmsearch -Z 5400 -T 16.15 RF01666.cm fasta

Table S17 – continued from previous page

command line
cmsearch -Z 5400 -T 25.50 RF01667.cm fasta
cmsearch -Z 5400 -T 16.15 RF01668.cm fasta
cmsearch -Z 5400 -T 25.50 RF01669.cm fasta
cmsearch -Z 5400 -T 17.85 RF01670.cm fasta
cmsearch -Z 5400 -T 23.80 RF01671.cm fasta
cmsearch -Z 5400 -T 68.00 RF01672.cm fasta
cmsearch -Z 5400 -T 51.00 RF01673.cm fasta
cmsearch -Z 5400 -T 42.50 RF01674.cm fasta
cmsearch -Z 5400 -T 42.50 RF01675.cm fasta
cmsearch -Z 5400 -T 21.25 RF01676.cm fasta
cmsearch -Z 5400 -T 34.00 RF01677.cm fasta
cmsearch -Z 5400 -T 42.50 RF01678.cm fasta
cmsearch -Z 5400 -T 42.50 RF01679.cm fasta
cmsearch -Z 5400 -T 51.00 RF01680.cm fasta
cmsearch -Z 5400 -T 10.20 RF01681.cm fasta
cmsearch -Z 5400 -T 51.00 RF01682.cm fasta
cmsearch -Z 5400 -T 19.55 RF01683.cm fasta
cmsearch -Z 5400 -T 27.20 RF01684.cm fasta
cmsearch -Z 5400 -T 18.70 RF01685.cm fasta
cmsearch -Z 5400 -T 42.50 RF01686.cm fasta
cmsearch -Z 5400 -T 27.20 RF01687.cm fasta
cmsearch -Z 5400 -T 19.55 RF01688.cm fasta
cmsearch -Z 5400 -T 29.75 RF01689.cm fasta
cmsearch -Z 5400 -T 34.00 RF01690.cm fasta
cmsearch -Z 5400 -T 15.30 RF01691.cm fasta
cmsearch -Z 5400 -T 22.95 RF01692.cm fasta
cmsearch -Z 5400 -T 27.20 RF01693.cm fasta
cmsearch -Z 5400 -T 17.85 RF01694.cm fasta
cmsearch -Z 5400 -T 17.85 RF01695.cm fasta
cmsearch -Z 5400 -T 29.75 RF01696.cm fasta
cmsearch -Z 5400 -T 34.00 RF01697.cm fasta
cmsearch -Z 5400 -T 68.00 RF01698.cm fasta
cmsearch -Z 5400 -T 22.95 RF01699.cm fasta
cmsearch -Z 5400 -T 25.50 RF01700.cm fasta
cmsearch -Z 5400 -T 28.985000 RF01701.cm fasta
cmsearch -Z 5400 -T 17.85 RF01702.cm fasta
cmsearch -Z 5400 -T 127.50 RF01703.cm fasta
cmsearch -Z 5400 -T 16.15 RF01704.cm fasta
cmsearch -Z 5400 -T 15.30 RF01705.cm fasta
cmsearch -Z 5400 -T 17.00 RF01706.cm fasta
cmsearch -Z 5400 -T 26.35 RF01707.cm fasta
cmsearch -Z 5400 -T 21.25 RF01708.cm fasta
cmsearch -Z 5400 -T 24.65 RF01709.cm fasta
cmsearch -Z 5400 -T 42.50 RF01710.cm fasta
cmsearch -Z 5400 -T 38.25 RF01711.cm fasta
cmsearch -Z 5400 -T 21.25 RF01712.cm fasta
cmsearch -Z 5400 -T 29.75 RF01713.cm fasta
cmsearch -Z 5400 -T 63.75 RF01714.cm fasta
cmsearch -Z 5400 -T 127.50 RF01715.cm fasta

Table S17 – continued from previous page

command line
cmsearch -Z 5400 -T 34.00 RF01716.cm fasta
cmsearch -Z 5400 -T 19.55 RF01717.cm fasta
cmsearch -Z 5400 -T 22.95 RF01718.cm fasta
cmsearch -Z 5400 -T 51.00 RF01719.cm fasta
cmsearch -Z 5400 -T 22.95 RF01720.cm fasta
cmsearch -Z 5400 -T 25.50 RF01721.cm fasta
cmsearch -Z 5400 -T 25.50 RF01722.cm fasta
cmsearch -Z 5400 -T 29.75 RF01723.cm fasta
cmsearch -Z 5400 -T 29.75 RF01724.cm fasta
cmsearch -Z 5400 -T 17.85 RF01725.cm fasta
cmsearch -Z 5400 -T 25.50 RF01726.cm fasta
cmsearch -Z 5400 -T 25.50 RF01727.cm fasta
cmsearch -Z 5400 -T 21.25 RF01728.cm fasta
cmsearch -Z 5400 -T 21.25 RF01729.cm fasta
cmsearch -Z 5400 -T 16.15 RF01730.cm fasta
cmsearch -Z 5400 -T 17.00 RF01731.cm fasta
cmsearch -Z 5400 -T 42.50 RF01732.cm fasta
cmsearch -Z 5400 -T 42.50 RF01733.cm fasta
cmsearch -Z 5400 -T 19.55 RF01734.cm fasta
cmsearch -Z 5400 -T 21.25 RF01735.cm fasta
cmsearch -Z 5400 -T 46.75 RF01736.cm fasta
cmsearch -Z 5400 -T 34.00 RF01737.cm fasta
cmsearch -Z 5400 -T 17.00 RF01738.cm fasta
cmsearch -Z 5400 -T 18.70 RF01739.cm fasta
cmsearch -Z 5400 -T 38.25 RF01740.cm fasta
cmsearch -Z 5400 -T 38.25 RF01741.cm fasta
cmsearch -Z 5400 -T 59.50 RF01742.cm fasta
cmsearch -Z 5400 -T 85.00 RF01743.cm fasta
cmsearch -Z 5400 -T 55.25 RF01744.cm fasta
cmsearch -Z 5400 -T 20.40 RF01745.cm fasta
cmsearch -Z 5400 -T 17.00 RF01746.cm fasta
cmsearch -Z 5400 -T 17.00 RF01747.cm fasta
cmsearch -Z 5400 -T 24.65 RF01748.cm fasta
cmsearch -Z 5400 -T 17.00 RF01749.cm fasta
cmsearch -Z 5400 -T 18.70 RF01750.cm fasta
cmsearch -Z 5400 -T 22.95 RF01751.cm fasta
cmsearch -Z 5400 -T 17.00 RF01752.cm fasta
cmsearch -Z 5400 -T 24.65 RF01753.cm fasta
cmsearch -Z 5400 -T 17.85 RF01754.cm fasta
cmsearch -Z 5400 -T 25.50 RF01755.cm fasta
cmsearch -Z 5400 -T 17.85 RF01756.cm fasta
cmsearch -Z 5400 -T 25.50 RF01757.cm fasta
cmsearch -Z 5400 -T 59.50 RF01758.cm fasta
cmsearch -Z 5400 -T 42.50 RF01759.cm fasta
cmsearch -Z 5400 -T 17.00 RF01760.cm fasta
cmsearch -Z 5400 -T 19.55 RF01761.cm fasta
cmsearch -Z 5400 -T 25.50 RF01762.cm fasta
cmsearch -Z 5400 -T 25.50 RF01763.cm fasta
cmsearch -Z 5400 -T 23.80 RF01764.cm fasta

Table S17 – continued from previous page

command line
cmsearch -Z 5400 -T 25.50 RF01765.cm fasta
cmsearch -Z 5400 -T 38.25 RF01766.cm fasta
cmsearch -Z 5400 -T 25.50 RF01767.cm fasta
cmsearch -Z 5400 -T 22.95 RF01768.cm fasta
cmsearch -Z 5400 -T 34.00 RF01769.cm fasta
cmsearch -Z 5400 -T 39.95 RF01770.cm fasta
cmsearch -Z 5400 -T 25.50 RF01771.cm fasta
cmsearch -Z 5400 -T 38.25 RF01772.cm fasta
cmsearch -Z 5400 -T 34.00 RF01773.cm fasta
cmsearch -Z 5400 -T 34.00 RF01774.cm fasta
cmsearch -Z 5400 -T 72.25 RF01775.cm fasta
cmsearch -Z 5400 -T 29.75 RF01776.cm fasta
cmsearch -Z 5400 -T 51.00 RF01778.cm fasta
cmsearch -Z 5400 -T 42.50 RF01779.cm fasta
cmsearch -Z 5400 -T 85.00 RF01780.cm fasta
cmsearch -Z 5400 -T 34.00 RF01781.cm fasta
cmsearch -Z 5400 -T 44.20 RF01782.cm fasta
cmsearch -Z 5400 -T 42.50 RF01783.cm fasta
cmsearch -Z 5400 -T 29.75 RF01784.cm fasta
cmsearch -Z 5400 -T 27.20 RF01785.cm fasta
cmsearch -Z 5400 -T 38.25 RF01786.cm fasta
cmsearch -Z 5400 -T 42.50 RF01787.cm fasta
cmsearch -Z 5400 -T 24.65 RF01788.cm fasta
cmsearch -Z 5400 -T 25.50 RF01789.cm fasta
cmsearch -Z 5400 -T 40.80 RF01790.cm fasta
cmsearch -Z 5400 -T 34.00 RF01791.cm fasta
cmsearch -Z 5400 -T 31.45 RF01792.cm fasta
cmsearch -Z 5400 -T 29.75 RF01793.cm fasta
cmsearch -Z 5400 -T 33.15 RF01794.cm fasta
cmsearch -Z 5400 -T 68.00 RF01795.cm fasta
cmsearch -Z 5400 -T 51.00 RF01796.cm fasta
cmsearch -Z 5400 -T 25.50 RF01797.cm fasta
cmsearch -Z 5400 -T 42.50 RF01798.cm fasta
cmsearch -Z 5400 -T 59.50 RF01800.cm fasta
cmsearch -Z 5400 -T 34.00 RF01802.cm fasta
cmsearch -Z 5400 -T 46.75 RF01803.cm fasta
cmsearch -Z 5400 -T 51.00 RF01804.cm fasta
cmsearch -Z 5400 -T 127.50 RF01807.cm fasta
cmsearch -Z 5400 -T 42.50 RF01808.cm fasta
cmsearch -Z 5400 -T 42.50 RF01809.cm fasta
cmsearch -Z 5400 -T 28.90 RF01810.cm fasta
cmsearch -Z 5400 -T 25.50 RF01811.cm fasta
cmsearch -Z 5400 -T 85.00 RF01812.cm fasta
cmsearch -Z 5400 -T 25.50 RF01813.cm fasta
cmsearch -Z 5400 -T 29.75 RF01814.cm fasta
cmsearch -Z 5400 -T 29.75 RF01815.cm fasta
cmsearch -Z 5400 -T 42.50 RF01816.cm fasta
cmsearch -Z 5400 -T 25.50 RF01817.cm fasta
cmsearch -Z 5400 -T 85.00 RF01818.cm fasta

Table S17 – continued from previous page

command line
cmsearch -Z 5400 -T 42.50 RF01819.cm fasta
cmsearch -Z 5400 -T 51.00 RF01820.cm fasta
cmsearch -Z 5400 -T 42.50 RF01821.cm fasta
cmsearch -Z 5400 -T 85.00 RF01822.cm fasta
cmsearch -Z 5400 -T 29.75 RF01823.cm fasta
cmsearch -Z 5400 -T 42.50 RF01824.cm fasta
cmsearch -Z 5400 -T 42.50 RF01825.cm fasta
cmsearch -Z 5400 -T 25.50 RF01826.cm fasta
cmsearch -Z 5400 -T 25.50 RF01827.cm fasta
cmsearch -Z 5400 -T 85.00 RF01828.cm fasta
cmsearch -Z 5400 -T 59.50 RF01829.cm fasta
cmsearch -Z 5400 -T 29.75 RF01830.cm fasta
cmsearch -Z 5400 -T 25.50 RF01831.cm fasta
cmsearch -Z 5400 -T 34.00 RF01832.cm fasta
cmsearch -Z 5400 -T 46.75 RF01833.cm fasta
cmsearch -Z 5400 -T 32.30 RF01834.cm fasta
cmsearch -Z 5400 -T 30.60 RF01835.cm fasta
cmsearch -Z 5400 -T 30.60 RF01836.cm fasta
cmsearch -Z 5400 -T 38.25 RF01837.cm fasta
cmsearch -Z 5400 -T 32.30 RF01838.cm fasta
cmsearch -Z 5400 -T 68.00 RF01839.cm fasta
cmsearch -Z 5400 -T 34.00 RF01840.cm fasta
cmsearch -Z 5400 -T 50.15 RF01841.cm fasta
cmsearch -Z 5400 -T 63.75 RF01842.cm fasta
cmsearch -Z 5400 -T 28.05 RF01843.cm fasta
cmsearch -Z 5400 -T 25.50 RF01844.cm fasta
cmsearch -Z 5400 -T 38.25 RF01845.cm fasta
cmsearch -Z 5400 -T 76.50 RF01846.cm fasta
cmsearch -Z 5400 -T 62.90 RF01847.cm fasta
cmsearch -Z 5400 -T 46.75 RF01848.cm fasta
cmsearch -Z 5400 -T 42.50 -g RF01849.cm fasta
cmsearch -Z 5400 -T 85.00 RF01850.cm fasta
cmsearch -Z 5400 -T 55.25 RF01851.cm fasta
cmsearch -Z 5400 -T 45.05 RF01852.cm fasta
cmsearch -Z 5400 -T 20.40 RF01853.cm fasta
cmsearch -Z 5400 -T 42.50 RF01854.cm fasta
cmsearch -Z 5400 -T 80.75 -g RF01855.cm fasta
cmsearch -Z 5400 -T 28.90 -g RF01856.cm fasta
cmsearch -Z 5400 -T 73.95 -g RF01857.cm fasta
cmsearch -Z 5400 -T 85.00 RF01858.cm fasta
cmsearch -Z 5400 -T 35.70 RF01859.cm fasta
cmsearch -Z 5400 -T 27.20 RF01860.cm fasta
cmsearch -Z 5400 -T 21.25 RF01861.cm fasta
cmsearch -Z 5400 -T 34.00 RF01862.cm fasta
cmsearch -Z 5400 -T 51.00 RF01863.cm fasta
cmsearch -Z 5400 -T 28.90 RF01864.cm fasta
cmsearch -Z 5400 -T 42.50 RF01865.cm fasta
cmsearch -Z 5400 -T 38.25 RF01866.cm fasta
cmsearch -Z 5400 -T 25.50 RF01867.cm fasta

Table S17 – continued from previous page

command line
cmsearch -Z 5400 -T 34.00 RF01868.cm fasta
cmsearch -Z 5400 -T 34.00 RF01869.cm fasta
cmsearch -Z 5400 -T 34.00 RF01870.cm fasta
cmsearch -Z 5400 -T 34.00 RF01871.cm fasta
cmsearch -Z 5400 -T 26.35 RF01872.cm fasta
cmsearch -Z 5400 -T 30.60 RF01873.cm fasta
cmsearch -Z 5400 -T 42.50 RF01874.cm fasta
cmsearch -Z 5400 -T 42.50 RF01875.cm fasta
cmsearch -Z 5400 -T 51.00 RF01876.cm fasta
cmsearch -Z 5400 -T 25.50 RF01877.cm fasta
cmsearch -Z 5400 -T 34.00 RF01878.cm fasta
cmsearch -Z 5400 -T 55.25 RF01879.cm fasta
cmsearch -Z 5400 -T 28.05 RF01880.cm fasta
cmsearch -Z 5400 -T 85.00 RF01881.cm fasta
cmsearch -Z 5400 -T 46.75 RF01882.cm fasta
cmsearch -Z 5400 -T 34.00 RF01883.cm fasta
cmsearch -Z 5400 -T 46.75 RF01884.cm fasta
cmsearch -Z 5400 -T 68.00 RF01885.cm fasta
cmsearch -Z 5400 -T 34.00 RF01886.cm fasta
cmsearch -Z 5400 -T 51.00 RF01887.cm fasta
cmsearch -Z 5400 -T 68.00 RF01888.cm fasta
cmsearch -Z 5400 -T 127.50 RF01889.cm fasta
cmsearch -Z 5400 -T 51.00 RF01890.cm fasta
cmsearch -Z 5400 -T 55.25 RF01891.cm fasta
cmsearch -Z 5400 -T 85.00 RF01892.cm fasta
cmsearch -Z 5400 -T 85.00 RF01893.cm fasta
cmsearch -Z 5400 -T 46.75 RF01894.cm fasta
cmsearch -Z 5400 -T 48.45 RF01895.cm fasta
cmsearch -Z 5400 -T 62.05 RF01896.cm fasta
cmsearch -Z 5400 -T 59.50 RF01897.cm fasta
cmsearch -Z 5400 -T 47.60 RF01898.cm fasta
cmsearch -Z 5400 -T 39.95 RF01899.cm fasta
cmsearch -Z 5400 -T 63.75 RF01900.cm fasta
cmsearch -Z 5400 -T 81.60 RF01901.cm fasta
cmsearch -Z 5400 -T 34.00 RF01902.cm fasta
cmsearch -Z 5400 -T 42.50 RF01903.cm fasta
cmsearch -Z 5400 -T 36.55 RF01904.cm fasta
cmsearch -Z 5400 -T 51.00 RF01905.cm fasta
cmsearch -Z 5400 -T 68.00 RF01906.cm fasta
cmsearch -Z 5400 -T 29.75 RF01907.cm fasta
cmsearch -Z 5400 -T 29.75 RF01908.cm fasta
cmsearch -Z 5400 -T 63.75 RF01909.cm fasta
cmsearch -Z 5400 -T 66.30 RF01910.cm fasta
cmsearch -Z 5400 -T 68.00 RF01911.cm fasta
cmsearch -Z 5400 -T 80.75 RF01912.cm fasta
cmsearch -Z 5400 -T 85.00 RF01913.cm fasta
cmsearch -Z 5400 -T 44.20 RF01914.cm fasta
cmsearch -Z 5400 -T 42.50 RF01915.cm fasta
cmsearch -Z 5400 -T 62.05 RF01916.cm fasta

Table S17 – continued from previous page

command line
cmsearch -Z 5400 -T 51.00 RF01917.cm fasta
cmsearch -Z 5400 -T 95.20 RF01918.cm fasta
cmsearch -Z 5400 -T 34.85 RF01919.cm fasta
cmsearch -Z 5400 -T 65.45 RF01920.cm fasta
cmsearch -Z 5400 -T 77.35 RF01921.cm fasta
cmsearch -Z 5400 -T 38.25 RF01922.cm fasta
cmsearch -Z 5400 -T 54.40 RF01923.cm fasta
cmsearch -Z 5400 -T 63.75 RF01924.cm fasta
cmsearch -Z 5400 -T 72.25 RF01925.cm fasta
cmsearch -Z 5400 -T 56.10 RF01926.cm fasta
cmsearch -Z 5400 -T 77.35 RF01927.cm fasta
cmsearch -Z 5400 -T 29.75 RF01928.cm fasta
cmsearch -Z 5400 -T 63.75 RF01929.cm fasta
cmsearch -Z 5400 -T 68.00 RF01930.cm fasta
cmsearch -Z 5400 -T 59.50 RF01931.cm fasta
cmsearch -Z 5400 -T 34.00 RF01932.cm fasta
cmsearch -Z 5400 -T 42.50 RF01933.cm fasta
cmsearch -Z 5400 -T 42.50 RF01934.cm fasta
cmsearch -Z 5400 -T 51.00 RF01935.cm fasta
cmsearch -Z 5400 -T 61.20 RF01936.cm fasta
cmsearch -Z 5400 -T 79.721500 RF01937.cm fasta
cmsearch -Z 5400 -T 60.35 RF01938.cm fasta
cmsearch -Z 5400 -T 57.80 RF01939.cm fasta
cmsearch -Z 5400 -T 59.50 RF01940.cm fasta
cmsearch -Z 5400 -T 68.00 RF01941.cm fasta
cmsearch -Z 5400 -T 42.50 RF01942.cm fasta
cmsearch -Z 5400 -T 49.30 RF01943.cm fasta
cmsearch -Z 5400 -T 51.85 RF01944.cm fasta
cmsearch -Z 5400 -T 38.25 RF01945.cm fasta
cmsearch -Z 5400 -T 63.75 RF01946.cm fasta
cmsearch -Z 5400 -T 38.25 RF01947.cm fasta
cmsearch -Z 5400 -T 34.00 RF01948.cm fasta
cmsearch -Z 5400 -T 42.50 RF01949.cm fasta
cmsearch -Z 5400 -T 102.00 RF01950.cm fasta
cmsearch -Z 5400 -T 23.80 RF01951.cm fasta
cmsearch -Z 5400 -T 38.25 RF01952.cm fasta
cmsearch -Z 5400 -T 55.25 RF01953.cm fasta
cmsearch -Z 5400 -T 24.65 RF01954.cm fasta
cmsearch -Z 5400 -T 29.75 RF01955.cm fasta
cmsearch -Z 5400 -T 25.50 RF01956.cm fasta
cmsearch -Z 5400 -T 29.75 RF01957.cm fasta
cmsearch -Z 5400 -T 20.40 RF01958.cm fasta
cmsearch -Z 5400 -T 559.30 -g RF01959.cm fasta
cmsearch -Z 5400 -T 425.00 -g RF01960.cm fasta
cmsearch -Z 5400 -T 29.75 RF01961.cm fasta
cmsearch -Z 5400 -T 34.00 RF01962.cm fasta
cmsearch -Z 5400 -T 34.00 RF01963.cm fasta
cmsearch -Z 5400 -T 42.50 RF01964.cm fasta
cmsearch -Z 5400 -T 38.25 RF01965.cm fasta

Table S17 – continued from previous page

command line
cmsearch -Z 5400 -T 59.50 RF01966.cm fasta
cmsearch -Z 5400 -T 34.00 RF01967.cm fasta
cmsearch -Z 5400 -T 85.00 RF01968.cm fasta
cmsearch -Z 5400 -T 34.00 RF01969.cm fasta
cmsearch -Z 5400 -T 49.30 RF01970.cm fasta
cmsearch -Z 5400 -T 29.75 RF01971.cm fasta
cmsearch -Z 5400 -T 29.75 RF01972.cm fasta
cmsearch -Z 5400 -T 42.50 RF01973.cm fasta
cmsearch -Z 5400 -T 25.50 RF01974.cm fasta
cmsearch -Z 5400 -T 31.45 RF01975.cm fasta
cmsearch -Z 5400 -T 31.45 RF01976.cm fasta
cmsearch -Z 5400 -T 31.45 RF01977.cm fasta
cmsearch -Z 5400 -T 29.75 RF01978.cm fasta
cmsearch -Z 5400 -T 46.75 RF01979.cm fasta
cmsearch -Z 5400 -T 63.75 RF01980.cm fasta
cmsearch -Z 5400 -T 85.00 RF01981.cm fasta
cmsearch -Z 5400 -T 42.50 RF01982.cm fasta
cmsearch -Z 5400 -T 30.60 RF01983.cm fasta
cmsearch -Z 5400 -T 29.75 RF01984.cm fasta
cmsearch -Z 5400 -T 42.50 RF01985.cm fasta
cmsearch -Z 5400 -T 25.50 RF01986.cm fasta
cmsearch -Z 5400 -T 42.50 RF01987.cm fasta
cmsearch -Z 5400 -T 29.75 RF01988.cm fasta
cmsearch -Z 5400 -T 27.20 RF01989.cm fasta
cmsearch -Z 5400 -T 27.20 RF01990.cm fasta
cmsearch -Z 5400 -T 42.50 RF01991.cm fasta
cmsearch -Z 5400 -T 85.00 RF01992.cm fasta
cmsearch -Z 5400 -T 45.90 RF01996.cm fasta
cmsearch -Z 5400 -T 60.35 RF01997.cm fasta
cmsearch -Z 5400 -T 25.33 -g RF01998.cm fasta
cmsearch -Z 5400 -T 30.515000 -g RF01999.cm fasta
cmsearch -Z 5400 -T 28.05 RF02000.cm fasta
cmsearch -Z 5400 -T 33.15 -g RF02001.cm fasta
cmsearch -Z 5400 -T 30.60 RF02002.cm fasta
cmsearch -Z 5400 -T 44.20 -g RF02003.cm fasta
cmsearch -Z 5400 -T 36.55 -g RF02004.cm fasta
cmsearch -Z 5400 -T 41.65 -g RF02005.cm fasta
cmsearch -Z 5400 -T 96.90 RF02006.cm fasta
cmsearch -Z 5400 -T 68.00 RF02007.cm fasta
cmsearch -Z 5400 -T 25.50 RF02008.cm fasta
cmsearch -Z 5400 -T 75.65 RF02009.cm fasta
cmsearch -Z 5400 -T 33.15 RF02010.cm fasta
cmsearch -Z 5400 -T 68.00 RF02011.cm fasta
cmsearch -Z 5400 -T 28.90 -g RF02012.cm fasta
cmsearch -Z 5400 -T 78.20 RF02013.cm fasta
cmsearch -Z 5400 -T 29.75 RF02014.cm fasta
cmsearch -Z 5400 -T 36.55 RF02015.cm fasta
cmsearch -Z 5400 -T 85.00 RF02016.cm fasta
cmsearch -Z 5400 -T 70.55 RF02017.cm fasta

Table S17 – continued from previous page

command line
cmsearch -Z 5400 -T 28.90 RF02018.cm fasta
cmsearch -Z 5400 -T 46.75 RF02019.cm fasta
cmsearch -Z 5400 -T 68.85 RF02020.cm fasta
cmsearch -Z 5400 -T 80.75 RF02021.cm fasta
cmsearch -Z 5400 -T 47.60 RF02022.cm fasta
cmsearch -Z 5400 -T 29.75 RF02023.cm fasta
cmsearch -Z 5400 -T 46.75 RF02024.cm fasta
cmsearch -Z 5400 -T 31.45 RF02025.cm fasta
cmsearch -Z 5400 -T 55.25 RF02026.cm fasta
cmsearch -Z 5400 -T 28.05 RF02027.cm fasta
cmsearch -Z 5400 -T 74.80 RF02028.cm fasta
cmsearch -Z 5400 -T 38.25 RF02029.cm fasta
cmsearch -Z 5400 -T 39.10 RF02030.cm fasta
cmsearch -Z 5400 -T 34.85 RF02031.cm fasta

Table S18 - Command lines used for the alignment pipeline

<table>
<tr><th>cmdline</th></tr>
<tr><td>LASTZ for the alignment of target(pig) and query genomes. sts.2bit contains a single pig genome sequence chopped if necessary to be at most 80,010,000 nt long. query.2bit contains one or more query genome sequences, the total sequence length is at most 80,000,000 nt.LASTZ is followed by axtChain which chains the gapped alignments.</td></tr>
<tr><td>lastz -format=lav -masking=50 -inner=2000 sts.2bit query.2bit > out.lav</td></tr>
<tr><td>lastz -format=lav -masking=50 -inner=2000 -ydrop=3400 -gappedthresh=6000 -hspthresh=2200 -scores=HoxD55.q sts.2bit query.2bit > out.lav</td></tr>
<tr><td>lavToPsl out.lav out.psl</td></tr>
<tr><td>liftUp</td></tr>
<tr><td>axtChain -psl -linearGap=medium -minScore=3000 ts.psl ttwobit.2bit qtwobit.2bit axt.chain</td></tr>
<tr><td>axtChain -psl -linearGap=loose -minScore=5000 -scoreScheme=HoxD55.q.axt ts.psl ttwobit.2bit qtwobit.2bit axt.chain</td></tr>
<tr><td>chainAntiRepeat ttwobit.2bit qtwobit.2bit axt.chain 0.fa.2bit.chain</td></tr>
<tr><td>chainMergeSort *.chain > chain</td></tr>
<tr><td>Filtering of the alignments from multiple coverage to single coverage of the pig genome</td></tr>
<tr><td>chainPreNet chain target.sizes query.sizes prenet.chain</td></tr>
<tr><td>chainNet prenet.chain -minSpace=1 target.sizes query.sizes stdout /dev/null | netSyntenic stdin net</td></tr>
<tr><td>Formation of the liftover chain files</td></tr>
<tr><td>netChainSubset -verbose=0 net prenet.chain net.subset.chain</td></tr>
<tr><td>chainStitchId net.subset.chain liftover.chain</td></tr>
<tr><td>Formation of the syntenic net</td></tr>
<tr><td>netFilter -syn net > syntenic.net</td></tr>
<tr><td>Formation of the reciprocal best net</td></tr>
<tr><td>chainStitchId liftover.chain stdout | chainSwap stdin stdout | chainSort stdin qrbest.chain</td></tr>
<tr><td>chainPreNet qrbest.chain query.sizes target.sizes stdout | chainNet -minSpace=1 -minScore=0 stdin query.sizes target.sizes stdout /dev/null | netSyntenic stdin reverse.rbest.net</td></tr>
<tr><td>netChainSubset reverse.rbest.net qrbest.chain stdout | chainStitchId stdin reverse.rbest.chain</td></tr>
<tr><td>chainSwap reverse.rbest.chain stdout | chainSort stdin rbest.chain</td></tr>
<tr><td>chainPreNet rbest.chain target.sizes query.sizes stdout | chainNet -minSpace=1 -minScore=0 stdin target.sizes query.sizes stdout /dev/null | netSyntenic stdin rbest.net</td></tr>
<tr><td>Formation of the maf from net</td></tr>
<tr><td>netChainSubset -splitOnInsert net prenet.chain chain</td></tr>
<tr><td>chainToAxt chain target.2bit query.2bit net.axt</td></tr>
<tr><td>axtSort net.axt net.sorted.axt</td></tr>
<tr><td>axtToMaf net.sorted.axt target.sizes query.sizes net.maf -tPrefix=susScr102. -qPrefix=query.</td></tr>
<tr><td>Formation of the multiple alignments for the net based mafs</td></tr>
<tr><td>roast + R=30 M=1 T=. E=target '(((((((((susScr102 (bosTau5 turTru1)) (equCab2 (felCat4 canFam2))) eriEur1) ((hg19 tarSyr1) ((mm9 rn4) oryCun2))) ((loxAfr3 echTel1) (dasNov2 choHof1))) monDom5) ornAna1) galGal3) xenTro2) danRer7)' susScr102.*.sing.maf maf.out</td></tr>
</table>

Table S19 - `LASTZ` and `axtChain` options

`LASTZ` and `axtChain` parameters used for the pairwise alignments of pig and distantly/closely related species. These choices are largely based on the ones made for the human pairwise alignments by UCSC. The classification of species as distantly or closely related to pig can be seen in the main text Table 4.

	Distant	Close
blastz_m	50	50
blastz_y	3400	default
blastz_l	6000	default
blastz_k	2200	default
blastz_q	HoxD55	default
chain min. score	5000	3000
chain linear gap	loose	medium

Table S20 - Final high, medium and low confident annotation

The final annotation is a combination of the results of the homology pipeline at different levels and the cleaned up `miRDeep` results at high confidence level and the `miRDeep` results prior to cleaning at lower confidence levels. See main paper Table 2 for row labels and further explanation. Medium confident loci includes high confident loci. Likewise, low confident includes both medium and high.

	# high-confident loci	# medium-confident loci	# low-confident loci
cisreg-elements	139	163	176
lncRNA-loci	58	58	58
miRNA	369	572	1,673
putative-miRNA	155	175	175
ribozyme	8	8	9
rRNA	185	186	186
snoRNA	638	635	667
snRNA	1,030	1,066	1,147
tRNA	810	837	32,320
other	153	158	198
conflicts	11	19	38
sum	3,556	3,877	36,647

Table S21 - 100nt upstream of human PolIII sequences

100nt upstream of curated human sequences known to be transcribed by polIII. Using these sequences we created the position weight matrices for the PSE and TATA box promoter elements for polIII transcripts.

```
>ENSG00000202542|ENST00000365672|Y RNA [Source:RFAM;Acc:RF00019]|Y_RNA
GTTTTAATAAAGGCTCCATTACCTTAAATATAGAGGCAGAGCCAGGGTCTGTGTTCTTAGCAATGCTTCTGTGGTACAATTGTATTAAGAGTAATTGCAT
>ENSG00000202514|ENST00000365644|Y RNA [Source:RFAM;Acc:RF00019]|Y_RNA
TGAGAGCTGCTTAGGACAAAGAATTGAGATGAATTTAAGTTACAAATTGATACCATAGTTAAACAAATATTGTATAATCCCAGGCTTTAAAATAATTATT
>ENSG00000199584|ENST00000362714|Y RNA [Source:RFAM;Acc:RF00019]|Y_RNA
TACATCTCTACAGAAATGTTTTCTCTGAGCTTATGATGCTAAAGATTGACAAAATACACCTCCCCTATCACCATCCTGCCCAGTTAAAAAATTCCTGCTG
>ENSG00000199710|ENST00000362840|Y RNA [Source:RFAM;Acc:RF00019]|Y_RNA
CTCCTGGTGTGGTGGAGCATATTGTCGAGTAGCTTCTTAAGACACGATACTTGGAAGTAAGTTTGTTGAAATTTTTGTACGTTTGAAATATATTTATCCT
>ENSG00000271819|ENST00000607728|U6 spliceosomal RNA [Source:RFAM;Acc:RF00026]|U6
TAACGGTTTTGGATGTATAGCCCCTTCTGACTCCTGGAATCCTTTATTTCCACAGCACTTCCCATGTATCCAAACAGAGGAAAAAATAATTAGTAGTATA
>ENSG00000252341|ENST00000516532|Y RNA [Source:RFAM;Acc:RF00019]|Y_RNA
CGGGGCAAGATCCTGTCTCAAAAAACAAAACAAAAAAAAATCCAAGTTGAGAATCTGTCTCAGCTAGTGAGTTTAATCTGTTTCCATTTATTTAAATTTA
>ENSG00000271923|ENST00000606534|U6 spliceosomal RNA [Source:RFAM;Acc:RF00026]|U6
AAGGAACCTGCCTCCTTACTATATATCTGTGCTAATATTTATATTTTAATTCATGCACTTACTAATTTTTCAAATATTTTATATTAAAATCAGAATTTAT
>ENSG00000272337|ENST00000606352|U6 spliceosomal RNA [Source:RFAM;Acc:RF00026]|U6
TGATATTTGAGGGCTCAAGTCTCCATTTAATTTTTTTTCTTAATACTTCAGTGAACACATGAAACTTGTCTTTGTTAGCATGGATTAAAATTAGAGTCGG
>ENSG00000252328|ENST00000516519|Vault RNA [Source:RFAM;Acc:RF00006]|Vault
GCTCCCACTTTTTGTCACTTTTTCTCTTTATACATTATGGAAGCTCAGTAATGATTAAATAGATGAATGATGTTTTAAAAATATATTTTAAAAAAGCAAA
>ENSG00000252759|ENST00000516950|Y RNA [Source:RFAM;Acc:RF00019]|Y_RNA
GGCTCAAGCAGCCCTCCTGCTTCGGCCTCCCAAAGTGCTGGGGTTACAGGCATAAGCCACGGCCCCCGACCCAATTGGAGGGCTTTAAAAATTATACTCA
```

Table S22 - PWM for the PSE derived from PolIII sequences

Position weight matrix for the PSE element for polIII transcripts.

```
log-odds matrix: alength= 4 w= 9 n= 10 bayes= 6.50779 E= 1.2e+001
-997   74  208 -997
 -66  216 -997 -997
-997 -997 -997  156
-997  148 -997   56
  -8  196 -997 -997
-166 -997   86   82
-997 -997  208  -17
  66   16 -997  -17
 -66  196  -72 -997
```

Table S23 - PWM for the TATA box derived from PolIII sequences

Position weight matrix for the TATA element for polIII transcripts.

```
log-odds matrix: alength= 4 w= 15 n= 10 bayes= 6.40939 E= 5.0e+002
-997  196   27 -176
-997  148   86  -76
 166 -997 -997 -997
 -66 -997   27   82
 -66 -997  160  -17
-166   16   86   24
  34   16 -997   24
-997  -84 -997  141
-997  -84  -72  124
  -8   16   27  -17
 166 -997 -997 -997
 151  -84 -997 -997
 151 -997  -72 -997
  -8  -84 -997   82
 114 -997 -997  -17
```

Table S24 - 100nt upstream of human PolII sequences
100nt upstream of curated human sequences known to be transcribed by polII. Using these sequences we created the position weight matrices for the PSEA and PSEB promoter elements for polII transcripts.

```
>ENSG00000200156|ENST00000363286|1|RNA, U5B small nuclear 1 [Source:HGNC Symbol;Acc:10212]
TAAAATTTTTTCTAGGTATAGAACCTTGGCATTCACTAGTCACCATCACTATACTAGGAGTTTCTGTTACCCGAGAAACGAGTTATGAAATTAACAAGC
>ENSG00000199568|ENST00000362698|1|RNA, U5A small nuclear 1 [Source:HGNC Symbol;Acc:10211]
TGTATAAACTTTCTCAGGTAGTAACCCTTGGGATTAGTAGACACCATCAGTGTACTAGGAATTGCAGTTACCCGAAAATTGAGTTACAGAAGTAACTGGT
>ENSG00000200169|ENST00000363299|-1|RNA, U5D small nuclear 1 [Source:HGNC Symbol;Acc:10214]
GTCTGAGACAAAAACAGAAGTCACTCTTTTGGGTTAATAGTAACCATTGCTAATCTAGTAGTGACCGTCCCCCGAGGACTGTGTGCAACCATTCCAACAC
>ENSG00000199377|ENST00000362507|-1|RNA, U5F small nuclear 1 [Source:HGNC Symbol;Acc:10216]
TCTACAGTGGAAGAAAAGCTTCTGTCTGCAGGTCCAAAGGCACCGTAAGTAGAGGGAGACCAGTCAATAGCTGGGAAGCCAGGCAAAAGGCTAACAGGCA
>ENSG00000199347|ENST00000362477|1|RNA, U5E small nuclear 1 [Source:HGNC Symbol;Acc:10215]
AAAGGCTGAAGTCAATAGCTCTTTTGGGCCGAAGGAAAGTTACCATTACCCGTTTAGGAGTAGCCGTTACCTGAGAACTGTAGTGTCGACGACTGATGTT
>ENSG00000201142|ENST00000364272|1|RNA, variant U1 small nuclear 8 [Source:HGNC Symbol;Acc:48315]
GGAAAGCGTTTTGGAAGAATGGCGCGACGAGCGAGCAGAGGGGAAGGTGGTGACCCTGAGCGCTCGGCTAGGGGAGAGGAGGCTGTGCTGTTTCTCCTCT
>ENSG00000201558|ENST00000364688|1|RNA, variant U1 small nuclear 6 [Source:HGNC Symbol;Acc:48314]
GGAGGGCTGGGGGGAGGGGGGGTGTGCGCGGGGCAAGTGACCGTGCGTGTAAAGGGTGAAGCGTGTGAGGCTGCCGGCGGGGCGGAGAGTGCAATAACTC
>ENSG00000200795|ENST00000363925|-1|RNA, U4 small nuclear 1 [Source:HGNC Symbol;Acc:10192]
AGCAAGCAATAAGTGAAGATTTTTCCATAGGCCCTAAACTCACCTTTGCGAAATAGGAAGCTGGTTTATTGGGAGTGATGAGCAGGGGGCGTAACAAATT
>ENSG00000201183|ENST00000364313|1|RNA, variant U1 small nuclear 12 [Source:HGNC Symbol;Acc:48317]
GCTAGGATGGCTCCGGGATGCGCGTGACGCAAGTGACCTTGCGTGTAAAGGGTGAGGAATATGAGGCTGCGGCGGGGCGGAGGGGTGTGAGCTTATACTT
>ENSG00000202538|ENST00000365668|-1|RNA, U4 small nuclear 2 [Source:HGNC Symbol;Acc:10193]
TGGGAACACGTCGTATACACGGACACACGGGCAGGCACTCACCCTCAATGTAATGGTAGTCATCATCCGTGGGGGAGCGGGGCGCGAACAGAACCTTTCC
>ENSG00000206968|ENST00000384241|-1|RNA, variant U1 small nuclear 16 [Source:HGNC Symbol;Acc:48321]
GGGGCGGGGGGGGTGGGGGGGGGGCGTGCGCGGGGCAAGTGACCGTGCGTGTAAAGGGTGAAGCGTGTGAGGCTGTGGCGGGGCGGAGGTGCAAAAGCTC
>ENSG00000207106|ENST00000384378|-1|RNA, variant U1 small nuclear 4 [Source:HGNC Symbol;Acc:48312]
GCTAGGATGGCTCCTGGATGCGCGTGACGCAAGTGACCTTGCGTGTAAAGGGTGAGGCATATGAGGCTGCGGCGGGGCGGAGGGGCGTGAGCTTATACTT
>ENSG00000206828|ENST00000384101|-1|RNA, variant U1 small nuclear 5 [Source:HGNC Symbol;Acc:48313]
GCTAGGATGGCTCCTGGATGCGCGTGACGCAAGTGACCTTGCGTGTAAAGGGTGAGGCATATGAGGCTGCGGCGGGGCGGAGGGGCGTGAGCTTATACTT
>ENSG00000206585|ENST00000383858|-1|RNA, variant U1 small nuclear 9 [Source:HGNC Symbol;Acc:37499]
GTGTCAGGGCTAGGAAGGCTCGGGGGTGCGCGGGGCAAGTGACCATGTGTGTAAAGGGTGAGGTATATGGAGCTGTGACAGGGCAGAAGTGTGTGAAGTC
>ENSG00000202496|ENST00000365626|1|RNA, variant U1 small nuclear 20 [Source:HGNC Symbol;Acc:48325]
GCTAGGATGGCTCCTGGATGCGCGTGACGCAAGTGACCTTGCGTGTAAAGGGTGAGGCATATGAGGCTGCGGCGGGGCGGAGGGGCGTGAGCTTATACTT
>ENSG00000206737|ENST00000384010|-1|RNA, variant U1 small nuclear 18 [Source:HGNC Symbol;Acc:37496]
GCTGGGGGGGGGGGGGGGGGGGGGGCTGCGCGGGGCAAGTGACCGTGTGTGTAAAGGGTGAAGCGTGTGAGGCTGTGGCGGGGCGGAGGTGCAAGAGCTC
>ENSG00000207205|ENST00000384476|-1|RNA, variant U1 small nuclear 15 [Source:HGNC Symbol;Acc:48320]
GGCGGGGGGGGTGGGGGGGGGGGGCGTGCGCGGGGCAAGTGACCGTGCGTGTAAAGGGTGAAGCGTGTGAGGCTGTGGCGGGGCGGAGGTGCAAAAGCTC
>ENSG00000207005|ENST00000384278|1|RNA, U1 small nuclear 2 [Source:HGNC Symbol;Acc:10123]
TGTCAGGGCTGGAAAGGGCTCGGGAGTGCGCGGGGCAAGTGACCGTGTGTGTAAAGAGTGAGGCGTATGAGGCTGTGTCGGGGCAGAGCCCGAAGATCTC
>ENSG00000206652|ENST00000383925|-1|RNA, U1 small nuclear 1 [Source:HGNC Symbol;Acc:10120]
TGTCAGGGCTGGAAAGGGCTCGGGAGTGCGCGGGGCAAGTGACCGTGTGTGTAAAGAGTGAGGCGTATGAGGCTGTGTCGGGGCAGAGGCACAACGTTTC
>ENSG00000202064|ENST00000365194|-1|RNA, variant U1 small nuclear 11 [Source:HGNC Symbol;Acc:10134]
GTCCCAGCTGTGTGTCAGGGCTAGGAGGGCTGGGGGGTGCGCGGGGCAAGCGACCGTGCGTGTAAAGGGTGAGGCGTACGGGGCGGAGGTGCAGGAGCTC
>ENSG00000206694|ENST00000383967|1|RNA, variant U1 small nuclear 3 [Source:HGNC Symbol;Acc:48311]
GCTAGGATGGCTCCGGGATGCGCGTGACGCAAGTGACCTTGCGTGTAAAGGGTGAGGAATATGAGGCTGCGGCGGGGCGGAGGGGTGTGAGCTTATACTT
>ENSG00000252135|ENST00000516326|-1|RNA, variant U1 small nuclear 2 [Source:HGNC Symbol;Acc:48308]
AGGGTGGTTGGGGATTGGTGGGGGTGGTTCTCAGAGCAAGTGACCATGCGTGTAAAGGTGAGGCGTATGGAGCTGTGGTGGGGCAGAGGTATGTGGACTG
>ENSG00000207501|ENST00000384770|1|RNA, variant U1 small nuclear 14 [Source:HGNC Symbol;Acc:48319]
GGGGGGTGGGGTGGGGGGGGGGGGCGTTCGCGGGGCAAGTGACCGTGCGTGTAAAGGGTGAAGCGTGTGAGGCTGTGGCGGGGCGGAGGTGCAAAAGCTC
>ENSG00000207340|ENST00000384610|-1|RNA, variant U1 small nuclear 10 [Source:HGNC Symbol;Acc:48316]
GGTGGGGGTGGGGGGCGGTGGGGGGTTGCGCGTGGGAAGTGACCGTGCGTGTAAGGGGTGAGGCGTATGGAGCTGTGGCAGGGCGGAGGCGTATGATCTC
```

```
>ENSG00000238471|ENST00000459346|-1|U2 spliceosomal RNA [Source:RFAM;Acc:RF00004]
TTAAGTACCATTAGAATGTAAGGATTCTTTTTAAAAAATTTGATTGTGCAGGGTTGGTTATTCAACCAATATGCAATACAATATTCAATACTGTATATTC
>ENSG00000207418|ENST00000384687|-1|RNA, variant U1 small nuclear 7 [Source:HGNC Symbol;Acc:37500]
GTGTCAGGGCTAGGAAGGCTCGGGGGTGCGCGGGGCAAGTGACCATGTGTGTAAAGGGTGAGGTATATGGAGCTGTGACAGGGCAGAAGTGTGTGAAGTC
>ENSG00000212456|ENST00000391154|1|RNA, variant U1 small nuclear 13 [Source:HGNC Symbol;Acc:48318]
TAGGAGGGCTGGGGGGGGCGGGGAGGTGCGCGGGGCAAGTGACCGTGCGTGTAAAGGGTGAAGCGTGTGAGGCTGTGGCGGGGCGGAGGTGCAAAAGCTC
>ENSG00000238953|ENST00000459035|1|U6atac minor spliceosomal RNA [Source:RFAM;Acc:RF00619]
AATCAACAATAAAAAACAAACAGGCTGAGCAGGAAACTGGAAAGAGACATGAAGAGACATTTCACTGAGGAGGATCCATAGAAGGAAAACAAGCACATAT
>ENSG00000207389|ENST00000384659|1|RNA, U1 small nuclear 4 [Source:HGNC Symbol;Acc:10128]
TGTCAGGGCTGGAAAGGGCTCGGGAGTGCGCGGGGCAAGTGACCGTGTGTGTAAAGAGTGAGGCGTATGAGGCTGTGTCGGGGCAGAGCCCGAAGATCTC
>ENSG00000238850|ENST00000459529|1|U2 spliceosomal RNA [Source:RFAM;Acc:RF00004]
TAAAGTACCATTAGAATGTAAGGATTCTTTTTAAAAAATATGATTGTGCAGGGTTGGTTATTCAACCAGTATGCAATACAATACTCAATACTGTATATTC
>ENSG00000207349|ENST00000384619|-1|RNA, variant U1 small nuclear 17 [Source:HGNC Symbol;Acc:48322]
GGGCGGGGTGGGGGTGGGGGTGCGGGTGCGCGGGGCAAGTGACCGTGTCTGTAAAGGTTGAGGCGTATGGAGCTGTCGCAGGGCGGAGATGTGTGAACTC
>ENSG00000207513|ENST00000384782|-1|RNA, U1 small nuclear 3 [Source:HGNC Symbol;Acc:10130]
TGTCAGGGCTGGAAAGGGCTCGGGAGTGCGCGGGGCAAGTGACCGTGTGTGTAAAGAGTGAGGCGTATGAGGCTGTGTCGGGGCAGAGCCCGAAGATCTC
>ENSG00000251784|ENST00000515975|1|U4atac minor spliceosomal RNA [Source:RFAM;Acc:RF00618]
AAAAAAAAAACAAGGAGACATTTAGAAACAAACAACTGATCCCTGAAATGAGCATCAACGGGACAATGGCCAATACAAGTCTGTTAAGGATGTGGAGCAA
>ENSG00000212544|ENST00000391242|-1|RNA, variant U1 small nuclear 19 [Source:HGNC Symbol;Acc:48324]
AGGAGGGCTGGGGGGGGGGGGGGGGTGCGCGGGGCAAGTGACCGTGCGTGTAAAGGGTGAAGCGTGTGAGGCTGTGGCGGGGCGGAGGTGCAAAAGCTC
>ENSG00000238421|ENST00000459549|-1|U1 spliceosomal RNA [Source:RFAM;Acc:RF00003]
GGCGGCATGGGGGCGGGGTGGGGGAATGCGCGGGGCAAGTGACCGTGCGTGTAAGGGGTGAGGCGTATGGAGCTGTGGCAGGGCGGAGGTGCGTTCATTC
>ENSG00000238603|ENST00000459096|-1|RNA, variant U1 small nuclear 1 [Source:HGNC Symbol;Acc:10133]
GGTGGGGGTGGGGGGCGGTGGGGGGTTGCGCGTGGGAAGTGACCGTGCGTGTAAGGGGTGAGGCGTATGGAGCTGTGGCAGGGCGGAGGCGTATGATCTC
>ENSG00000221676|ENST00000408749|-1|RNA, U6atac small nuclear (U12-dependent splicing) [Source:HGNC Symbol;Acc:3401
TTTATAAATGTACCTCCATGGATAGCGAACAAGAAGTCACCCTCACCGAAAGGCGAGTGGAGCTTTCGTCCTTAAATAAAGTGCGCAGGGAAGCCGAGGC
>ENSG00000264389|ENST00000582426|-1|U1 spliceosomal RNA [Source:RFAM;Acc:RF00003]
TGAAAACAATCCAAATATCCTTCAACTAGTAAATGGATAAACAAAATGGTACTACATCCATGCAAGGCAATATCATTCAACAATAAAAGGGGACAAAAAT
>ENSG00000271739|ENST00000606574|-1|U1 spliceosomal RNA [Source:RFAM;Acc:RF00003]
ATGCAGCAAAAGTGGTTCTAAGAGTGAAGTTAGACCTACATGAAACATATAAACACCTGCATGAAAACAGAAGAAATATATCAAAGAAATAACCAAACAC
>ENSG00000264252|ENST00000580951|1|RNA, variant U1 small nuclear 12 [Source:HGNC Symbol;Acc:48317]
GCTAGGATGGCTCCGGGATGCGCGTGACGCAAGTGACCTTGCGTGTAAAGGGTGAGGAATATGAGGCTGCGGCGGGGCGGAGGGGTGTGAGCTTATACTT
>ENSG00000265248|ENST00000583575|-1|U1 spliceosomal RNA [Source:RFAM;Acc:RF00003]
AAACAATCCAAATATCCTTCAACTAGTAAATGGACAAACAAAATGGATCAACTACACCCATGCAAGGCAATATCATTCAACAATAAAAGGGAACAAAACT
>ENSG00000263971|ENST00000579513|-1|U1 spliceosomal RNA [Source:RFAM;Acc:RF00003]
TGAAAACAATCCAAATATCCTTCAACTAGTAAATGGATAAACAAAATGGTACTATGTCCATGCAAGGCAGTATCATTCAACAATAAAAGGGGACAAAAAT
>ENSG00000264998|ENST00000580281|-1|U1 spliceosomal RNA [Source:RFAM;Acc:RF00003]
TGAAAACAATCCAAATATCCTTCAACTAGTAAATAGATAAACAAAATTCAACTACATCCAGGCAAGGCAATGTCATTCAACAATAAAAGGGGGCAAAACT
>ENSG00000272359|ENST00000607443|-1|U4 spliceosomal RNA [Source:RFAM;Acc:RF00015]
AACATTGTCCATGTAAATTAGAATGTGCAGCCAACGCTGCATTCAAAAGCAAATTAAGAACATTAAAGGTTTTTATTATTAAAAGACATTAAAATGAACT
>ENSG00000265983|ENST00000577618|-1|U1 spliceosomal RNA [Source:RFAM;Acc:RF00003]
TGAAAACAATACAAATATCCTTCAATTAGTAAATGGATTAACAAAATGATACTACATCCATGCAAGGCAATATCATTCAACAATAAAAGGGAACAAAAAC
>ENSG00000266689|ENST00000581688|-1|U1 spliceosomal RNA [Source:RFAM;Acc:RF00003]
TGAAAACAATCCAAATTTCCATCAACTAGTAAACAGATAAACAAAATTCAACTACATCCAGGCAAGGCAATATCATTCAACAATAAAAGGGGACAAAACT
>ENSG00000263871|ENST00000579987|-1|U1 spliceosomal RNA [Source:RFAM;Acc:RF00003]
TGAAAACAATACAAATATCCTTCAATTAGTAAATGGATTAACAAAATGATACTACATCCATGCAAGGCAATATCATTCAACAATAAAAGGGAACAAAAAC
>ENSG00000270834|ENST00000603951|-1|RNA, variant U1 small nuclear 10 [Source:HGNC Symbol;Acc:48316]
GGTGGGGGTGGGGGGCGGTGGGGGGTTGCGCGTGGGAAGTGACCGTGCGTGTAAGGGGTGAGGCGTATGGAGCTGTGGCAGGGCGGAGGCGTATGATCTC
>ENSG00000270722|ENST00000605806|-1|RNA, variant U1 small nuclear 7 [Source:HGNC Symbol;Acc:37500]
GTGTCAGGGCTAGGAAGGCTCGGGGGTGCGCGGGGCAAGTGACCATGTGTGTAAAGGGTGAGGTATATGGGGCTGTGACAGGGCAGAAGTGTGTGAAGTC
>ENSG00000266860|ENST00000578081|-1|U1 spliceosomal RNA [Source:RFAM;Acc:RF00003]
TGAAAACAATACAAATATCCTTCAATTAGTAAATGGATTAACAAAATGATACTACATCCATGCAAGGCAATATCATTCAACAATAAAAGGGAACAAAAAC
>ENSG00000264704|ENST00000578102|-1|U1 spliceosomal RNA [Source:RFAM;Acc:RF00003]
AAACAATCCAAATATCCTTCAACTAGTAAATGGATAAACAAAATGGATAAACTACATTCATGCAAGGCAATATCATTCAACAGTAAAAGGGAACAAAACG
>ENSG00000268972|ENST00000594904|-1|RNA, U6atac small nuclear (U12-dependent splicing) [Source:HGNC Symbol;Acc:3401
```

```
TTTATAAATGTACCTCCATGGATAGCGAACAAGAAGTCACCCTCACCGAAAGGCGAGTGGAGCTTTCGTCCTTAAATAAAGTGCGCAGGGAAGCCGAGGC
>ENSG00000271345|ENST00000603039|-1|RNA, variant U1 small nuclear 9 [Source:HGNC Symbol;Acc:37499]
GTGTCAGGGCTAGGAAGGCTCGGGGGTGCGCGGGGCAAGTGACCATGTGTGTAAAGGGTGAGGTATATGGAGCTGTGACAGGGCAGAAGTGTGTGAAGTC
>ENSG00000272160|ENST00000607255|1|U4 spliceosomal RNA [Source:RFAM;Acc:RF00015]
CTGGAGAAGAAGGCTCCATGTTGGCCCCAGAATGGCATACTCTTGGCTCCAAAGACATTAAGTCTTCATCTTCCCACACTAAGAAACAGGAAAATTTTTA
>ENSG00000262704|ENST00000572180|1|U4 spliceosomal RNA [Source:RFAM;Acc:RF00015]
AGAGATACAAACGAAAGACCCCTAATTATAGATTTTAAAGTTTTCCTTATCCTCTTCCATTTGGCTACTAGGTGATTCTACTGTGTAAAAACTTAGCTCC
>ENSG00000270860|ENST00000603281|-1|RNA, variant U1 small nuclear 17 [Source:HGNC Symbol;Acc:48322]
GGGCGGGGTGGGGGTGGGGGTGCGGGTGCGCGGGGCAAGTGACCGTGTCTGTAAAGGTTGAGGCGTATGGAGCTGTCGCAGGGCGGAGATGTGTGAACTC
>ENSG00000270840|ENST00000605036|-1|RNA, variant U1 small nuclear 6 [Source:HGNC Symbol;Acc:48314]
AGGAGGGCTGGGGGGGAGGGGGGTGTGCGCGGGGCAAGTGACCGTGCGTGTAAAGGGTGAAGCGTGTGAGGCTGCCGGCGGGGCGGAGGTGCAATAACTC
>ENSG00000264960|ENST00000578508|-1|U1 spliceosomal RNA [Source:RFAM;Acc:RF00003]
AAACAATCCAAATATCCTTCAACTAGTAAATGGACAAACAAAATGGATCAACTACATCCATGCAAGGCAATATCATTCAACAATAAAAGGGAACAAAACT
>ENSG00000262656|ENST00000572849|1|U1 spliceosomal RNA [Source:RFAM;Acc:RF00003]
TTTCTGAGCAAAGAAAACGATCTGTTCCCCTACTCATGTGGGCTTGTATTCATGCATATATTTACTTGTTAATCCATTCCTTCAATAAATGGGAATTCAA
>ENSG00000270483|ENST00000581810|1|RNA, variant U1 small nuclear 5 [Source:HGNC Symbol;Acc:48313]
GCTAGGATGGCTCCGGGATGCGCGTGAGGCAAGTGACCTTGCGTGTAAAGGGTGAGGAATATGAGGCTGCGGCGGGGCGGAGGGGCGTGAGCTTATACTT
>ENSG00000271684|ENST00000603895|-1|RNA, variant U1 small nuclear 18 [Source:HGNC Symbol;Acc:37496]
GGGGTGGGGGGGGGGGGGGGGGGCTGCGCGGGGCAAGTGACCGTGCGTGTAAAGGGTGAAGCGTGTGAGGCTGTGGCGGGGCGGAGGTGCAAGATCTC
>ENSG00000264893|ENST00000584018|-1|U1 spliceosomal RNA [Source:RFAM;Acc:RF00003]
AAACAATCCAAATATCCTTCAACTAGTAAATGGATAAACAAAATGGATAAACTACATTCATGCAAGGCAATATCATTCAACAGTAAAAGGGAACAAAACG
>ENSG00000263374|ENST00000579215|-1|RNA, variant U1 small nuclear 11 [Source:HGNC Symbol;Acc:10134]
GTCCCAGCTGTGTGTCAGGGCTAGGAGGGCTGGGGGGTGCGCGGGGCAAGCGACCGTGCGTGTAAAGGGTGAGGCGTACGGGGCGGAGGTGCAGGAGCTC
>ENSG00000265044|ENST00000579257|1|RNA, variant U1 small nuclear 15 [Source:HGNC Symbol;Acc:48320]
GGCGGGGGGGGTGGGGGGGGGGGGCGTGCGCGGGGCAAGTGACCGTGCGTGTAAAGGGTGAAGCGTGTGAGGCTGTGGCGGGGCGGAGGTGCAAAAGCTC
>ENSG00000272020|ENST00000606575|-1|U1 spliceosomal RNA [Source:RFAM;Acc:RF00003]
TTGGGAAGGTCTCCATGTTCTGAACTGGGGGTCCATATTCTGAAAGGTGGGCATGCGCGGCATCACCATTGCTGGGGCTGCCGTCAAAACACATAGTGCT
>ENSG00000266884|ENST00000580213|-1|U1 spliceosomal RNA [Source:RFAM;Acc:RF00003]
TGAAAACAATCCAAATATCCTTCAACTAGTAAATAGATAAACAAAATTCAACTACATCCAGGCAAGGCAATGTCATTCAACAATAAAAGGGGGCAAAACT
>ENSG00000268065|ENST00000595178|1|RNA, variant U1 small nuclear 14 [Source:HGNC Symbol;Acc:48319]
GGGGGGGTGGGGTGGGGGGGGGGGCGTTCGCGGGGCAAGTGACCGTGCGTGTAAAGGGTGAAGCGTGTGAGGCTGTGGCGGGGCGGAGGTGCAAAAGCTC
>ENSG00000262278|ENST00000577078|-1|U2 spliceosomal RNA [Source:RFAM;Acc:RF00004]
AGAGGATGGCTTGAGCCCAGGAGTTTGAGACTAGCCTGGACAGCATGTGAGACCCCACTTCTTTCTAAAAAAAAAAAAAAAAAAAAAATCAATTGTTTTG
>ENSG00000263451|ENST00000577892|1|RNA, variant U1 small nuclear 8 [Source:HGNC Symbol;Acc:48315]
GGAAAGCGTTTTGGAAGAATGGCGCGACGAGCGAGCAGAGGGGAAGGTGGTGACCCTGAGCGCTCGGCTAGGGGAGAGGAGGCTGTGCTGTTTCTCCTCT
>ENSG00000265838|ENST00000581325|-1|U1 spliceosomal RNA [Source:RFAM;Acc:RF00003]
TGAAAACAATCCAAATATCCTTCAACTAGTAAATGGATAAACAAAATGGTACTATGTCCATGCAAGGCAGTATCATTCAACAATAAAAGGGGACAAAAAT
>ENSG00000266081|ENST00000584937|-1|U1 spliceosomal RNA [Source:RFAM;Acc:RF00003]
TGAAAACAATCCAAATATCCTTCAACTAGTAAATGGATAAACAAAATGGTACTACGTCCATGCAAGGCAGTATCATTCAACAATAAAAGGGGACAAAAAT
>ENSG00000265059|ENST00000582199|-1|U1 spliceosomal RNA [Source:RFAM;Acc:RF00003]
AAACAATCCAAATATCCTTCAACTAGTAAATGGATAAACAAAATGGATAAACTACATTCATGCAAGGCAATATCATTCAACAGTAAAAGGGAACAAAACG
>ENSG00000265074|ENST00000580475|-1|U1 spliceosomal RNA [Source:RFAM;Acc:RF00003]
AAACAATCCAAATATCCTTCAACTAGTAAATGGACAAACAAAATGGATCAACTACACCCATGCAAGGCAATATCATTCAACAATAAAAGGGAACAAAACT
>ENSG00000264640|ENST00000577825|-1|U1 spliceosomal RNA [Source:RFAM;Acc:RF00003]
AAACAATCCAAATATCCTTCAACTAGTAAATGGACAAACAAAATGGATCAACTACATCCATGCAAGGCAATATCATTCAACAATAAAAGGGAACAAAACT
```

Table S25 - PWM for the PSEA derived from PolII sequences

Position weight matrix for the PSEA element for polII transcripts.

-209	204	-82	-291
163	-1285	-1285	-91
165	-1285	-304	-191
11	-1285	101	-391
-341	-269	-462	220
-17	-52	73	-159
123	-369	-1285	61
-1285	233	-231	-191
-209	242	-1285	-1285
-33	-137	1	85
-441	-369	-1285	226
-283	107	76	-291
29	126	-462	9
11	-88	76	-291
-1285	122	-1285	155

Table S26 - PWM for the PSEB derived from PolII sequences

Position weight matrix for the PSEB element for polII transcripts.

-441	-369	-263	218
159	-169	-1285	-132
163	-210	-1285	-159
165	-1285	-263	-232
-341	-110	86	48
-51	-169	110	-291
-141	-1285	123	-91
39	-1285	-263	148
-241	-37	58	61
152	-269	-1285	-45
71	-369	23	-74
44	-210	69	-291
-96	209	-363	-132
104	-1285	23	-391
44	-269	-304	141

Figure S1a - `miRDeep` **predictions with read profile best aligning with snoRNAs**

Five read profiles for `miRDeep` predicted miRNAs where the read profiles are best aligned with snoRNA read profiles according to `deepBlockAlign`. From top to bottom the best matching read profiles were found to be SNORD61, SNORA3, snoU89, SNORD49, and SNORA52. In the figure, the `RNAz` track and the `deepBlockAlign` track are colored dark blue; the high confident annotation based on `miRDeep` is colored with a lighter blue; the number of reads from the small RNA library that cover each base is shown in the bottom of each plot (blue for reads on the negative strand and red for reads on the positive strand). **(Continued on next page)**

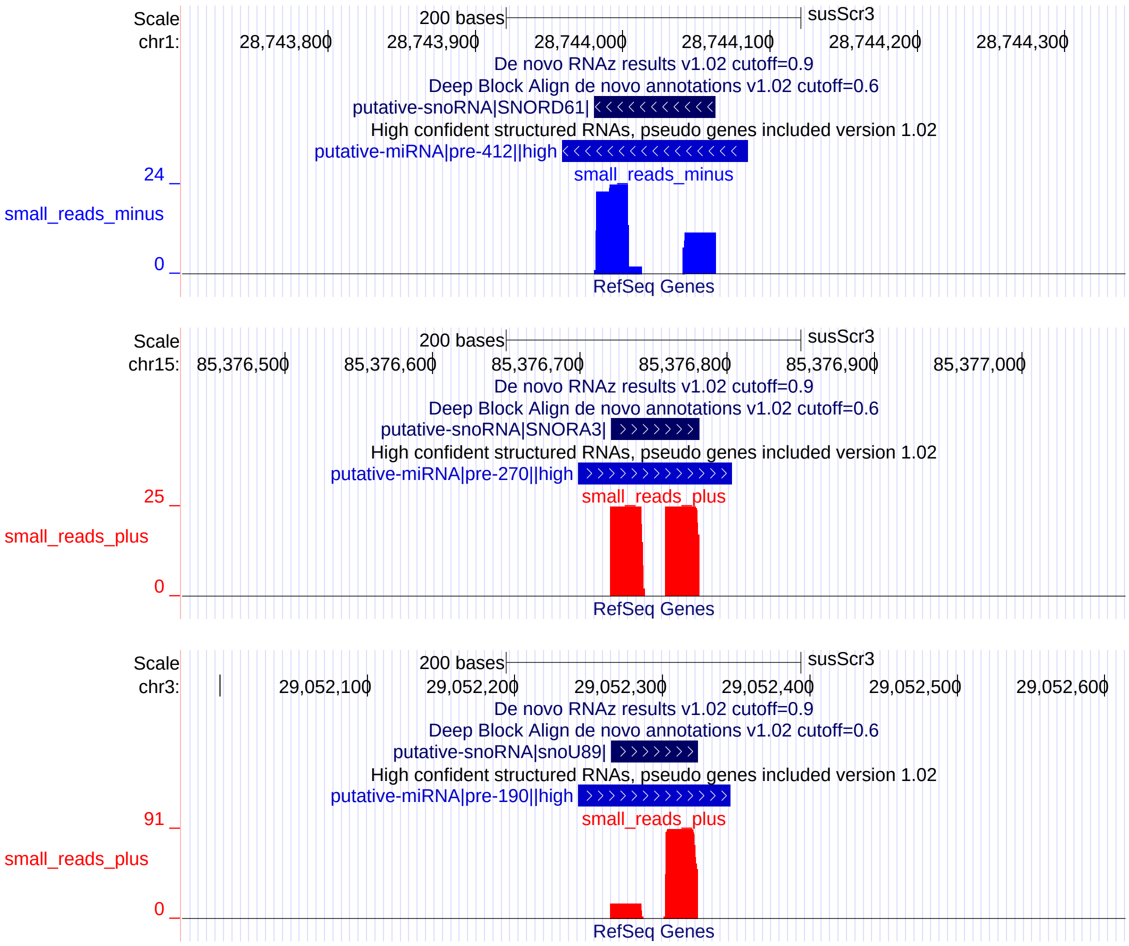

Figure S1b - `miRDeep` **predictions with read profiles best aligning with snoRNAs**
Five read profiles for `miRDeep` predicted miRNAs where the read profiles are best aligned with snoRNA read profiles according to `deepBlockAlign`. From top to bottom the best matching read profiles were found to be SNORD61, SNORA3, snoU89, SNORD49, and SNORA52. In the figure, the `RNAz` track and the `deepBlockAlign` track are colored dark blue; the high confident annotation based on `miRDeep` is colored with a lighter blue; the number of reads from the small RNA library that cover each base is shown in the bottom of each plot (blue for reads on the negative strand and red for reads on the positive strand). (Continued from previous page)

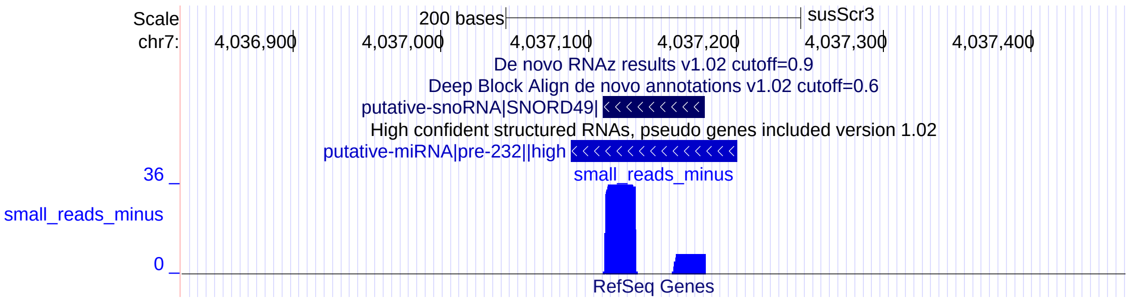

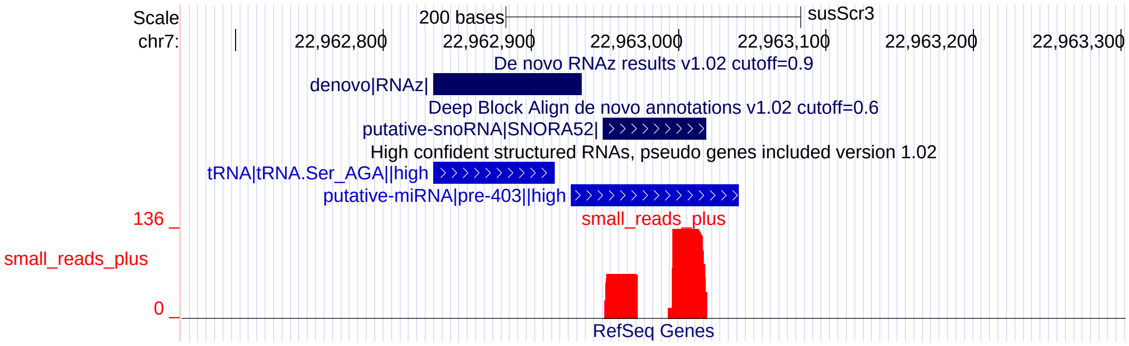

Figure S2 - `miRDeep` **prediction with read profile not aligning to those of known ncRNAs**
Four read profiles for `miRDeep` predicted miRNAs where the read profiles do not aligning to those of any known ncRNA within the `deepBlockAlign` score cutoff of 0.6. In the figure, the `RNAz` track and the `deepBlockAlign` track are colored dark blue; the high confident annotation based on `miRDeep` is colored with a lighter blue; the number of reads from the small RNA library that cover each base, is shown in the bottom of each plot (blue for reads on the negative strand and red for reads on the positive strand).

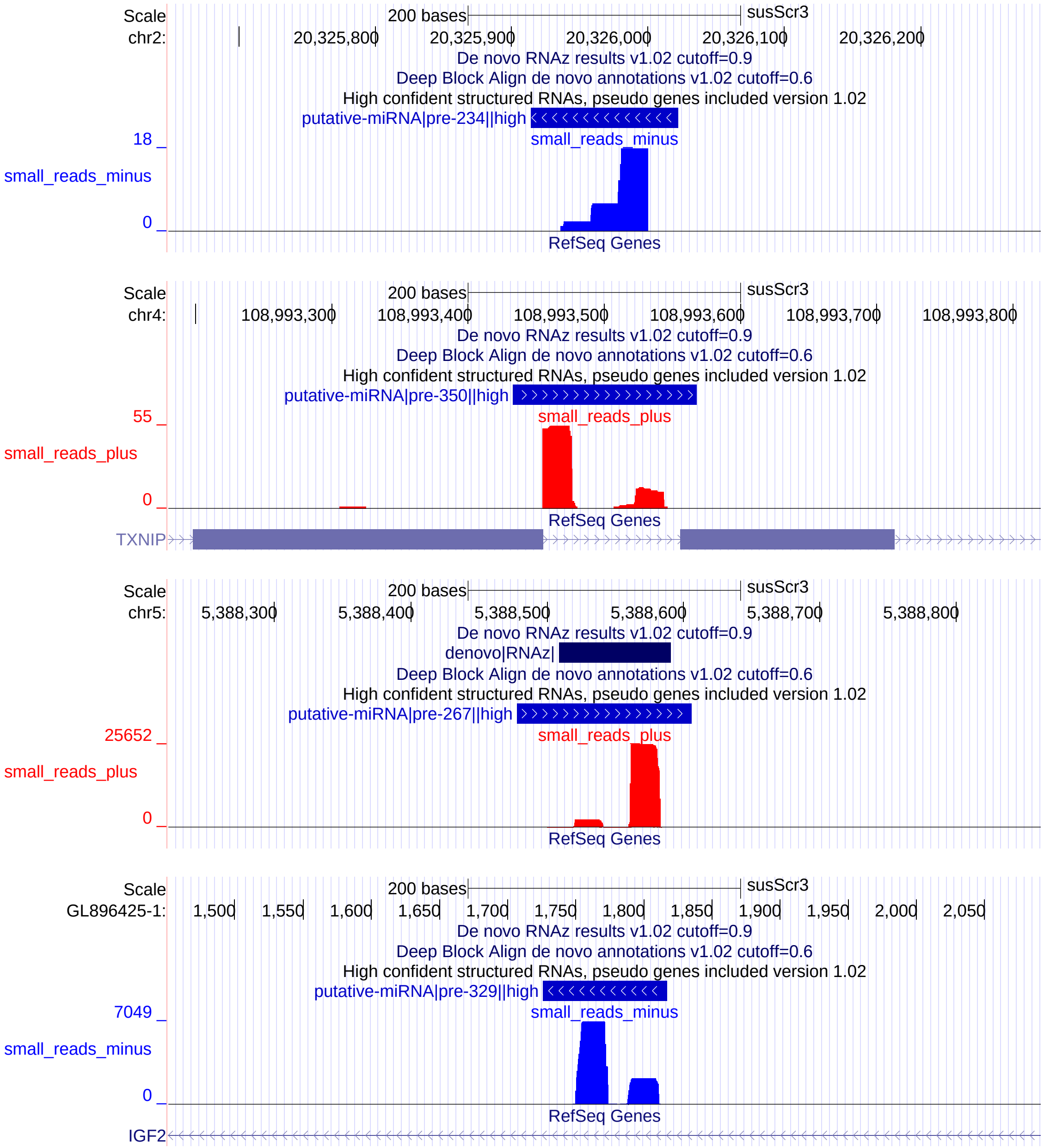

Figure S3 - Read profile annotated by `deepBlockAlign` with overlapping `RNAz` loci

Three read profiles for RNAs, which overlap with an `RNAz` locus and thus have conserved structure. However, they are not annotated by the high confident homology pipeline, nor detected by the `miRDeep` program. From top to bottom the read profiles are aligned with tRNA.ala, mir-876, mir-2964. In the figure, the `RNAz` track and the `deepBlockAlign` track are colored dark blue; the number of reads from the small RNA library that cover each base, is shown in the bottom of each plot (blue for reads on the negative strand and red for reads on the positive strand).

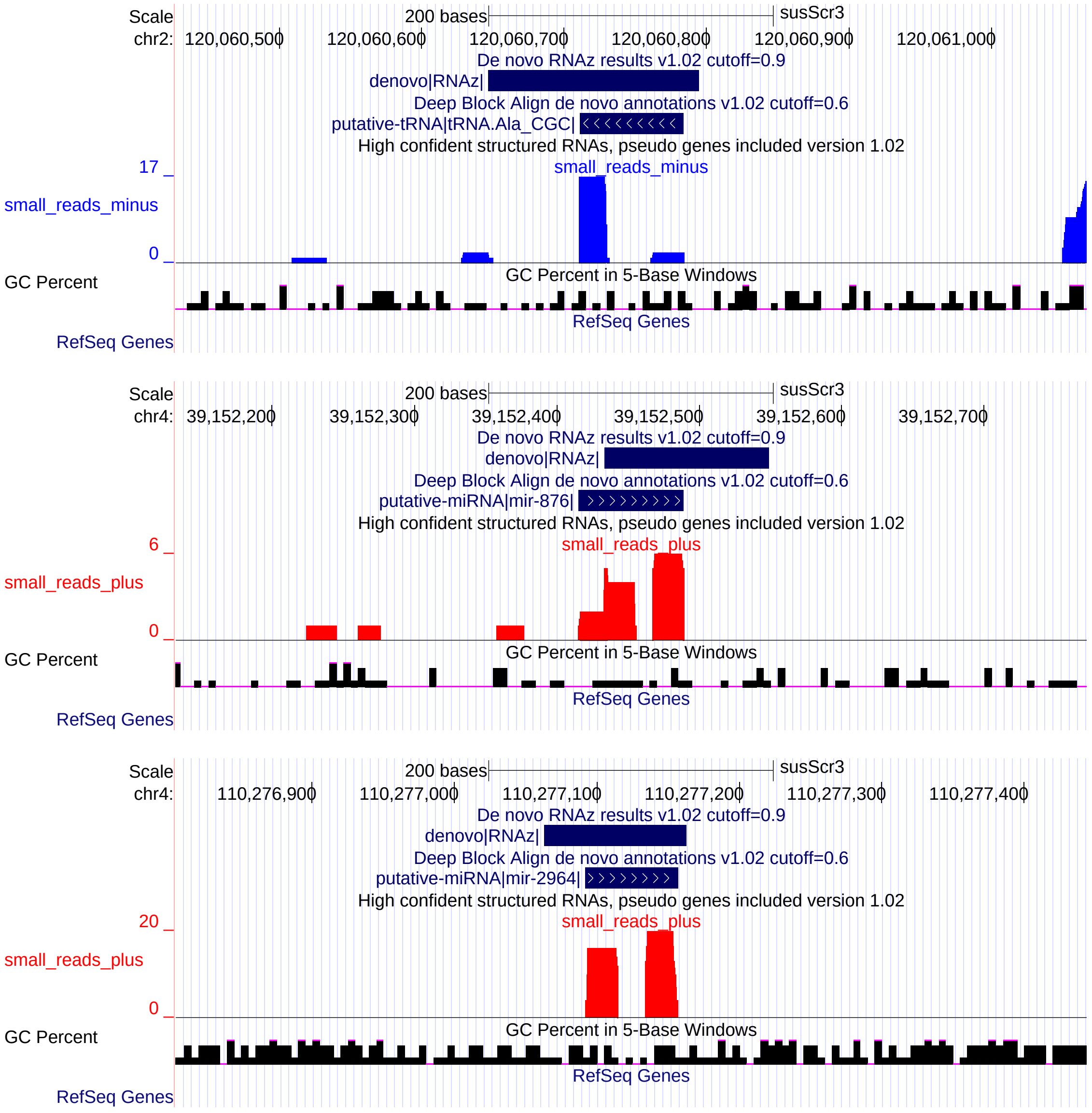

Figure S4 - Read profile annotated by `deepBlockAlign` and overlapping with mir-431

Read profile recognized by `deepBlockAlign` as a miRNA. However, the locus is missed by both high confident `BLAST` and `miRDeep`. The locus has conserved RNA structure as it overlaps with an `RNAz` annotation and medium confident `BLAST` identifies the locus as mir-431. In the figure, the `RNAz` track and `deepBlockAlign` track are colored dark blue; the medium confident annotation based on `BLAST` is colored with a lighter blue; the number of reads from the small RNA library that cover each base, is shown in the bottom of each plot (blue for reads on the negative strand and red for reads on the positive strand).

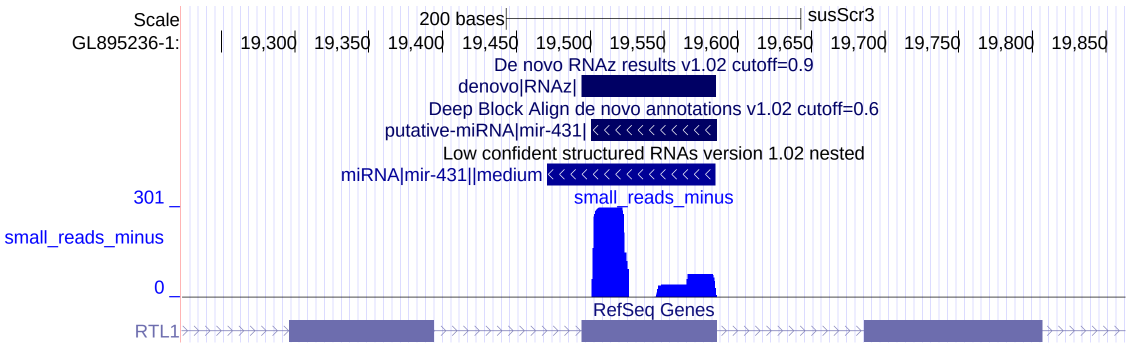

Figure S5 - Read profile annotated by `deepBlockAlign` and overlapping with mir-223

Read profile recognized by `deepBlockAlign` as a miRNA, However, the locus is missed by both high confident `BLAST` and `miRDeep`. The locus has conserved RNA structure as it overlaps with an `RNAz` annotation and medium confident `BLAST` identifies the locus as mir-223. In the figure, the `RNAz` track and `deepBlockAlign` track are colored dark blue; the medium confident annotation based on `BLAST` is colored with a lighter blue; the number of reads from the small RNA library that cover each base, is shown in the bottom of each plot (blue for reads on the negative strand and red for reads on the positive strand).

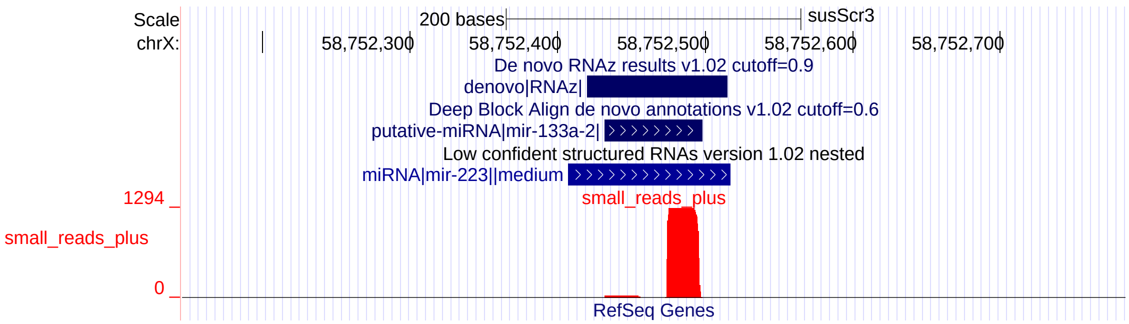

Figure S6 - Read profile annotated by `deepBlockAlign` and overlapping with mir-1388

Read profile recognized by `deepBlockAlign` as a miRNA. However, the locus is missed by both high confident `BLAST` and `miRDeep`. Medium confident `BLAST` identifies the locus as mir-1388. In the figure, the `RNAz` track and `deepBlockAlign` track are colored dark blue; the medium confident annotation based on `BLAST` is colored with a lighter blue; the number of reads from the small RNA library that cover each base, is shown in the bottom of each plot (blue for reads on the negative strand and red for reads on the positive strand).

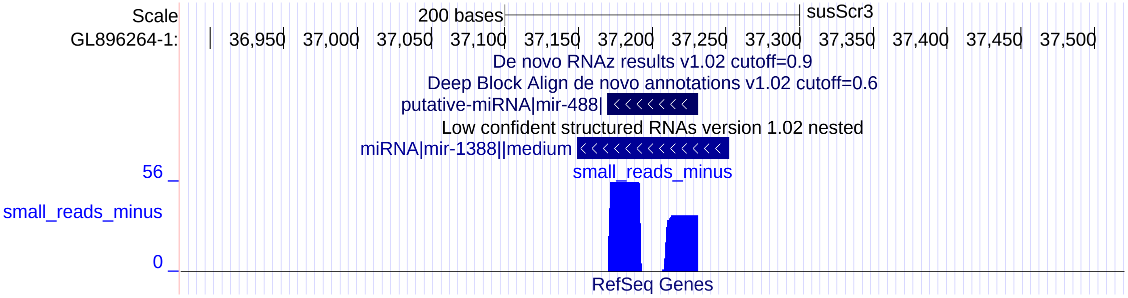

Figure S7 - Cluster size of ncRNA cluster found in the high confident annotation of the pig genome
The result of the clustering procedure of the high confident annotation. Plots the number of clusters with a given number of ncRNAs. For example, there is 28 clusters containing exactly 4 ncRNAs.

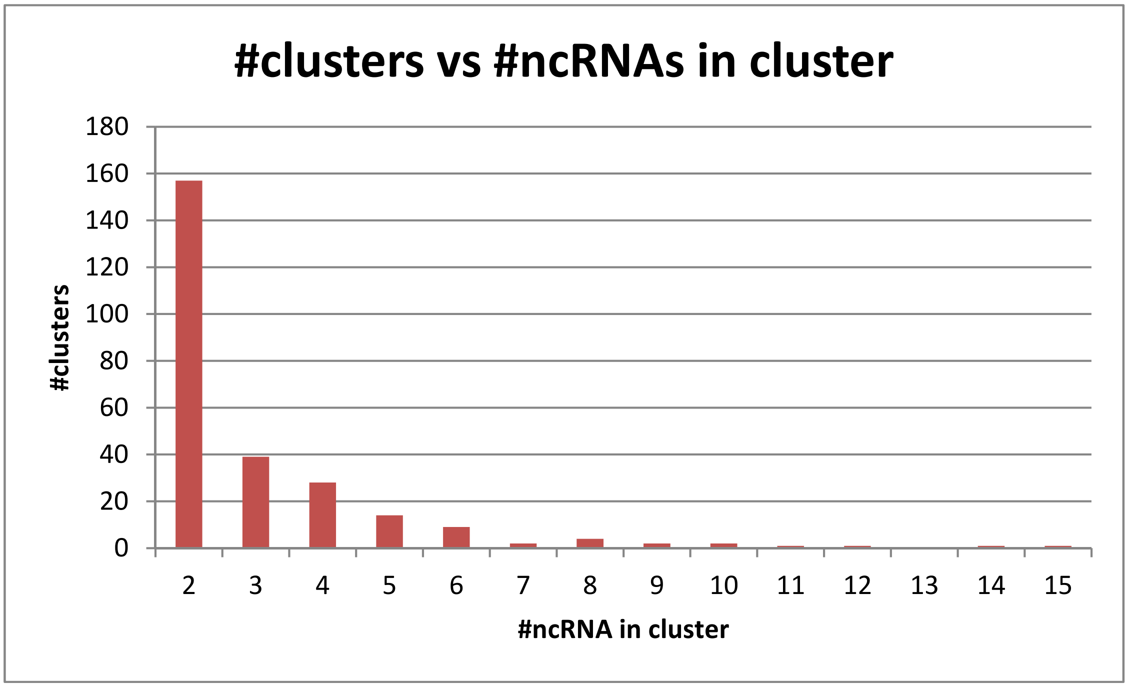

Figure S8 - Number of `RNAz` loci versus maximum sequence identity cutoff outside the Laurasiatherian lineage

The number of `RNAz` loci found with a maximum sequence identity of the loci outside the Laurasiatherian branch/lineage. For example, if we allow up to 70.0%, we would detect just under 10,000 lineage specific loci.

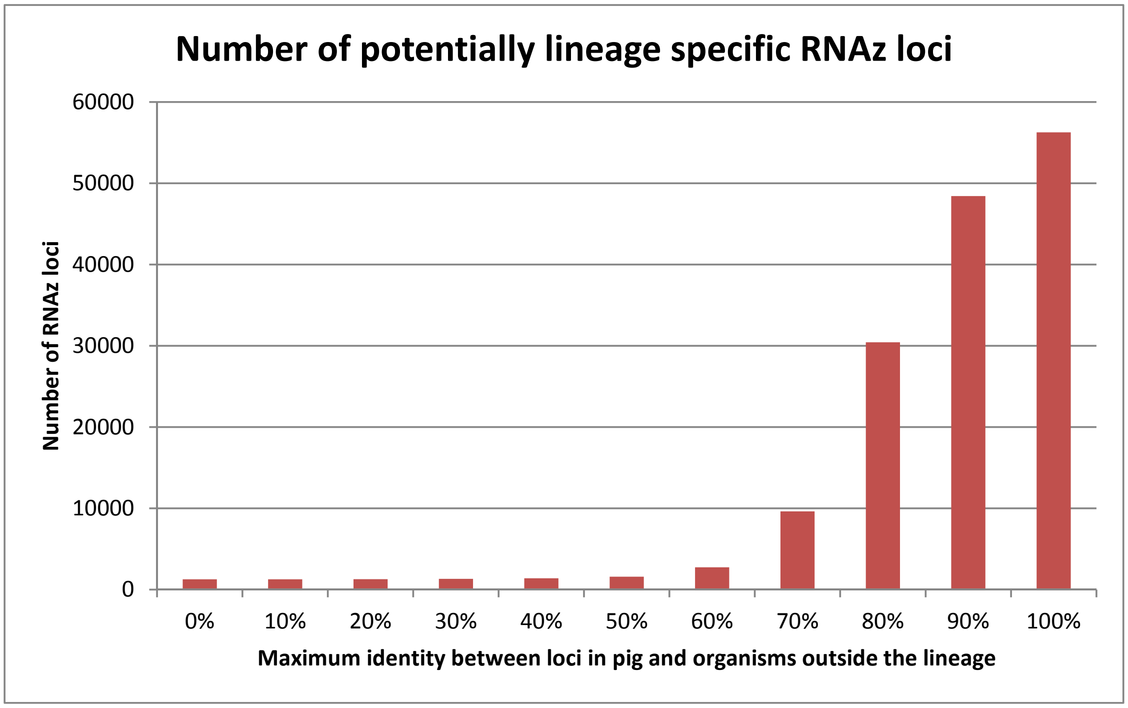

Figure S9 - Number of RNAz loci versus minimum sequence identity cutoff inside the Laurasiatherian lineage

The number of RNAz loci found with a minimum sequence identity inside the lineage. Here we only consider the subset of 1,300 RNAz loci considered to be lineage specific by looking the sequence identity outside the lineage.

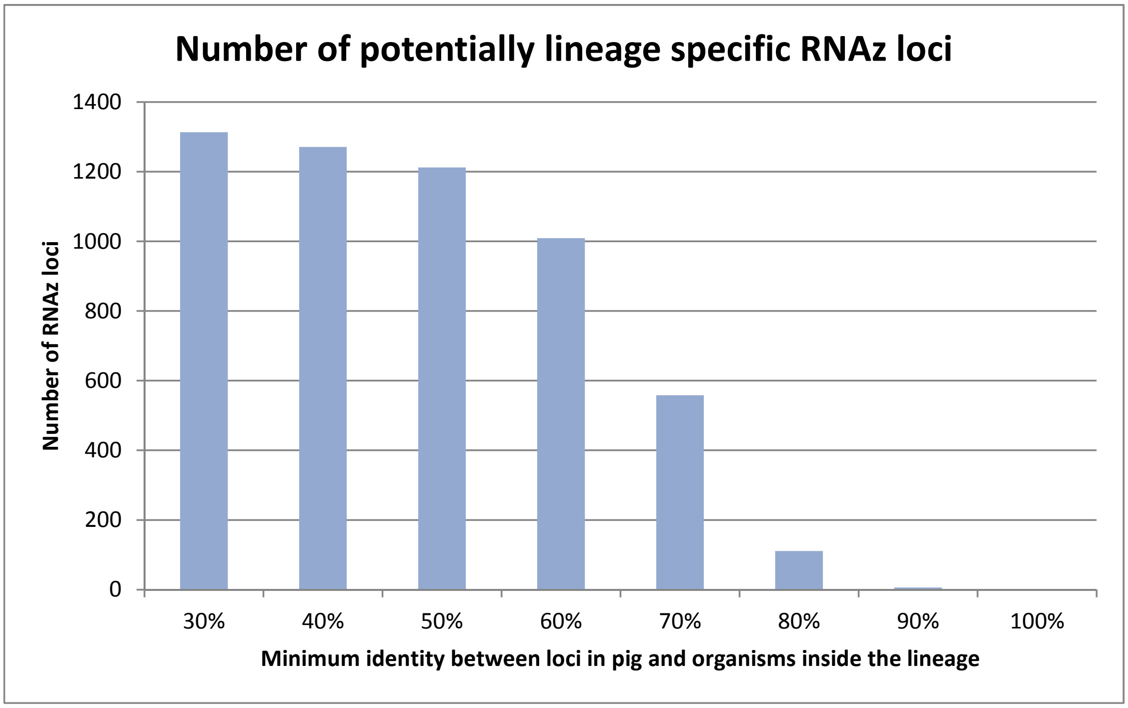

Figure S10 - Conservation of the 1,595 human miRNA loci in the pig genome

The 1,595 miRNAs known from human are found to be more or less conserved in the pig genome. The plot shows the number of miRNAs versus their conservation. For example, just over 1,000 of the 1,595 human miRNAs are at least 60% conserved by sequence in pig.

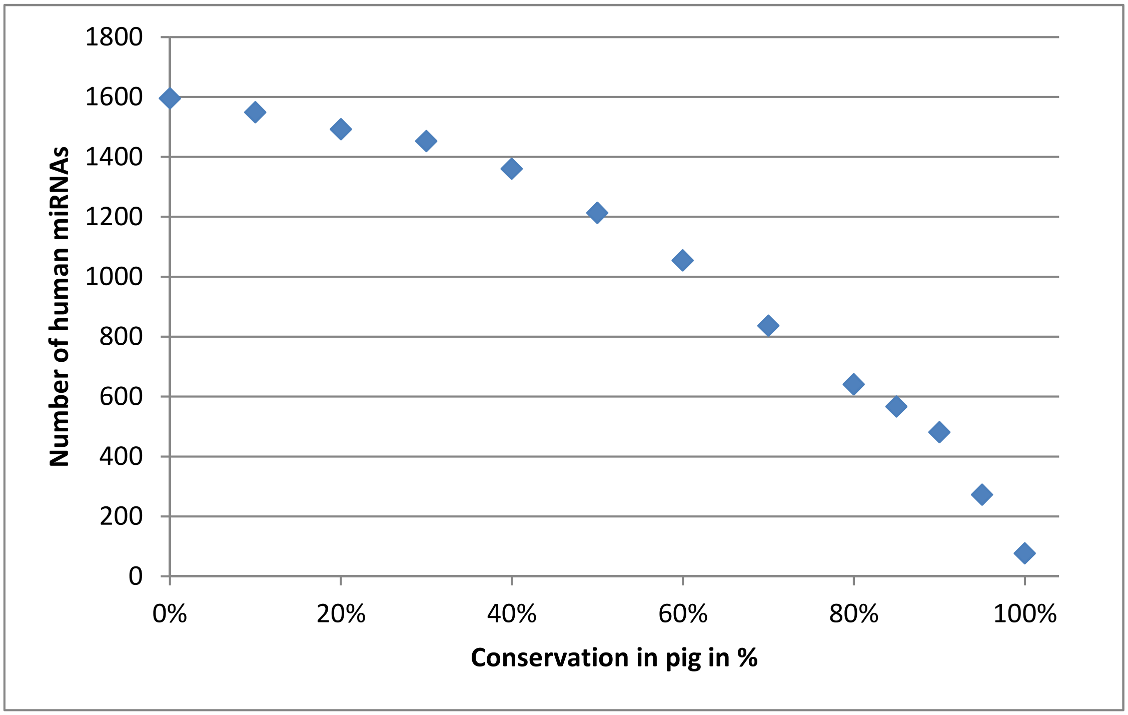

Figure S11 - Conservation in the pig genome of the human miRNA loci in miRNA clusters
Plot similar to the previous one. Here, however, we restrict the human miRNAs to those which are part of a miRNA cluster in human.

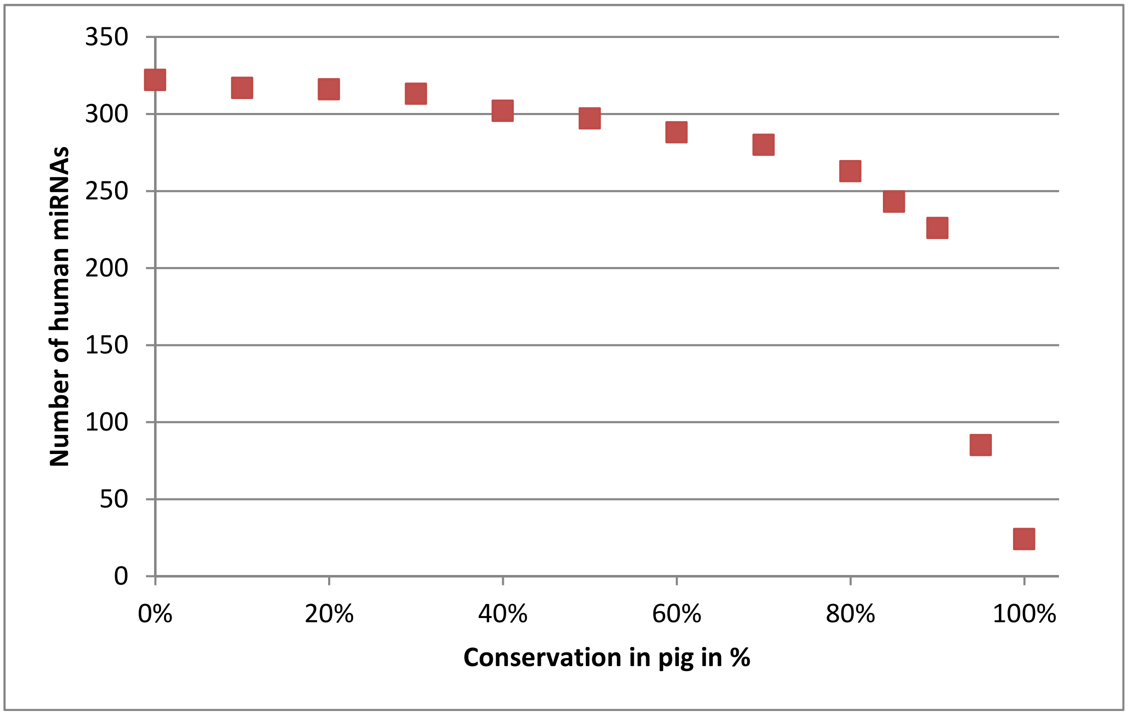

Figure S12 - mir-379 cluster in pig

Plot of the mir-379 cluster in pig. The cluster is incomplete in the assembly and only contains miRNAs from 376c to 656, while miRNAs 379-495 are missing. In the figure, the RNAz track is colored dark blue; the high confident confident annotations based are colored with a lighter blue; the curated annotation is colored green; the human miRNAs from miRBase, which have been lifted over from hg19 to pig are colored red; the number of reads from the small RNA library that cover each base, is shown in the bottom of each plot (blue for reads on the negative strand and red for reads on the positive strand).

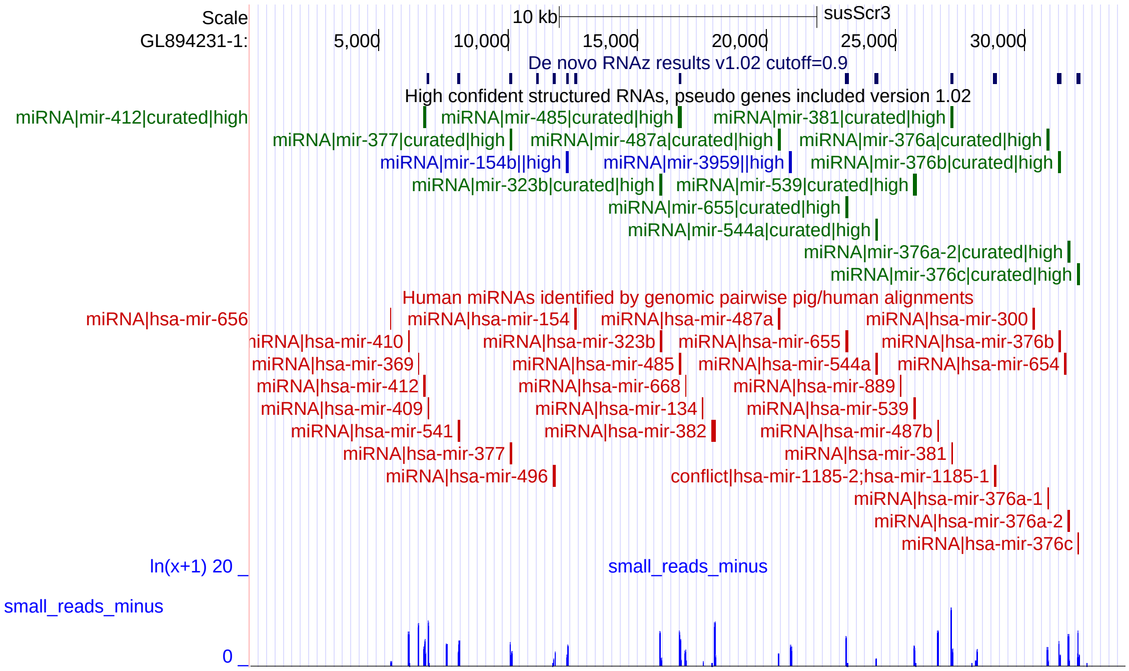

Figure S13 - mir-493 cluster in pig

The mir-493 cluster is broken in the pig assembly. miRNAs are found on both strands, and even though the miRNAs are confirmed by high confident `BLAST`, they are not conserved in the pairwise alignments. In the figure, the `RNAz` track is colored dark blue; the high confident confident annotations based are colored with a lighter blue; the curated annotation is colored green; the human miRNAs from miRBase, which have been lifted over from hg19 to pig are colored red; the number of reads from the small RNA library that cover each base, is shown in the bottom of each plot (blue for reads on the negative strand and red for reads on the positive strand).

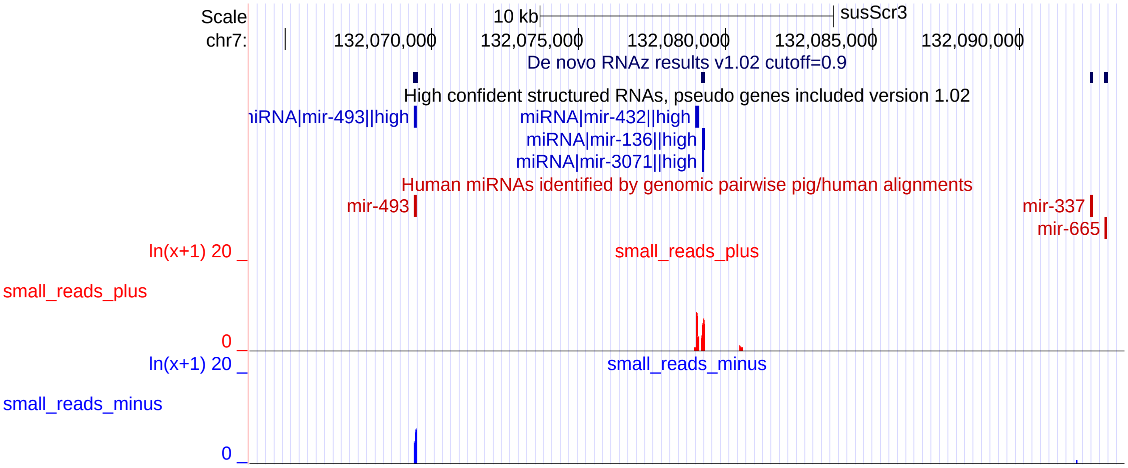

Figure S14 - mir-532 cluster in pig

The mir-532 cluster is mostly conserved in pig, however, some miRNAs, *e.g.* mir-501 and mir-502 are poorly conserved. See main text for details. In the figure, the `RNAz` track is colored dark blue; the high confident confident annotations based are colored with a lighter blue; the curated annotation is colored green; the human miRNAs from miRBase, which have been lifted over from hg19 to pig are colored red; the number of reads from the small RNA library that cover each base, is shown in the bottom of each plot (blue for reads on the negative strand and red for reads on the positive strand).

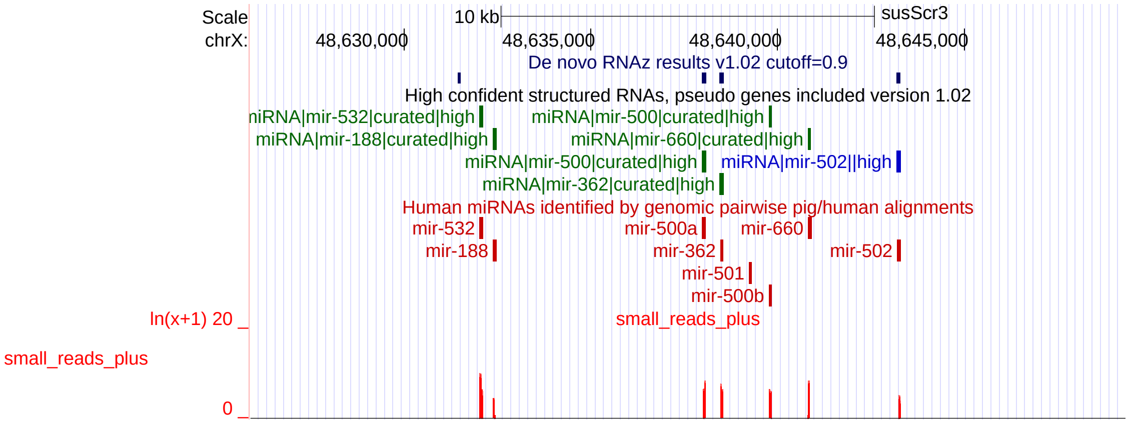

Figure S15 - mir-17 cluster in pig

The mir-17 cluster is well conserved in pig and the miRNAs are supported by reads. In the figure, the RNAz track is colored dark blue; the high confident confident annotations based are colored with a lighter blue; the curated annotation is colored green; the human miRNAs from miRBase, which have been lifted over from hg19 to pig are colored red; the number of reads from the small RNA library that cover each base, is shown in the bottom of each plot (blue for reads on the negative strand and red for reads on the positive strand).

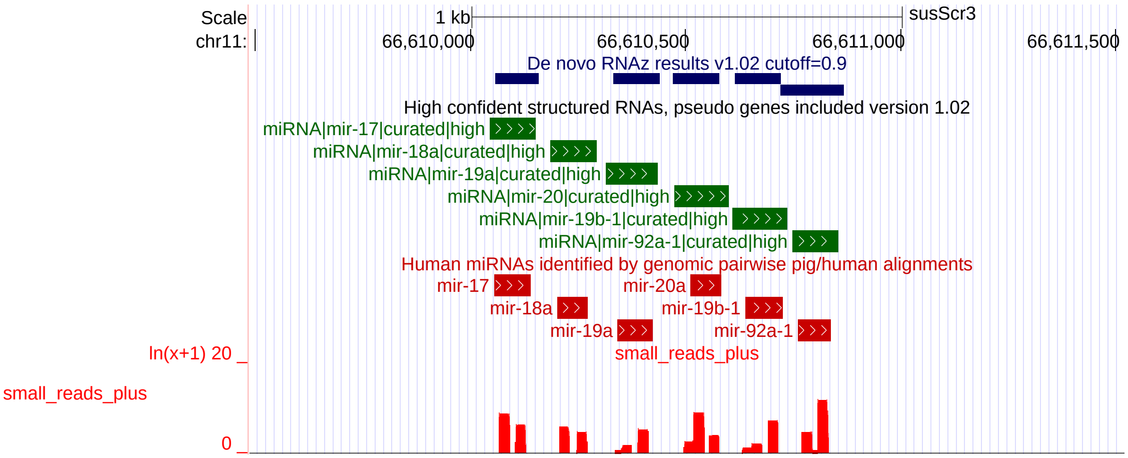

Figure S16 - mir-363 cluster in pig

The mir-363 cluster is well conserved in pig and the miRNAs are supported by reads. In the figure, the `RNAz` track is colored dark blue; the high confident confident annotations based are colored with a lighter blue; the curated annotation is colored green; the human miRNAs from miRBase, which have been lifted over from hg19 to pig are colored red; the number of reads from the small RNA library that cover each base, is shown in the bottom of each plot (blue for reads on the negative strand and red for reads on the positive strand).

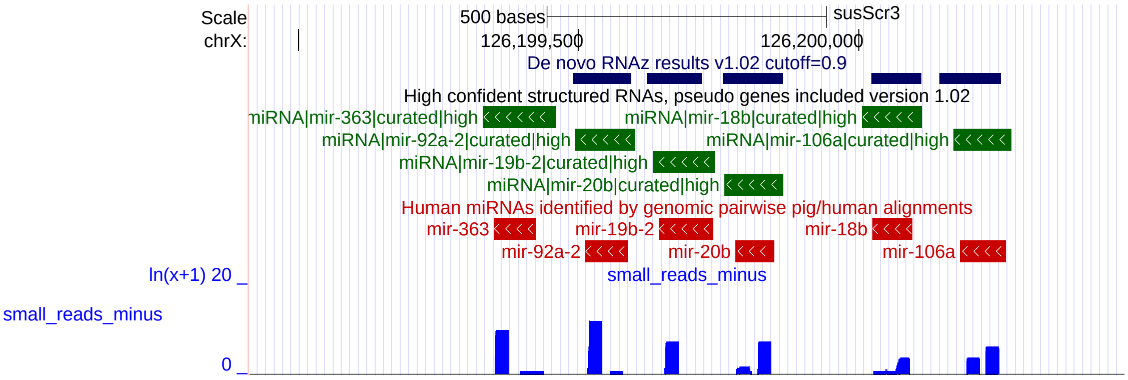

Figure S17 - mir-367 cluster in pig

Only 3 of the 5 miRNAs in the mir-367 cluster are identified by high confident `BLAST`, however, the cluster appears complete in the pairwise alignment. In the figure, the `RNAz` track is colored dark blue; the high confident confident annotation based on `BLAST` is colored with a lighter blue, except for the curated annotation, which is colored green; the human miRNAs from miRBase which have been lifted over from hg19 to pig are colored red; the number of reads from the small RNA library that cover each base, is shown in the bottom of each plot (blue for reads on the negative strand and red for reads on the positive strand).

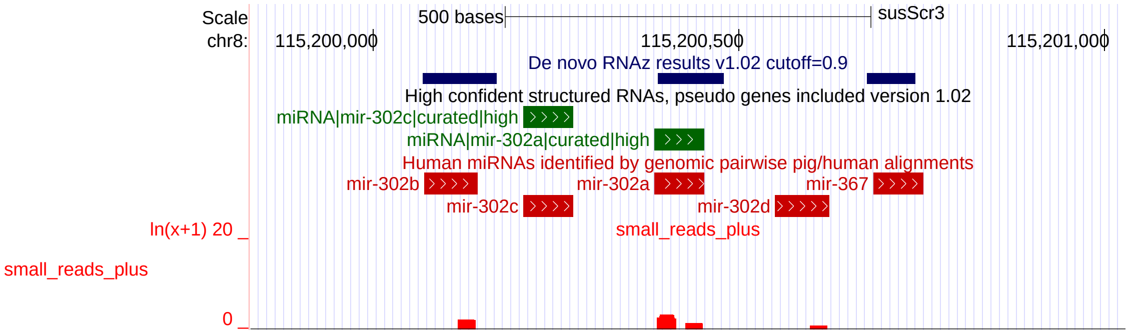

Figure S18 - mir-513a cluster in pig

The mir-513a and mir-514b clusters are incomplete in the pig genome, leading to several problems in the pairwise alignments. Three miRNAs are observed in the `miRDeep` analysis of these clusters, which may be assigned as mir-506, mir-508 and likely mir-509. The mir-509 miRNA is confirmed by high confident `BLAST` and is therefore curated. In the figure, the `RNAz` track is colored dark blue; the high confident confident annotations based are colored with a lighter blue; the curated annotation is colored green; the human miRNAs from miRBase, which have been lifted over from hg19 to pig are colored red; the number of reads from the small RNA library that cover each base, is shown in the bottom of each plot (blue for reads on the negative strand and red for reads on the positive strand).

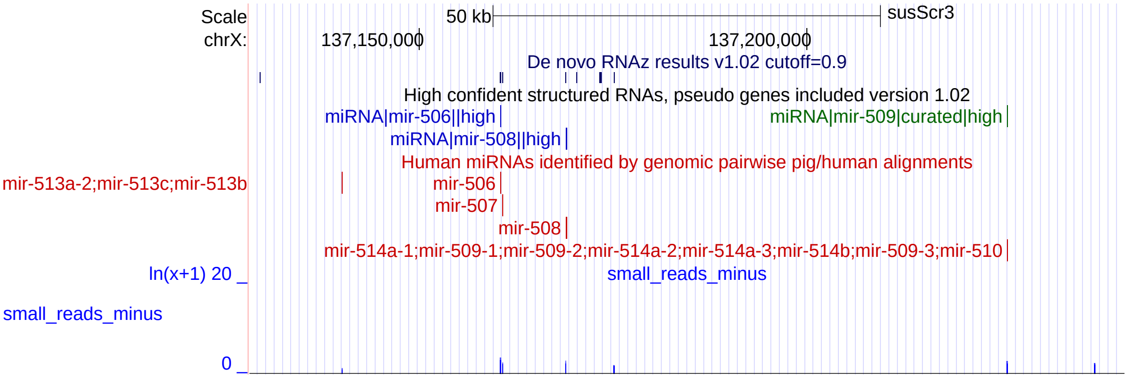

Figure S19 - mir-450b cluster in pig

The mir-450b cluster consists of 6 miRNAs in human, but it is incomplete in the pig assembly. The miRNAs that we find are supported by both BLAST and reads from the small RNA library. In the figure, the RNAz track is colored dark blue; the high confident confident annotations based are colored with a lighter blue; the curated annotation is colored green; the human miRNAs from miRBase, which have been lifted over from hg19 to pig are colored red; the number of reads from the small RNA library that cover each base, is shown in the bottom of each plot (blue for reads on the negative strand and red for reads on the positive strand).

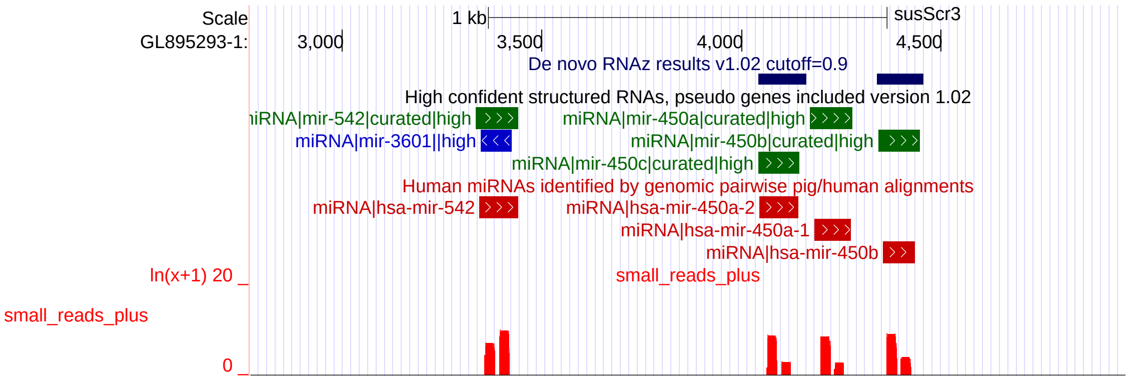

Figure S20 - mir-892c cluster in pig

The mir-892 cluster consists of 6 miRNAs in human, neither of these are found in pig by high confident `BLAST`. However, the entire cluster is reproduced in pig according to the human-pig pairwise alignments. We do not annotate these miRNAs, they are supported by neither high confident `BLAST`, nor reads from the small RNA library. In the figure, the `RNAz` track is colored dark blue; the high confident confident annotations based are colored with a lighter blue; the curated annotation is colored green; the human miRNAs from miRBase, which have been lifted over from hg19 to pig are colored red; the number of reads from the small RNA library that cover each base, is shown in the bottom of each plot (blue for reads on the negative strand and red for reads on the positive strand).

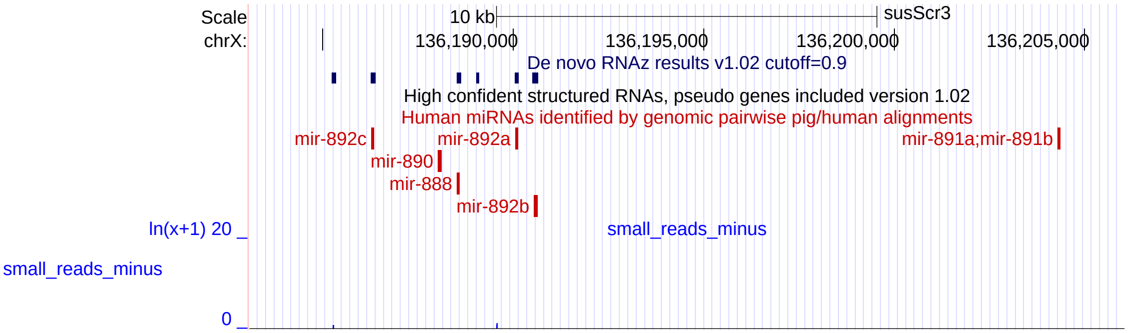

Figure S21 - False positives in the `Infernal` results
Number of hits in the shuffled pig sequence from `Infernal`/Rfam models at their family specific gathering score cutoffs versus a global `Infernal` E-value cutoff. The graph is limited to the 514 families with hits in the un-shuffled pig sequence.

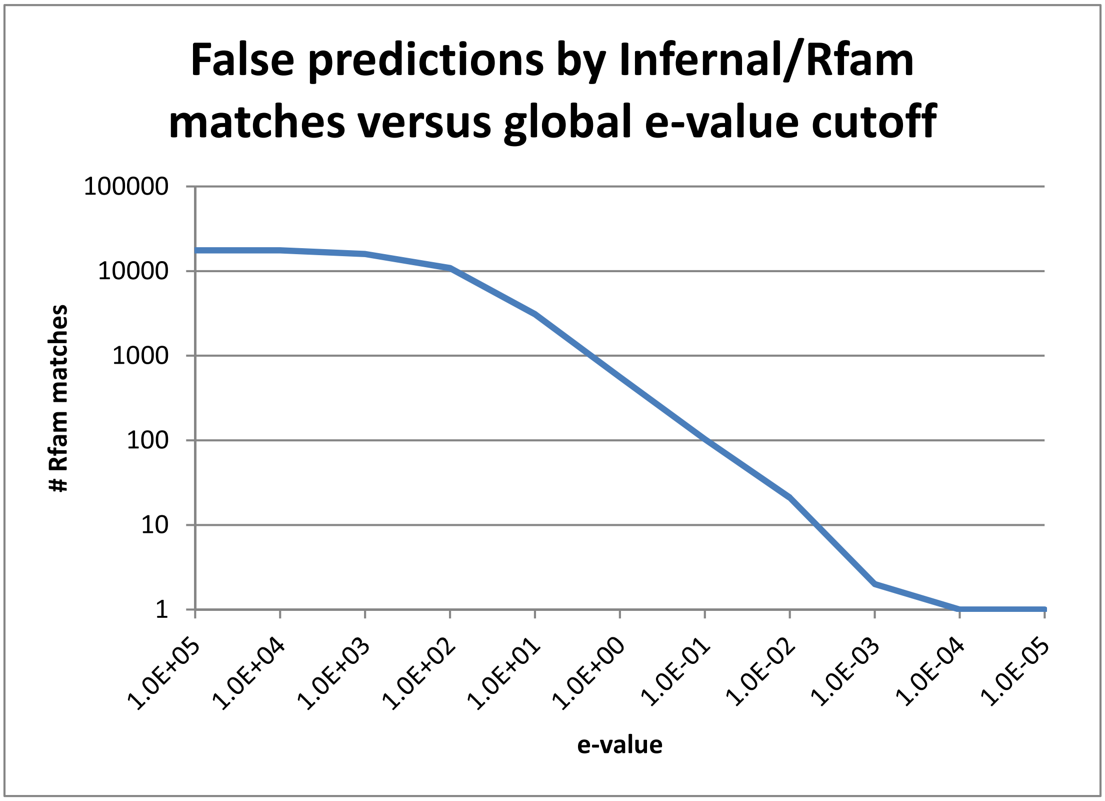

Figure S22 - PolIII: Logo PSE element and TATA-box

l.h.s Logo of the PSE element found for the PolIII transcript in human. r.h.s Logo of the TATA-Box found for the PolIII transcript in human

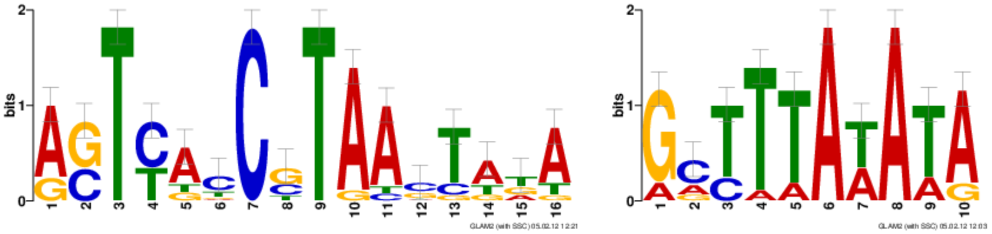

Figure S23 - Density plot of the position distribution of the PSEA elements

l.h.s Density plot of the position distribution of the PSEA elements for the putative PolII sequences (black lines) and random sequences (red lines). The doted lines represent the PSEA distribution for scores belonging to the highest 20% quantile. The peak clearly shows that PSEA is preferentially found 50nts upstream of the transcript start. r.h.s Density plot of the distribution of the TATA-Box location for putative PolIII sequences (black line) and a set of 1000 random sequences (red line). TATA-Boxes are preferentially found directly upstream of the transcript start.

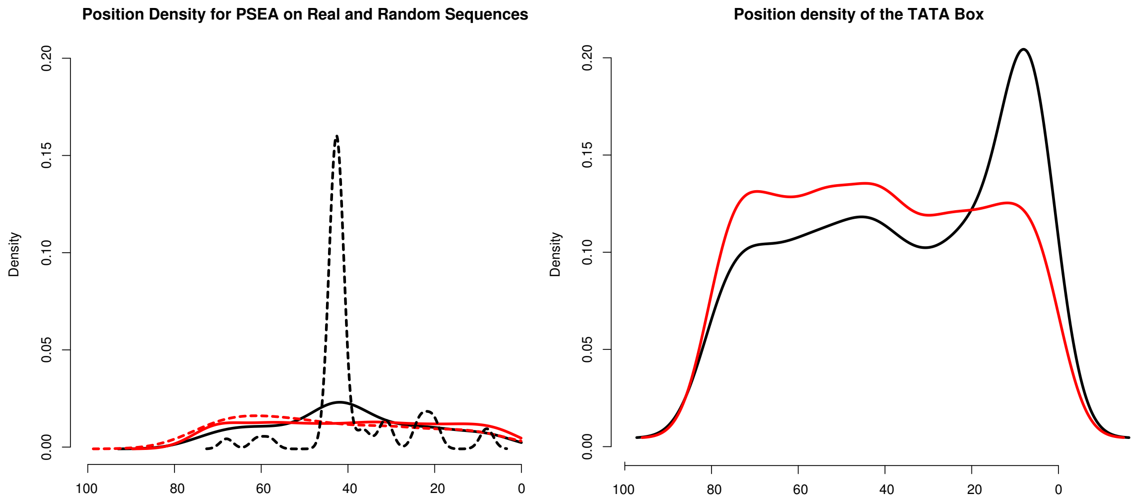

Figure S24 - PolII: Logo of PSE element and TATA-box

l.h.s Logo of the PSE element found for the PolII transcript in human. r.h.s Logo of the TATA-Box found for the PolII transcript in human.

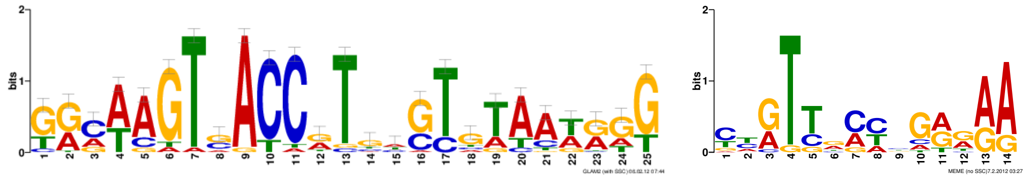

References

1. **Kozomara A, Griffiths-Jones S: miRBase: integrating microRNA annotation and deep-sequencing data. *Nucleic Acids Research* 2011, 39(suppl 1):D152–D157.**

2. **Griffiths-Jones S, Saini HK, van Dongen S, Enright AJ: miRBase: tools for microRNA genomics. *Nucleic Acids Research* 2008, 36(suppl 1):D154–D158.**

3. **Griffiths-Jones S, Grocock RJ, van Dongen S, Bateman A, Enright AJ: miRBase: microRNA sequences, targets and gene nomenclature. *Nucleic Acids Research* 2006, 34(suppl 1):D140–D144.**

4. **Griffiths-Jones S: The microRNA Registry. *Nucleic Acids Research* 2004, 32(suppl 1):D109–D111.**

5. **Gardner PP, Daub J, Tate JG, Nawrocki EP, Kolbe DL, Lindgreen S, Wilkinson AC, Finn RD, Griffiths-Jones S, Eddy SR, Bateman A: Rfam: updates to the RNA families database. *Nucleic Acids Research* 2009, 37(suppl 1):D136–D140.**

6. **Gardner PP, Daub J, Tate J, Moore BL, Osuch IH, Griffiths-Jones S, Finn RD, Nawrocki EP, Kolbe DL, Eddy SR, Bateman A: Rfam: Wikipedia, clans and the “decimal” release. *Nucleic Acids Research* 2010.**

7. **Pruesse E, Quast C, Knittel K, Fuchs BM, Ludwig W, Peplies J, Glöckner FO: SILVA: a comprehensive online resource for quality checked and aligned ribosomal RNA sequence data compatible with ARB. *Nucleic Acids Research* 2007, 35(21):7188–7196.**

8. **Lestrade L, Weber MJ: snoRNA-LBME-db, a comprehensive database of human H/ACA and C/D box snoRNAs. *Nucleic Acids Research* 2006, 34(suppl 1):D158–D162.**

9. **Jühling F, Mörl M, Hartmann RK, Sprinzl M, Stadler PF, Pütz J: tRNAdb 2009: compilation of tRNA sequences and tRNA genes. *Nucleic Acids Research* 2009, 37(suppl 1):D159–D162.**
